# MeSi(CH_2_SnRO)_3_ (R=Ph, Me_3_SiCH_2_): Building Blocks for Triangular‐Shaped Diorganotin Oxide Macrocycles

**DOI:** 10.1002/anie.202012248

**Published:** 2020-10-19

**Authors:** Jihed Ayari, Christian R. Göb, Iris M. Oppel, Michael Lutter, Wolf Hiller, Klaus Jurkschat

**Affiliations:** ^1^ Fakultät für Chemie und Chemische Biologie Technische Universität Dortmund Otto-Hahn-Straße 6 44227 Dortmund Germany; ^2^ Institut für Anorganische Chemie RWTH Aachen 52056 Aachen Germany

**Keywords:** DOSY NMR spectroscopy, electrospray-ionization mass spectrometry, macrocycles, organotin oxides, X-ray crystallography

## Abstract

The syntheses of the novel silicon‐bridged *tris*(tetraorganotin) compounds MeSi(CH_2_SnPh_2_R)_3_ (**2**, R=Ph; **5**, R=Me_3_SiCH_2_) and their halogen‐substituted derivatives MeSi(CH_2_SnPh_(3−*n*)_I_*n*_)_3_ (**3**, *n*=1; **4**, *n*=2) and MeSi(CH_2_SnI_2_R)_3_ (**6**, R=Me_3_SiCH_2_) are reported. The reaction of compound **4** with di‐*t*‐butyltin oxide (*t*‐Bu_2_SnO)_3_ gives the oktokaideka‐nuclear (18‐nuclear) molecular diorganotin oxide [MeSi(CH_2_SnPhO)_3_]_6_ (**7**) while the reaction of **6** with sodium hydroxide, NaOH, provides the trikonta‐nuclear (30‐nuclear) molecular diorganotin oxide [MeSi(CH_2_SnRO)_3_]_10_ (**8**, R=Me_3_SiCH_2_). Both **7** and **8** show belt‐like ladder‐type macrocyclic structures and are by far the biggest molecular diorganotin oxides reported to date. The compounds have been characterized by elemental analyses, electrospray mass spectrometry (ESI‐MS), NMR spectroscopy, ^1^H DOSY NMR spectroscopy (**7**), IR spectroscopy (**7**, **8**), and single‐crystal X‐ray diffraction analysis (**2**, **7**, **8**).

## Graphical abstract

The first representatives of a new generation of large molecular diorganotin oxides showing Reuleaux triangle‐shaped (**7**) and heart‐formed (**8**) structures are reported. Based on simple syntheses of a great variety of precursor molecules, the concept holds enormous potential for future work in supramolecular chemistry involving main‐group elements and beyond.

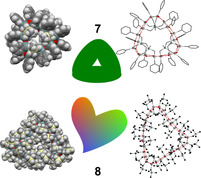

## Introduction

Metal chalcogenides with the empirical formula (M_*x*_E_*y*_)_*n*_ (M=transition or main group metal; E=O, S, Se; *n*=infinite) are classical ionic compounds found as ores in nature. Their reactivity and structures in the solid state have been extensively studied since the beginning of chemical research and chemistry textbooks describe essential topics of these compounds.[^1a^, ^1b^, ^1c^, ^1d^, ^1e^] For a long time, metal chalcogenides mainly served as raw materials for the metallurgy. However, over the years, chemists learned also about some intriguing physical properties of these compounds, such as semiconductivity,[^1a^, ^1f^] nonlinear optoelectronic behaviour,^[1g]^ and thermochromism,[^1a^, ^1b^, ^1c^, ^1d^, ^1e^] just to mention three out of many. Such properties are of utmost importance for high‐tech applications. Academic curiosity and even more the need for a better understanding of structure‐property relationships motivated chemists trying to cut out molecular entities from the three‐dimensional polymeric metal chalcogenides. This was achieved by formally replacing metal‐chalcogen bonds by a great variety of metal‐ligand bonds, with the ligands being inorganic as well as organic moieties. As a result, the concept of polynuclear metal chalcogenido clusters was established and the achievements made over the years for both main group‐ and transition metal‐containing such clusters were regularly reviewed.^[2]^ This type of chemistry is also well established for the element tin. Randomly selected representatives are (RSn)_4_E_6_ (R=organic substituent with or without additional functionality, E=O,^[3]^ S,^[4]^Se^[4]^) showing adamantane‐ or double decker‐type structures, dodecanuclear tinoxo clusters {(RSn)_12_O_14_(OH)_6_} (R=*i*‐Pr, *n*‐Bu, Me_3_SiCH_*2*_, ferrocenyl),^[5]^ Sn_12_O_8_(OH)_4_(OEt)_28_(HOEt)_4_,^[6a]^ [(BuSn)_12_(μ_3_‐O)_14_(μ_2_‐OH)_6_](L^1^)_2_⋅2 EtOH,^[6b]^ [NaO_4_(BuSn)_12_(OH)_3_(O)_9_(OCH_3_)_12_(Sn(H_2_O)_2_)],^[6c]^ and [NaO_4_BuSn_12_(OCH_3_)_12_(O,OH)_12_],^[6d]^ tetraorganodistannoxanes {R_2_(X)SnOSn(X)R_2_}_2_ (R=organic substituent, X=electronegative substituent),^[7]^ [{R(X)Sn(CH_2_)_*n*_Sn(X)R}O]_4_ (R=organic substituent; X=halide, hydroxide, carboxylate; *n*=1,^[8a]^ 3–8, 10, 12^[8b]^), [(R_2_SnO)_3_(R_2_SnOH)_2_CO_3_]_2_ (R=organic substituent),^[9]^ and [(2,4,6‐*i*‐Pr_3_C_6_H_2_Sn)_8_(μ_4_‐O)_2_(μ_3_‐O)_8_‐(μ_2_‐O)_4_(μ_2_OH)_8_{Sn(OH)}_4_].^[10]^ Recently, this well‐established chemistry got new momentum by the spectacular findings reported by *S. Dehnen* et al. about laser‐induced white light‐emitting ability of the simple styryltin silsesquisulphide [(StySn)_4_S_6_],^[11]^ but also by extending the Sn‐nuclearity of tinoxo clusters to the impressive number 34 in [(*n*‐BuSn)_34_Na_2_(OH)_14_O_40_(PA)_8_]⋅2(PA)⋅8 H_2_O (PA=propionic acetate), as published by *L. Zhang* et al.^[12]^ One aspect from these studies is that the steric bulk and identity of the substituents bound to the tin centre control the nuclearity of the clusters. For organic substituents R, the general trend is that, as result of reactions with water or hydroxide, monoorganotin precursors RSnX_3_ (X=halogen, alkoxide, carboxylate) tend to give clusters of higher nuclearity than diorganotin precursors R_2_SnX_2_ do. On the other hand, triorganotin compounds R_3_SnX can only give distannoxanes R_3_SnOSnR_3_ as result of such reactions. However, in combination with appropriately designed ligands and the concept of self‐assembly, they as well as diorganotin precursors R_2_SnX_2_ may also serve for the formation of large‐membered polynuclear rings.^[13]^

**Scheme 1:**
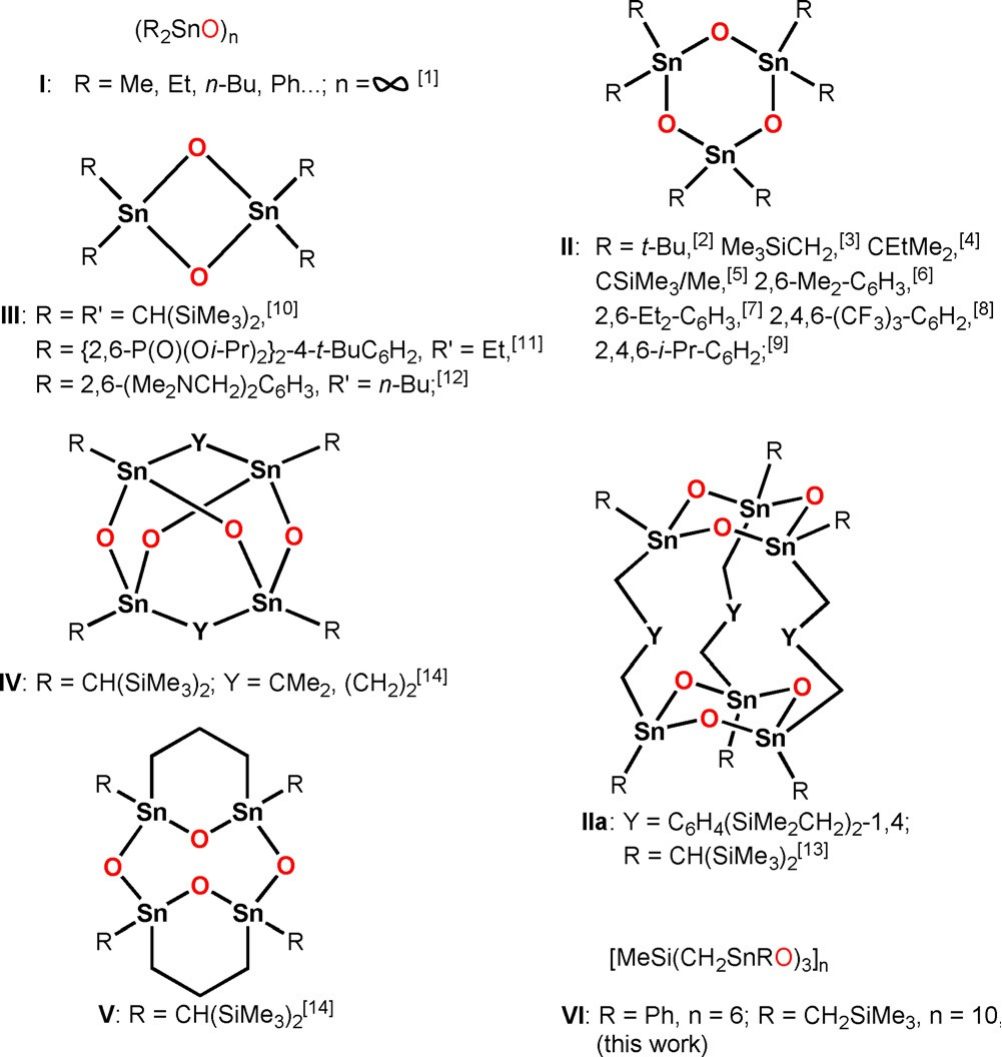
Different types of diorganotin oxides.

It is common knowledge that the complete replacement of the electronegative substituents X in diorganotin compounds of type R_2_SnX_2_ (X=halogen, alkoxide, carboxylate) with oxide dianion gives the corresponding diorganotin oxides (R_2_SnO)_*n*_. Depending on the identity of the organic substituents R, these oxides can either be polymeric (type **I**, *n*=∞),^[14]^ trimeric (type **II**, *n*=3)[^15^, ^16^, ^17^, ^18^, ^19^, ^20^, ^21^, ^22^] or even dimeric (type **III**, *n*=2) (Scheme 1).[^23^, ^24^, ^25^] Sterically less demanding organic substituents such as *n*‐alkyl or phenyl give polymers. These, because of intermolecular O→Sn interactions making the tin atoms five‐ or even six‐coordinate, are almost insoluble in common organic solvents. Increasing the steric bulk of the organic substituents enables the formation of six‐ or even four‐membered rings in which the tin atoms are four‐coordinate. The same principle holds for the formation of the molecular diorganotin oxides of types **IIa** (in which two parallel six‐membered Sn_3_O_3_ rings are linked to each other by three organic spacers),^[26]^ **IV** (adamantane‐type structure),^[27]^ and **V** (the only crystallographically characterized diorganotin oxide containing an eight‐membered Sn_4_O_4_ ring).^[27]^ More recently, intramolecular N→Sn or P=O→Sn coordination proved to be alternatives to steric bulk for the stabilization of type **III** diorganotin oxides.[^24^, ^25^] Considering what is stated above, we pose the question whether diorganotin oxides can be synthesized the structures of which are in between the polymers of type **I** on the one hand and the eight‐, six‐, and four‐membered rings of types **II**, **III**, and **V** on the other hand. Herein, we report that diorganotin diiodide precursors containing sterically less‐crowded substituents (Ph, Me_3_SiCH_2_), but having a particular tripod architecture give, by reaction with appropriate oxide, respectively hydroxide anion releasing reagents, cyclic polynuclear molecular diorganotin oxides (Scheme 1, type **VI**) of unprecedented large sizes. In these, the tin atoms adopt the coordination number five.

## Results and Discussion

**Scheme 2:**
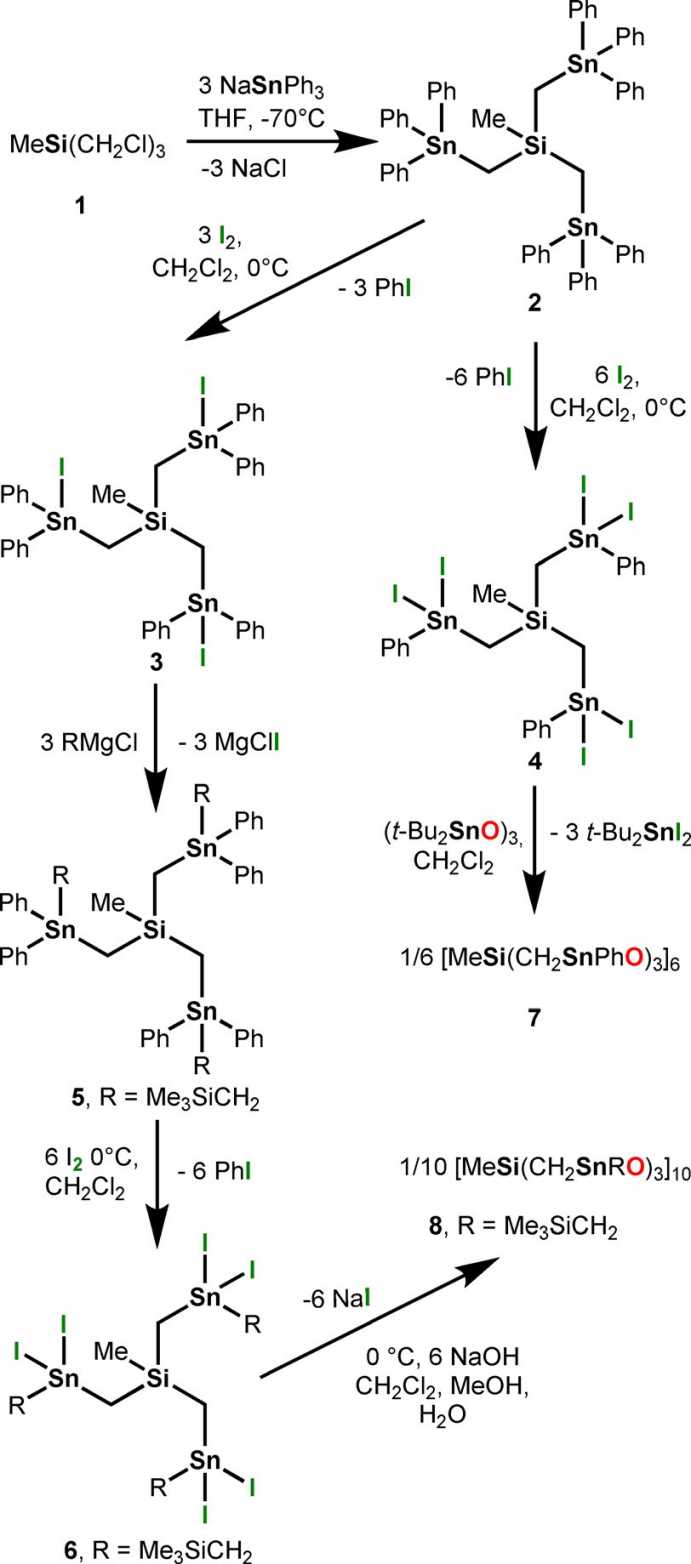
Syntheses of the compounds **2**–**8**.

The reaction in tetrahydrofuran of methyl‐*tris*(chloromethyl) silane, MeSi(CH_2_Cl)_3_ (**1**)^[28]^ with three molar equiv triphenyl sodium stannide, NaSnPh_3_, gave *tris*(triphenylstannylmethyl) silane, MeSi(CH_2_SnPh_3_)_3_ (**2**) as colourless crystalline material in almost quantitative yield (Scheme 2). Compound **2** easily converted to the *tris*(mono) and *tris*(di) halogenated derivatives MeSi(CH_2_SnIPh_2_)_3_ (**3**) and MeSi(CH_2_SnI_2_Ph)_3_ (**4**) by its reaction with three, respectively six molar equiv of elemental iodine (Scheme 2). The treatment of **3** with three molar equiv trimethylsilylmethylmagnesium chloride, Me_3_SiCH_2_MgCl, gave the corresponding tetraorganotin derivative MeSi[CH_2_Sn(CH_2_SiMe_3_)Ph_2_]_3_ (**5**) which, by reaction with six molar equiv elemental iodine, provided MeSi[CH_2_Sn(CH_2_SiMe_3_)I_2_]_3_ (**6**). Compound **2** is a colourless crystalline material while its derivatives **3**–**6** represent slightly yellow oils. All compounds show good solubility in common organic solvents such as CH_2_Cl_2_, CHCl_3_, THF, and CH_3_CN. The Supporting Information contains the analytical data including the molecular structure of **2** (Figure S12) as determined by single crystal X‐ray diffraction analysis. The data are as expected and confirm unambiguously the identities of compounds **2**–**6**.

Treatment in CH_2_Cl_2_ of the organotin iodide **4** with (*t*‐Bu_2_SnO)_3_ gave a reaction mixture a ^119^Sn NMR spectrum of which displayed four resonances at *δ* 61 (*t*‐Bu_2_SnI_2_), −203 (**7**), −225 (**7**), and −228 ppm (**7**), respectively (Supporting Information, Figure S59). The spectrum indicates complete oxygen transfer from (*t*‐Bu_2_SnO)_3_ to the organotin iodide **4** and formation of *t*‐Bu_2_SnI_2_ and the oktokaideka‐nuclear (18‐nuclear) organotin oxide [MeSi(CH_2_SnPhO)_3_]_6_, **7** (Scheme 2). The latter compound was isolated from the reaction mixture as colourless crystalline material.

**Figure 1:**
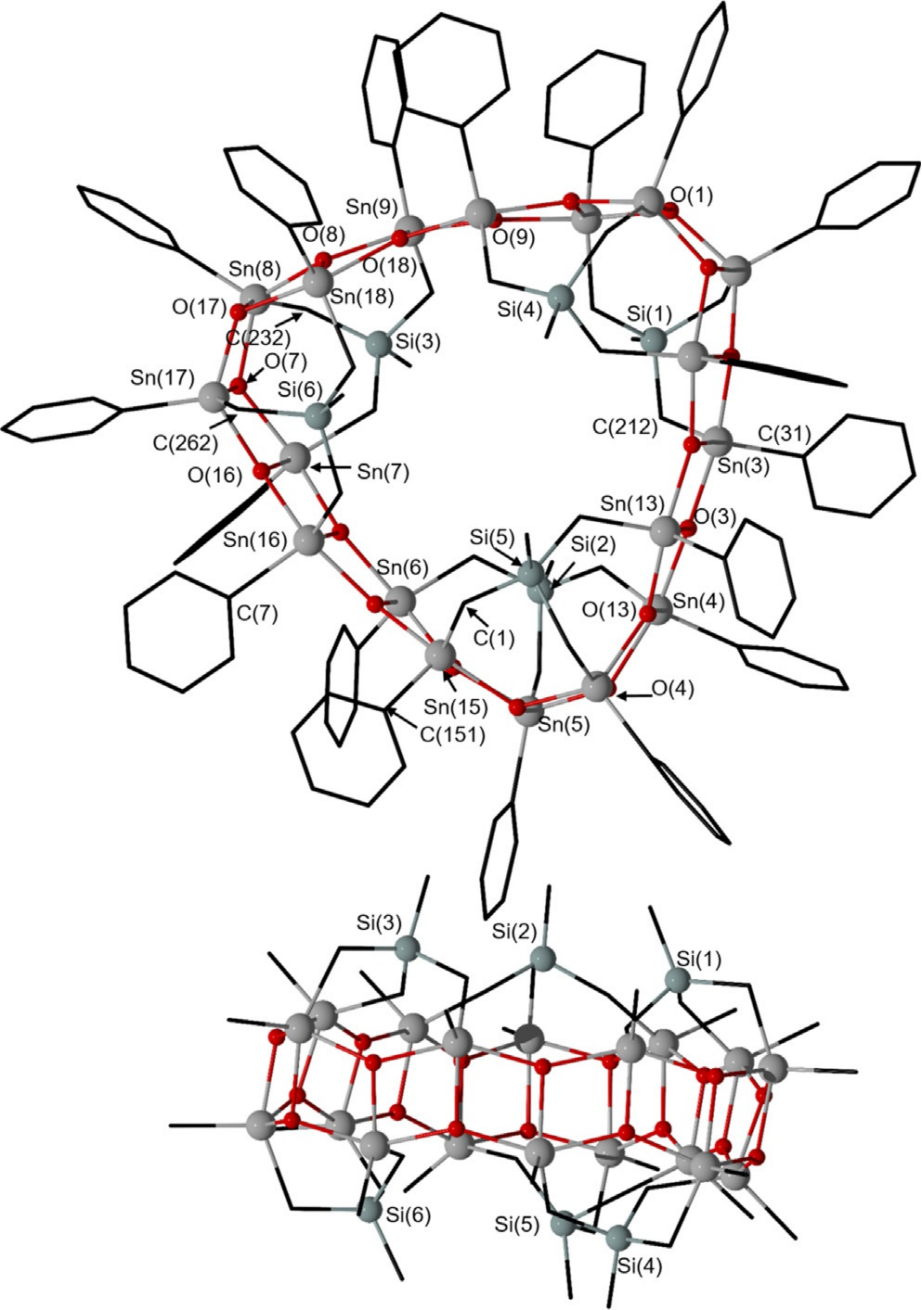
Top: General view (ball and stick) of a molecule of the organotin oxide **7** containing the numbering of the atoms that appear below in the listing of distances and angles. The hydrogen atoms are omitted for clarity. Bottom: Side view of a molecule of **7** including the numbering for the silicon atoms. Selected interatomic distances (Å): Sn‐C 2.05(2) (Sn6‐C707) −2.256(17) (Sn16‐C265), Sn‐O_ax_ 2.074(8) (Sn13‐O13) −2.158(7) Å (Sn4‐O4), Sn‐O_equ_ 2.009(9) (Sn7‐O16) −2.055(8) Å (Sn9‐O18). Selected interatomic angles: C_equ_‐Sn‐C_equ_ 111.6(4) (C31‐Sn3‐C212) −138.1(7)° (C1‐Sn15‐C151), O_ax_‐Sn‐O_ax_ 147.8(3) (O3‐Sn4‐O4) −150.8(3)° (O17‐Sn18‐O18), C_ax_‐Sn‐O_ax_ 150.0(4) (C262‐Sn17‐O7) −153.4(4)° (C232‐Sn8‐O17).

It crystallized as a solvate from dichloromethane solution. Figure 1 shows its molecular structure. The Figure caption contains selected interatomic distances and angles. Compound **7** crystallizes in the monoclinic space group *P*2_**1**_/*n* with four crystallographic equivalent molecules in the unit cell. Each of these contains six MeSi(CH_2_SnPh)_3_ moieties in which the tin atoms are connected by a total of 18 oxygen atoms giving a triangular shaped, belt‐like macromolecule with diameters ranging between 20.06(1) (H44⋅⋅⋅H94) and 23.00(1) (H84⋅⋅⋅H144) and a thickness ranging between 10.47(1) (H55⋅⋅⋅H145) and 10.97(1) Å (H5⋅⋅⋅H154) (Supporting Information Figure S51). Three MeSi moieties (containing Si1–Si3) are located above and three such moieties (containing Si4–Si6) are located below the belt formed by the 18 tin and 18 oxygen centres (Figure 1; Supporting Information, Figure S54).

Each of the 18 crystallographic independent tin atoms is penta‐coordinated and shows a distorted trigonal bipyramidal environment. For each of the Sn(1), Sn(3), Sn(4), Sn(6), Sn(7), Sn(9), Sn(10), Sn(12), Sn(13), Sn(15), Sn(16), and Sn(18) atoms, the two carbon atoms (C_*i*_ atom from the phenyl substituent and the methylene carbon atom) and one oxygen atom occupy the equatorial positions. The corresponding C_eq_‐Sn‐C_eq_ angles vary between 111.6(4) (C31‐Sn3‐C212) and 138.1(7)° (C1‐Sn15‐C151). Two oxygen atoms take the axial positions with the O_ax_‐Sn‐O_ax_ angles varying between 147.8(3) (O3‐Sn4‐O4) and 150.8(3)° (O17‐Sn18‐O18). The corresponding Sn‐O_ax_ distances vary between 2.074(8) (Sn13‐O13) and 2.158(7) Å (Sn4‐O4). The Sn‐O_equ_ distances involving oxygen atoms in equatorial positions are slightly shorter and vary between 2.009(9) (Sn7‐O16) and 2.055(8) Å (Sn9‐O18). Notably, for the Sn(2), Sn(5), Sn(8), Sn(11), Sn(14), and Sn(17) atoms the corresponding methylene carbon atom and one out of the adjacent three oxygen atoms take the axial positions whereas the C_*i*_ and the two remaining oxygen atoms occupy the equatorial positions. This is in contrast to a situation as expected from the polarity rule^[29]^ according to which the electronegative substituents occupy the axial positions in a trigonal bipyramidal structure. The C_ax_‐Sn‐O_ax_ angles vary between 150.0(4) (C262‐Sn17‐O7) and 153.4(4)° (C232‐Sn8‐O17).

**Figure 2:**
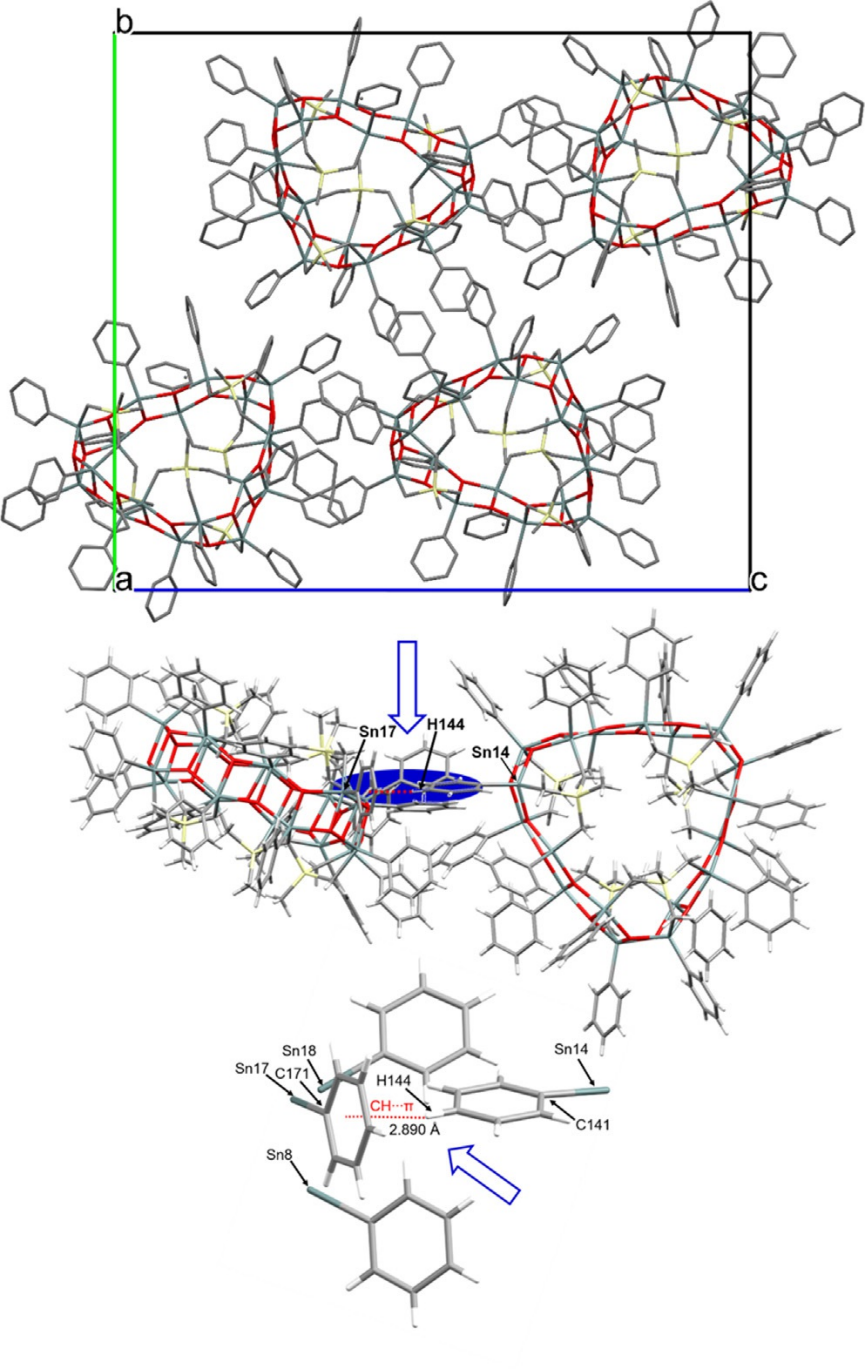
Top: Crystal packing of **7**. The hydrogen atoms are omitted for clarity. Bottom: Illustration of the C−H⋅⋅⋅π interactions at a H(144)‐centroid (C171‐C176) distance of 2.89(1) Å.

The crystal structure of **7** (Figure 2) is characterized by C−H⋅⋅⋅π interactions at a H(144)‐centroid (C171–C176) distance of 2.89(1) Å.

**Figure 3:**
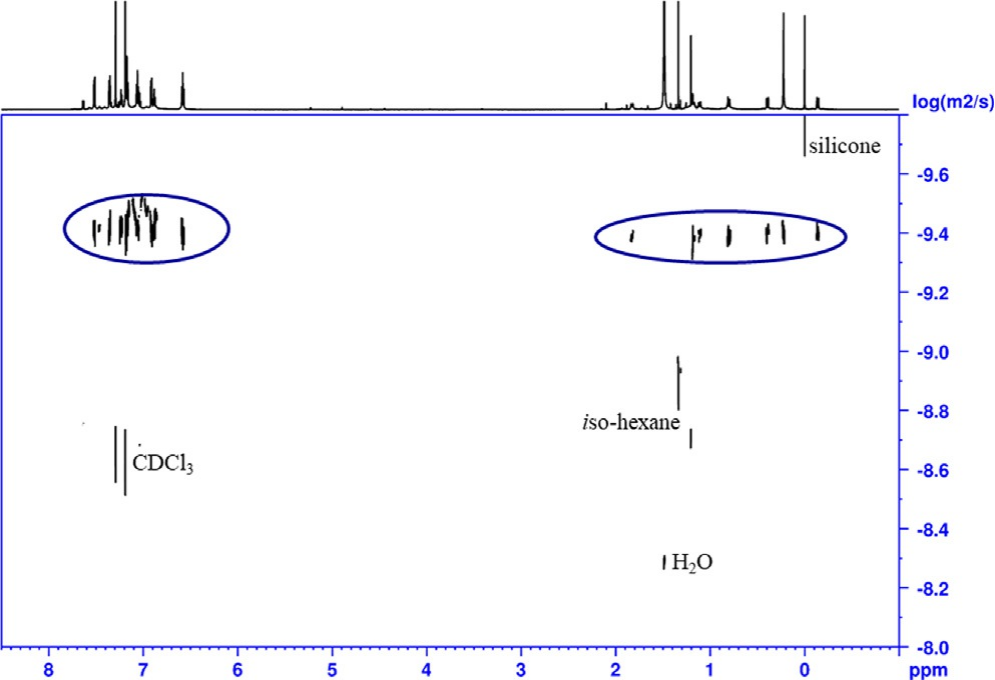
2D ^1^H DOSY NMR spectrum of [MeSi(CH_2_SnPhO)_3_]_6_, **7**, in CDCl_3_.

The identity of compound **7** is retained in solution. The compound is kinetically inert on the ^1^H (for details see Supporting Information), and ^119^Sn NMR time scales. Thus, a ^119^Sn NMR spectrum of a solution of single crystalline **7** in CDCl_3_ shows three equally intense resonances at *δ*−204 ppm (^2^ *J*(^119^Sn‐^117/119^Sn) 180, 315 Hz; ^2^ *J*(^119^Sn‐^29^Si) 59 Hz), *δ*−225 ppm (^2^ *J*(^119^Sn‐^117/119^Sn) 315 Hz), and *δ*−228 ppm (^2^ *J*(^119^Sn‐^117/119^Sn) 180 Hz). The chemical shifts are in agreement with pentacoordinated tin atoms showing a SnC_2_O_3_ substituent pattern.^[30]^ A ^1^H DOSY NMR spectrum (CDCl_3_ solution, room temperature, Figure 3) provided a diffusion coefficient of 3.9(1)×10^−10^ m^2^ s^−1^. This, by using the Einstein‐Stokes equation, gave a calculated hydrodynamic diameter of 20.8 Å and a sphere volume of 4813 Å^3^. These values fit reasonably well with the single crystal X‐ray diffraction data discussed above.

Finally, an electrospray ionization mass spectrum (ESI MS; Supporting Information, Figures S66–S73) revealed a mass cluster centred at *m*/*z=*4324.1823 that corresponds to {[MeSi(CH_2_SnPhO)_3_]_6+_H^+^}, [**7**+H]^+^. In addition, there are mass clusters centred at *m*/*z* 1442.7312, *m*/*z* 2161.5910, 2884.4515, and *m*/*z* 3636.3600 that are assigned to {[MeSi(CH_2_SnPhO)_3_]_2_+H^+^}, {[MeSi(CH_2_SnPhO)_3_]_3_+H^+^}, {[MeSi(CH_2_SnPhO)_3_]_4_+H^+^}, and {[MeSi(CH_2_SnPhO)_3_]_5_ + MeOH + H^+^}, respectively.

The reaction of the diorganotin diiodide derivative **6** with sodium hydroxide, NaOH, in a mixture of dichloromethane, methanol, and water (Scheme 2) gave a crude reaction mixture a ^119^Sn NMR spectrum of which was rather complex and showed both broad and sharp resonances between −125 and −170 ppm (see Supporting Information, Figure S82). After the work‐up procedure, a microcrystalline material was obtained. From this, an extremely small crystal was picked and identified by single crystal X‐ray diffraction analysis as the molecular diorganotin oxide solvate **8**. Although the elemental analysis of the bulk crystalline material, obtained from the reaction between **6** and NaOH (Scheme 2), perfectly matches with the empirical formula [MeSi(CH_2_SnCH_2_SiMe_3_O)_3_]_*n*_, one cannot be sure whether it exclusively consists of the trikonta‐nuclear species **8** (*n*=10). Given the insufficient amount of material, no powder X‐ray diffraction analysis of the bulk material was performed.

**Figure 4:**
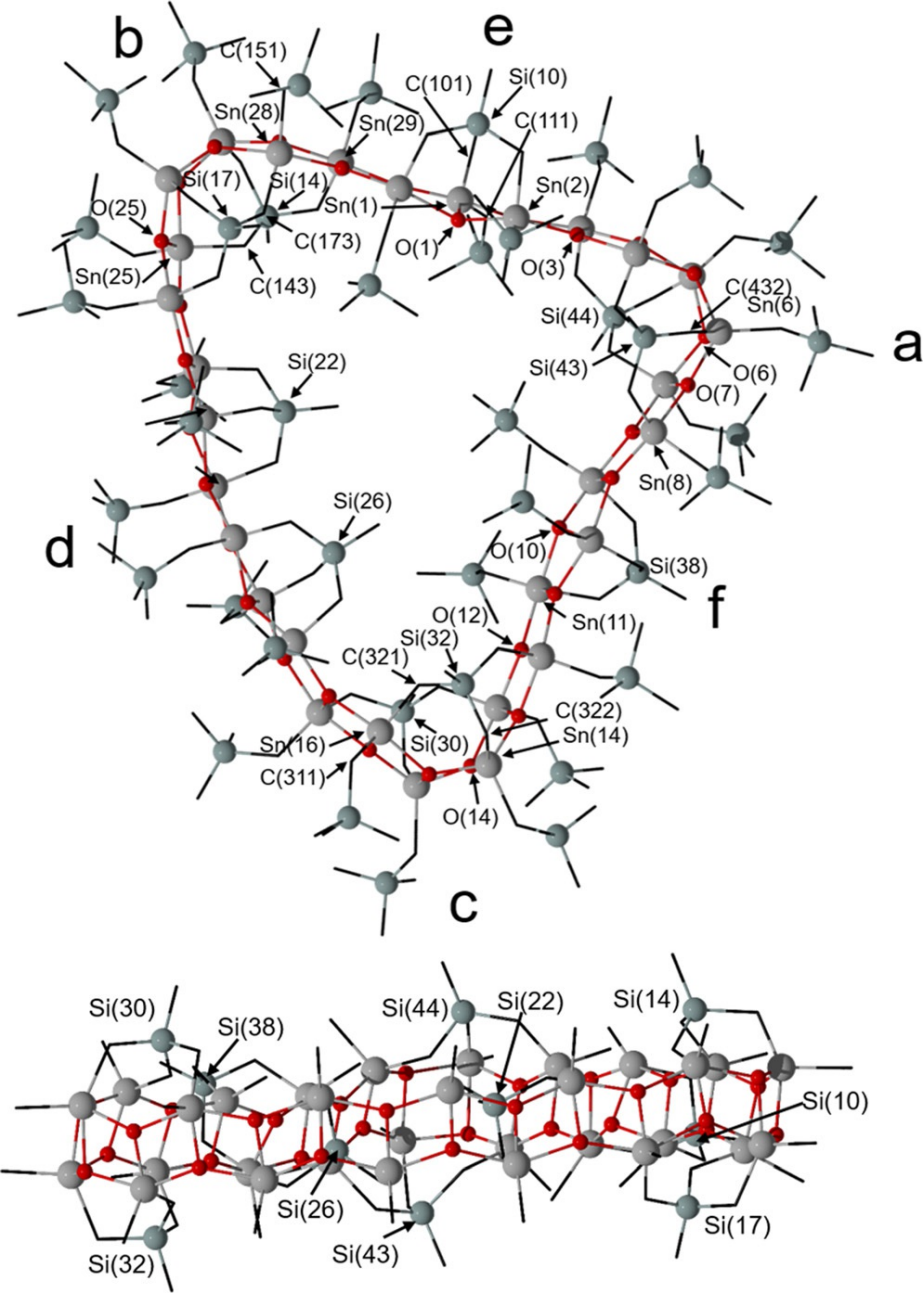
Top: General view (ball and stick) of a molecule of the organotin oxide **8** containing the numbering of the atoms that appear below in the listing of distances and angles. The hydrogen atoms are omitted for clarity. Bottom: Side view of a molecule of **8** including the numbering for the silicon atoms. The letters **a**–**f** refer to the different building blocks the belt‐type structure is composed of. Selected interatomic distances (Å). Sn‐O: 1.95(2) (Sn25‐O25, in **b**) −2.23(2) (Sn8‐O7, in **a**), Sn‐C: 2.01(8) (Sn1‐C111, in **e**) −2.38(4) (Sn28‐C151, in **b**). Selected interatomic angles (deg). O_ax_‐Sn‐O_ax_: 145.2(9) (O1‐Sn2‐O3, in **e**) −153.8(7)° (O10‐Sn11‐O12, in **f**), C_eq_‐Sn‐C_eq_: 119.0(10) (C311‐Sn16‐C321, in **c**) −139.8(15)° (C151‐Sn28‐C173, in **b**), C_ax_‐Sn‐O_ax_: 146.2(11) (O6‐Sn6‐C432, in **a**) −150.9(10)° (O14‐Sn14‐C322, in **c**). Figure S75 in the Supporting Information shows an image of the complete molecular structure including numbering of the atoms.

Figure 4 shows its simplified molecular structure and the Figure caption contains selected interatomic distances and angles. The compound crystallizes in the triclinic space group *P* $\bar{1}$ with two molecules in the unit cell.

**Figure 5:**
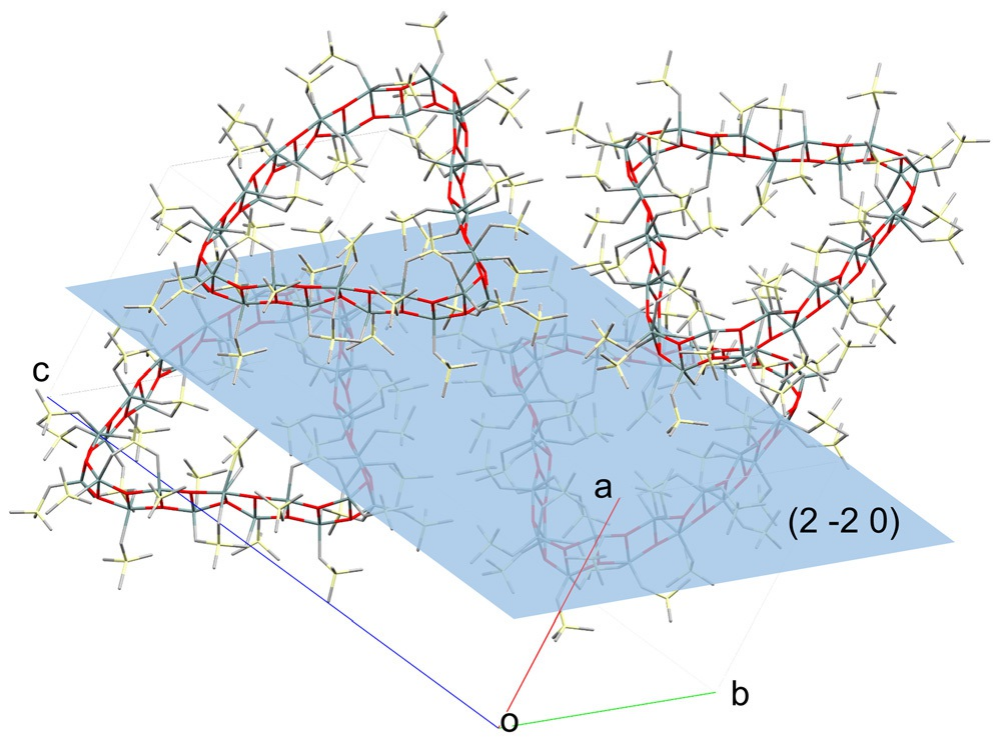
Crystal structure of **8**. The hydrogen atoms are omitted for clarity.

Compound **8** is a trikonta‐nuclear (30‐nuclear) molecular diorganotin oxide [MeSi(CH_2_SnRO)_3_]_10_ (R=Me_3_SiCH_2_) in which ten MeSi(CH_2_SnRO)_3_ moieties are connected giving a belt‐like ladder‐type heart‐shaped macrocycle (Figure 4). In this, the three methyl groups attached to Si(14), Si(30), and Si(44), respectively, are above and the three methyl groups attached to Si(17), Si(32), and Si(43), respectively, are below the ring plane. The two methyl groups attached to Si(22) and Si(26), respectively, point into the ring cavity and the two methyl groups attached to Si(10) and Si(38), respectively, point outside the ring (Supporting Information, Figure S77, left). The methylene carbon atoms C(111), C(121), C(371), C(391), C(401), and C(491) which are attached to Sn(1), Sn(30), Sn(11), Sn(10), Sn(9), and Sn(2), respectively and which belong to the trimethylsilylmethyl substituents point also inside the ring while the remaining substituents point outside (Supporting Information, Figure S76, right). The overall structure is rather complex. A closer inspection reveals it formally being composed of different subunits, i. e., the corner units **a** (with Sn3‐ Sn8), **b** (with Sn24–Sn29), and **c** (with Sn12–Sn17), the double spacer **d** (with Sn18–Sn23), and the single spacers **e** (with Sn1, Sn2, Sn30) and **f** (with Sn9–Sn11) (Figure 5).

Like in the oktokaideka‐nuclear diorganotin oxide **7**, all tin centres in **8** are five‐coordinated and, except Sn(1), show distorted trigonal bipyramidal environments. For the Sn(2)—Sn(4), Sn(7)–Sn(13), Sn(16)–Sn(25), Sn(29), and Sn(30) atoms, for each case two carbon atoms (the Me_3_Si*C*H_2_ and the MeSi*C*H_2_ methylene carbon atoms) and one oxygen atom occupy the equatorial positions. The other two oxygen atoms take the axial positions. The corresponding C_eq_‐Sn‐C_eq_ angles vary between 119.0(10) (C311‐Sn16‐C321) and 139.8(15)° (C151‐Sn28‐C173). The O_ax_‐Sn‐O_ax_ angles vary between 145.2(9) (O1‐Sn2‐O3) and 153.8(7)° (O10‐Sn11‐O12). The Sn(1) atom exhibits a distorted square pyramidal environment with the O(2), O(30), C(101), and C(111) atoms occupying the equatorial positions with O(2)‐Sn(1)‐O(30) and C(101)‐Sn(1)‐C(111) angles of 150.5(8) and 154(2)°, respectively. The O(1) atom occupies the apical position. The geometry about the Sn(28) atom is a borderline case between trigonal bipyramidal and square pyramidal with the O(27)‐Sn(28)‐O(29) and C(151)‐Sn(28)‐C(173) angles being 149.7(8) and 139.8(15)°. In analogy to **7**, there are again six tin centres (Sn5, Sn6, Sn26, Sn27, Sn14, Sn15) belonging to the corner units (**a**), (**b**), and (**c**), respectively, that violate in their coordination environment the polarity rule.^[29]^ For each of these tin centres, the corresponding MeSi*C*H_2_ methylene carbon atom and one out of the adjacent three oxygen atoms take the axial positions whereas the Me_3_Si*C*H_2_ methylene carbon and the two remaining oxygen atoms occupy the equatorial positions. The C_ax_‐Sn‐O_ax_ angles vary between 146.2(11) (O6‐Sn6‐C432, in **a**) and 150.9(10)° (O14‐Sn14‐C322, in **c**). Figure 5 shows the packing of **8** in the crystal. The Sn_30_O_30_ belt is located in the (2 −2 0) plane.

A ^119^Sn NMR spectrum of a CDCl_3_ solution of the crystalline bulk material the single crystal was taken from, (Supporting Information, Figure S83) revealed three, within the experimental error almost equally intense, resonances at *δ*−148 (^2^ *J*(^119^Sn‐^117/119^Sn)=230 Hz), *δ*−159 (^2^ *J*(^119^Sn‐^117/119^Sn)=257 Hz), and *δ*−164 ppm (^2^ *J*(^119^Sn‐^117/119^Sn)=219 Hz). In addition, there are broad, partially structured resonances between *δ*−126 and *δ*−146 ppm. A ^29^Si NMR spectrum (Supporting Information, Figure S81) of the same sample showed a major intense broad unsymmetrically shaped signal at *δ* 0.9 ppm and a sharp resonance at *δ*−21.9 ppm. A ^1^H NMR spectrum (Supporting Information, Figure S79) revealed signals for the SiC*H*_3_, SiC*H*_2_Sn, SnC*H*_2_SiMe_3_, and Si(C*H*_3_)_3_ protons with correct integral ratio of 3:6:6:27. Attempts at obtaining ^1^H DOSY NMR spectrum failed as the sample became gel‐like over time. From the NMR data at hand, we conclude that the identity of **8** is not retained in solution. Apparently, the solution contains a mixture of different species. With caution and in analogy to **7**, we assign the three sharp ^119^Sn resonances (vide supra) to the oktokaideka‐nuclear diorganotin oxide [MeSi(CH_2_SnCH_2_SiMe_3_O)_3_]_6._ Either the latter is present right from the beginning in the isolated bulk crystalline material or it forms upon dissolution of this material.

An ESI MS (Supporting Information, Figures S85–S94) of a solution of the microcrystalline bulk material in CH_3_CN/CH_2_Cl_2_ revealed mass clusters centred at *m*/*z* 750.9293, *m*/*z* 1646,8168, *m*/*z* 2254.7523, *m*/*z* 3077.6608, and *m*/*z* 4506.3980. These are assigned to [MeSi(CH_2_SnCH_2_SiMe_3_O)_3_+H^+^]^+^, {[MeSi(CH_2_Sn(OH)_2_CH_2_SiMe_3_)_3_]_2_ + 2 H_2_O + H^+^}^+^, {[MeSi(CH_2_SnCH_2_SiMe_3_O)_3_]_6_ + 2 H^+^}^2+^, {[MeSi(CH_2_SnCH_2_SiMe_3_O)_3_]_4_+H^+^}^+^, and {[MeSi(CH_2_SnCH_2_SiMe_3_O)_3_]_6_+H^+^}^+^, respectively.

**Scheme 3:**
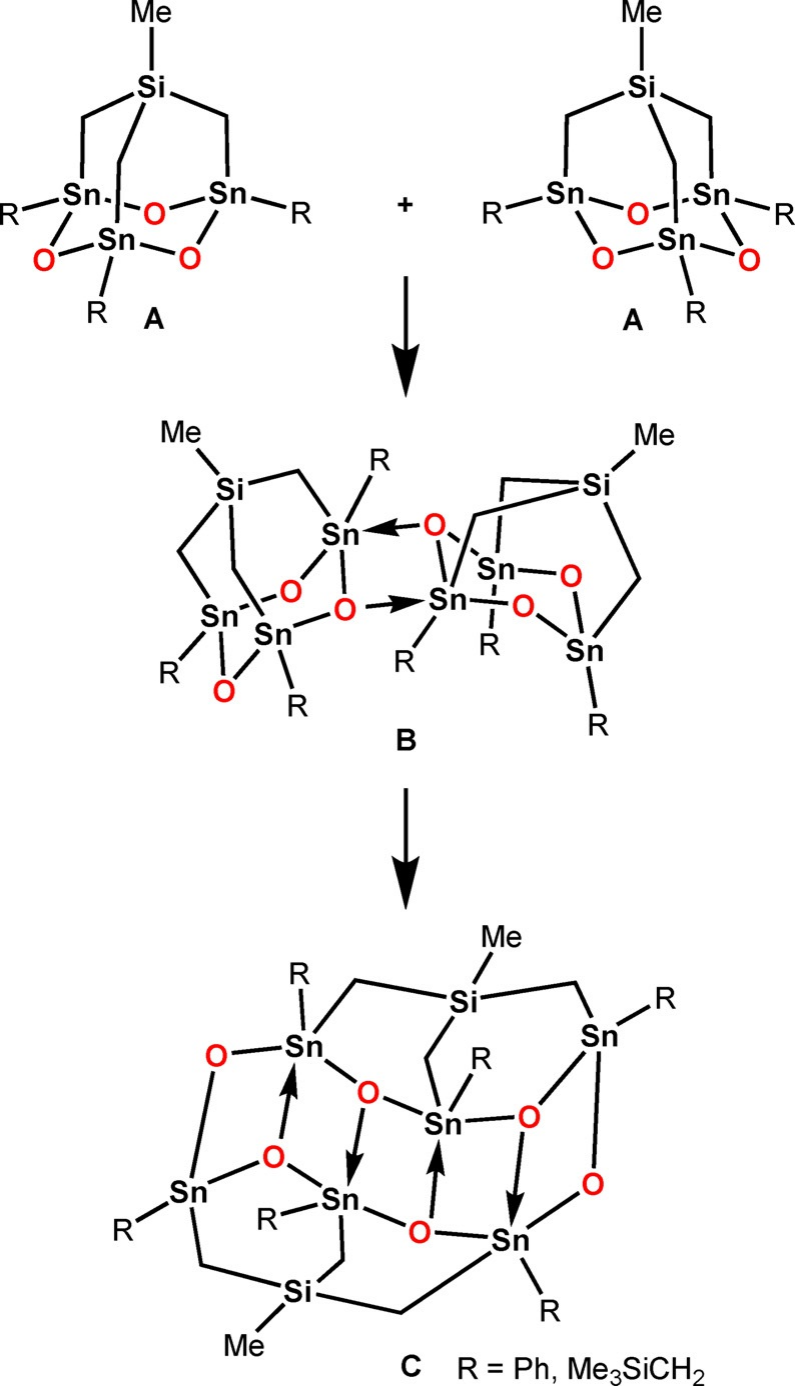
Association of two adamantane‐type diorganotin oxide moieties **A** undergoing subsequent ring‐opening dimerization giving **C**. The existence in solution of these moieties gets support from electrospray ionization mass spectrometry.

Although no detailed mechanistic studies have been performed, the formation of **7** and **8** can formally be seen as a stepwise process as shown in Scheme 3. Molecular diorganotin oxides **A** with adamantane‐type structure undergo ring‐opening dimerization via the intermediate **B** giving the hexanuclear product **C**. In case of R=Ph, three **C**‐moieties assemble giving the oktokaideka‐nuclear diorganotin oxide **7**.

In case of R=Me_3_SiCH_2_, however, **C**‐moieties combine with **A**‐ and **B**‐moieties giving, as one product out of probably several, the trikonta‐nuclear molecular diorganotin oxide **8**. This view gets support from the ESI mass spectrometric studies revealing mass clusters that are in line with the presence of **A**‐ and **C**‐moieties (vide supra).

## Conclusion

In conclusion we have shown that simple tripod‐type diorganotin halides such as MeSi(CH_2_SnRI_2_)_3_ (R=Ph, Me_3_SiCH_2_) serve as precursors for the synthesis of novel belt‐shaped molecular diorganotin oxides [MeSi(CH_2_SnRO)_3_]_*n*_ of unprecedented oktokaideka (*n*=18) and trikonta (*n*=30) nuclearity. The results obtained fit well into the ongoing interest in large‐sized metaloxido clusters in general^[2k]^ and tinoxido clusters of high nuclearity in particular.[^5g^, ^6b^, ^12^, ^31^, ^32^] The concept shown herein holds great potential for future work and we encourage interested readers to step into the field. Just to mention a few options out of many, the variation of the substituents R and/or replacing the CH_3_ group with other substituents, the variation of the spacing between the silicon and tin centres as well as replacement of the MeSi bridgehead moiety with MeGe or with isoelectronic P, P=E (E=O, S, Se) or PM (M=transition metal moiety such as W(CO)_5_ and others) might give a plethora of novel diorganotin oxides showing polynuclear structures. Moreover, replacing the organic substituent R in Roesky's (RSn)_4_O_6_ ( R=(Me_3_Si)_2_CH)^[3]^ by a R′ of slightly reduced steric bulk could give well defined oligomers [(R′Sn)_4_O_6_]_*n*_ similar to **7** and **8**.

## Conflict of interest

The authors declare no conflict of interest.

## Supporting information

As a service to our authors and readers, this journal provides supporting information supplied by the authors. Such materials are peer reviewed and may be re‐organized for online delivery, but are not copy‐edited or typeset. Technical support issues arising from supporting information (other than missing files) should be addressed to the authors.

Supplementary
