# Supplementary material for: MeSi(CH2SnRO)3 (R=Ph, Me3SiCH2): Building Blocks for Triangular‐Shaped Diorganotin Oxide Macrocycles
Source: Angew Chem Int Ed Engl. 2020 Oct 19;59(52):23892–8. doi: 10.1002/anie.202012248 (PMC7756359; doi:10.1002/anie.202012248)
Supplement: Supplementary file 1 — Supplementary [file ANIE-59-23892-s001.pdf]

Angewandte Chemie
Eine Zeitschrift der Gesellschaft Deutscher Chemiker

Supporting Information

$MeSi(CH_2SnRO)_3$ (R = Ph, Me_3SiCH_2): Building Blocks for Triangular-Shaped Diorganotin Oxide Macrocycles

Jihed Ayari, Christian R. Göb, Iris M. Oppel, Michael Lutter, Wolf Hiller, and Klaus Jurkschat**

anie_202012248_sm_miscellaneous_information.pdf

Table of Contents

- **General aspects.**
- **Synthesis procedures for compounds 1– 8.**
- **Analytical data for compounds 1– 8 and Figures showing different images of the structures of 7 and 8 in the solid state (including Tables S1-S4 containing interatomic distances and angles).**
- **Table S5: Crystallographic data for compounds 2, 7, and 8.**
- **References**

Experimental Procedures

General aspects.

All solvents were dried and purified according to standard procedures and freshly distilled prior to use. $MeSi(CH_2Cl)_3$, [1] $NaSnPh_3$, [2] and $(t\text{-}Bu_2SnO)_3$[3] were synthesized according to the literature. Ph_3SnCl, $MeSiCl_3$, elemental iodine, and silver chloride were commercially available. They were used without further purification. The ^{1}H, ^{13}C, ^{29}Si and ^{119}Sn NMR spectra were recorded on Bruker DPX-400, DRX-600, and AVIII-600 spectrometers. Solution ^{1}H, ^{13}C, ^{29}Si, and ^{119}Sn NMR chemical shifts δ are given in ppm and were referenced to Me_4Si (^{1}H, ^{13}C, ^{29}Si), and Me_4Sn (^{119}Sn). Elemental analyses were performed on a LECO-CHNS-932 analyzer. The electrospray mass spectra were recorded with a Thermoquest−Finnigan instrument. The samples were introduced as solution in CH_3CN or CH_2Cl_2 through a syringe pump operating at 0.5 μL min^{-1}. The capillary voltage was 4.5 kV, whereas the cone skimmer voltage was varied between 50 and 250 kV. Identification of the expected ions was assisted by comparison of experimental and calculated isotope distribution patterns. The m/z values reported correspond to those of the most intense peak in the corresponding isotope pattern. Melting points were determined using a Büchi Melting Point M-560. IR spectra (ATR) were recorded on a PerkinElmer FTIR spectrometer.

The DOSY (diffusion-ordered spectroscopy) measurement was performed with a pulse sequence using double stimulated echo for convection compensation and LED and bipolar gradient pulses for diffusion.[4] The measurements were executed with an AVANCE-III HD 600 MHz NMR spectrometer equipped with a 5 mm heliumcooled BBFO probe from Bruker BioSpin GmbH (Rheinstetten,Germany). Thirty-two different gradient strengths varying between 3% and 95% of the maximum strength of 53 G/cm were used. Thirty-two scans per gradient strength were acquired with 16 kB data points of the FID (acquisition time of 0.97 s) and a relaxation delay of 1.5 s. According to the DOSY figure, the expansion indicates one conformational rearrangement of the compound in solution with diffusion coefficient of $3.89 \cdot 10^{-10}(+/- 1 \cdot 10^{-11})$ m^2s^{-1}.

Single crystal X-ray diffraction data was collected with either an Oxford Diffraction Xcalibur, equipped with a Saphire3 CCD detector and an Enhance fine focus sealed tube (Mo-Kα), or a Stoe StadiVari, equipped with a Pilatus 200K HPC detector and a GeniX 3D microfocus sealed tube (Cu-Kα, Xenocs), by using ω-scans. Data collection, integration, absorption correction and space group determination were performed with the respective software packages CrysAlisPro (2014 and 2019) or X-Area (2018). Structure solutions were obtained with ShelXS (2008 and 2013) using the direct method. The structure models were refined by using ShelXL (2018) with a least squares procedure against F^2. Unresolved electron density was subtracted from the structure factor by applying a solvent mask in the software package OLEX2. The solvent mask for compound **7** was calculated to be 836 electrons in a volume of 3500 $Å^3$ in per unit cell. This is consistent with the presence of 24 molecules dichloromethane per unit cell, which account for 1008 electrons per unit cell. The solvent mask for compound **8** was calculated to be 165 electrons in a volume of 2220 $Å^3$ in per unit cell. This is consistent with the presence of 16 molecules dichloromethane per unit cell, which account for 672 electrons per unit cell. Compound **8** was measured using Cu-Kα radiation. With an absorption coefficient of 20.7 mm^{-1}, the observed amount of electrons is systematically underestimated. Hydrogen atoms were refined using riding models with $U_{eq}(H)$ of $1.5 \cdot Ueq(C)$ for terminal methyl groups, and $1.2 \cdot Ueq(C)$ for other groups.

CCDC numbers in Table S2 contain the supplementary crystallographic data for this publication. The data can be obtained free of charge from The Cambridge Crystallographic Data Centre via www.ccdc.cam.ac.uk/structures.

Synthesis procedures for compounds 2− 8

- **Synthesis of tris(triphenylstannylmethyl)methylsilane MeSi(CH_2SnPh_3)$_3$ (2)**

To a solution of $SnPh_3Cl$ (10 g, 25.94 mmol) in THF (250 mL) were added metallic sodium (1.43 g, 62.26 mmol, 2.4 equiv) and a catalytic amount of naphthalene. The mixture was stirred at room temperature for 3 days, during which its colour changed to deep black. Further activation to accelerate the process was realized by sonification with ultrasound (45 min). After the solution had been separated from non-reacted sodium, **1** (1.65 g, 8.62 mmol,) was added dropwise at −70 °C under magnetic stirring. Overnight, the reaction mixture was warmed to room temperature and the solvent was evaporated in vacuo. The residue obtained was extracted with 300 mL diethyl ether followed by washing with 150 mL distilled water in order to remove the sodium chloride. The organic phase was dried over anhydrous $MgSO_4$ and filtrated. The solvent was removed from the filtrate under reduced pressure, giving **2** as amorphous white solid (9.59 g, 8.54 mmol, 98% yield). Further purification was achieved by recrystallization from hot *iso*-hexane to give transparent needles with a mp of 132 °C.^{1}H NMR ($CDCl_3$, 400.25, 298 K): δ −0.19 (s, 3H, SiCH_3), 0.33 (s, 6H, $^2J(^1H-^{117/119}Sn)$ = 78 Hz, SiCH_2Sn), 7.30−7.44 (complex pattern, 45H, Ph). $^{13}C\{^1H\}$ NMR ($CDCl_3$, 150.94, 298 K): δ −1.7 ($^3J(^{13}C-^{117/119}Sn)$ = 20 Hz, $^1J(^{13}C-^{29}Si)$ = 48 Hz, $^1J(^{13}C-^{117/119}Sn)$ = 262/274 Hz, SiCH$_2$Sn), 3.9 ($^3J(^{13}C-^{117/119}Sn)$ = 12 Hz, $^1J(^{13}C-^{29}Si)$ = 51 Hz, SiCH$_3$), 128.4 ($^3J(^{13}C-^{117/119}Sn)$ = 49 Hz, C_m), 128.7 ($^4J(^{13}C-^{117/119}Sn)$ = 10 Hz, C_p), 136.9 (^2J $(^{13}C-^{117/119}Sn)$ = 37 Hz, C_o), 139.6 ($^1J(^{13}C-^{117/119}Sn)$ = 460/492 Hz, C_i). ^{29}Si NMR ($CDCl_3$, 79.52, 298 K): δ 8.7 ($^2J(^{29}Si-^{117/119}Sn)$ = 21 Hz, *Si*CH$_2$Sn). ^{119}Sn NMR ($CDCl_3$, 149.26, 298 K): δ −89 (*Sn*Ph$_3$). Anal. Calcd (%) for $C_{58}H_{54}SiSn_3$: C 61.36, H 4.79. Found: C 61.3, H 4.8. Electrospray MS: m/z (%) positive mode 119.1 (100, Sn^+), 383.0097 ($C_{18}H_{15}SnO_2$) (50, [M – $C_{40}H_{43}SiSn_2$ + 2 H_2O]).

- **Synthesis of tris(iodidodiphenylstannylmethyl)methylsilane MeSi(CH_2SnPh_2I)$_3$ (3)**

Over a period of 3h, elemental iodine (0.341 g, 1.34 mmol, 2.88 equiv) was added in small portions at 0 °C to a stirred solution of **2** (0.529 g, 465.97 µmol, 1 equiv) in dichloromethane. The stirring was continued and the reaction mixture was warmed to room temperature overnight. Dichloromethane and iodobenzene were removed in vacuo (10^{-3} mmHg) to afford a slightly yellow oil in 95% yield (0.502 g, 391.18 µmol). Several washings with *iso*-hexane realized further purification.^{1}H NMR ($CDCl_3$, 400.25, 298 K): δ 0.15 (s, 3H, SiCH_3), 0.99 (s, 6H, $^2J(^1H-^{117/119}Sn)$ = 80 Hz, SiCH_2Sn), 7.35−7.67 (complex pattern, 15H, Ph). $^{13}C\{^1H\}$ NMR ($CDCl_3$, 150.94, 298 K): δ 3.76 ($^3J(^{13}C-^{117/119}Sn)$ = 15 Hz, SiCH$_3$), 4.14 ($^3J(^{13}C-^{117/119}Sn)$ = 23 Hz, $^1J(^{13}C-^{29}Si)$ = 48 Hz, $^1J(^{13}C-^{117/119}Sn)$ = 253, 264 Hz, SiCH$_2$Sn), 128.8 ($^3J(^{13}C-^{117/119}Sn)$ = 60 Hz, C_m), 129.9 ($^4J(^{13}C-^{117/119}Sn)$ = 14 Hz, C_p), 135.8 (^2J $(^{13}C-^{117/119}Sn)$ = 50 Hz, C_o), 137.6 ($^1J(^{13}C-^{117/119}Sn)$ = 520/544 Hz, C_i). ^{29}Si NMR ($CDCl_3$, 119.26, 298 K): δ 8.97 ($^2J(^{29}Si-^{117/119}Sn)$ = 28 Hz, *Si*CH$_2$Sn). ^{119}Sn NMR ($CDCl_3$, 223.85, 298 K): δ −67 (*Sn*IPh$_2$). Anal. Calcd (%) for $C_{40}H_{33}I_3SiSn_3$: C 37.4, H 3.06. Found: C 38.3, H 3.4. Electrospray

MS: m/z (%) positive mode 919.2 $C_{39}H_{44}NaSiSn_3^+$ (100, [M – Me − 3I⁻ + 4 H⁺ + Na⁺]⁺), 969.2 $C_{12}H_{23}I_3O_2SiSn_3^+$ (100, [M – 5Ph⁻ + 6 H⁺ + Na⁺ + 2MeOH]⁺), m/z (%) negative mode 127.3 I⁻ (100, [M – $C_{40}H_{33}I_2SiSn_3^+$]⁻).

- **Synthesis of tris(diiodidophenylstannylmethyl)methylsilane MeSi(CH_2SnPhI_2)$_3$ (4)**

Over a period of 10h, elemental iodine (8.39 g, 33.06 mmol, 6 equiv) was added in small portions at 0 °C to a stirred solution of **2** (6.36 g, 5.60 mmol, 1 equiv) in CH_2Cl_2. The stirring was continued and the reaction mixture was warmed to room temperature overnight. Dichloromethane and iodobenzene were removed in vacuo (10^{-3} mmHg) to afford a yellow oil in 99% yield (7.95 g, 5.54 mmol).^{1}H NMR ($CDCl_3$, 400.25, 298 K): δ 0.53 (s, 3H, SiCH_3), 1.71 (s, 6H, ^2J(^{1}H−$^{117/119}$Sn) = 84 Hz, SiCH_2Sn), 7.43−7.73 (complex pattern, 15H, Ph). ^{13}C{^{1}H} NMR ($CDCl_3$, 150.94, 298 K): δ 3.23 (^3J(^{13}C−$^{117/119}$Sn) = 20 Hz, ^1J(^{13}C−^{29}Si) = 40 Hz, SiCH_3), 11.9 (^3J(^{13}C−$^{117/119}$Sn) = 20 Hz, ^1J(^{13}C−^{29}Si) = 50 Hz, ^1J(^{13}C−$^{117/119}$Sn) = 259/272 Hz, SiCH_2Sn), 129.2 (^3J(^{13}C−$^{117/119}$Sn) = 78 Hz, C_m), 131.1 (^4J(^{13}C−$^{117/119}$Sn) = 16 Hz, C_p), 134.1 (^2J (^{13}C−$^{117/119}$Sn) = 59 Hz, C_o), 136.5 (^1J(^{13}C−$^{117/119}$Sn) = 580/601 Hz, C_i). ^{29}Si NMR ($CDCl_3$, 119.26, 298 K): δ 8.8 (^2J(^{29}Si−$^{117/119}$Sn) = 36 Hz, *Si*CH_2Sn). ^{119}Sn NMR ($CDCl_3$, 223.85, 298 K): δ −229 (*Sn*I_2Ph). Anal. Calcd (%) for $C_{22}H_{24}I_6SiSn_3$: C 18.43, H 1.69. Found: C 18.8, H 1.9. Electrospray MS: m/z (%) positive mode 392.1 $I_2SnH_3O^+$ (30, [M − $C_{22}H_{24}I_4SiSn_2$ + H⁺ + H_2O]⁺), 721.0 $C_{16}H_{19}ISiSn_3^+$ (15, [M – $C_6H_5I_5$]⁺), m/z (%) negative mode 127.3 I⁻ (8, [M − $C_{22}H_{24}I_5SiSn_3$]⁻), 381.0 I_3^- (100, [M − $C_{22}H_{24}I_3SiSn_3$]⁻), 1450.3017 ($C_{22}H_{25}I_6OSiSn_3$*1.00 [M + OH⁻]⁻ + $C_{22}H_{24}I_6ClSiSn_3$*0.10 [M + Cl⁻]⁻), 1560.1988 $C_{22}H_{24}I_7OSiSn_3^-$ ([M + I]⁻).

- **Synthesis of tris[diphenyl(trimethylsilylmethyl)stannylmethyl)methylsilane MeSi[$CH_2Sn(CH_2SiMe_3)Ph_2$]$_3$ (5)**

A solution of MeSi(CH_2SnPh_2I)$_3$, **2** (4.77g, 3.56 mmol, 0.9 equiv) in THF (120 mL) was added dropwise to a solution of Me_3SiCH_2MgCl, prepared from Me_3SiCH_2Cl (1.46 g, 11.87 mmol, 3 equiv) and magnesium (0.307 g, 12.66 mmol, 3.2 equiv) in THF (40 mL), for a period of 1h. After the addition had been completed, the reaction mixture was heated to reflux overnight and then cooled to room temperature. THF was distilled off under reduced pressure; then cold water (50 mL) was added, and the mixture was extracted three times with 100 mL diethyl ether. The combined organic phases were dried over $MgSO_4$ and the solvents removed under reduced pressure, giving **5** as a slightly yellow oil (4.341 g, 3.73 mmol, 94 %). Further purification was achieved by several wash with iso-hexane. ^{1}H NMR ($CDCl_3$, 600.29, 298 K): δ −0.24 (s, 3H, SiCH_3), −0.14 (s, 27H, Si(CH_3)$_3$), 0.04 (s, 6H, CH_2SiMe$_3$), 0.10 (s, 6H, ^2J(^{1}H−$^{117/119}$Sn) = 72/74 Hz, SiCH_2Sn), 7.30−7.41 (complex pattern, 30H, Ph). ^{13}C{^{1}H} NMR ($CDCl_3$, 100.46, 298 K): δ −3.33 (^1J(^{13}C−$^{117/119}$Sn) = 255/267 Hz, CH_2SiMe$_3$), −0.06 (^3J(^{13}C−$^{117/119}$Sn) = 21 Hz, ^1J(^{13}C−$^{117/119}$Sn) = 248/266 Hz, SiCH_2Sn), 1.53 (^3J(^{13}C−$^{117/119}$Sn) = 14 Hz, ^1J(^{13}C−^{29}Si) = 51 Hz , Si(CH_3)$_3$), 3.85 (^3J(^{13}C−$^{117/119}$Sn) = 12 Hz, SiCH_3), 128.1 (C_m), 128.4 (C_p), 136.7 (^2J (^{13}C−$^{117/119}$Sn) = 38 Hz, C_o), 141.4 (^1J(^{13}C−$^{117/119}$Sn) = 452/477 Hz, C_i). ^{29}Si NMR ($CDCl_3$, 79.52, 298 K): δ 7.84 (^2J(^{29}Si−$^{117/119}$Sn) = 26 Hz, *Si*CH_3), 2.68 (^1J(^{29}Si−^{13}C) = 51 Hz, CH_2*Si*Me$_3$). ^{119}Sn NMR ($CDCl_3$, 223.85, 298 K): δ −49 (*Sn*$CH_2SiMe_3Ph_2$). Anal. Calcd (%) for $C_{52}H_{72}Si_4Sn_3$: C 53.58, H 6.23. Found: C 54.2, H 6.2. Electrospray MS: m/z (%) positive mode 1129.3 (10, $C_{43}H_{69}Cl_2O_2Si_3Sn_3^+$).

- **Synthesis of tris[diiodido(trimethylsilylmethyl)stannylmethyl)methylsilane MeSi[$CH_2Sn(CH_2SiMe_3)I_2$]$_3$ (6)**

Over a period of 3h, elemental iodine (0.769 g, 3.03 mmol, 6 equiv) was added in small portions at 0 °C to a stirred solution of **5** (0.589 g, 505.31 μmol, 1 equiv) in CH_2Cl_2. The stirring was continued and the reaction mixture was warmed to room temperature overnight. Dichloromethane and iodobenzene were removed in vacuo (10^{-3} mmHg) to afford a yellow solid in 99% yield (0.732 g, 500.25

µmol). ^{1}H NMR (CDCl$_3$, 400.25, 298 K): *δ* 0.16−0.23 (s, 27H, Si(C*H*$_3$)$_3$), 0.67 (s, 3H, SiC*H*$_3$), 1.45 (s, 6H, $^{2}J(^{1}$H−$^{117/119}$Sn) = 88/92 Hz, C*H*$_2$SiMe$_3$), 1.72 (s, 6H, $^{2}J(^{1}$H−$^{117/119}$Sn) = Hz, SiC**H**$_2$Sn). ^{13}C{^{1}H} NMR (CDCl$_3$, 150.94, 298 K): *δ* 1.74−1.34 ($^{3}J(^{13}$C−$^{117/119}$Sn) = 26 Hz, $^{1}J(^{13}$C−^{29}Si) = 52 Hz, Si(*C*H$_3$)$_3$), 3.13 ($^{3}J(^{13}$C−$^{117/119}$Sn) = 18 Hz, Si*C*H$_3$), 12.60 ($^{1}J(^{13}$C−$^{117/119}$Sn) = 242/254 Hz, $^{1}J(^{13}$C−^{29}Si) = 49 Hz, ($^{3}J(^{13}$C−$^{117/119}$Sn) = 24 Hz, Si*C*H$_2$Sn), 14.74 ($^{1}J(^{13}$C−^{29}Si) = 43 Hz, $^{1}J(^{13}$C−$^{117/119}$Sn) = 251/264 Hz, *C*H$_2$SiMe$_3$). ^{29}Si NMR (CDCl$_3$, 79.52, 298 K): *δ* 3.81 ($^{2}J(^{29}$Si−$^{117/119}$Sn) = 39 Hz, $^{1}J(^{29}$Si−^{13}C) = 53 Hz, CH$_2$*Si*Me$_3$), 9.52 ($^{2}J(^{29}$Si−$^{117/119}$Sn) = 44 Hz, *Si*Me). ^{119}Sn NMR (CDCl$_3$, 149.26, 298 K): *δ* −190 (*Sn*CH$_2$SiMe$_3$I$_2$). Anal. Calcd (%) for $C_{16}H_{42}I_6Si_4Sn_3$: C 13.12, H 2.89. Found: C 13.1, H 2.9. Electrospray MS: m/z (%) positive mode 824.9353 $C_4H_{12}I_3SiSn_3^+$ (100, [M – (CH$_2$SiMe$_3$I)$_3$ + H$^+$]$^+$).

- **Synthesis of [MeSi(CH$_2$SnPhO)$_3$]$_6$ (7)**

To a solution of **4** (888.00 mg, 0.581 mmol, 1 equiv) in 30 mL of CH_2Cl_2 was added freshly synthesised *t*-Bu$_2$SnO (434.45 mg, 1.75 mmol, 3 equiv). The resulting mixture was stirred at room temperature overnight. Chloroform was removed in vacuo (10^{-3} mmHg) and *t*-Bu$_2$SnI$_2$ was washed out successively with *iso*-hexane. A white residue was obtained (433.54 mg, 98 %). Recrystallization of the latter from CH_2Cl_2/ diethyl ether gave transparent crystals suitable for X-ray diffraction analysis. The crystals did not melt up to 400 °C. ^{1}H NMR (CDCl$_3$, 500.08, 298 K): δ −0.06 ($^{2}J(^{1}$H-$^{117/119}$Sn) 50 Hz, $^{2}J(^{1}$H-^{1}H) 10 Hz)/0.48 ($^{2}J(^{1}$H-$^{117/119}$Sn) 70 Hz, $^{2}J(^{1}$H-^{1}H) 10 Hz), 0.88 ($^{2}J(^{1}$H-$^{117/119}$Sn) 85 Hz, $^{2}J(^{1}$H-^{1}H) 15 Hz)/1.20 ($^{2}J(^{1}$H-$^{117/119}$Sn) not measured, $^{2}J(^{1}$H-^{1}H) 14.7 Hz), and 1.28 ppm ($^{2}J(^{1}$H-$^{117/119}$Sn) not measured, $^{2}J(^{1}$H-^{1}H) 10 Hz)/1.91 ($^{2}J(^{1}$H-$^{117/119}$Sn) 120 Hz, $^{2}J(^{1}$H-^{1}H) 10 Hz), 0.3, 1.42 (s, 18H, SiC*H*$_3$), 6.65−7.72 (complex pattern, 90H, Ph). ^{13}C{^{1}H} NMR (CDCl$_3$, 125.75, 298 K): *δ* 5.3, 29.1 (SiCH$_3$), 7.5 ($^{1}J(^{13}$C−^{29}Si) = 80 Hz), 13.6, 14.6 (Si*C*H$_2$Sn)), 127.5, 128.1, 128.4 (C_m), 129.4, 129.5 (we didn't recognised the third signal) (C_p), 135.3, 135.4, 135.7 (C_o), 143.1, 143.9, 144.3 (C_i) we didn't recognised the $^{117/119}$Sn satellites fault of bad resolution of the spectrum. ^{29}Si NMR no signal could be detected even with long measurement.^{119}Sn NMR (CDCl$_3$, 149.26, 298 K): *δ* −204 $^{2}J(^{117/119}$Sn−^{29}Si) = 59 Hz), ($^{2}J(^{119}$Sn−^{117}Sn) = 180 Hz, $^{2}J(^{119}$Sn−$^{117/119}$Sn) = 315 Hz, −225 ($^{2}J(^{119}$Sn−$^{117/119}$Sn) = 315 Hz), −228 ($^{2}J(^{119}$Sn−^{117}Sn) = 180 Hz) (*Sn*Ph). Anal. Calcd (%) for $C_{132}H_{144}O_{18}Si_6Sn_{18}$: C 36.67 H 3.36 Found: C 35.9, H 4.4. The sample used for the elemental analysis was kept under vaccum (0.1 mm Hg) for one hour to remove any residual solvent. Electrospray MS: m/z (%) positive mode:1442.7312 $C_{44}H_{49}O_6Si_2Sn_6^+$ (100, [MeSi(CH$_2$SnPhO)$_3$]$_2$ + H$^+$)$^+$, 2161.5910 $C_{66}H_{73}O_9Si_3Sn_9^+$ (0.7, [MeSi(CH$_2$SnPhO)$_3$]$_3$ + H$^+$)$^+$, 3636.3600 $C_{11}H_{125}O_{16}Si_5Sn_{15}^+$ (2, [MeSi(CH$_2$SnPhO)$_3$]$_3$ + MeOH + H$^+$)$^+$, 4324.1823 $C_{132}H_{145}O_{18}Si_6Sn_{18}^+$ ([MeSi(CH$_2$SnPhO)$_3$]$_6$ + H$^+$)$^+$. IR (cm^{-1}): ν(Sn−O−Sn) 695−726; ν(Sn−C) 494−559.

- **Synthesis of [MeSi(CH$_2$SnCH$_2$SiMe$_3$O)$_3$]$_{10}$ (8)**

Over a period of 1h, a solution of NaOH in water (20mL) (83.25mg, 2.08 mmol, 6 equiv) was added to a solution of **6** (508.00 mg, 346.90 µmol, 1 equiv) in 50 mL of a mixture of CH_2Cl_2 and methanol at 0°C. The resulting mixture was stirred at room temperature overnight. All solvents were removed in vacuo (10^{-3} mmHg) and NaI was washed out successively with water. An oily residue (255.29 mg, 98 %) was obtained showing good solubility in $CHCl_3$ and CH_2Cl_2. Crystallization from CH_2Cl_2/ diethyl ether gave transparent crystals suitable for X-ray diffraction analysis, mp 347 °C. ^{1}H NMR (CDCl$_3$, 600.29, 298 K): *δ* 0.04−0.33 (complex pattern, 270H, CH$_2$Si(C*H*$_3$)$_3$), 0.85−0.89 (complex pattern, 30H, Si C*H*$_3$), 1.26 (s, 60H, SiC*H*$_2$Me$_3$), 1.59 (s, 60H, SiC*H*$_2$Sn). ^{13}C{^{1}H} NMR (CDCl$_3$, 150.94, 298 K): *δ* 1.02−2.67 (SiCH$_3$), 14.12, 22.69, 29.36, 31,12 (SiCH$_2$Sn), 29.69 (*C*H$_2$SiMe$_3$). Even with long data acquisition, no $^{117/119}$Sn satellites were obtained .^{29}Si NMR (CDCl$_3$, 119.26, 298 K): *δ* −21 (*Si*Me), −0.8−1.6 (CH$_2$*Si*Me$_3$). ^{119}Sn NMR (CDCl$_3$, 223.85, 298 K): *δ* −163−126 (*Sn*CH$_2$SiMe$_3$). Anal. Calcd (%) for $C_{160}H_{420}O_{30}Si_{40}Sn_{30}$: C 25.59 H 5.64 Found: C 25.6, H 5.7. The sample used for the elemental analysis was kept under vaccum (0.1 mm Hg) for one hour to remove any residual solvent. Electrospray MS: m/z (%) positive mode: 750.9293 $C_{16}H_{43}O_3Si_4Sn_3^+$

(2.5, $[MeSi(CH_2SnCH_2SiMe_3O)_3] + H^+)^+$, 1646.8168 $C_{35}H_{93}Cl_2NO_7Si_8Sn_6^{2+}$: (0.5, $[MeSi(CH_2SnCH_2SiMe_3O)_3]_2 + CH_2Cl_2 + CH_3CN + H_2O + H^+)^+$, 2254.7532 $C_{96}H_{254}O_{18}Si_{24}Sn_{18}^{2+}$: (0.1, $[MeSi(CH_2SnCH_2SiMe_3O)_3]_3$*2 + $2H^+)^{2+}$, 4506.3980 $C_{96}H_{253}O_{18}Si_{24}Sn_{18}^+$ (4, $[MeSi(CH_2SnCH_2SiMe_3O)_3]_6 + H^+)^+$. IR ($cm^{-1}$): ν(Sn−O−Sn) 652−713; ν(Sn−C) 423−550.

Results and Discussion

Discussion of the 1H NMR spectra of compound **7**:

A ^{1}H NMR spectrum ($CDCl_3$ solution, Figures S56, 57) shows two resonances at δ 0.30 (integral 2) and 1.42 (integral 1) ppm, respectively, that are assigned to non-equivalent $SiCH_3$ protons. The non-equivalence of the $SiCH_3$ protons (ratio 2:1) becomes visible when looking perpendicular through the plane defined by the belt (Supporting Information, Figure S54). The $SiCH_2$ protons appear as three equally intense AX-type resonances at δ –0.06 ($^{2}J(^{1}H{-}^{117/119}Sn)$ 50 Hz, $^{2}J(^{1}H{-}^{1}H)$ 10 Hz)/0.48 ($^{2}J(^{1}H{-}^{117/119}Sn)$ 70 Hz, $^{2}J(^{1}H{-}^{1}H)$ 10 Hz), 0.88 ($^{2}J(^{1}H{-}^{117/119}Sn)$ 85 Hz, $^{2}J(^{1}H{-}^{1}H)$ 15 Hz)/1.20 ($^{2}J(^{1}H{-}^{117/119}Sn)$ not measured, $^{2}J(^{1}H{-}^{1}H)$ 15 Hz), and 1.28 ppm ($^{2}J(^{1}H{-}^{117/119}Sn)$ not measured, $^{2}J(^{1}H{-}^{1}H)$ 10 Hz)/1.91 ($^{2}J(^{1}H{-}^{117/119}Sn)$ 120 Hz, $^{2}J(^{1}H{-}^{1}H)$ 10 Hz), respectively. 2D NMR spectra unambiguously support the assignment of the ^{1}H resonances (see Supporting Information, Figures S61−S64).

Analytical data for compounds 1– 8

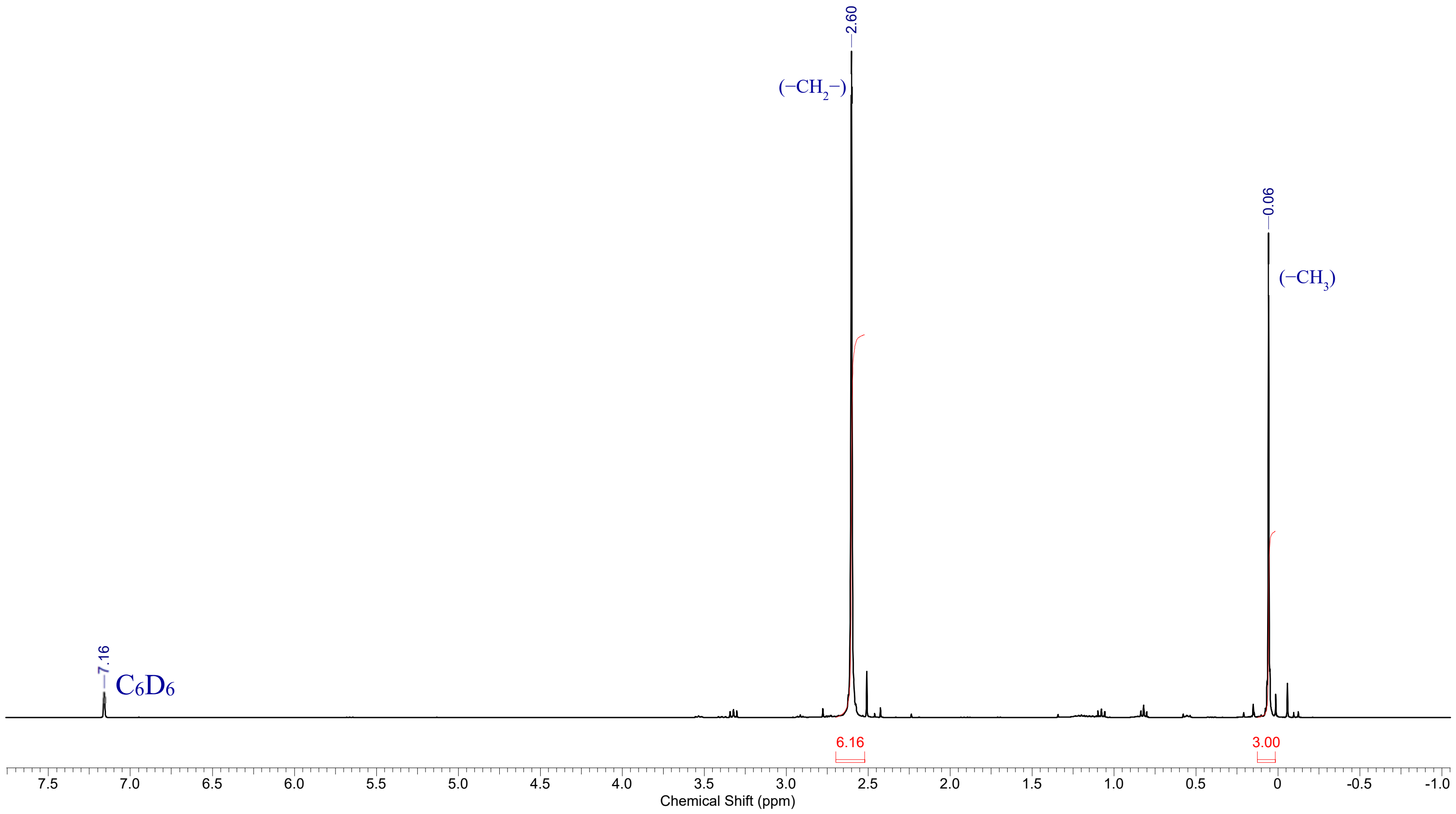

Figure S1. ^{1}H NMR spectrum (400.25 MHz, C_6D_6) of compound **1**.

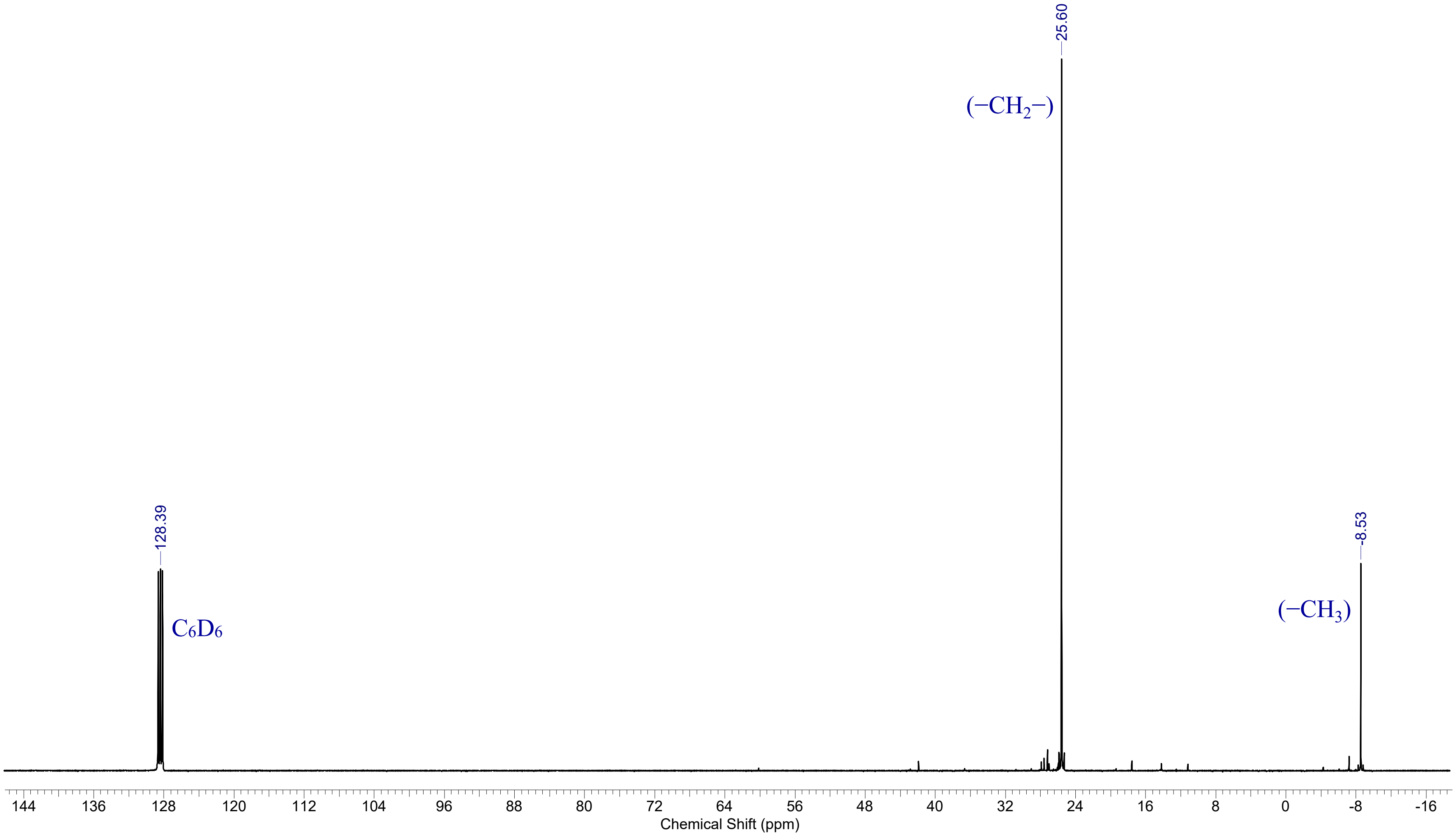

Figure S2. ^{13}C NMR spectrum (100.64 MHz, C_6D_6) of compound **1**.

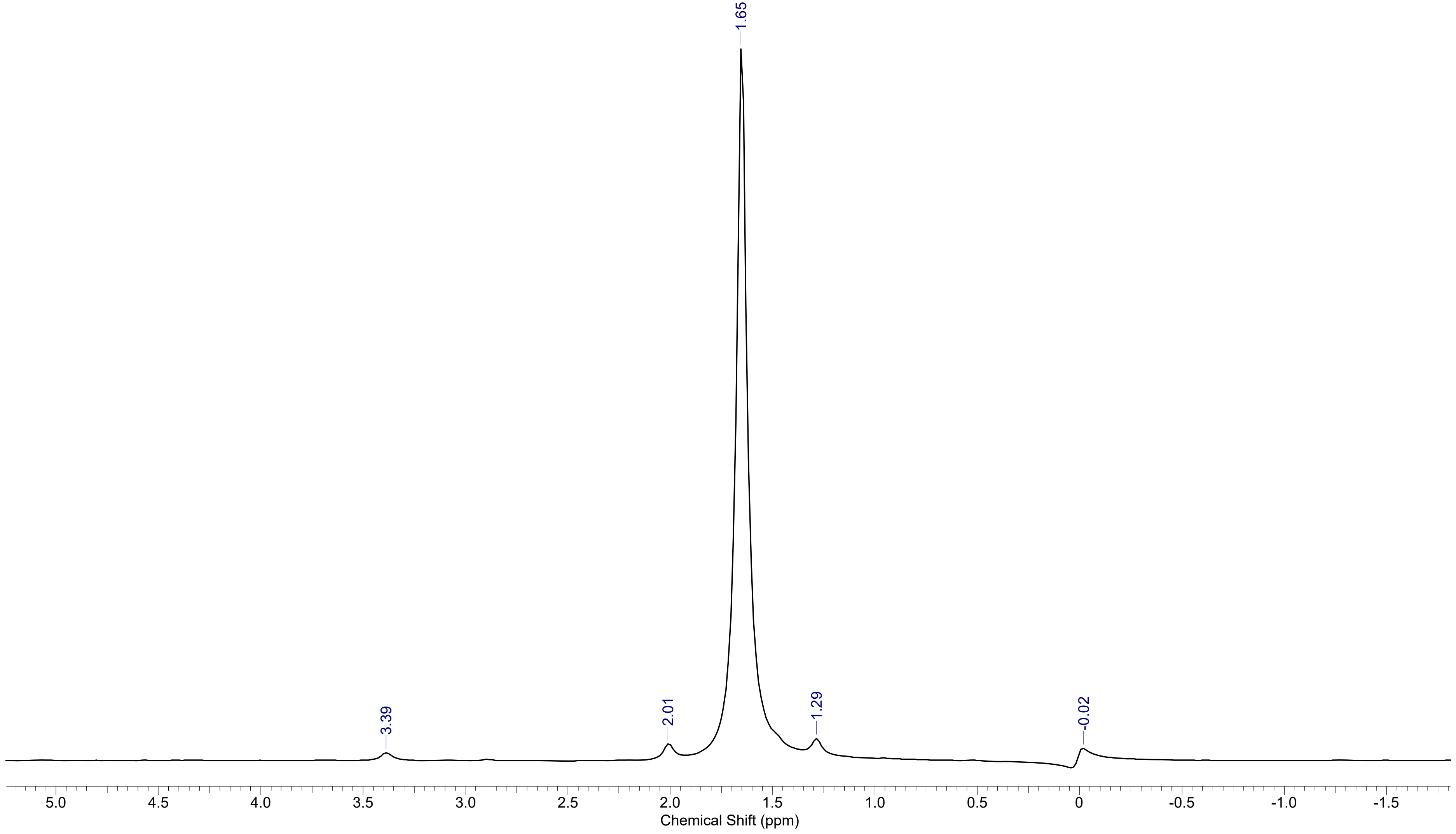

Figure S3. ^{29}Si NMR spectrum (79.52 MHz, C_6D_6) of compound **1**.

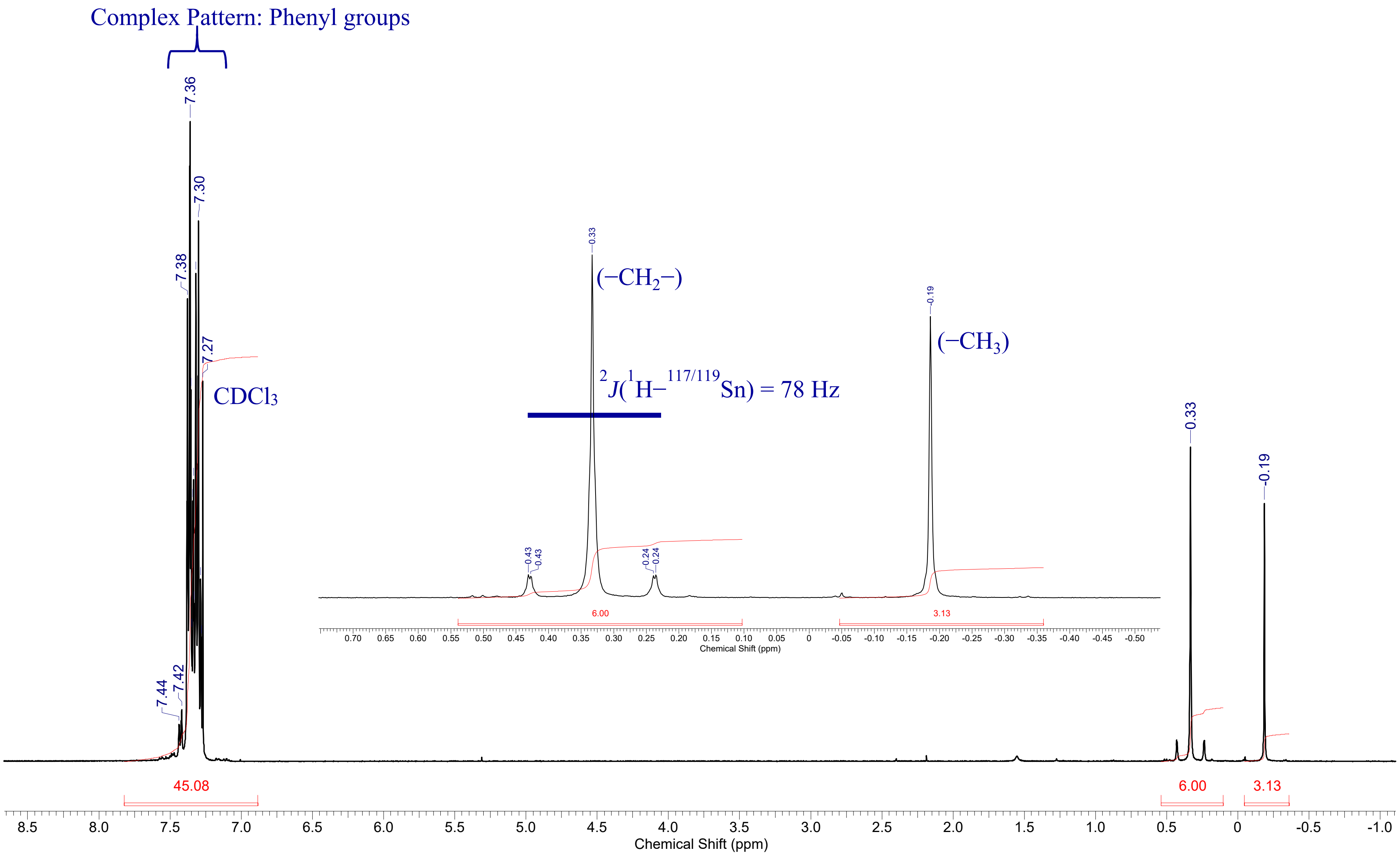

Figure S4. ^{1}H NMR spectrum (400.25 MHz, $CDCl_3$) of compound **2**.

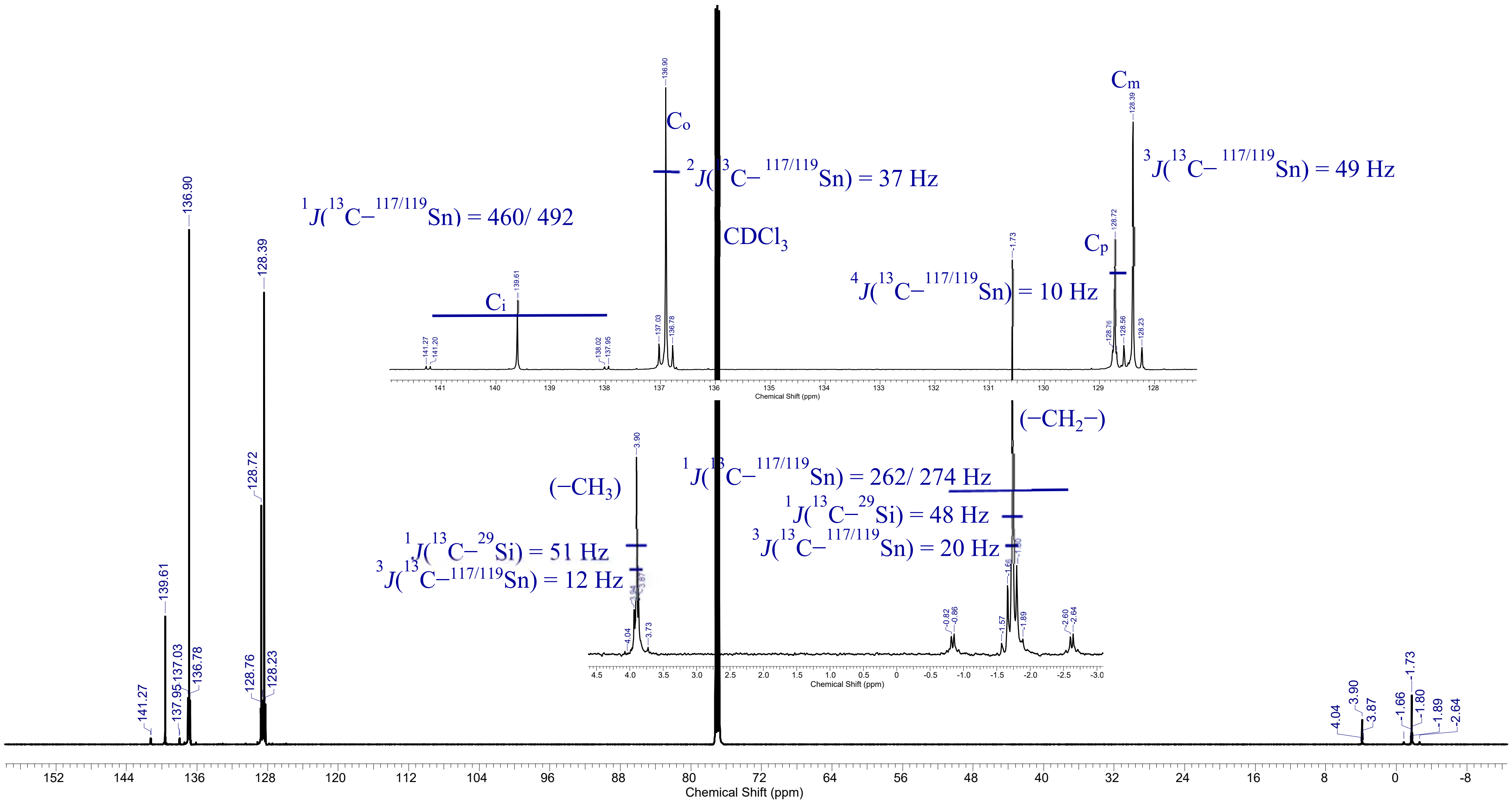

Figure S5. ^{13}C NMR spectrum (150.94 MHz, $CDCl_3$) of compound **2**.

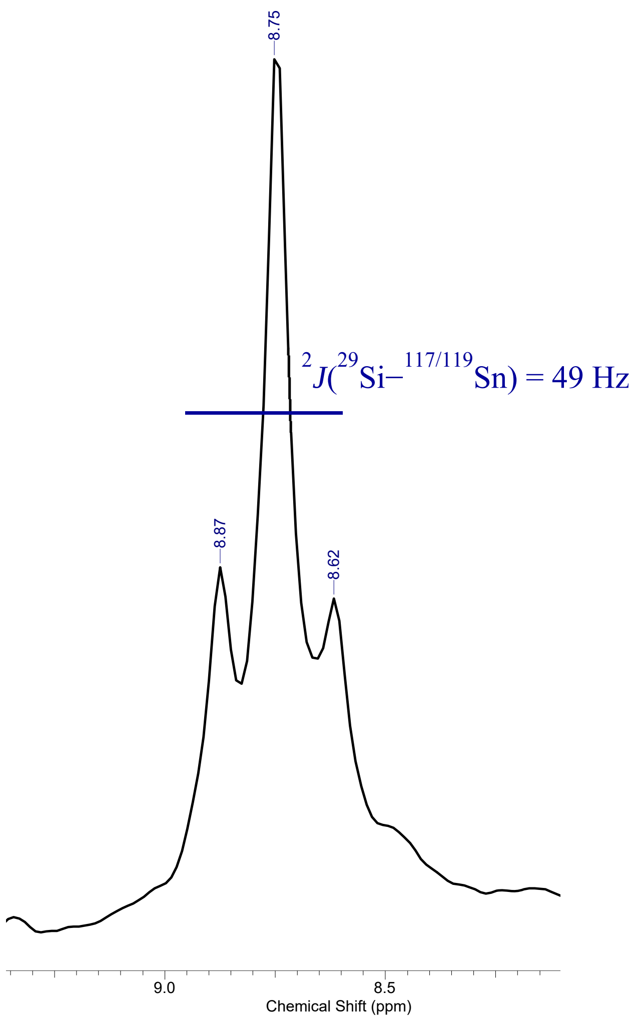

Figure S6. ^{29}Si NMR spectrum (149.26 MHz, $CDCl_3$) of compound **2**.

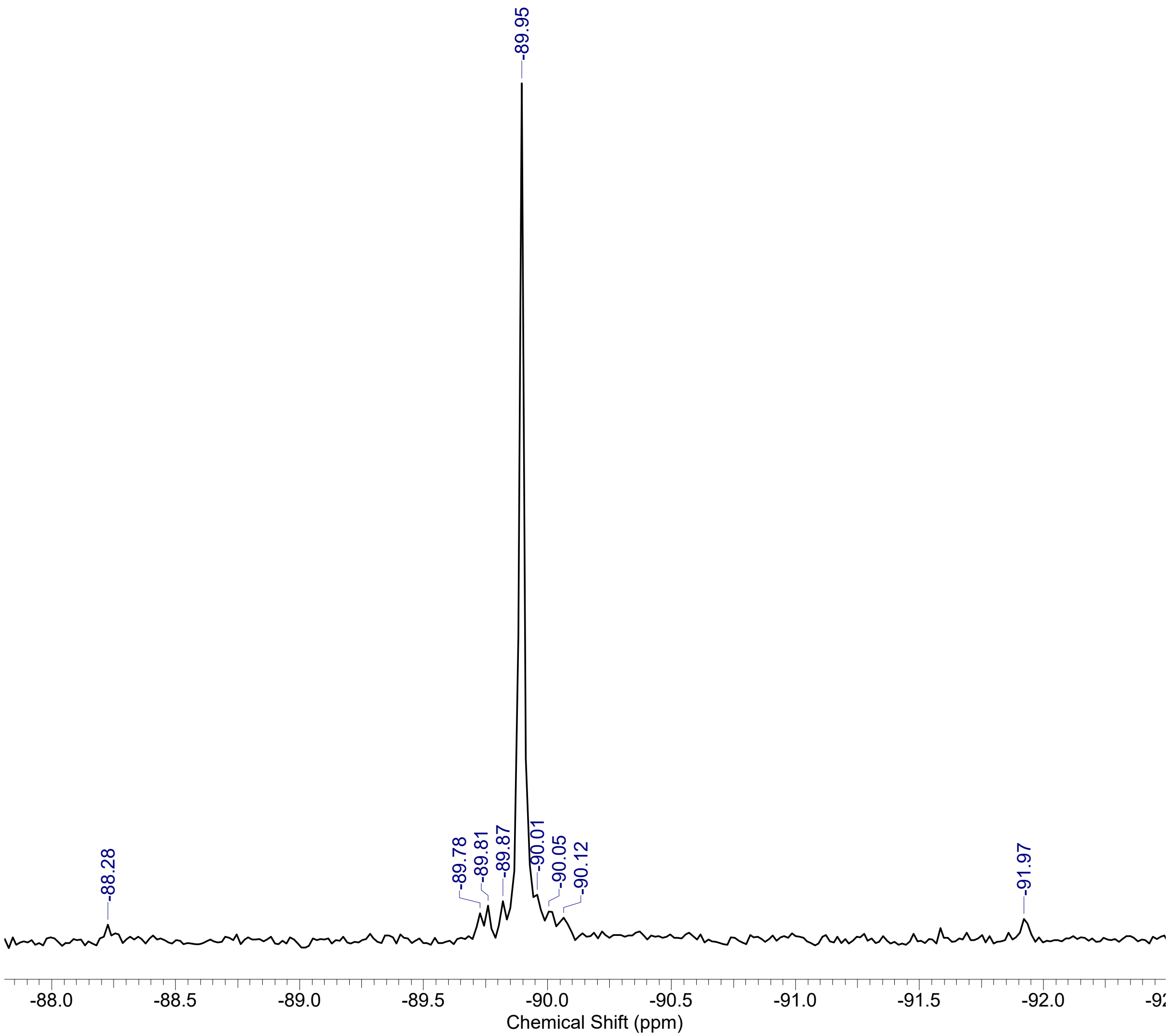

Figure S7. ^{119}Sn NMR spectrum (149.26 MHz, $CDCl_3$) of compound **2.**

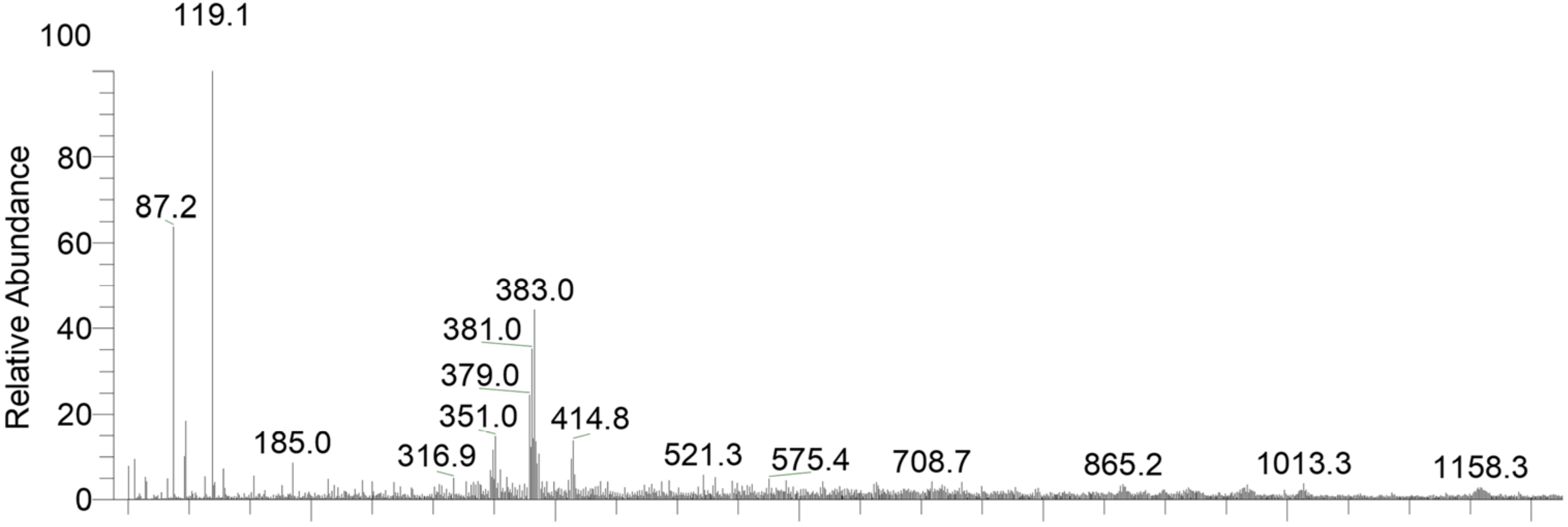

Figure S8. ESI-MS spectrum (positive mode) of **2**. The horizontal axis shows the m/z values.

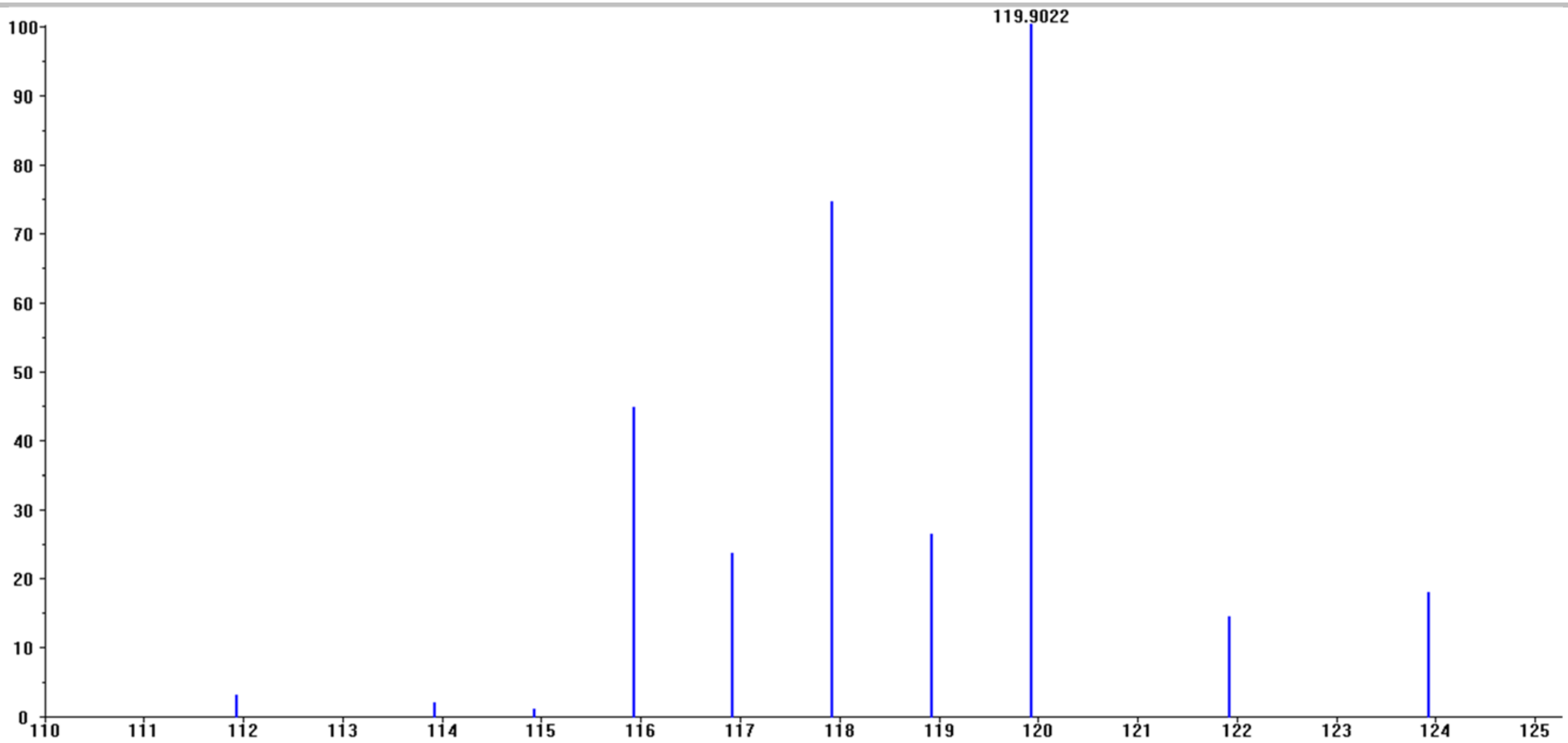

Figure S9. Simulated mass cluster of Sn^{+}. The horizontal axis shows the m/z values.

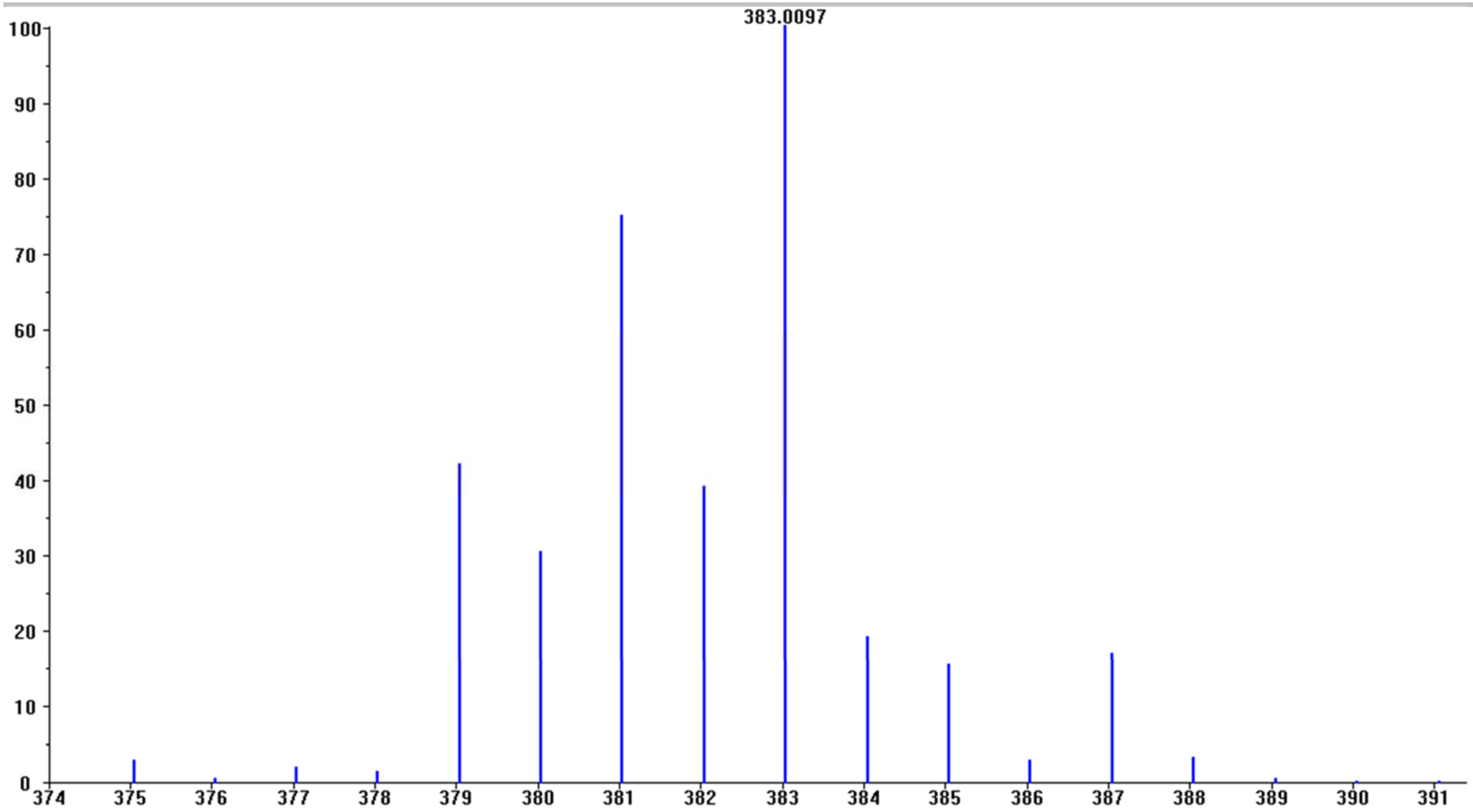

Figure S10. Simulated mass cluster of $C_{18}H_{15}SnO_2$. The horizontal axis shows the m/z values.

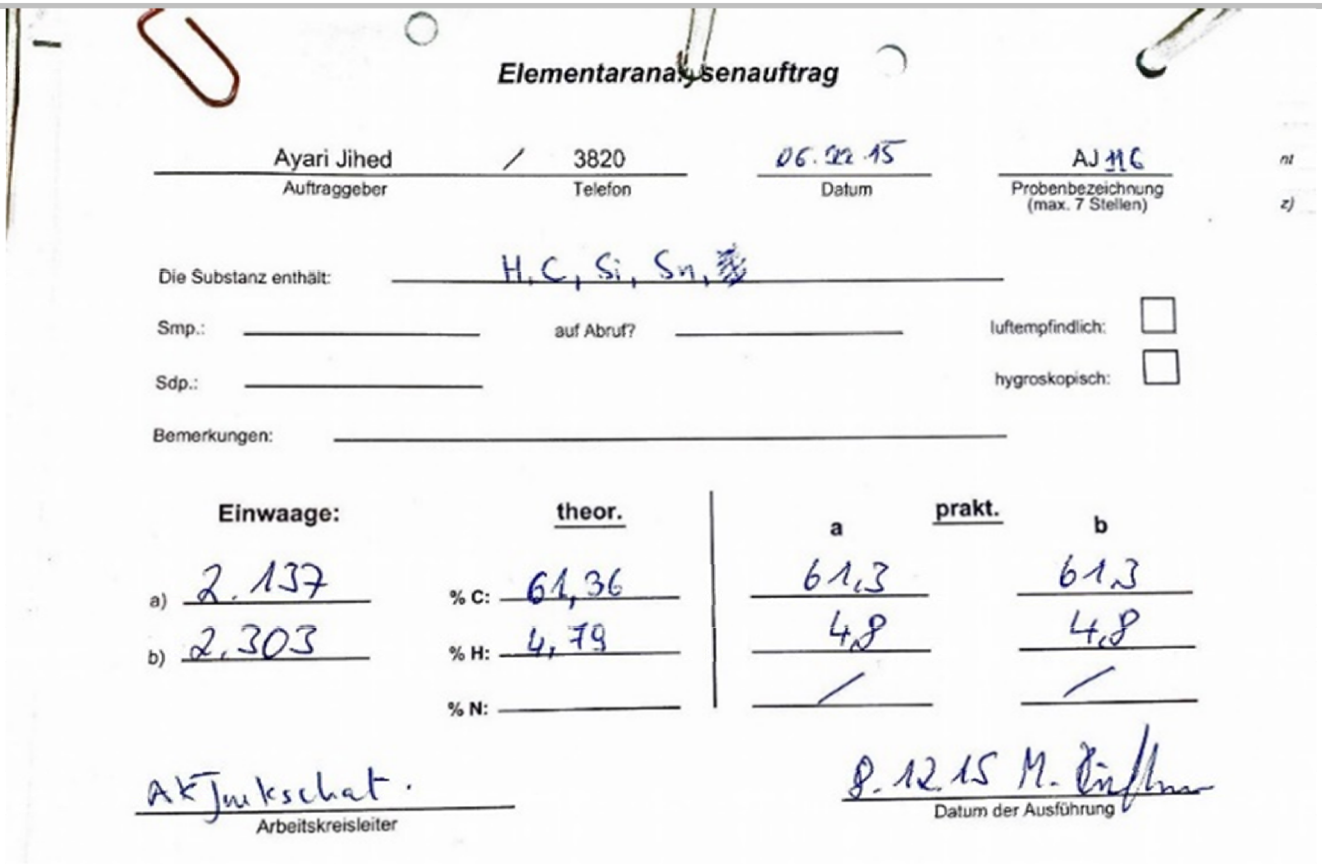

Elementaranalysenauftrag

Ayari Jihed / 3820	06.12.15	AJ 116
Auftraggeber / Telefon	Datum	Probenbezeichnung (max. 7 Stellen)

Die Substanz enthält: H, C, Si, Sn, [illegible]

Smp.: ______ auf Abruf? ______ luftempfindlich: ☐

Sdp.: ______ hygroskopisch: ☐

Bemerkungen: ______

Einwaage:		theor.	prakt. a	prakt. b
a) 2.137	% C:	61,36	61,3	61,3
b) 2,303	% H:	4,79	4,8	4,8
	% N:		/	/

AK Jurkschat.
Arbeitskreisleiter

8.12.15 M. [illegible]
Datum der Ausführung

Figure S11. Elemental analysis of **2**.

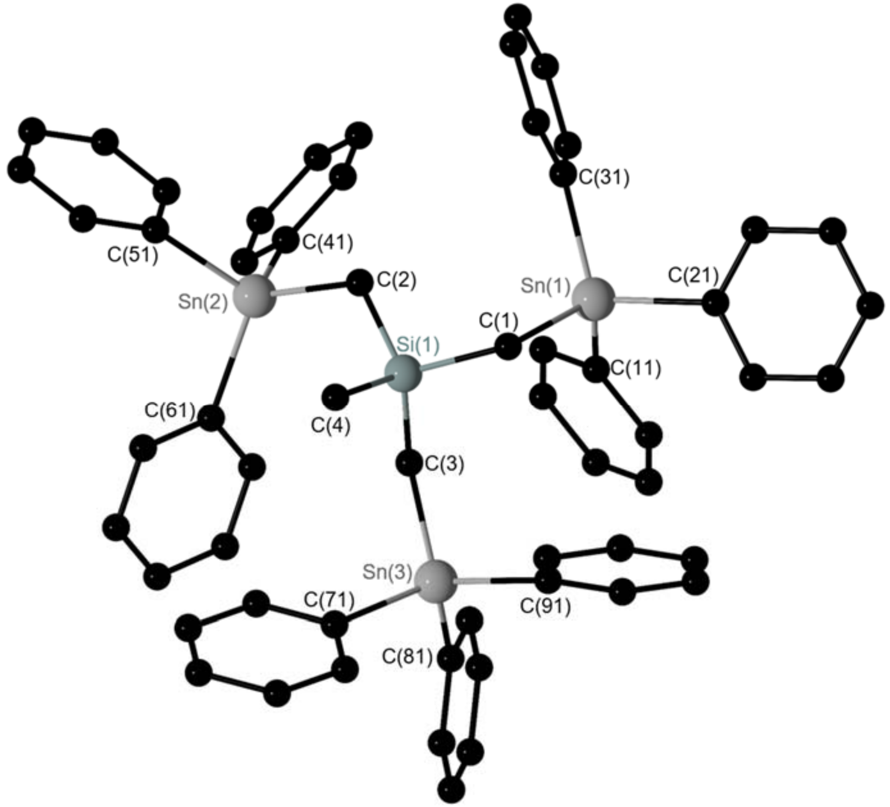

Figure S12. POV-Ray image of the molecular structure of $MeSi(CH_2SnPh_3)_3$, **2**. Selected interatomic distances (Å): Sn(2)−C(51) 2.120(7), Sn(2)−C(61) 2.1687(14), Si(1)−C(1) 1.867(3), Si(1)−C(2) 1.871(3), Si(1)−C(3) 1.866(3), Si(1)−C(4) 1.860(3), Sn(1)−C(1) 2.154(3), Sn(1)−C(2) 2.147(3), Sn(1)−C(3) 2.132(3). Selected interatomic angles (°): C(2)−Sn(2)−C(61) 118.66(10), C(3)−Sn(3)−C(81) 105.13(9), (C(3)−Si(1)−C(2) 110.52(14), C(4)−Si(1)−C(2) 107.96(17), Si(1)−C(1)−Sn(1) 117.40(14), Si(1)−C(2)−Sn(2) 120.29(15), Si(1)−C(3)− Sn(3) 120.52(14).

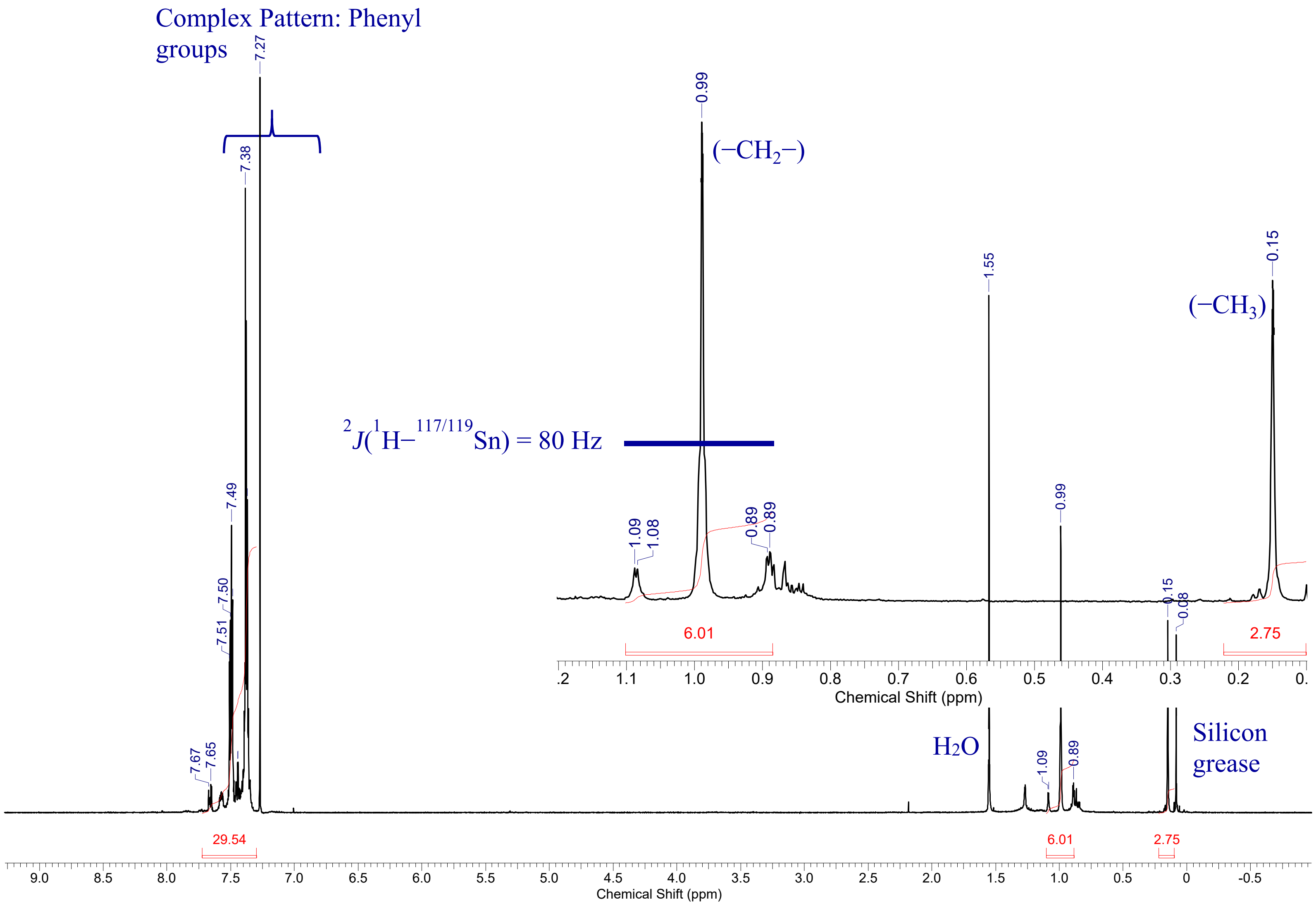

Figure S13. ^{1}H NMR spectrum (400.25 MHz, $CDCl_3$) of compound **3**.

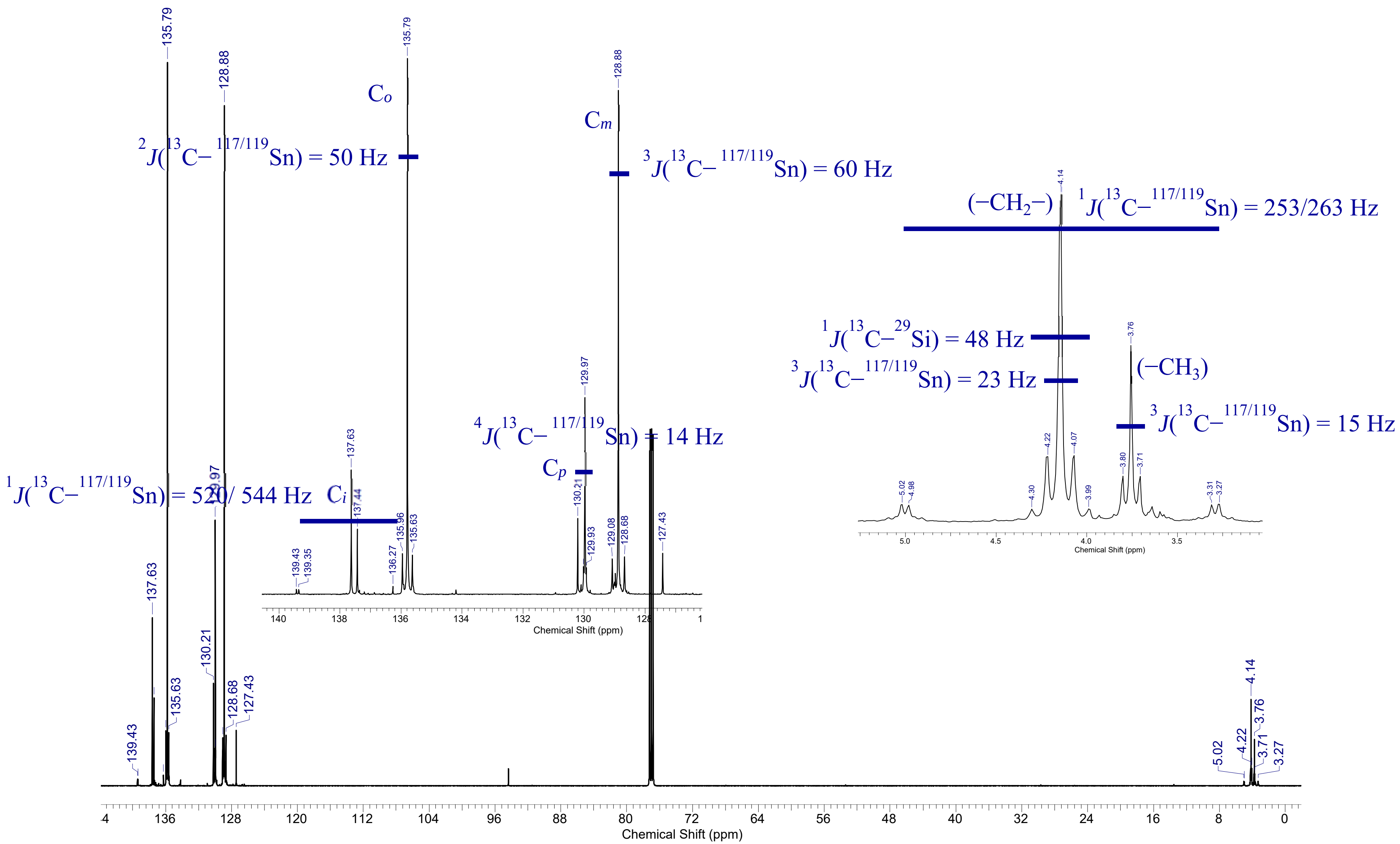

Figure S14. ^{13}C NMR spectrum (150.94 MHz, $CDCl_3$) of compound **3**.

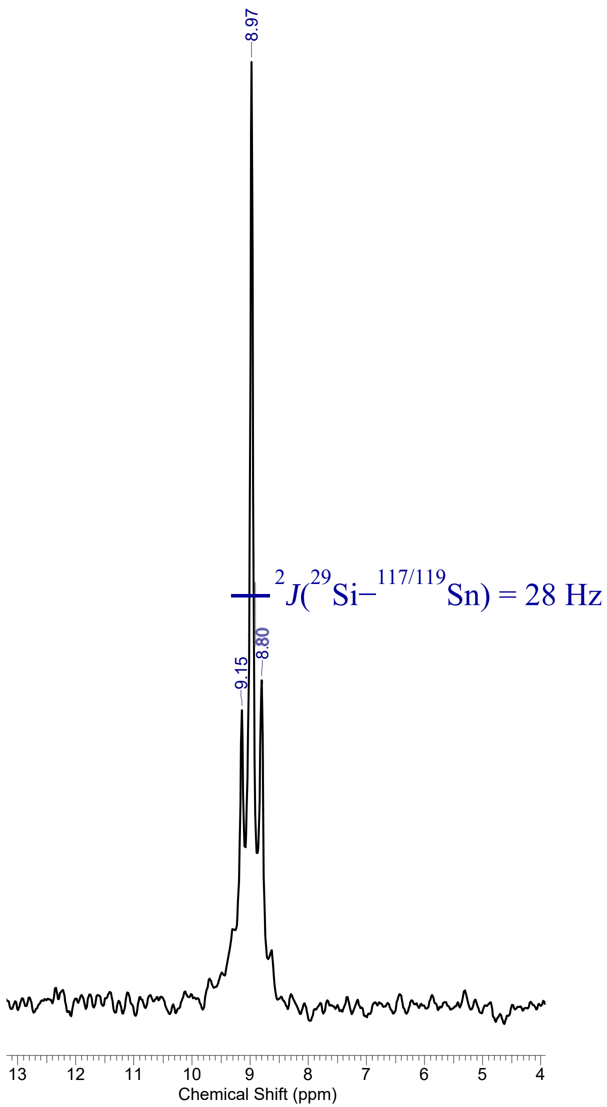

Figure S15. ^{29}Si NMR spectrum (79.52 MHz, $CDCl_3$) of compound **3**.

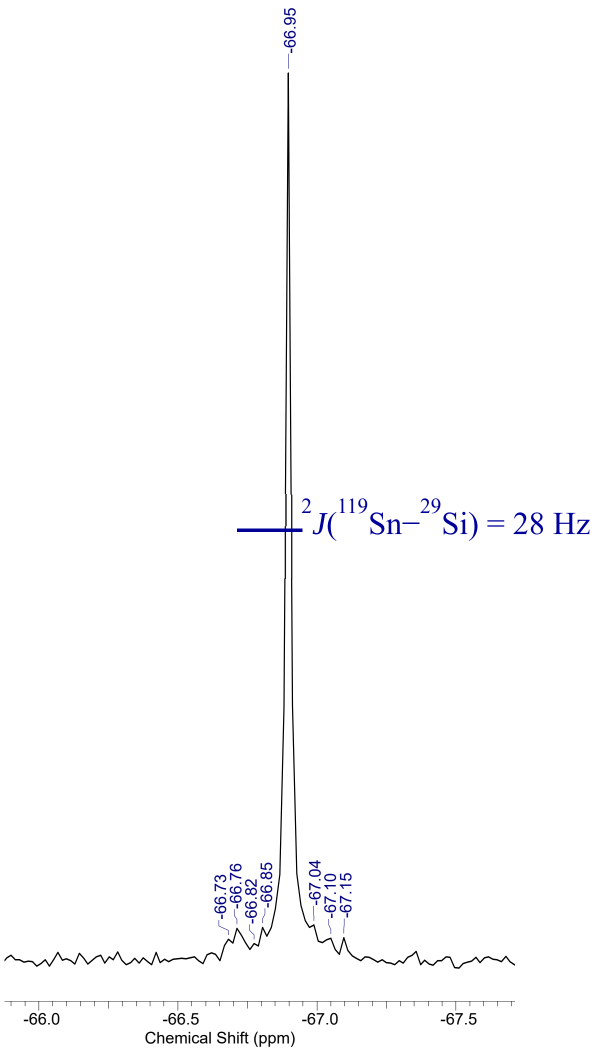

Figure S16. ^{119}Sn NMR spectrum (149.26 MHz, $CDCl_3$) of compound **3**.

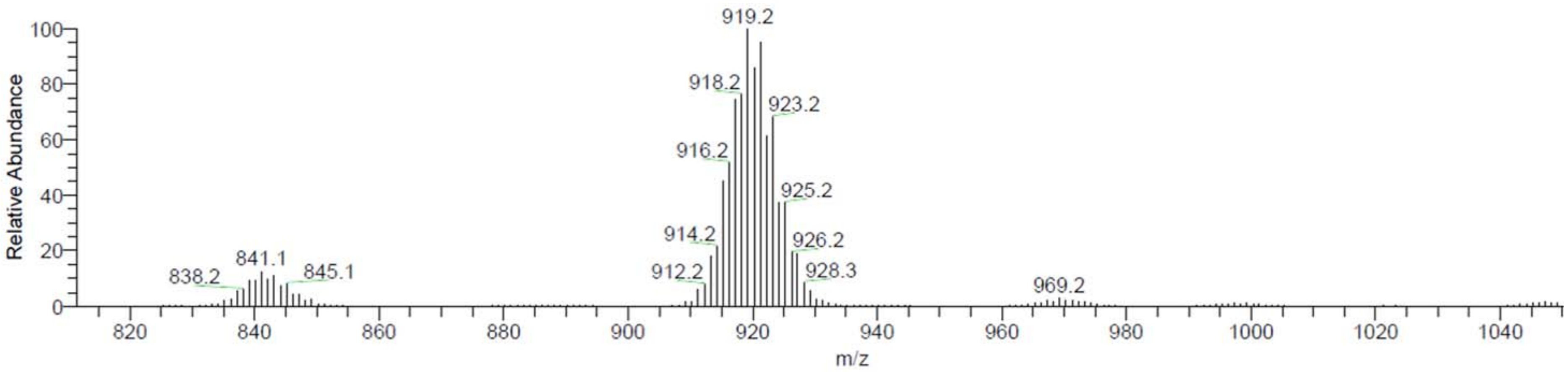

Figure S17. A fraction of the ESI MS spectrum (positive mode) of **3**. The horizontal axis shows the m/z values.

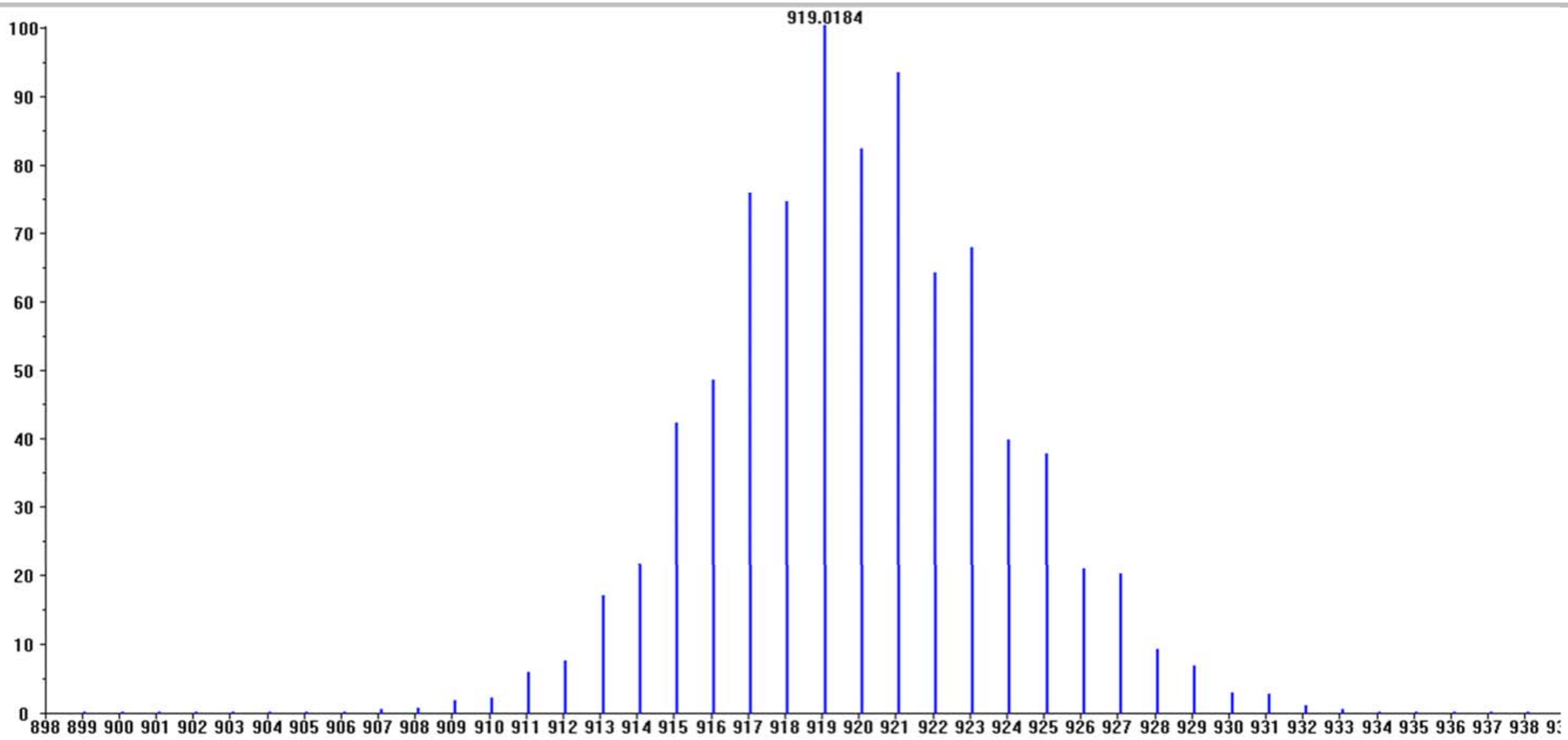

Figure S18. Simulated mass cluster for $C_{39}H_{44}NaSiSn_3^+$. The horizontal axis shows the m/z values.

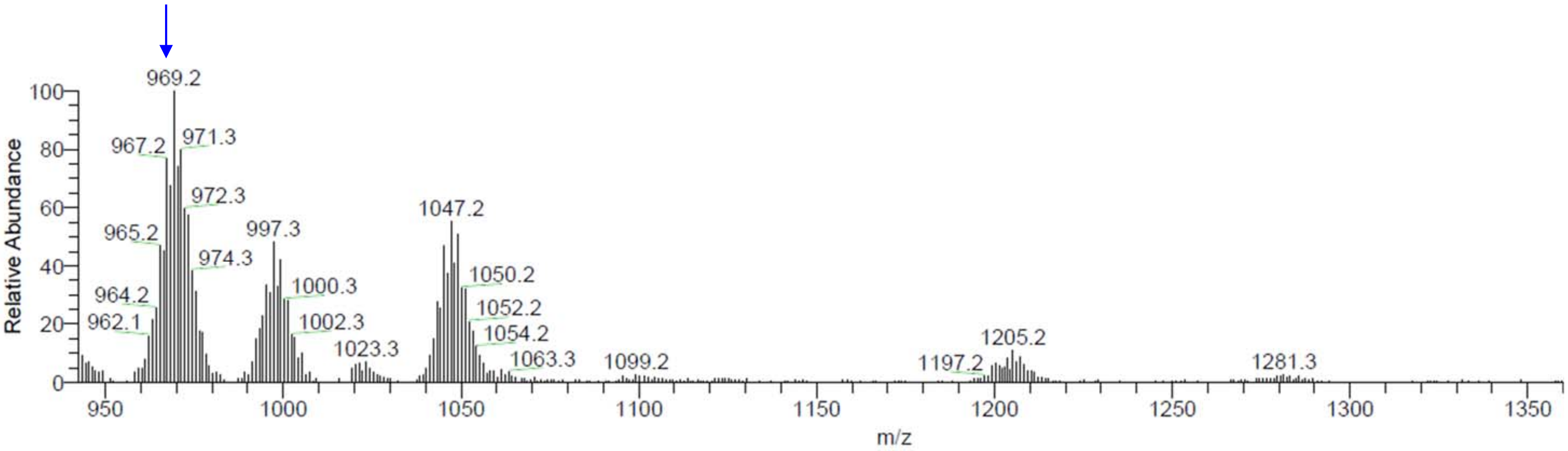

Figure S19. A fraction of the ESI MS spectrum (positive mode) of **3**. The blue arrow refers to the subsequent figure S20. The horizontal axis shows the m/z values.

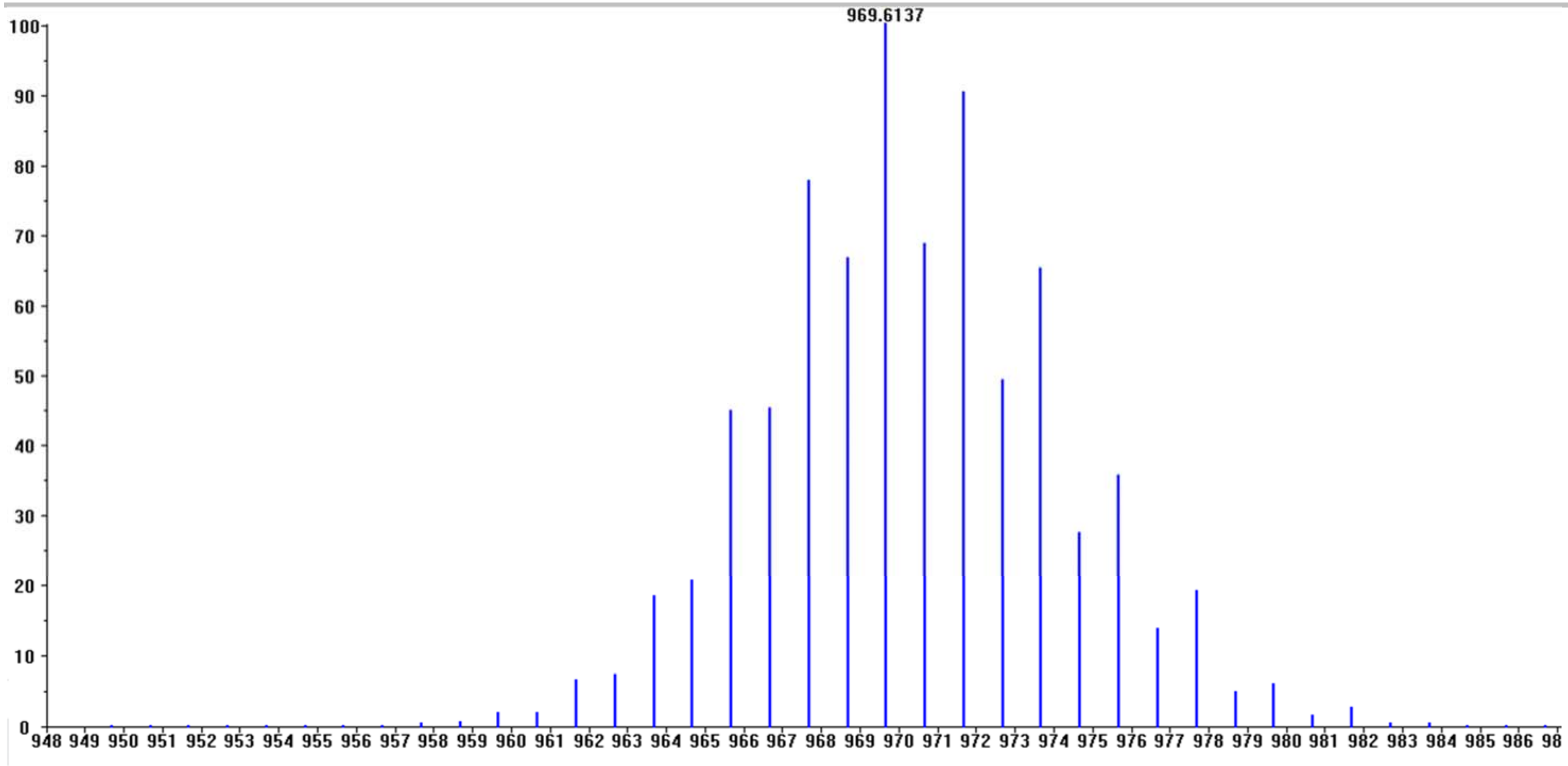

Figure S20. Simulated mass cluster for $C_{12}H_{23}I_3O_2SiSn_3^+$. The horizontal axis shows the m/z values.

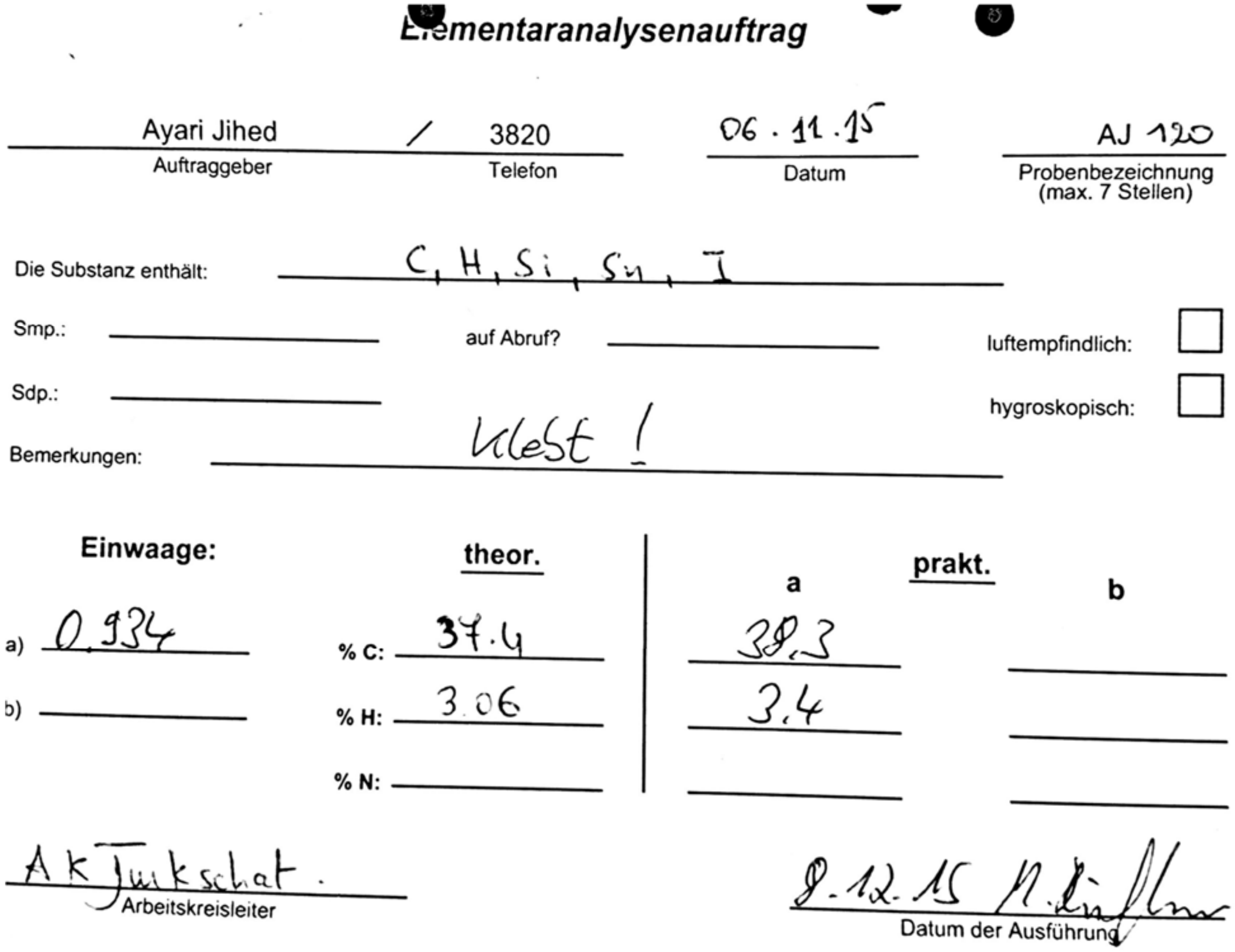

Elementaranalysenauftrag

Ayari Jihed /	3820	06.11.15	AJ 120
Auftraggeber	Telefon	Datum	Probenbezeichnung (max. 7 Stellen)

Die Substanz enthält: C, H, Si, Sn, I

Smp.: auf Abruf? luftempfindlich: ☐

Sdp.: hygroskopisch: ☐

Bemerkungen: klebt!

Einwaage:		theor.	prakt. a	prakt. b
a) 0.934	% C:	37.4	38.3	
b)	% H:	3.06	3.4	
	% N:			

AK Jurkschat. — Arbeitskreisleiter

8.12.15 [illegible] — Datum der Ausführung

Figure S21. Elemental analysis of **3**.

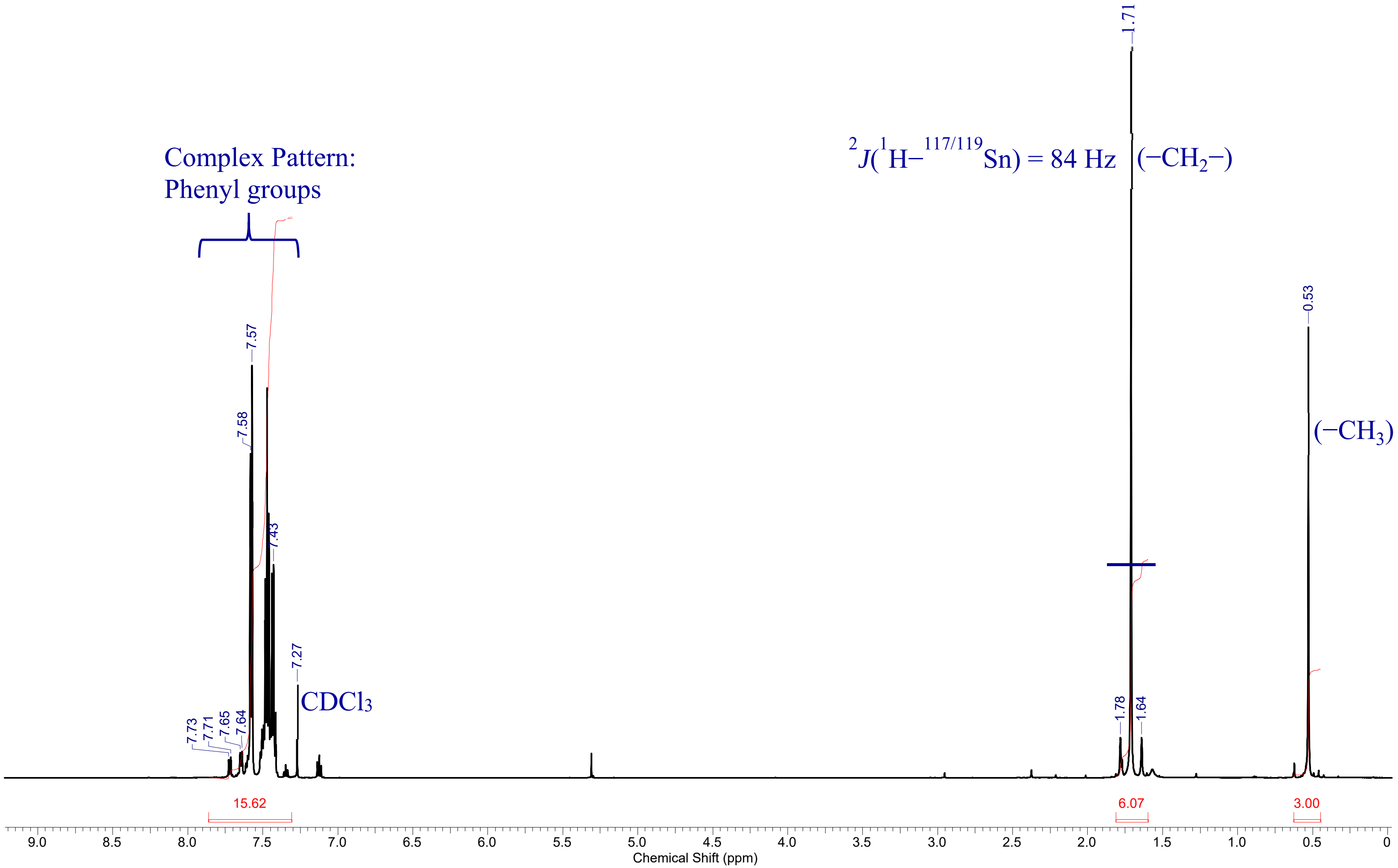

Figure S22. ^{1}H NMR spectrum (400.25 MHz, $CDCl_3$) of compound **4**.

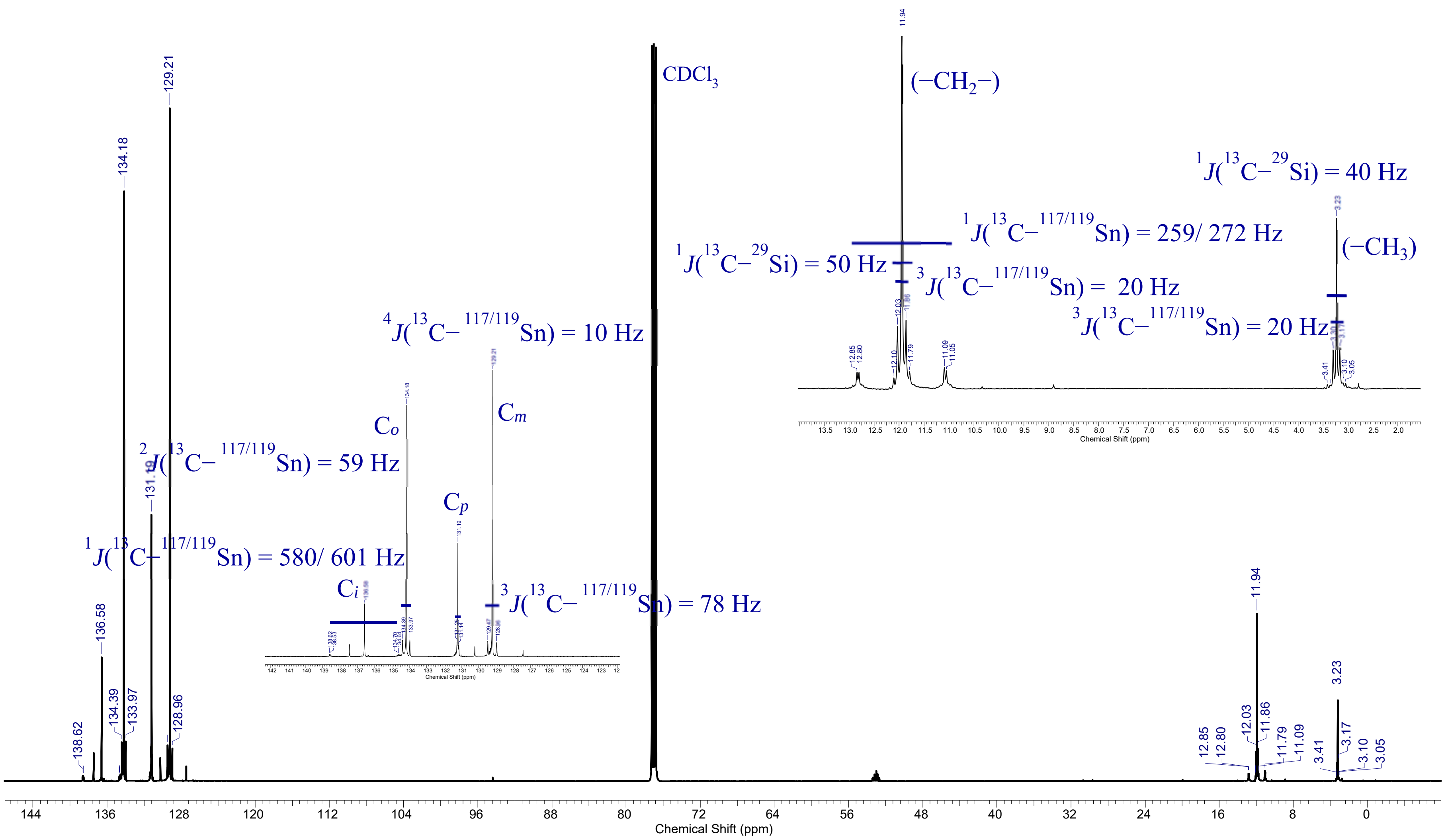

Figure S23. ^{13}C NMR spectrum (150.94 MHz, $CDCl_3$) of compound **4**.

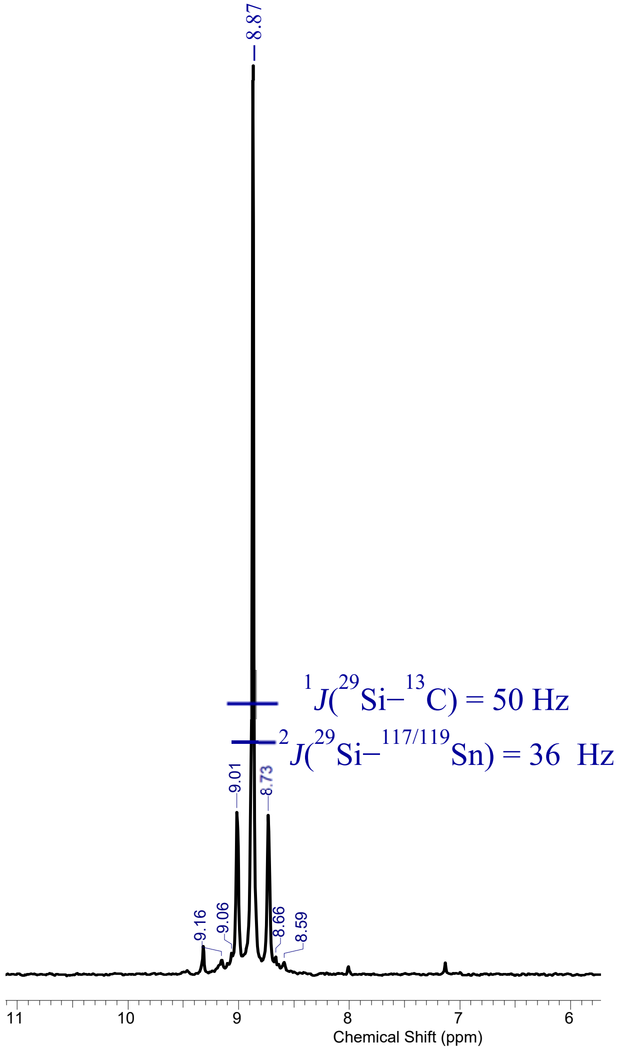

Figure S24. ^{29}Si NMR spectrum (79.52 MHz, $CDCl_3$) of compound **4**.

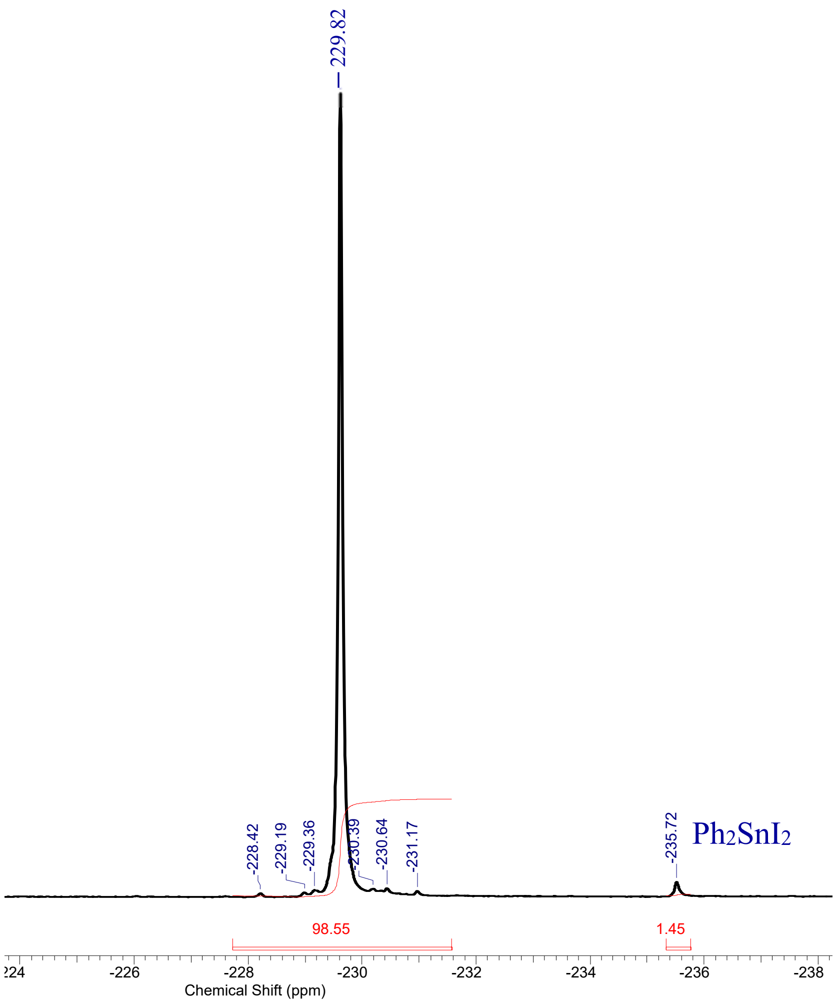

Figure S25. ^{119}Sn NMR spectrum (149.26 MHz, $CDCl_3$) of compound **4**.

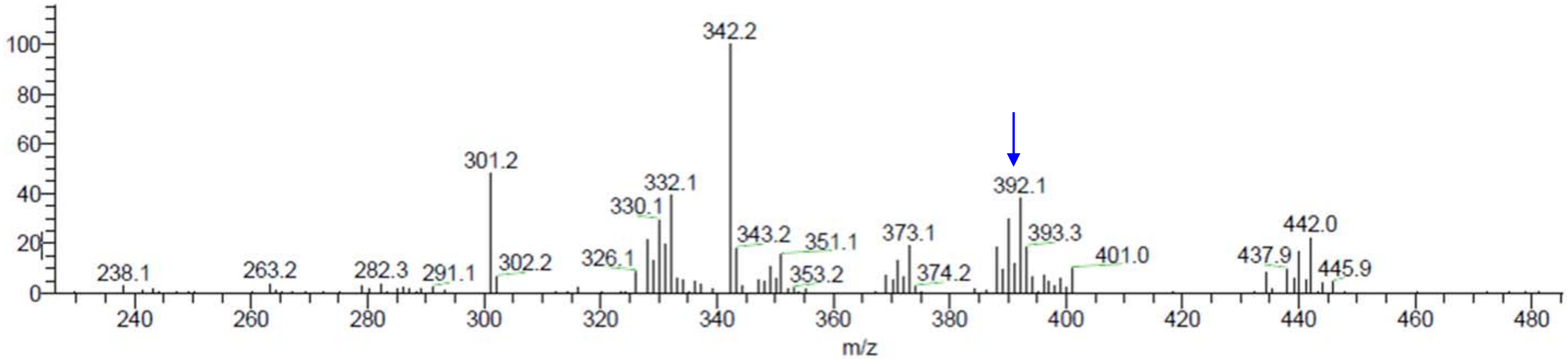

Figure S26. Fraction of the ESI MS spectrum (positive mode) of **4**. The blue arrow refers to the subsequent figure S27.The horizontal axis shows the m/z values.

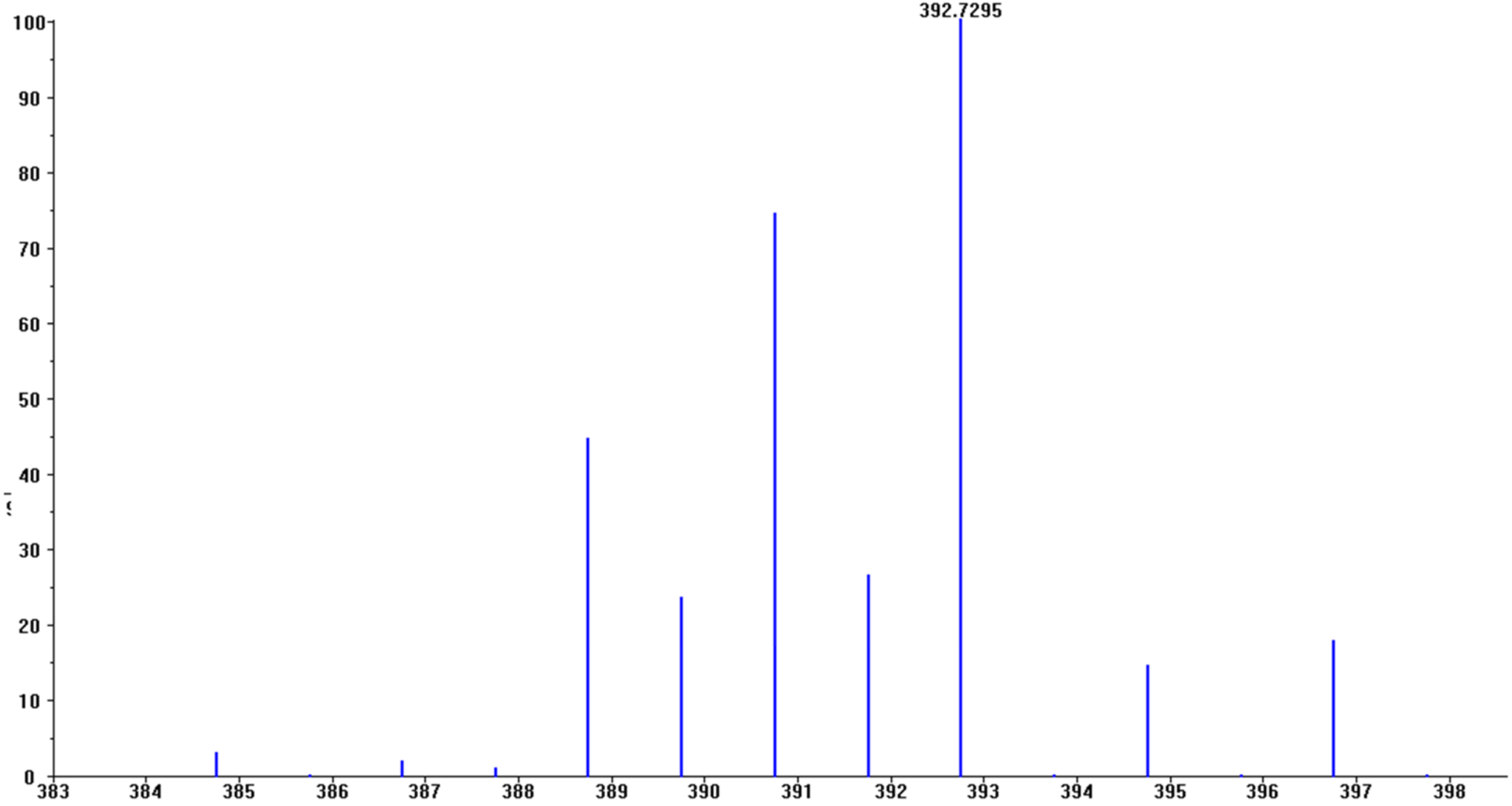

Figure S27. Simulated mass cluster for $H_3I_2SnO^+$. The horizontal axis shows the m/z values.

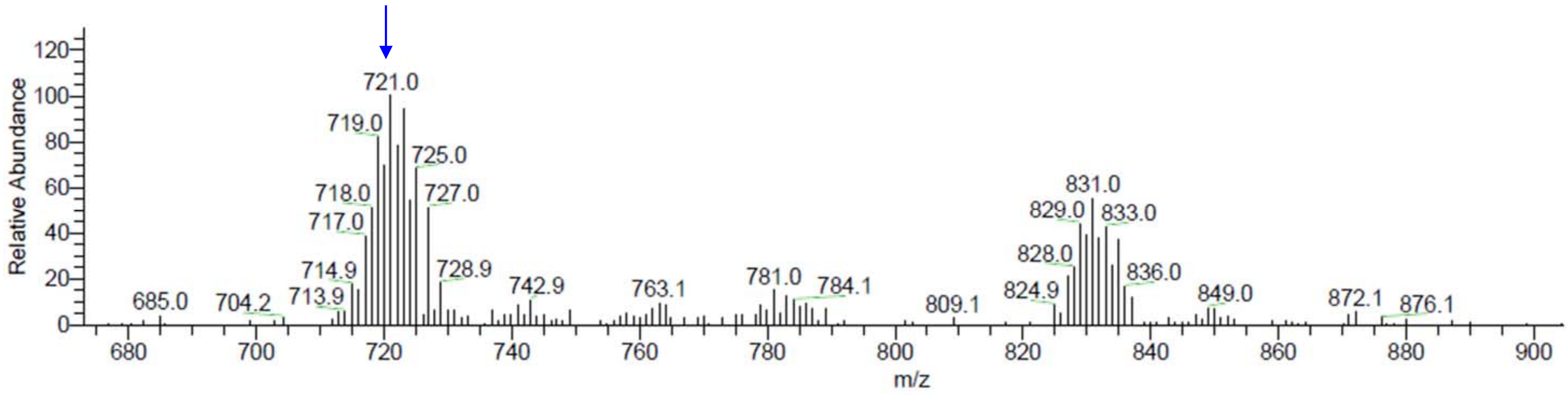

Figure S28. Fraction of the ESI MS spectrum (positive mode) of **4**. The horizontal axis shows the m/z values.

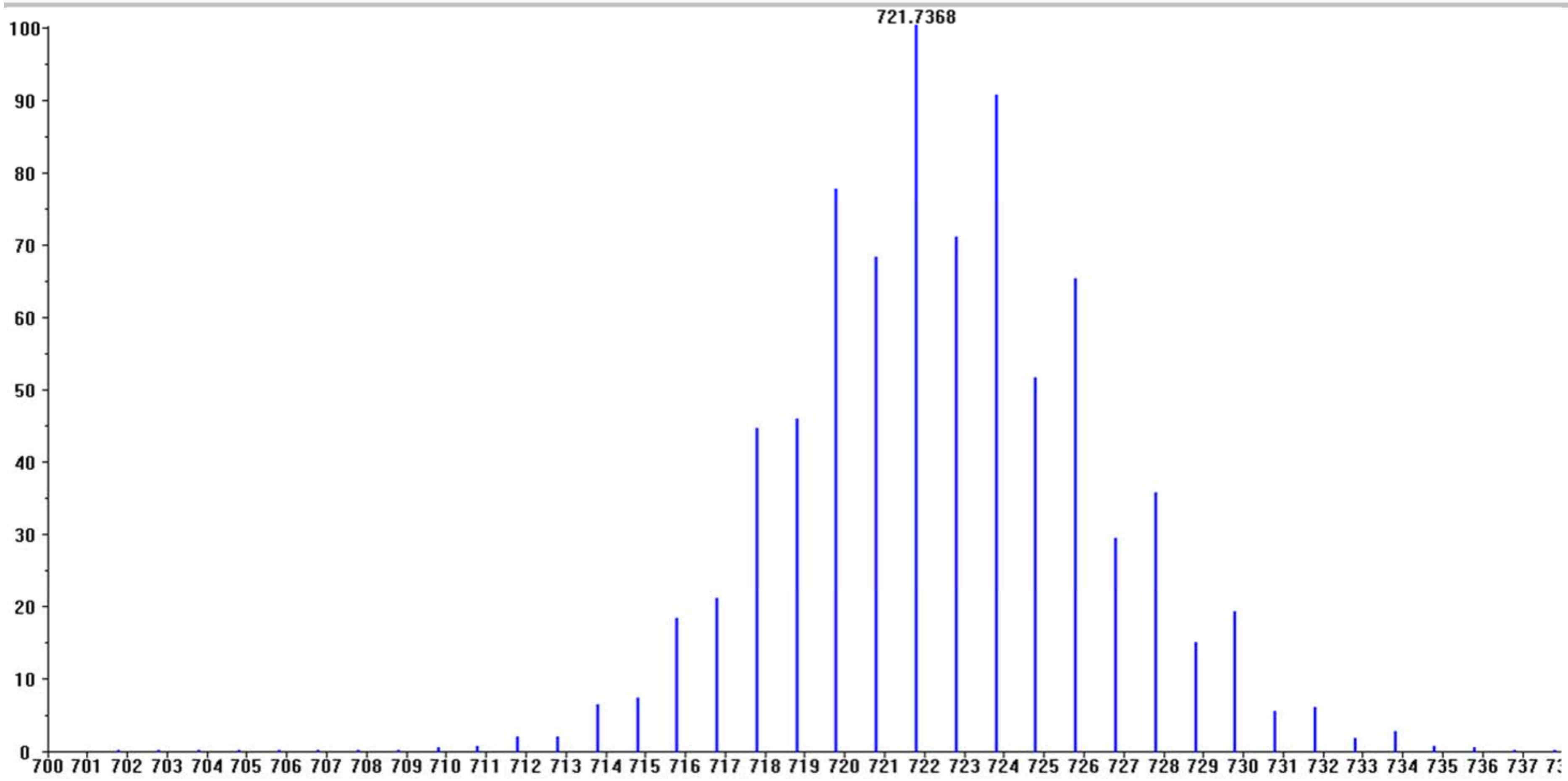

Figure S29. Simulated mass cluster for $C_{16}H_{19}ISiSn_3^+$. The horizontal axis shows the m/z values.

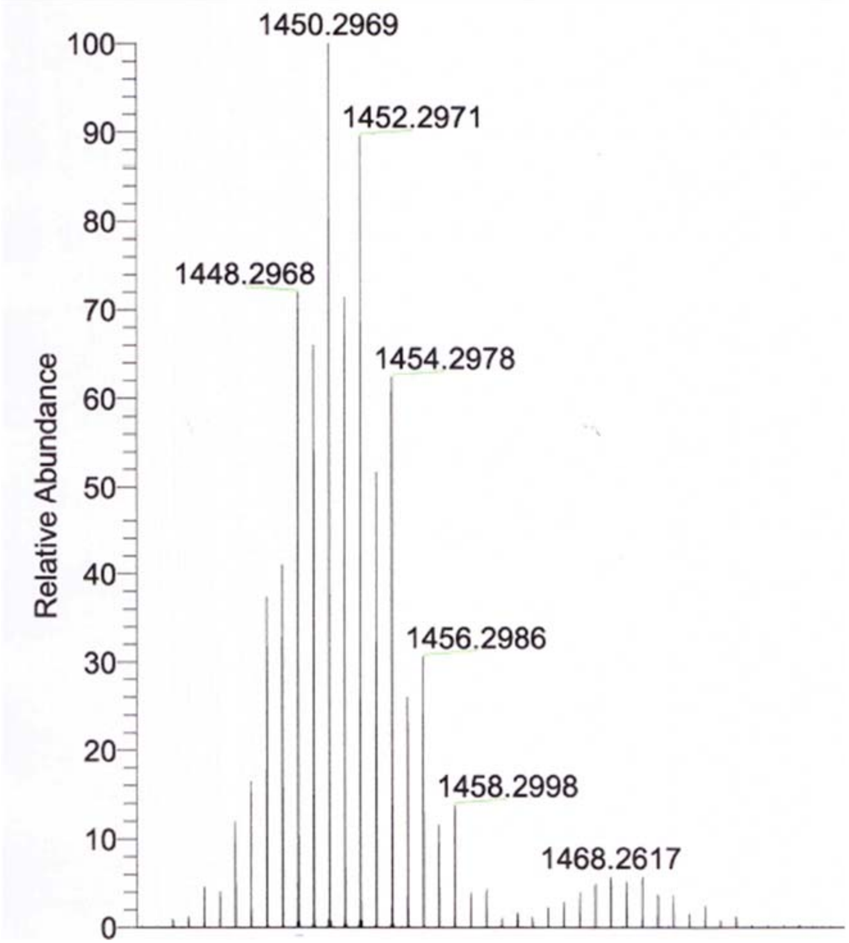

Figure S30. A fraction of the ESI MS spectrum (negative mode) of **4**. The horizontal axis shows the m/z values.

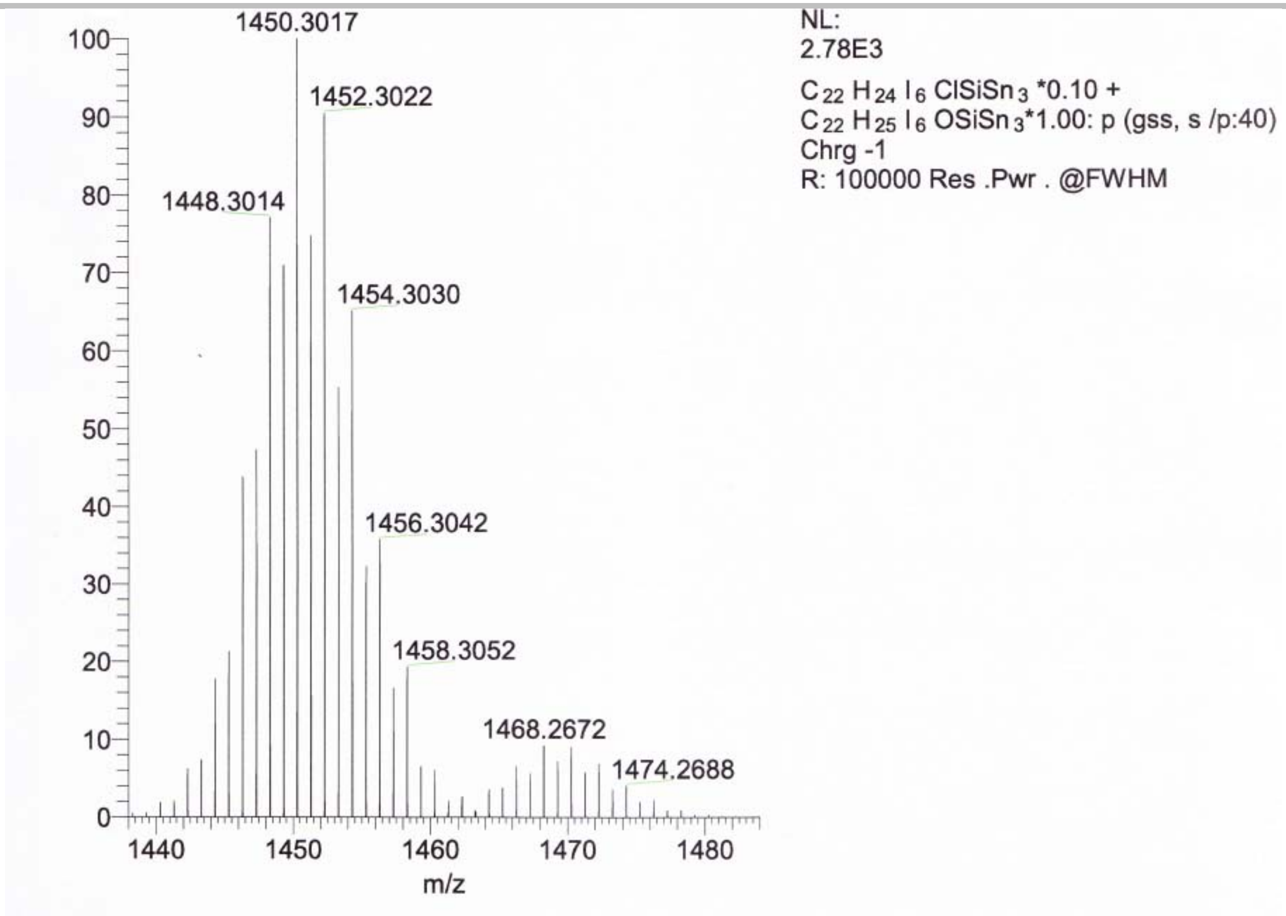

Figure S31. Simulated mass cluster for $C_{22}H_{24}I_6ClSiSn_3 \cdot 0.10$ + $C_{22}H_{25}I_6OSiSn_3 \cdot 1.0$. The horizontal axis shows the m/z values.

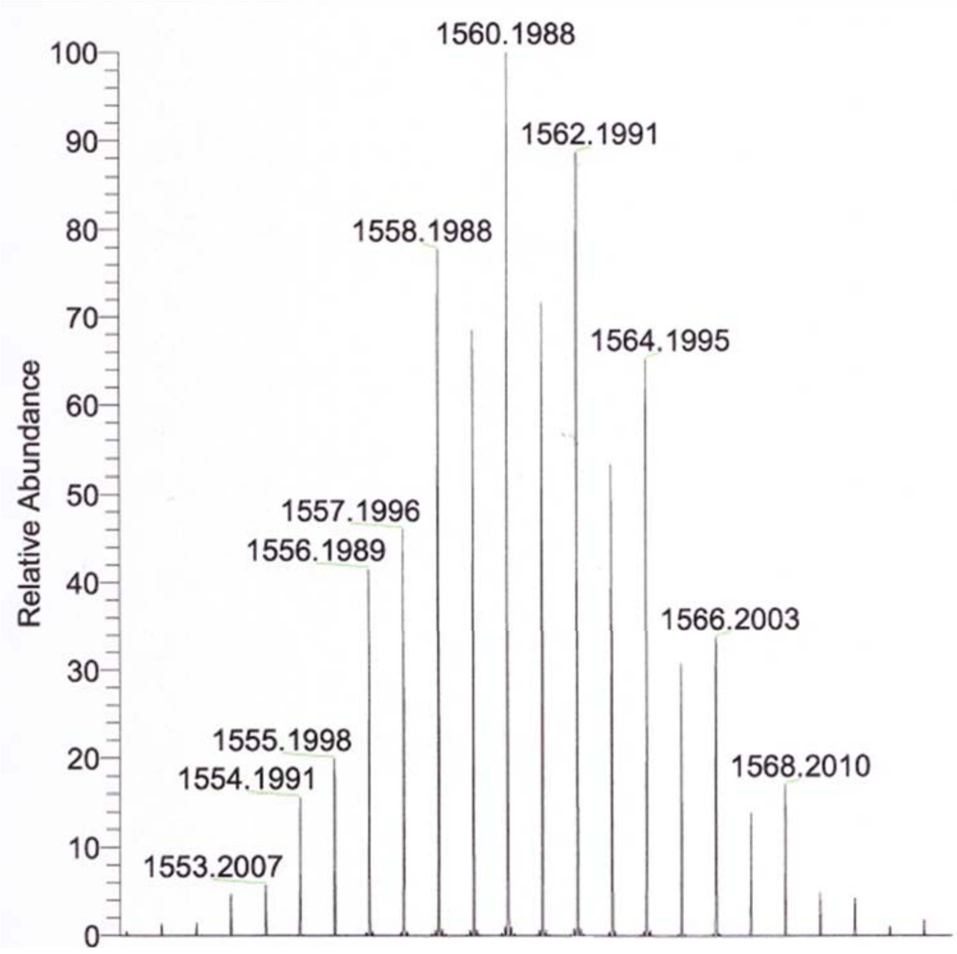

Figure S32. A fraction of the ESI MS spectrum (negative mode) of **4**. The horizontal axis shows the m/z values.

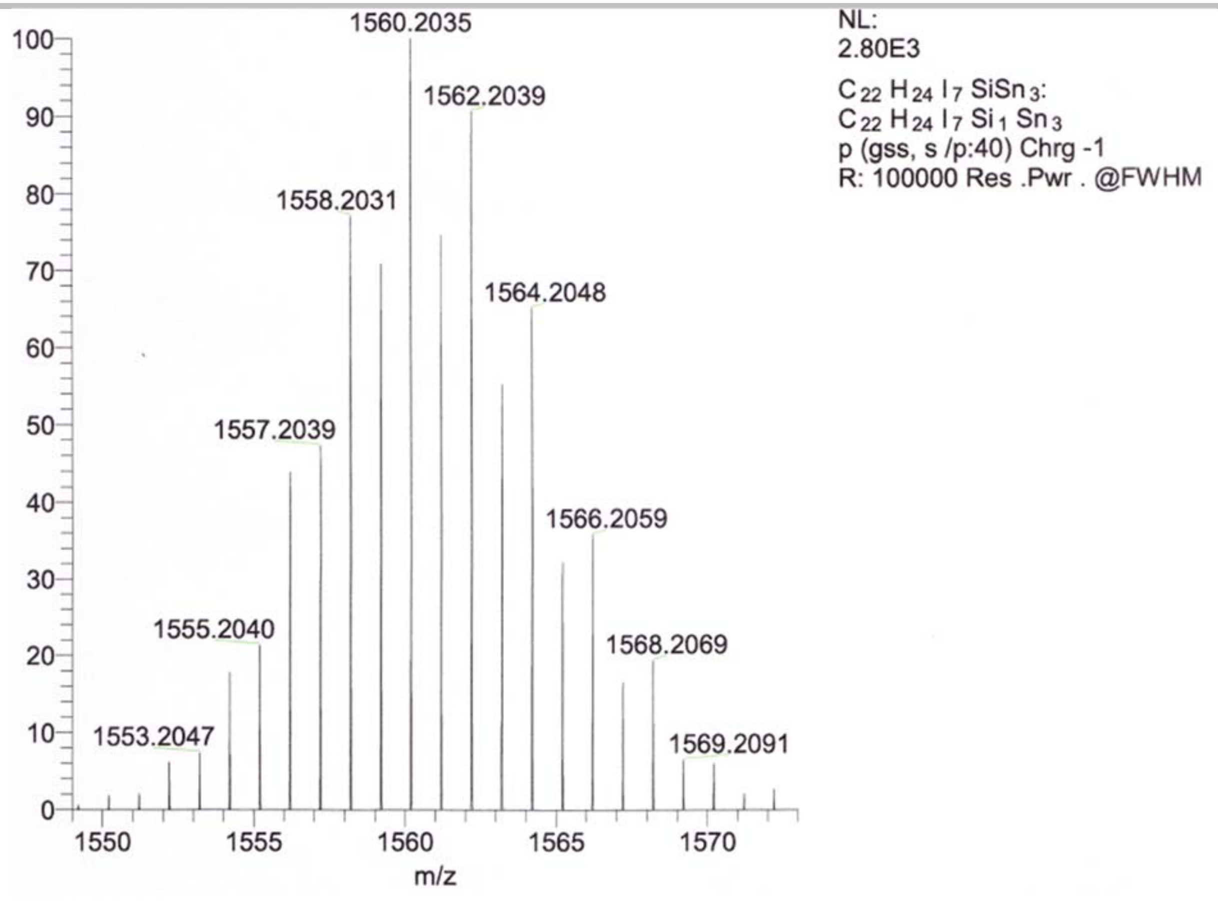

Figure S33. Simulated mass cluster for $C_{22}H_{24}I_7SiSn_3^-$. The horizontal axis shows the m/z values.

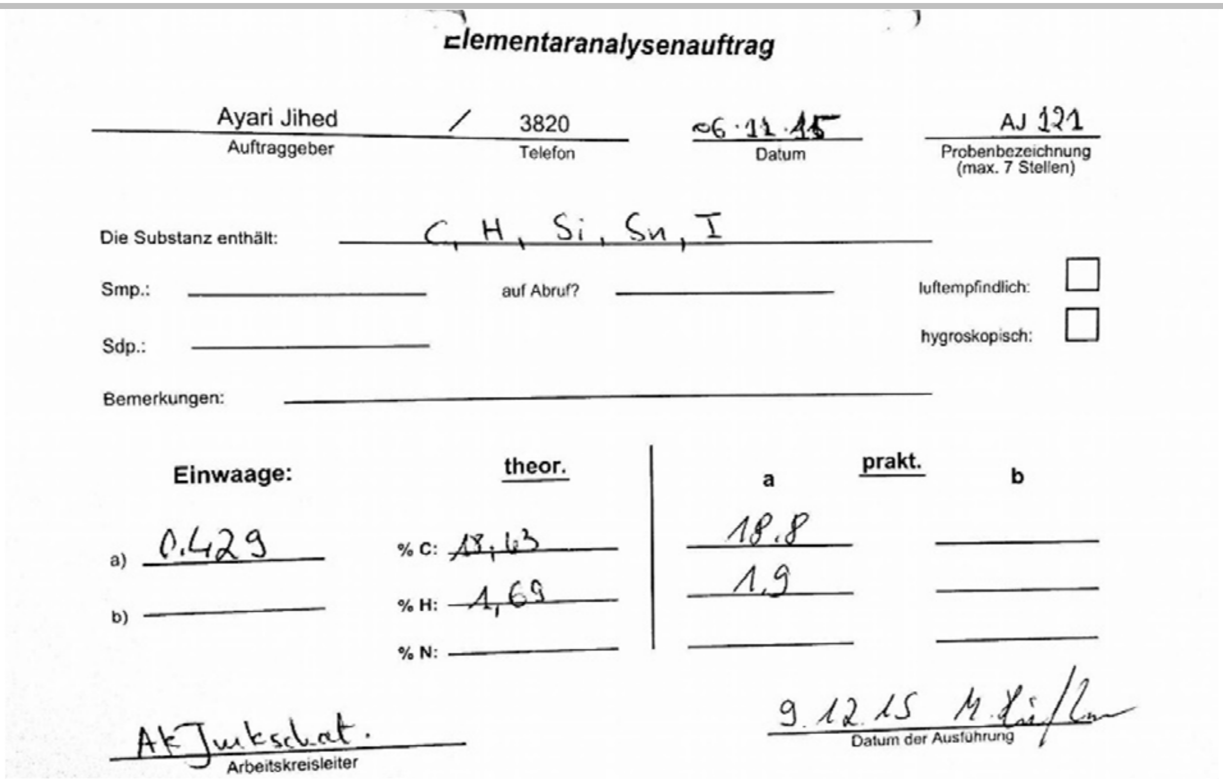

Elementaranalysenauftrag

Ayari Jihed / 3820 06.11.15 AJ 121

Auftraggeber Telefon Datum Probenbezeichnung (max. 7 Stellen)

Die Substanz enthält: C, H, Si, Sn, I

Smp.: auf Abruf? luftempfindlich: ☐

Sdp.: hygroskopisch: ☐

Bemerkungen:

Einwaage:		theor.	prakt. a	prakt. b
a) 0.429	% C:	18,63	18.8	
b)	% H:	1,69	1,9	
	% N:			

AK Jutzschat. Arbeitskreisleiter

9.12.15 Datum der Ausführung

Figure S34. Elemental analysis of **4**.

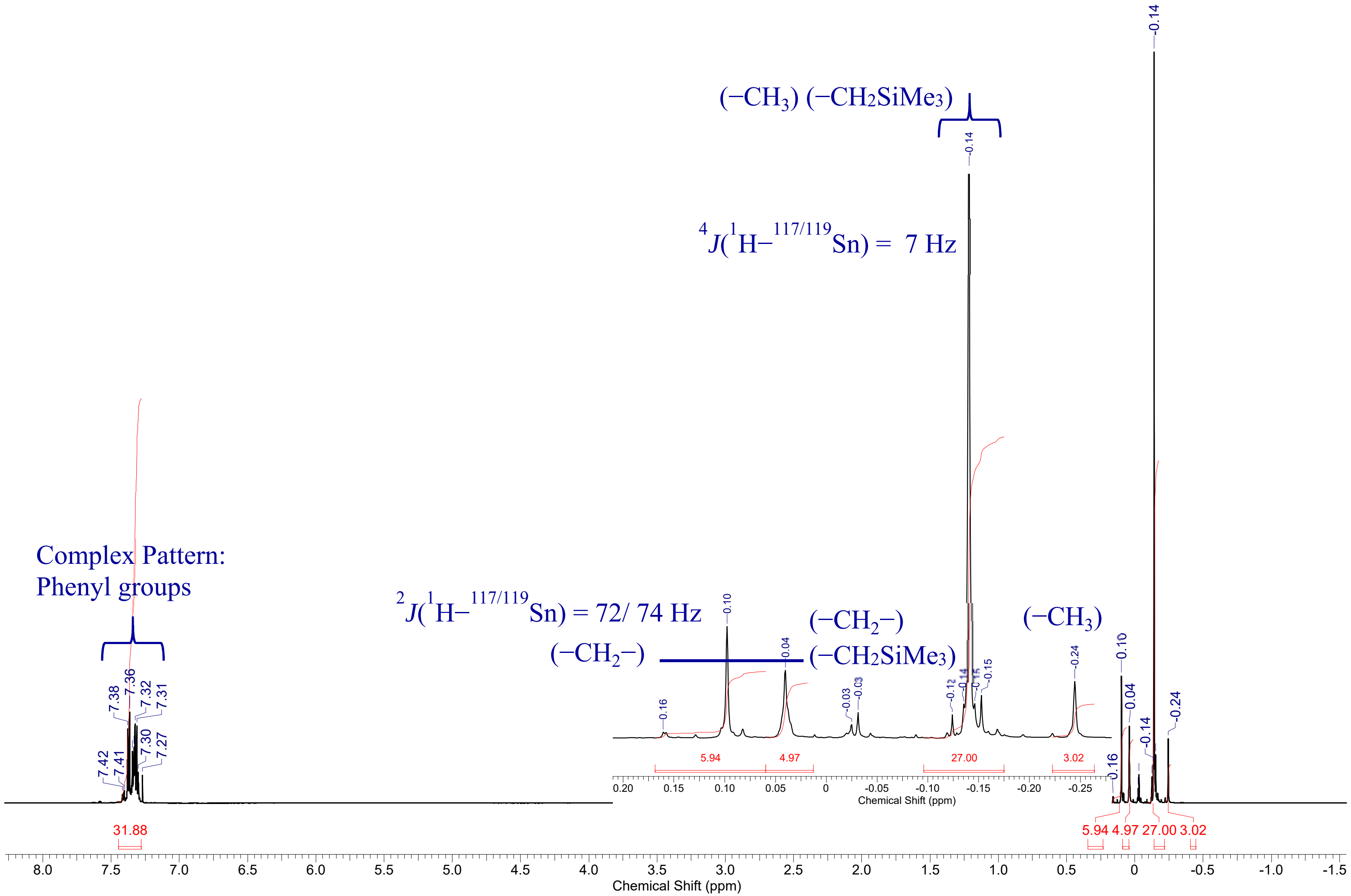

Figure S35. ^{1}H NMR spectrum (600.29 MHz, $CDCl_3$) of compound **5**.

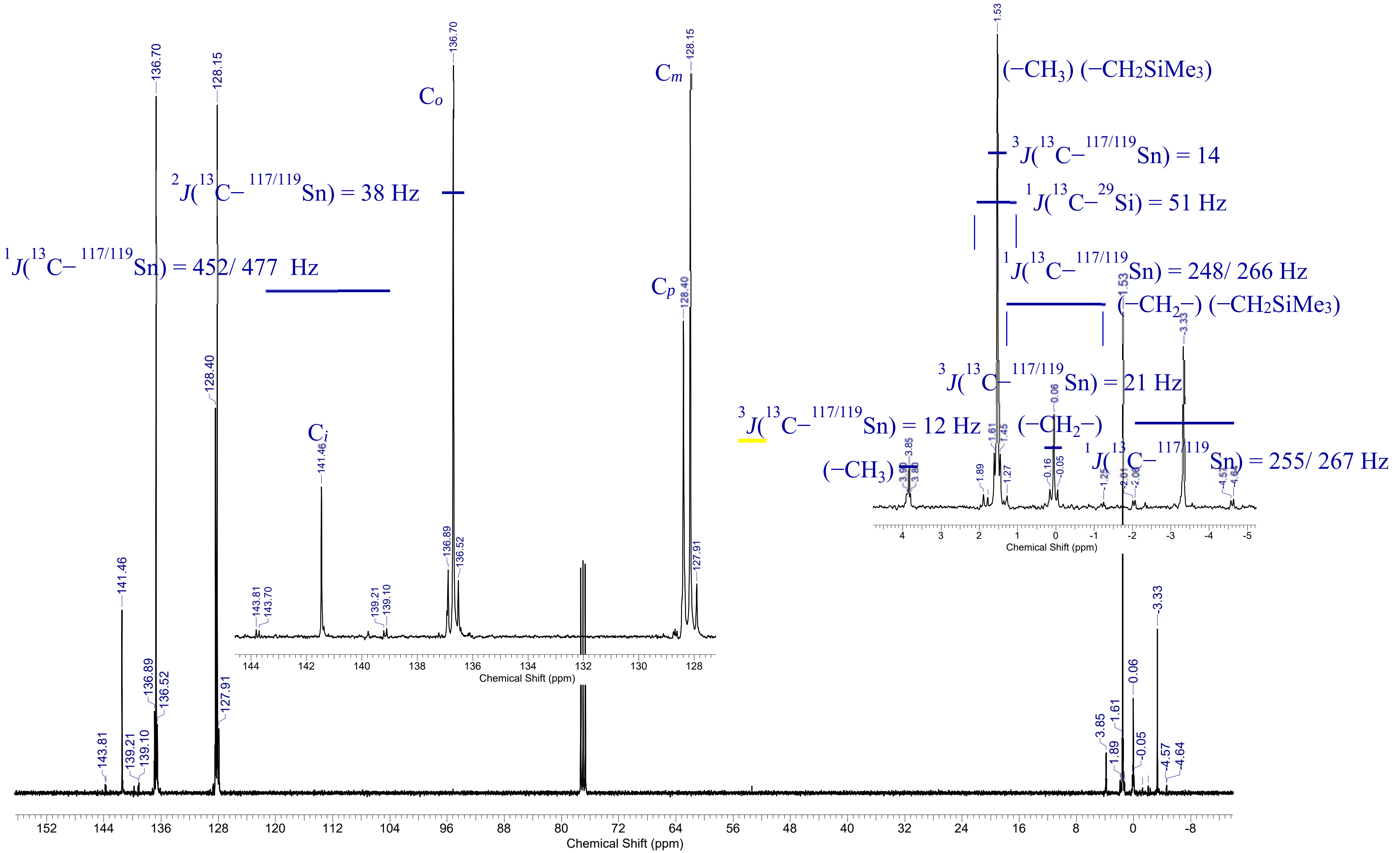

Figure S36. ^{13}C NMR spectrum (100.46 MHz, CDCl3) of compound **5**.

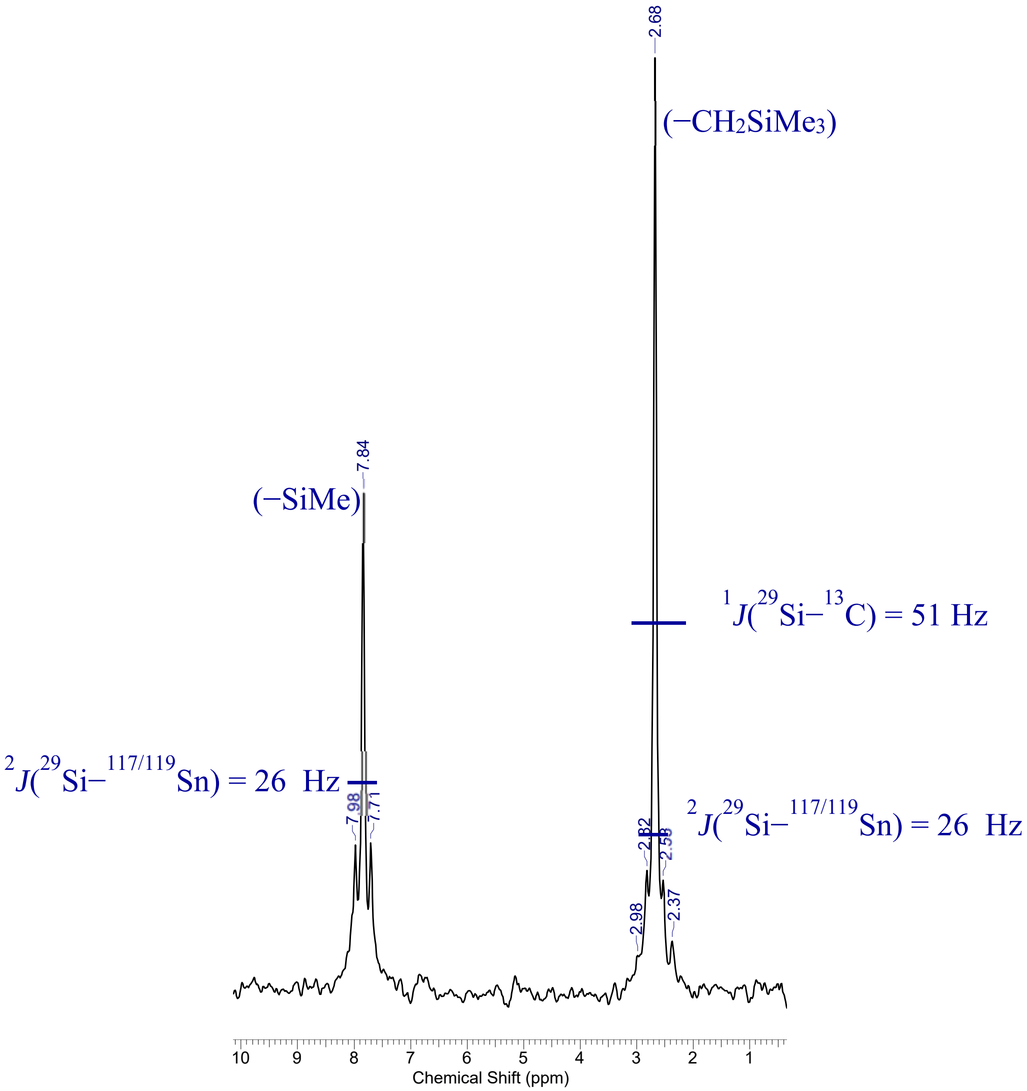

Figure S37. ^{29}Si NMR spectrum (79.52 MHz, $CDCl_3$) of compound **5**.

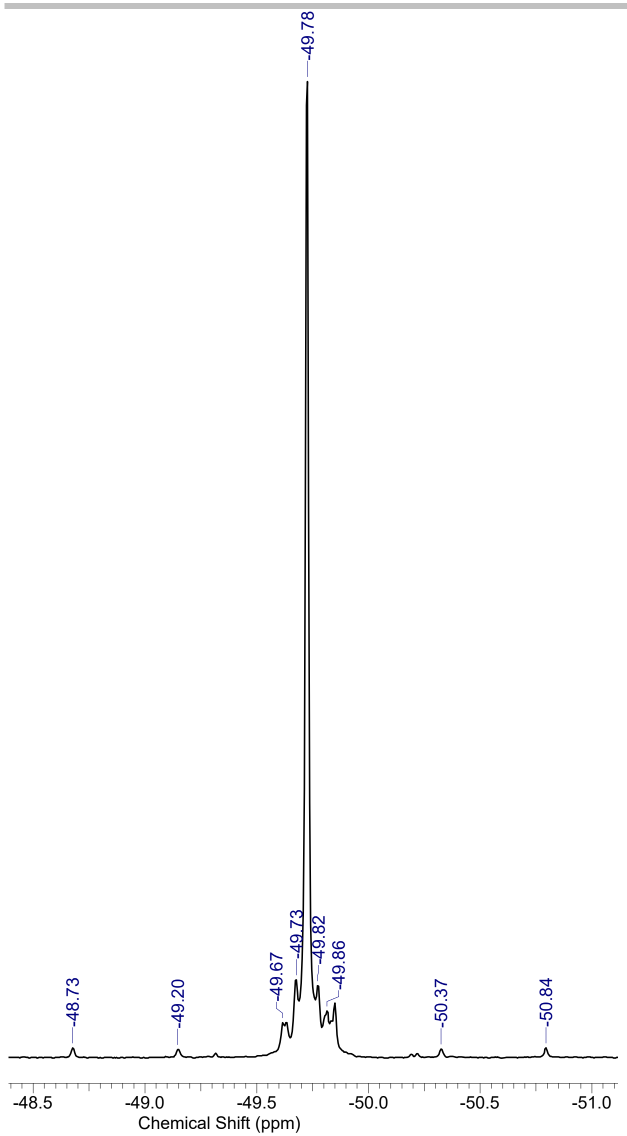

Figure S38. ^{119}Sn NMR spectrum (223.85MHz, $CDCl_3$) of compound **5**.

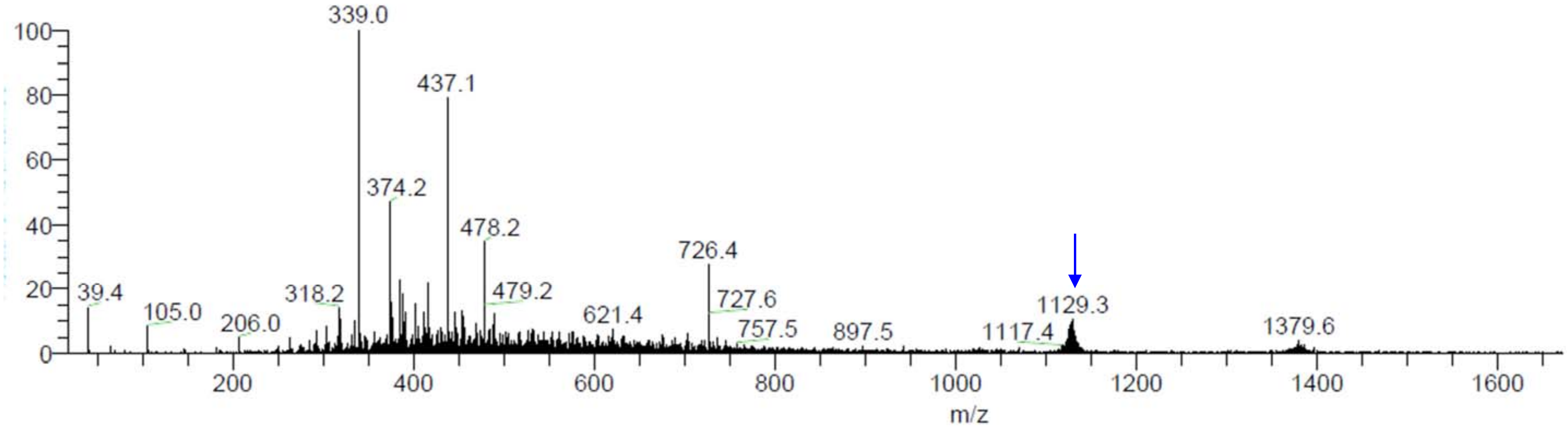

Figure S39. ESI MS spectrum (positive mode) of **5**. The horizontal axis shows the m/z values. The blue arrow refers to the subsequent Figures S40 and S41.

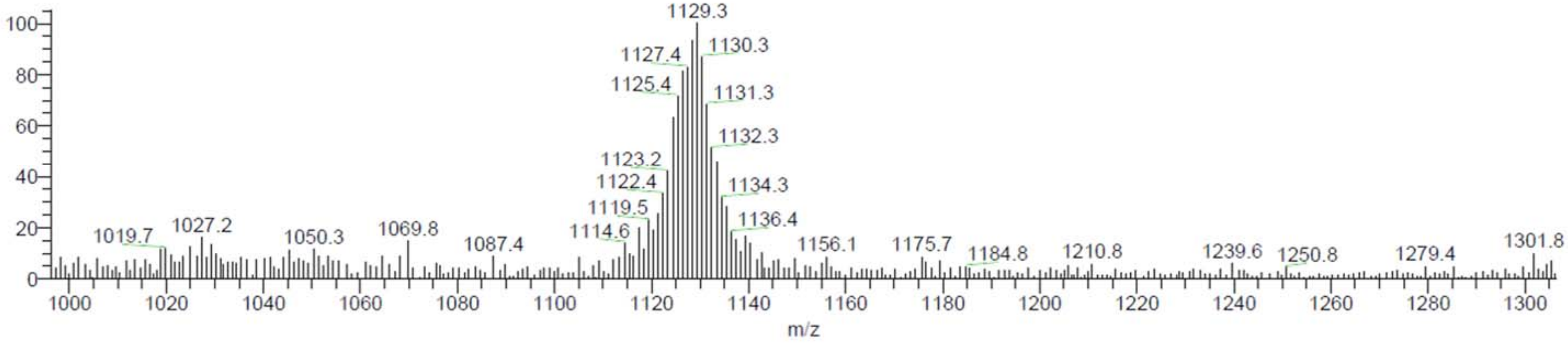

Figure S40. A fraction of the ESI MS spectrum (positive mode) of **5.** The horizontal axis shows the m/z values.

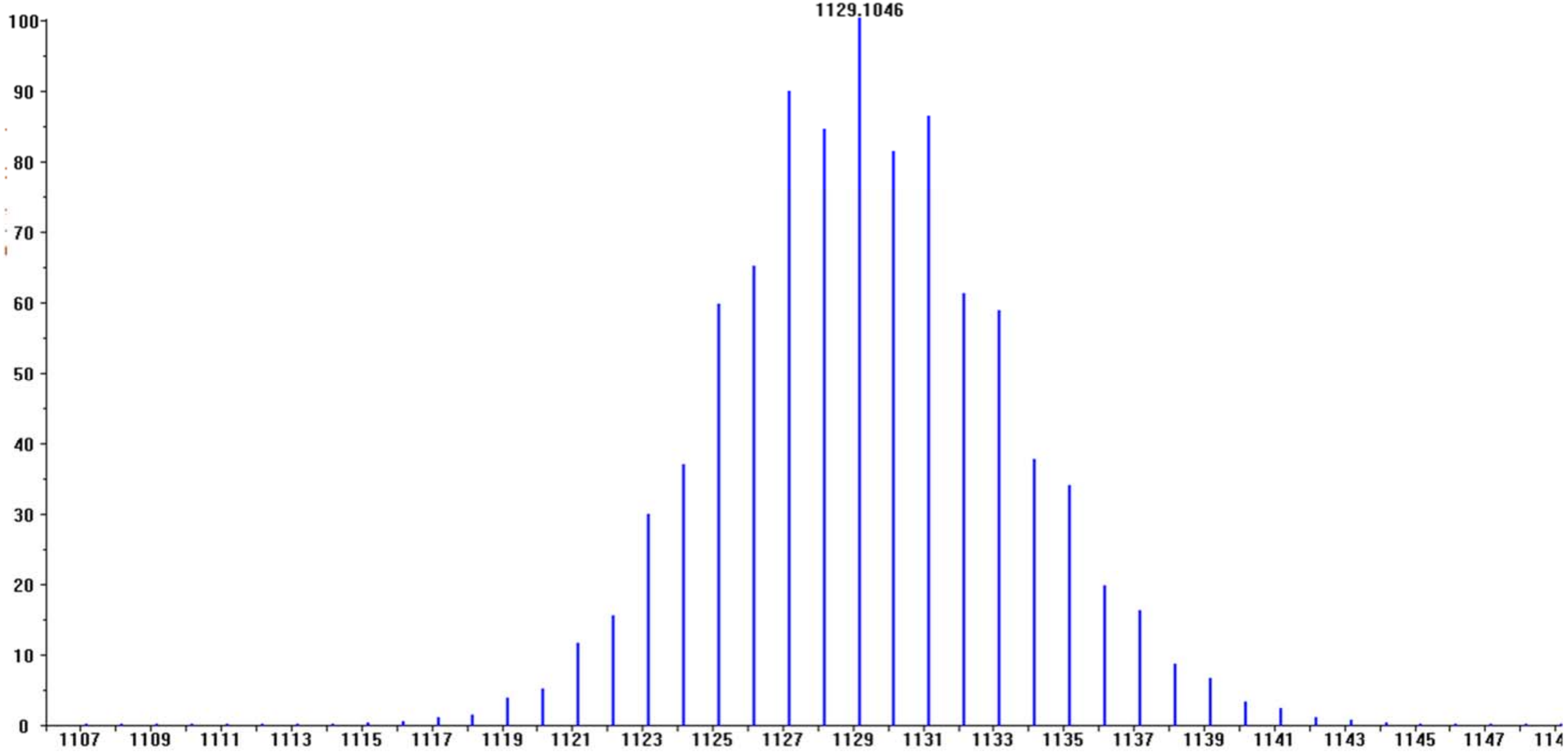

Figure S41. Simulated mass cluster for $C_{43}H_{69}Cl_2O_2Si_3Sn_3^+$. The horizontal axis shows the m/z values.

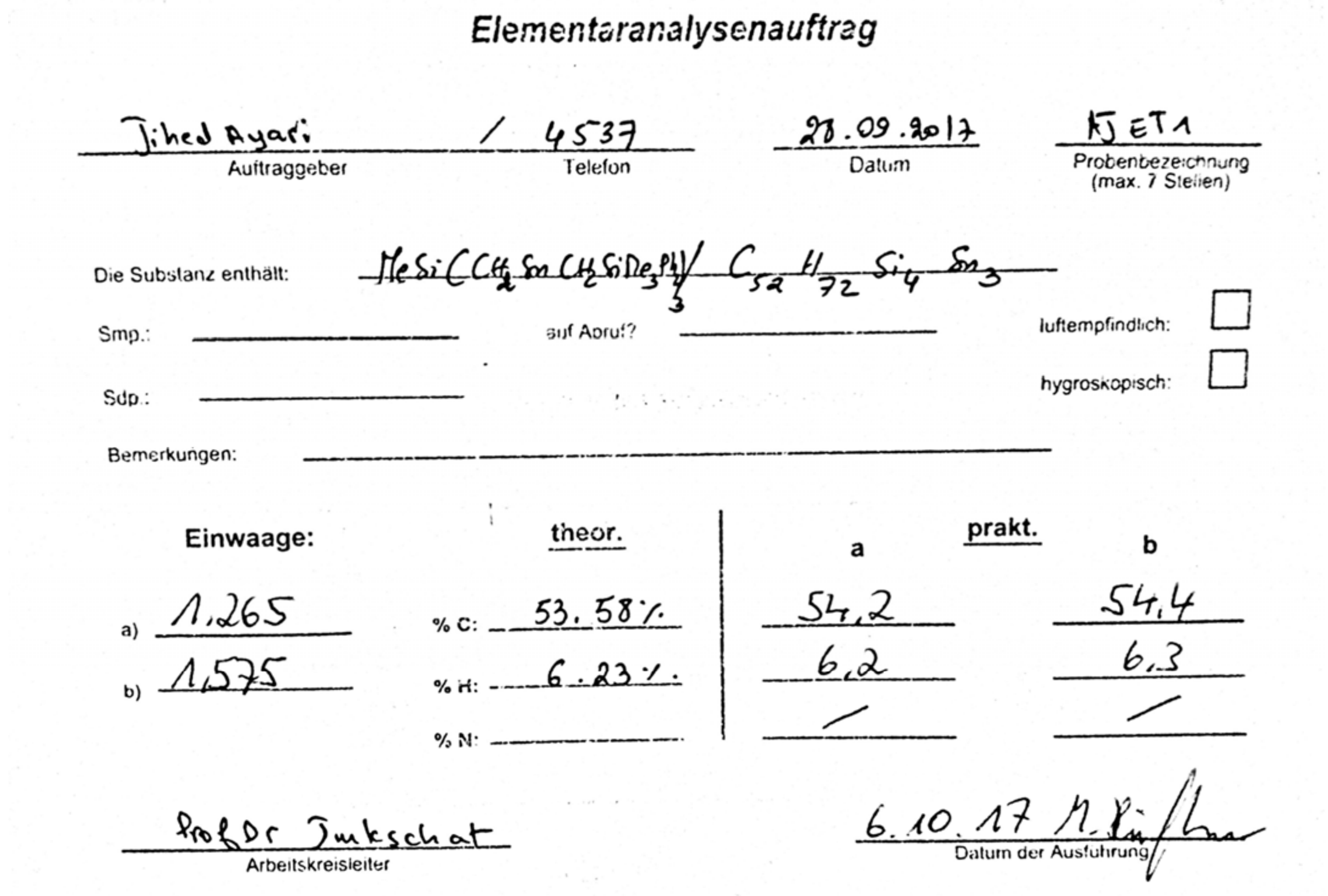

Elementaranalysenauftrag

Jihed Ayari / 4537 — Auftraggeber / Telefon

28.09.2017 — Datum

KJ ET1 — Probenbezeichnung (max. 7 Stellen)

Die Substanz enthält: $MeSi(CH_2Sn(CH_2SiMe_3)Ph_2)_3$ / $C_{52}H_{72}Si_4Sn_3$

Smp.: ______ auf Abruf? ______ luftempfindlich: ☐

Sdp.: ______ hygroskopisch: ☐

Bemerkungen: ______

Einwaage:		theor.	prakt. a	prakt. b
a) 1,265	% C:	53.58 %	54,2	54,4
b) 1,575	% H:	6.23 %	6,2	6,3
	% N:		/	/

Prof Dr Jurkschat — Arbeitskreisleiter

6.10.17 M. [illegible] — Datum der Ausführung

Figure S42. Elemental analysis of **5**.

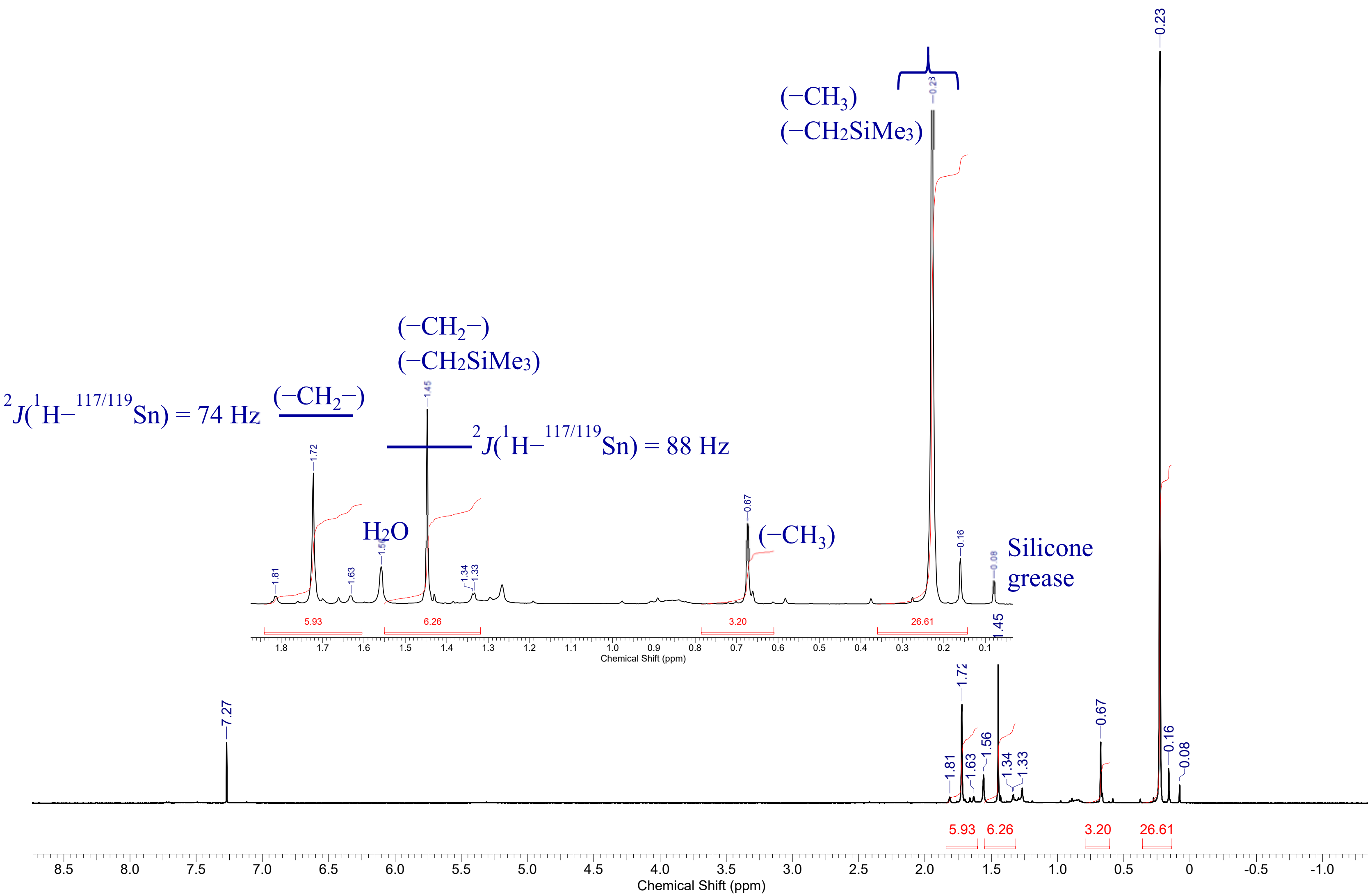

Figure S43. ^{1}H NMR spectrum (400.25 MHz, CDCl3) of compound **6**.

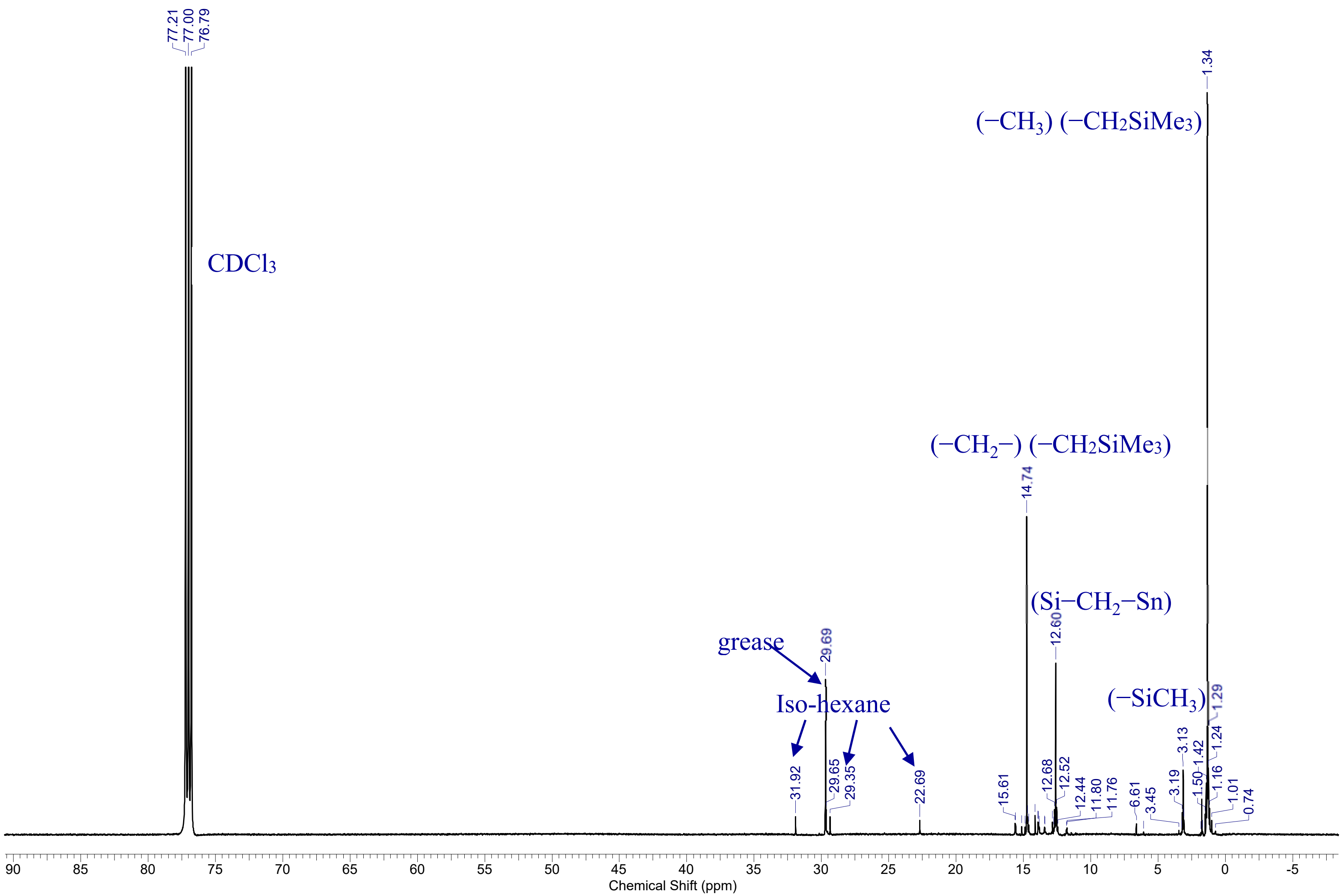

Figure S44. ^{13}C NMR spectrum (150.46 MHz, $CDCl_3$) of compound **6**.

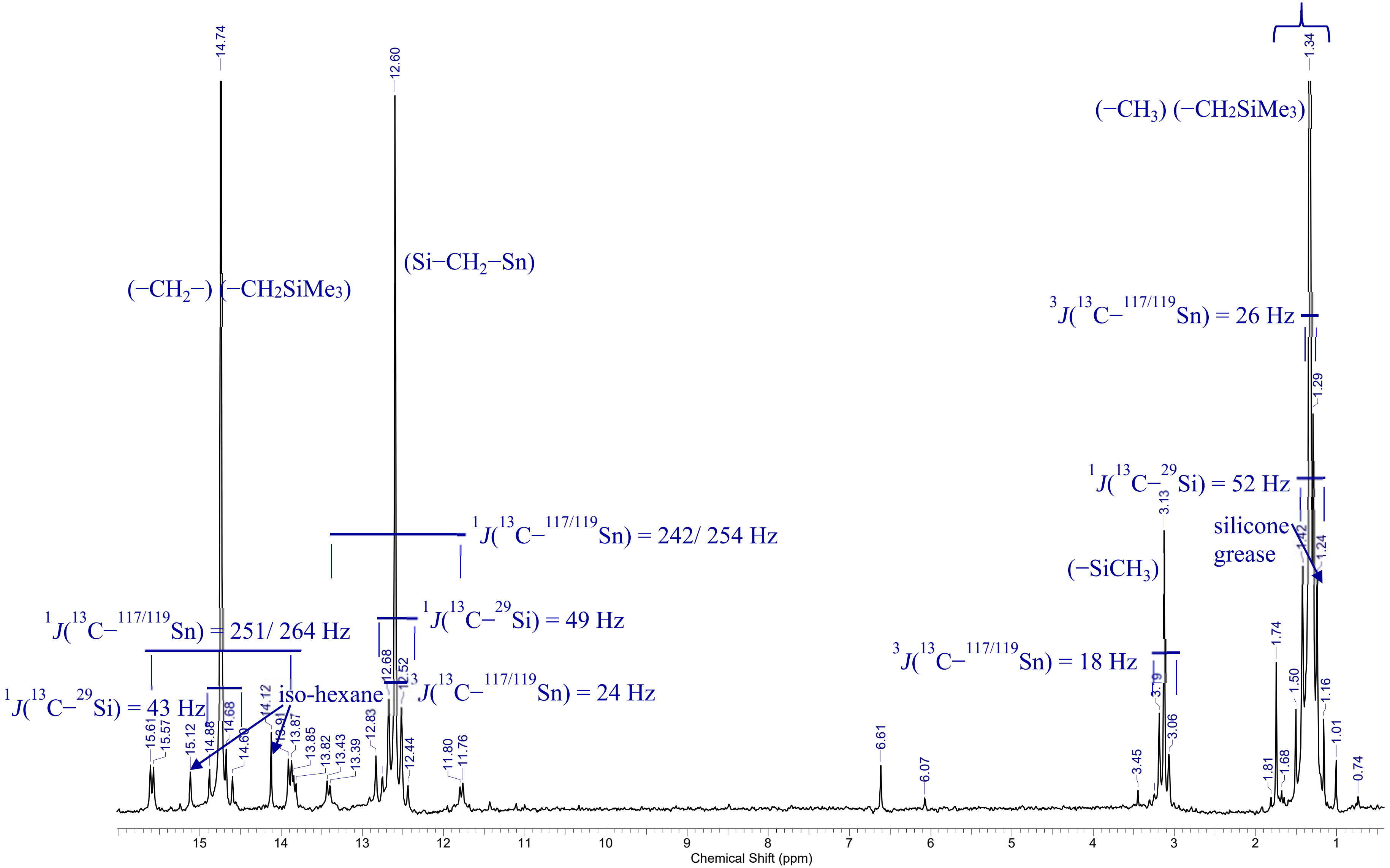

Figure S45. ^{13}C NMR spectrum (150.46 MHz, $CDCl_3$) of compound **6**.

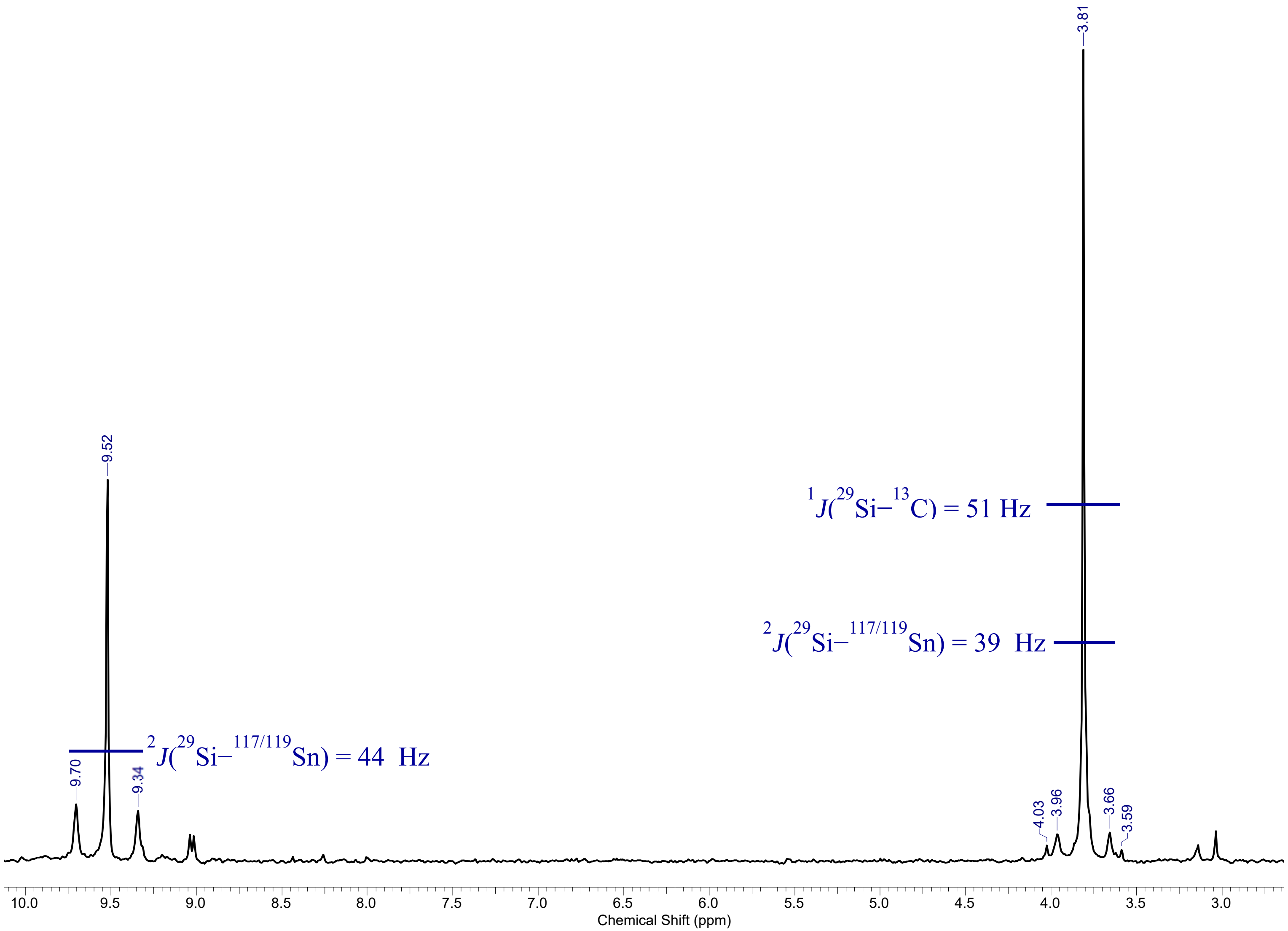

Figure S46. ^{29}Si NMR spectrum (79.52 MHz, $CDCl_3$) of compound **6**.

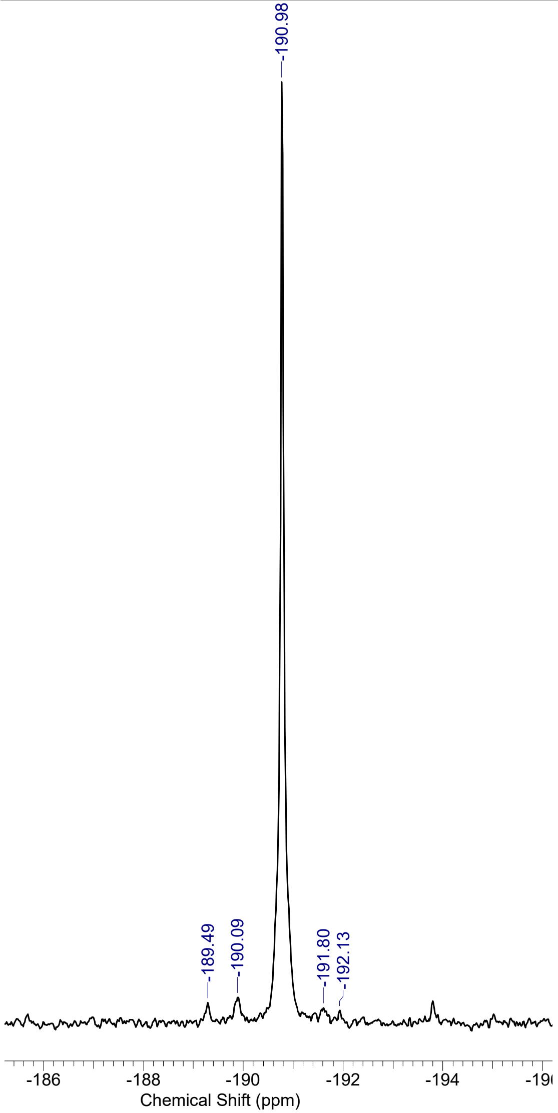

Figure S47. ^{119}Sn NMR spectrum (149.26MHz, $CDCl_3$) of compound **6**.

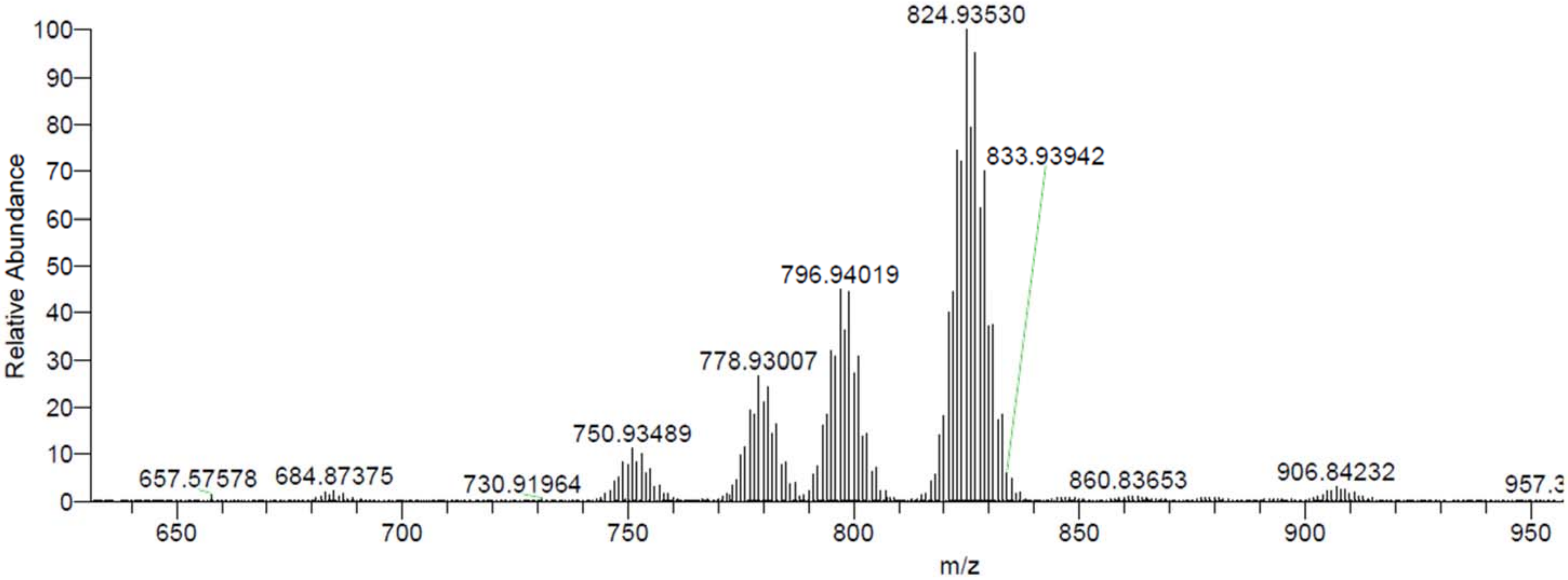

Figure S48. The ESI MS spectrum (positive mode) of **6**. The horizontal axis shows the m/z values.

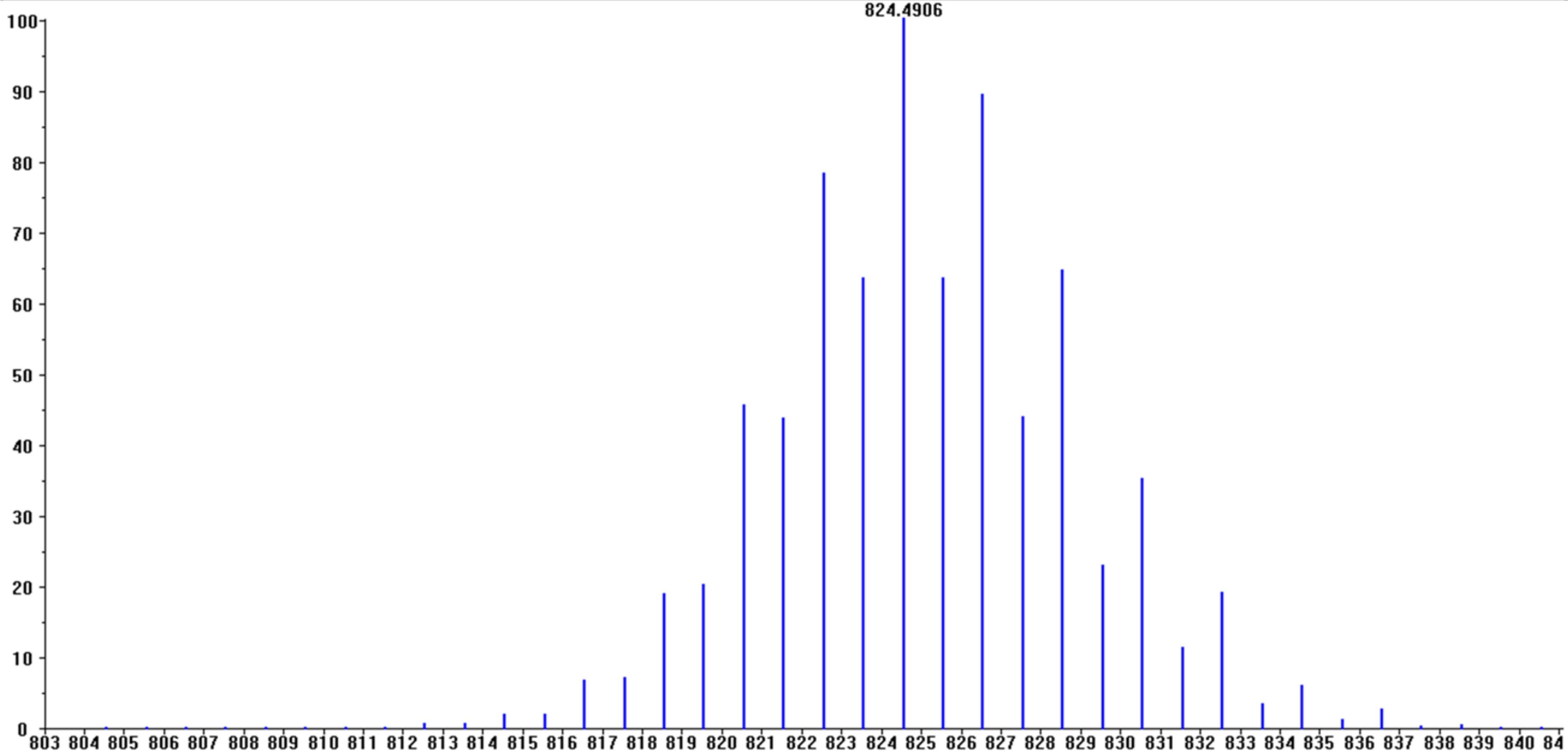

Figure S49. Simulated mass cluster for $C_4H_{12}I_3SiSn_3^+$. The horizontal axis shows the m/z values.

Elementaranalysenauftrag ②

Jibed Aysi.	/ 4537	23.06.2018	AJ273.
Auftraggeber	Telefon	Datum	Probenbezeichnung (max. 7 Stellen)

Die Substanz enthält: $C_{18}H_{42}Cl_2I_6Si_4Sm_3$ ($C_{16}H_{42}I_6Si_4Sm_3$)

Smp.: ______ auf Anruf? ______ luftempfindlich: ☐

Sdp.: ______ hygroskopisch: ☐

Bemerkungen: ______

Einwaage:		theor.	prakt. a	prakt. b
a) 1.653	% C:	13.12 %	13.1	13.1
b) 2.343	% H:	2.89 %	2,9	2,9
	% N:		/	/

Akjunkulat — Arbeitskreisleiter

13.7.18 [illegible] — Datum der Ausführung

Figure S50. Elemental analysis of **6**.

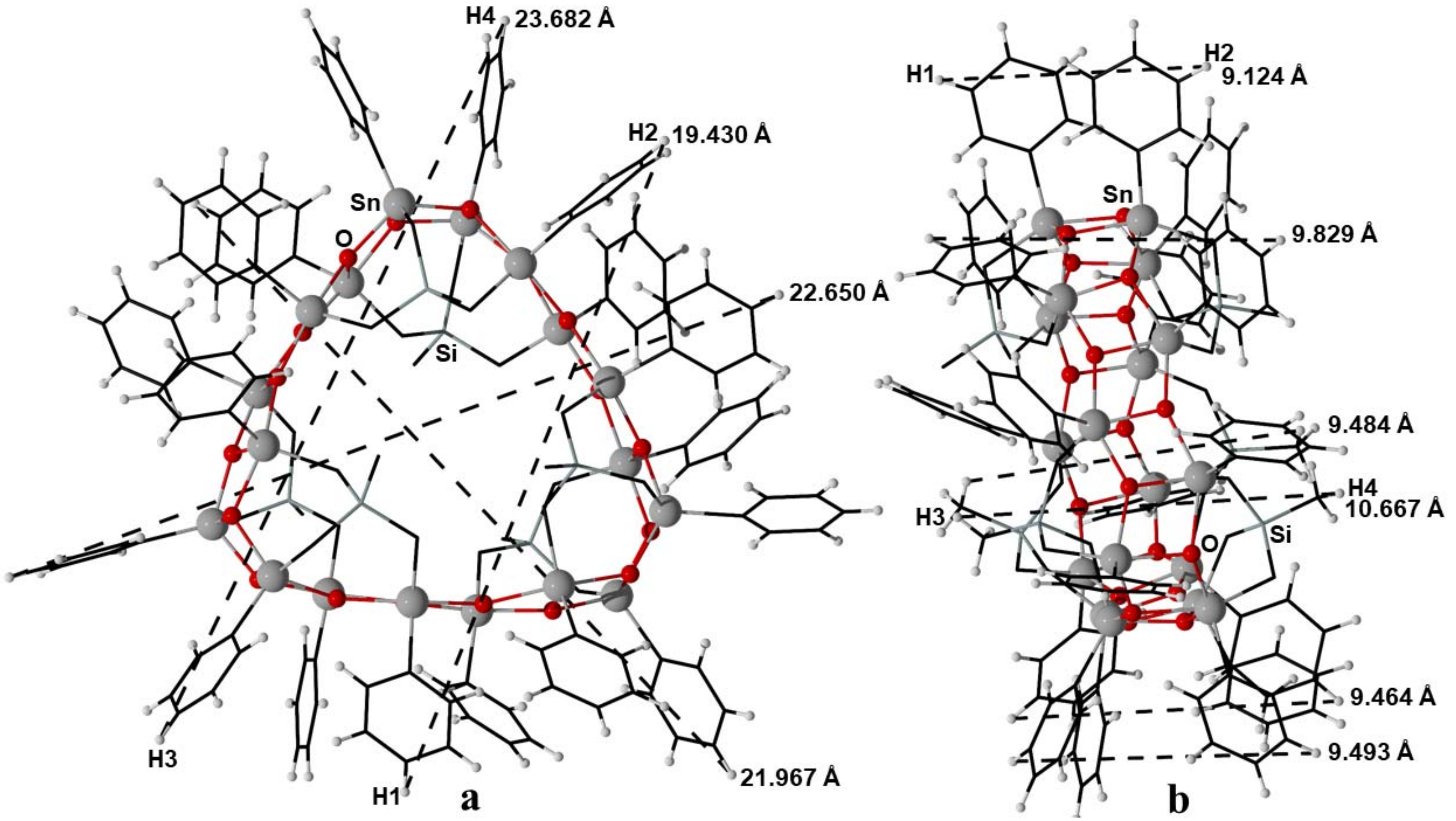

Figure S51. Front view a) and side view b) (POV-Ray) of **7** including the H44···H94 (20.06(1) Å) and H84···H144 (23.00(1) Å) distances and the distances indicative for the thickness (H55···H155 10.47(1) Å, H5···H154 10.97(1) Å).

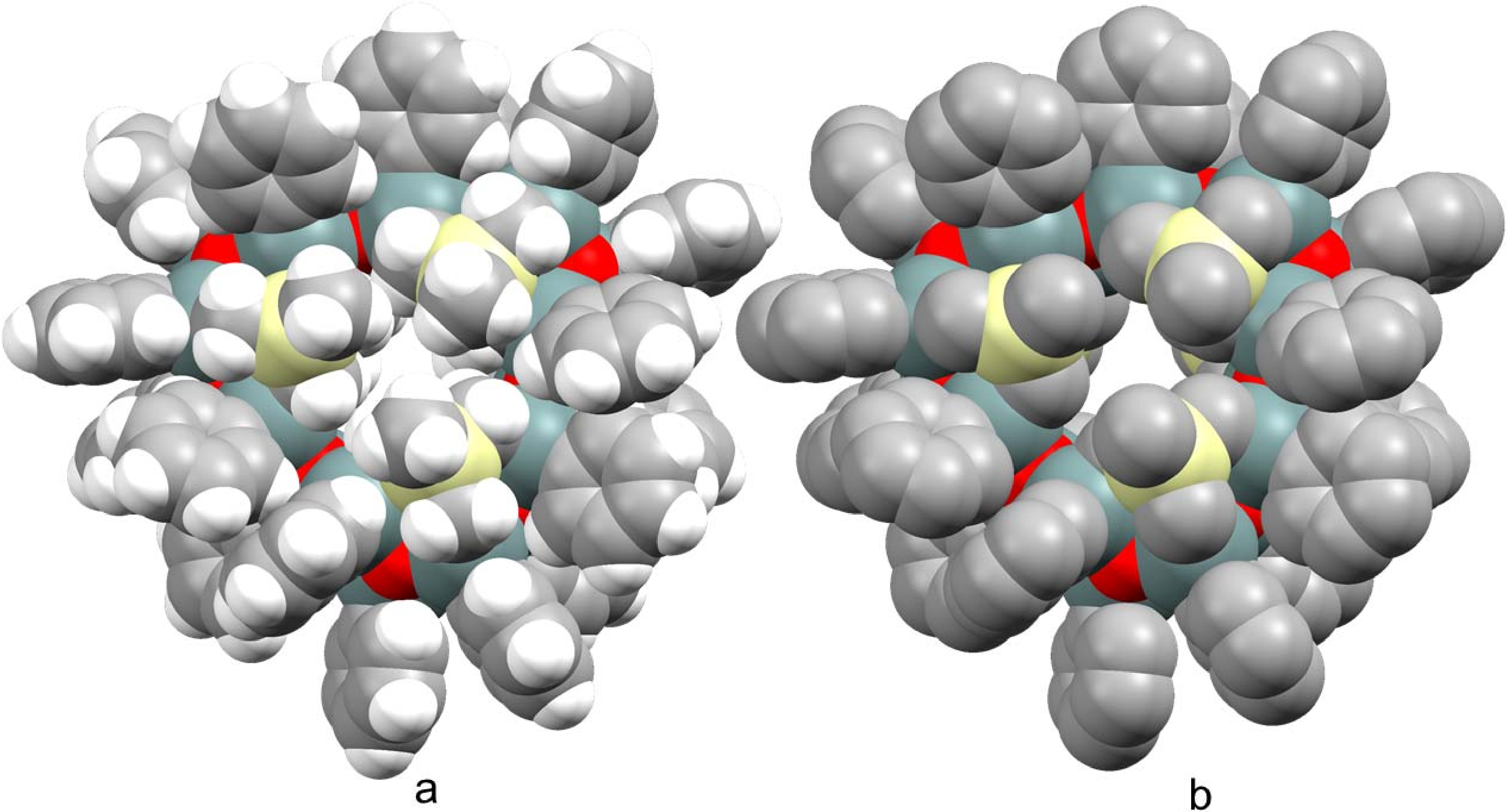

Figure S52. POV Ray images of the macrocycle **7** in space fill mode (left site with hydrogen atoms, right site without hydrogen atoms).

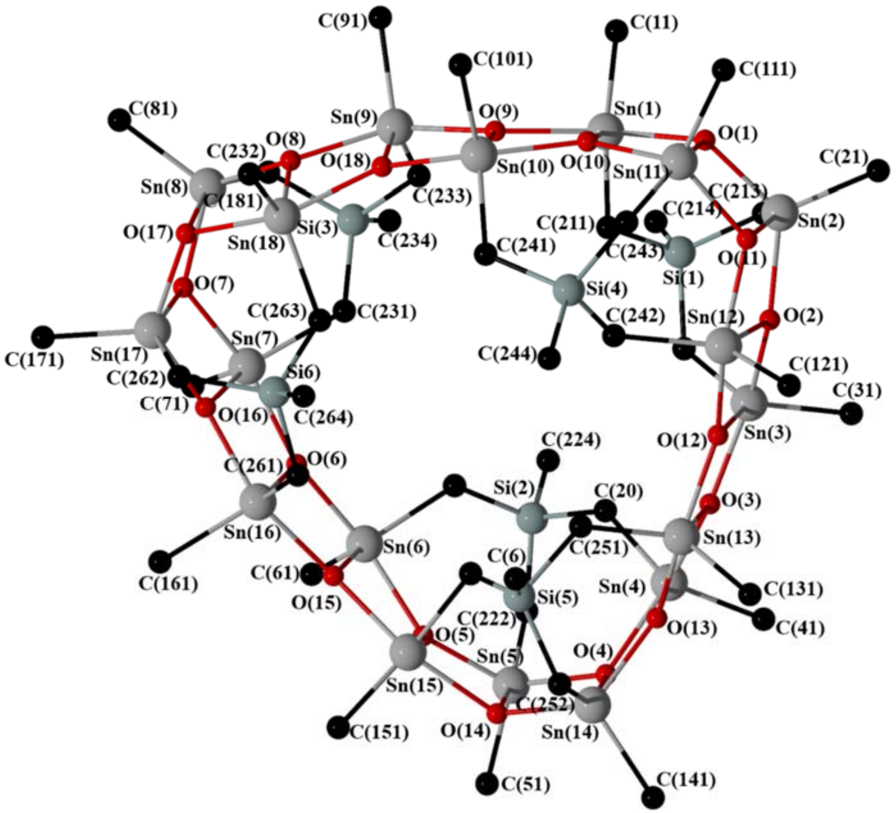

Figure S53. POV-Ray image of the structure of **7**. The subsequent Tables S1 and S2 contain interatomic distances and angles, respectively, involving the tin atoms. Of the phenyl substituents, only the C_{ipso} carbon atoms are shown.

Table S1. Interatomic distances (Å) for compound **7**.

Sn1 – O1	2.114(8)	Sn5 – O4	2.023(8)	Sn9 – O8	2.115(7)	Sn13 – O3	2.030(8)
Sn1 – O9	2.135(8)	Sn5 – O5	2.035(8)	Sn9 – O9	2.094(8)	Sn13 – O12	2.101(7)
Sn1 – O10	2.035(8)	Sn5 – O14	2.184(9)	Sn9 – O18	2.058(8)	Sn13 – O13	2.076(8)
Sn1 – C11	2.117(5)	Sn5 – C51	2.131(6)	Sn9 – C91	2.145(7)	Sn13 – C131	2.135(7)
Sn1 – C211	2.105(12)	Sn5 – C222	2.152(12)	Sn9 – C233	2.096(14)	Sn13 – C251	2.098(16)
Sn2 – O1	2.044(7)	Sn6 – O5	2.141(8)	Sn10 – O9	2.045(8)	Sn14 – O4	2.162(8)
Sn2 – O2	2.075(7)	Sn6 – O6	2.110(7)	Sn10 – O10	2.110(8)	Sn14 – O13	2.087(8)
Sn2 – O11	2.136(8)	Sn6 – O15	2.030(9)	Sn10 – O18	2.077(9)	Sn14 – O14	2.011(9)
Sn2 – C21	2.125(7)	Sn6 – C19	2.087(16)	Sn10 – C101	2.131(7)	Sn14 – C141	2.132(6)
Sn2 – C213	2.131(13)	Sn6 – C707	2.087(8)	Sn10 – C241	2.121(11)	Sn14 – C252	2.149(13)
Sn3 – O2	2.135(8)	Sn7 – O6	2.138(7)	Sn11 – O1	2.165(8)	Sn15 – O5	2.008(10)
Sn3 – O3	2.116(8)	Sn7 – O7	2.124(7)	Sn11 – O10	2.046(9)	Sn15 – O14	2.134(8)
Sn3 – O12	2.038(8)	Sn7 – O16	2.008(9)	Sn11 – O11	2.039(8)	Sn15 – O15	2.124(9)
Sn3 – C31	2.125(6)	Sn7 – C71	2.096(9)	Sn11 – C111	2.126(7)	Sn15 – C1	2.142(19)
Sn3 – C212	2.109(13)	Sn7 – C231	2.153(12)	Sn11 – C242	2.147(12)	Sn15 – C151	2.053(10)
Sn4 – O3	2.150(9)	Sn8 – O7	2.033(7)	Sn12 – O2	2.015(8)	Sn16 – O6	2.051(8)
Sn4 – O4	2.156(7)	Sn8 – O8	2.043(8)	Sn12 – O11	2.123(7)	Sn16 – O15	2.111(8)
Sn4 – O13	2.016(9)	Sn8 – O17	2.198(8)	Sn12 – O12	2.126(7)	Sn16 – O16	2.111(8)
Sn4 – C20	2.16(3)	Sn8 – C81	2.127(7)	Sn12 – C121	2.098(8)	Sn16 – C9	2.061(17)
Sn4 – C41	2.121(8)	Sn8 – C232	2.138(12)	Sn12 – C243	2.113(15)	Sn16 – C161	2.260(17)
		Sn17 – O7	2.141(8)	Sn18 – O8	2.044(8)		
		Sn17 – O16	2.088(7)	Sn18 – O17	2.104(8)		
		Sn17 – O17	2.036(7)	Sn18 – O18	2.146(8)		
		Sn17 – C171	2.140(8)	Sn18 – C181	2.123(7)		
		Sn17 – C262	2.143(13)	Sn18 – C263	2.145(11)		

Table S2. Interatomic angles (°) for compound **7**.

O1–Sn1–O9	149.4(3)	O3–Sn3–O2	149.2(3)	C20–Sn4–C41	136.4(8)	O6–Sn6–O5	150.1(4)
O1–Sn1–C11	98.3(3)	O3–Sn3–C31	100.6(3)			O15–Sn6–O5	74.0(3)
O10–Sn1–O1	75.0(3)	O12–Sn3–O2	73.6(3)	Sn4–O4–Sn14	101.1(3)	O15–Sn6–O6	76.5(3)
O10–Sn1–O9	74.4(3)	O12–Sn3–O3	75.7(3)	Sn5–O4–Sn4	129.4(4)	C19–Sn6–O6	99.8(5)
O10–Sn1–C11	119.2(3)	O12–Sn3–C31	124.8(3)	Sn5–O4–Sn14	104.8(3)	C19–Sn6–C707	107.7(8)
O10–Sn1–C211	113.5(4)	O12–Sn3–C212	123.5(4)			C19–Sn6–C27	142.7(15)
C11–Sn1–O9	97.8(3)	C31–Sn3–O2	97.4(3)	O4–Sn5–O5	125.6(3)		
C211–Sn1–O1	96.5(4)	C212–Sn3–O2	97.8(4)	O4–Sn5–O14	75.0(3)	Sn6–O6–Sn7	153.0(4)
C211–Sn1–O9	94.1(4)	C212–Sn3–O3	98.5(4)	O4–Sn5–C51	116.9(3)	Sn16–O6–Sn6	103.1(3)
C211–Sn1–C11	127.3(4)	C212–Sn3–C31	111.7(4)	O4–Sn5–C222	96.5(4)	Sn16–O6–Sn7	103.9(3)
				O5–Sn5–O14	74.0(3)		
Sn1–O1–Sn11	101.8(3)	Sn3–O3–Sn4	150.6(4)	O5–Sn5–C51	110.7(3)	O6–Sn7–C231	92.9(4)
Sn1–O1–Sn2	127.4(4)	Sn13–O3–Sn3	103.9(4)	O5–Sn5–C222	91.5(4)	O7–Sn7–O6	148.7(3)
		Sn13–O3–Sn4	104.1(3)	C51–Sn5–O14	97.8(4)	O7–Sn7–C231	98.6(4)
				C51–Sn5–C222	108.9(5)	O16–Sn7–O6	74.9(3)
O1–Sn2–O2	127.3(3)	O3–Sn4–O4	147.7(3)	C222–Sn5–O14	152.8(4)	O16–Sn7–O7	74.5(3)
O1–Sn2–O11	76.2(3)	O3–Sn4–C20A	93.8(8)			O16–Sn7–C71	115.2(5)
O1–Sn2–C21	108.8(3)	O4–Sn4–C20A	103.4(8)	Sn5–O5–Sn6	139.6(5)	O16–Sn7–C231	123.0(5)
O1–Sn2–C213	96.0(4)	O13–Sn4–O3	73.0(3)	Sn15–O5–Sn5	110.0(4)	C71–Sn7–O6	98.4(4)
O2–Sn2–O11	73.6(3)	O13–Sn4–O4	75.9(3)	Sn15–O5–Sn6	104.3(3)	C71–Sn7–O7	99.9(4)
O2–Sn2–C21	116.7(3)	O13–Sn4–C41	110.9(4)			C71–Sn7–C231	121.8(6)
O2–Sn2–C213	90.1(4)	O13–Sn4–C20	112.4(8)	O15–Sn6–C19	125.1(6)		
C21–Sn2–O11	96.7(4)	O13–Sn4–C20A	137.6(10)	O15–Sn6–C707	127.0(7)	Sn7–O7–Sn17	102.9(3)
C21–Sn2–C213	112.7(5)	C41–Sn4–O3	99.4(4)	O15–Sn6–C27	91.7(14)	Sn8–O7–Sn7	128.7(4)
C213–Sn2–O11	150.5(4)	C41–Sn4–O4	99.6(3)	C19–Sn6–O5	93.2(5)	Sn8–O7–Sn17	105.0(4)
Sn2–O2–Sn3	139.1(4)	C41–Sn4–C20A	111.0(10)	C707–Sn6–O6	100.3(6)		
Sn2–O1–Sn11	103.2(3)	C20–Sn4–O3	88.6(8)	C27–Sn6–O5	91.0(7)	O7–Sn8–O8	121.9(3)

Sn2–O2–Sn12	108.0(3)	C20–Sn4–O4	95.4(8)	C27–Sn6–O6	94.6(8)	O7–Sn8–O17	75.2(3)
Sn12–O2–Sn3	105.5(3)	O9–Sn10–O10	74.8(3)	Sn12–O11–Sn2	101.9(3)	Sn4–O13–Sn13	107.3(3)
		O9–Sn10–O18	75.5(3			Sn4–O13–Sn14	108.6(4)
O7–Sn8–C81	112.4(3)	O9–Sn10–C101	112.5(3)	O2–Sn12–O11	75.1(3)	Sn13–O13–Sn14	139.0(5)
O7–Sn8–C232	94.7(4)	O9–Sn10–C241	127.2(4)	O2–Sn12–O12	74.2(3)		
O8–Sn8–O17	74.3(3)	O10–Sn10–C101	96.6(4)	O2–Sn12–C121	115.6(4)	O13–Sn14–O4	74.3(3)
O8–Sn8–C81	119.3(3)	O10–Sn10–C241	94.9(4)	O2–Sn12–C243	118.0(5)	O13–Sn14–C141	110.3(3)
O8–Sn8–C232	91.4(4)	O18–Sn10–O10	149.6(3)	O11–Sn12–O12	149.0(3)	O13–Sn14–C252	90.0(4)
C81–Sn8–O17	96.6(3)	O18–Sn10–C101	100.4(4)	C121–Sn12–O11	99.0(3)	O14–Sn14–O4	75.7(3)
C81–Sn8–C232	110.0(4)	O18–Sn10–C241	98.0(4)	C121–Sn12–O12	98.3(4)	O14–Sn14–O13	128.1(3)
C232–Sn8–O17	153.4(4)	C241–Sn10–C101	120.2(5)	C121–Sn12–C243	126.4(5)	O14–Sn14–C252	97.2(5)
				C243–Sn12–O11	96.7(4)	C141–Sn14–O4	100.4(4)
Sn8–O8–Sn9	142.7(4)	Sn1–O10–Sn10	105.5(4)	C243–Sn12–O12	93.4(4)	C141–Sn14–C252	107.2(5)
Sn8–O8–Sn18	107.8(3)	Sn1–O10–Sn11	108.9(4)			C252–Sn14–O4	151.7(4)
Sn18–O8–Sn9	105.2(4)	Sn11–O10–Sn10	142.3(4)	Sn3–O12–Sn12	105.1(3)		
				Sn3–O12–Sn13	104.2(4)	Sn14–O14–Sn5	104.4(3)
O8–Sn9–C91	100.3(3)	O10–Sn11–O1	73.7(3)	Sn13–O12–Sn12	149.5(4)	Sn14–O14–Sn15	126.7(4)
O9–Sn9–O8	148.4(3)	O10–Sn11–C111	111.7(3)			Sn15–O14–Sn5	100.1(4)
O9–Sn9–C91	101.1(3)	O10–Sn11–C242	91.4(4)	O3–Sn13–O12	76.2(3)		
O9–Sn9–C233	96.9(4)	O11–Sn11–O1	75.7(3)	O3–Sn13–O13	74.3(3)	O5–Sn15–O14	75.6(3)
O18–Sn9–O8	75.0(3)	O11–Sn11–O10	125.3(3)	O3–Sn13–C131	122.1(4)	O5–Sn15–O15	74.9(3)
O18–Sn9–O9	74.9(3)	O11–Sn11–C111	116.0(4)	O3–Sn13–C251	124.1(6)	O5–Sn15–C151	118.1(5)
O18–Sn9–C91	115.7(4)	O11–Sn11–C242	94.8(4)	O12–Sn13–C131	100.8(4)	O5–Sn15–C1	103.8(6)
O18–Sn9–C233	131.3(4)	C111–Sn11–O1	96.7(3)	O13–Sn13–O12	150.4(3)	O5–Sn15–C2	105.3(14)
C233–Sn9–O8	95.9(4)	C111–Sn11–C242	111.5(4)	O13–Sn13–C131	96.4(4)	O5–Sn15–C1A	146.5(11)
C233–Sn9–C91	112.9(5)	C242–Sn11–O1	151.5(4)	O13–Sn13–C251	95.2(5)	O14–Sn15–C1	92.2(6)
Sn9–O18–Sn10	104.2(3)			C251–Sn13–O12	99.6(5)	O14–Sn15–C2	85.3(9)
Sn9–O9–Sn1	151.4(4)	Sn11–O11–Sn2	104.4(3)	C251–Sn13–C131	113.5(5)	O14–Sn15–C1A	98.6(11)

Sn10–O9–Sn1	104.3(4)	Sn11–O11–Sn12	126.2(4)			O15–Sn15–O14	150.1(3)
Sn10–O9–Sn9	104.0(3)	Sn7–O16–Sn17	109.1(3)	Sn9–O18–Sn18	103.6(4)	O8–Sn18–O17	76.3(3)
		Sn17–O16–Sn16	140.4(5)	Sn10–O18–Sn18	150.5(4)	O8–Sn18–O18	74.6(3)
O15–Sn15–C1	90.1(6)					O17–Sn18–O18	150.9(3)
O15–Sn15–C2	107.1(8)	O16–Sn17–O7	72.5(3)	O6 –Sn16–O15	76.0(3)	O8–Sn18–C181	125.0(3)
O15–Sn15–C1A	103.3(11)	O17–Sn17–O7	76.4(3)	O6–Sn16–O16	74.6(3)	O17–Sn18–C181	101.6(3)
C151–Sn15–O14	101.2(4)	O17–Sn17–O16	128.2(3)	O15–Sn16–O16	150.6(3)	C181–Sn18–O18	93.7(3)
C151–Sn15–O15	96.9(5)	O16–Sn17–C171	111.8(3)	O6–Sn16–C9	122.2(8)	O8–Sn18–C263	111.3(4)
C151–Sn15–C1	137.9(7)	O17–Sn17–C171	113.9(3)	C9–Sn16–O15	98.6(7)	O17–Sn18–C263	95.5(4)
C1A–Sn15–C2	107.0(17)	C171–Sn17–O7	100.4(4)	C9–Sn16–O16	98.6(7)	C263–Sn18–O18	96.4(4)
		O7–Sn17–C262	149.9(4)	C9–Sn16–C261	114.0(8)	C181–Sn18–C263	123.5(5)
Sn6–O15–Sn15	104.1(3)	O16–Sn17–C262	91.5(4)	O6–Sn16–C161	108.2(7)		
Sn6–O15–Sn16	103.8(4)	O17–Sn17–C262	95.5(4)	O15–Sn16–C161	100.3(7)	Sn18–O17–Sn8	100.2(3)
Sn16–O15–Sn15	149.8(5)	C171–Sn17–C262	109.2(4)	O16–Sn16–C161	90.8(6)		
				O6–Sn16–C261	123.8(4)		
		Sn17–O17–Sn8	102.8(3)	C261–Sn16–O15	98.7(5)		
		Sn17–O17–Sn18	122.4(4)	C261–Sn16–O16	95.9(4)		
				C261–Sn16–C161	127.5(7)		
				Sn7–O16–Sn16	106.4(3)		

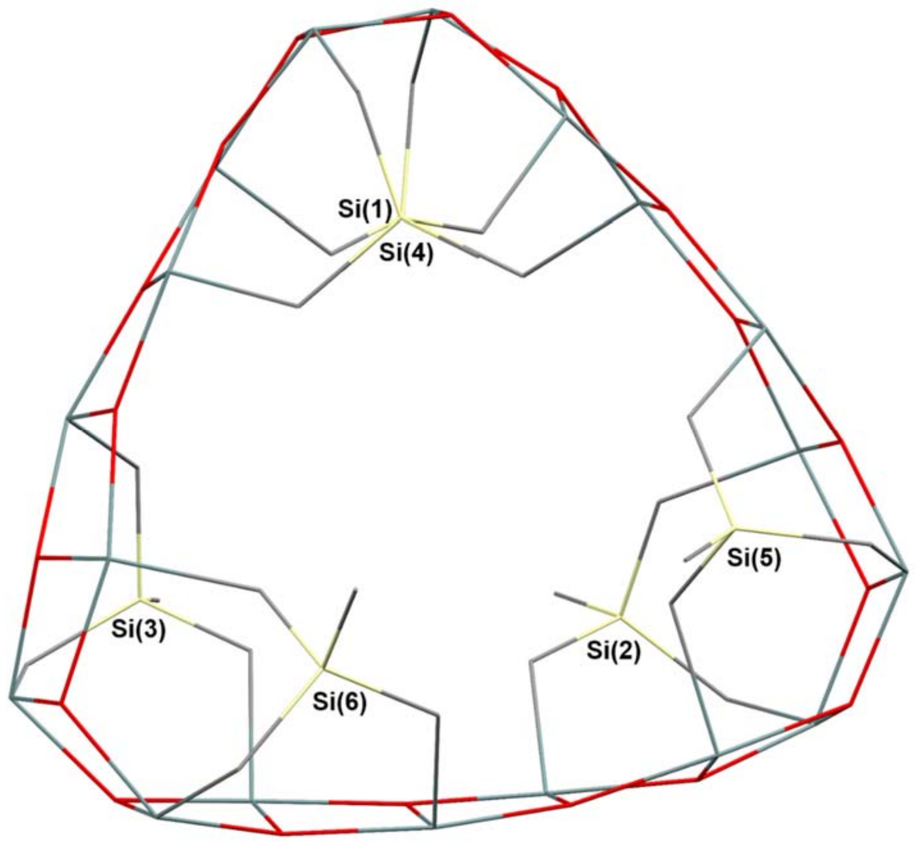

Figure S54. Simplified structure of **7** showing the non-equivalence of the $SiCH_3$ moieties.

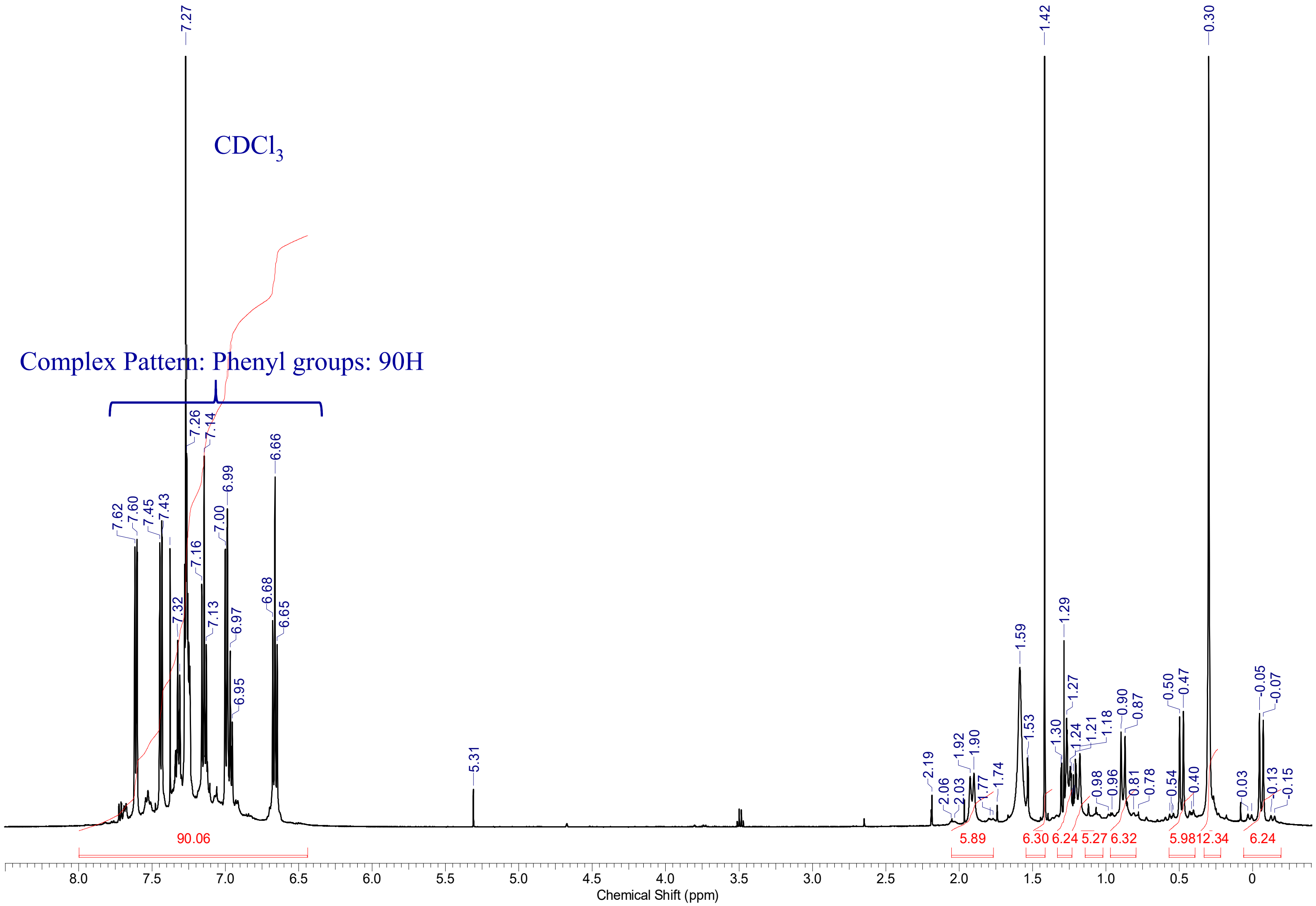

Figure S55. ^{1}H NMR spectrum (500.08 MHz, $CDCl_3$) of compound **7**.

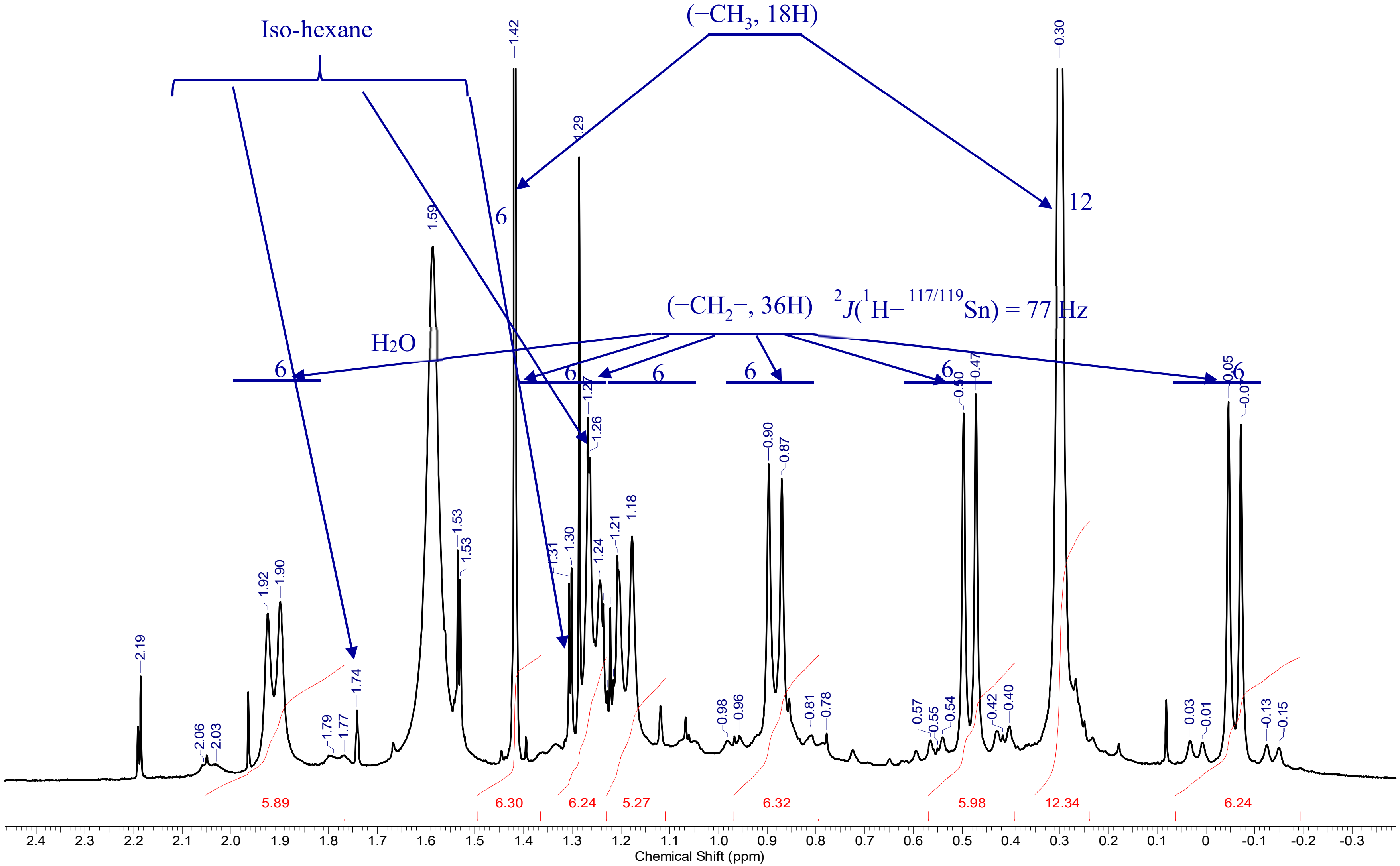

Figure S56. ^{1}H NMR spectrum (500.08 MHz, $CDCl_3$) of compound **7**: aliphatic part.

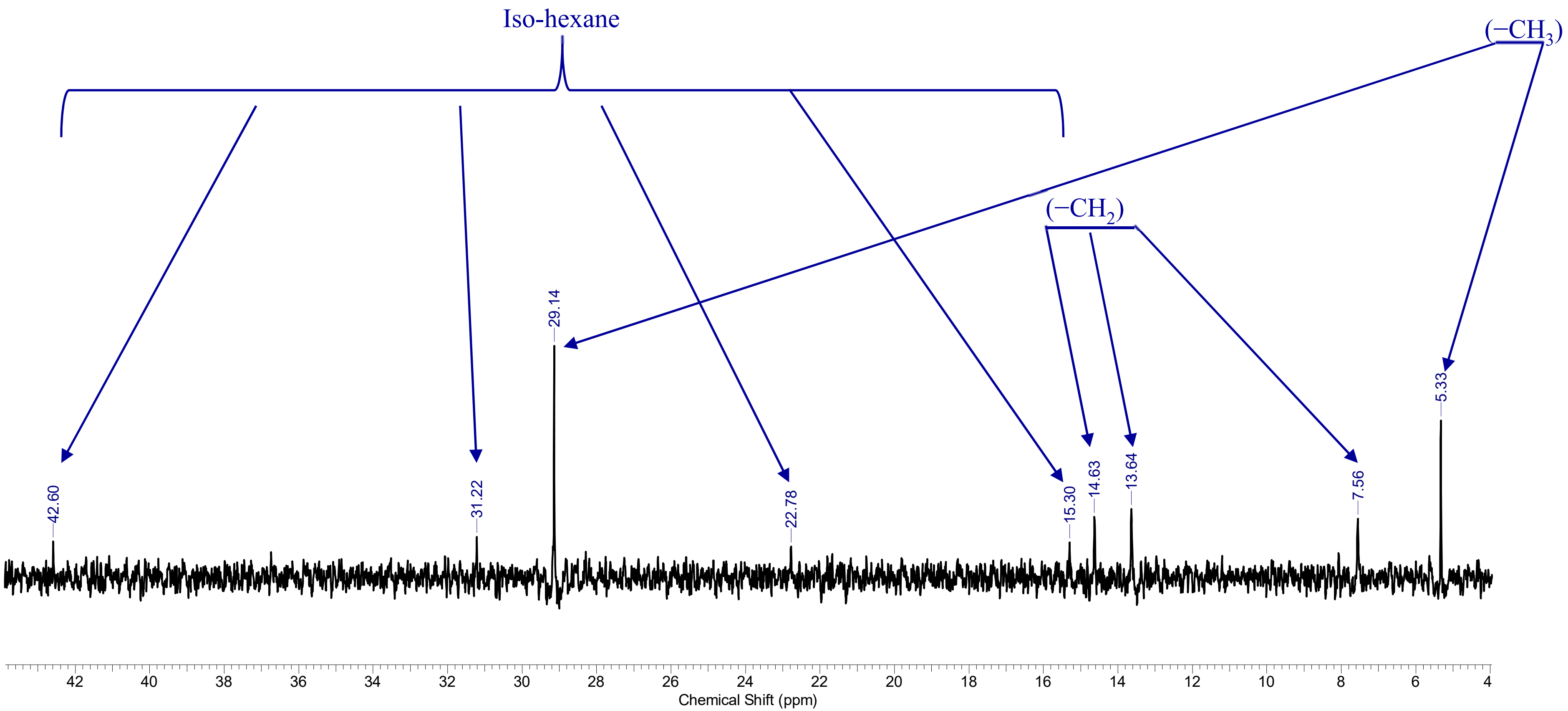

Figure S57. ^{13}C NMR spectrum (125.75 MHz, C_2DCl_2) of compound **7** (aliphatic part).

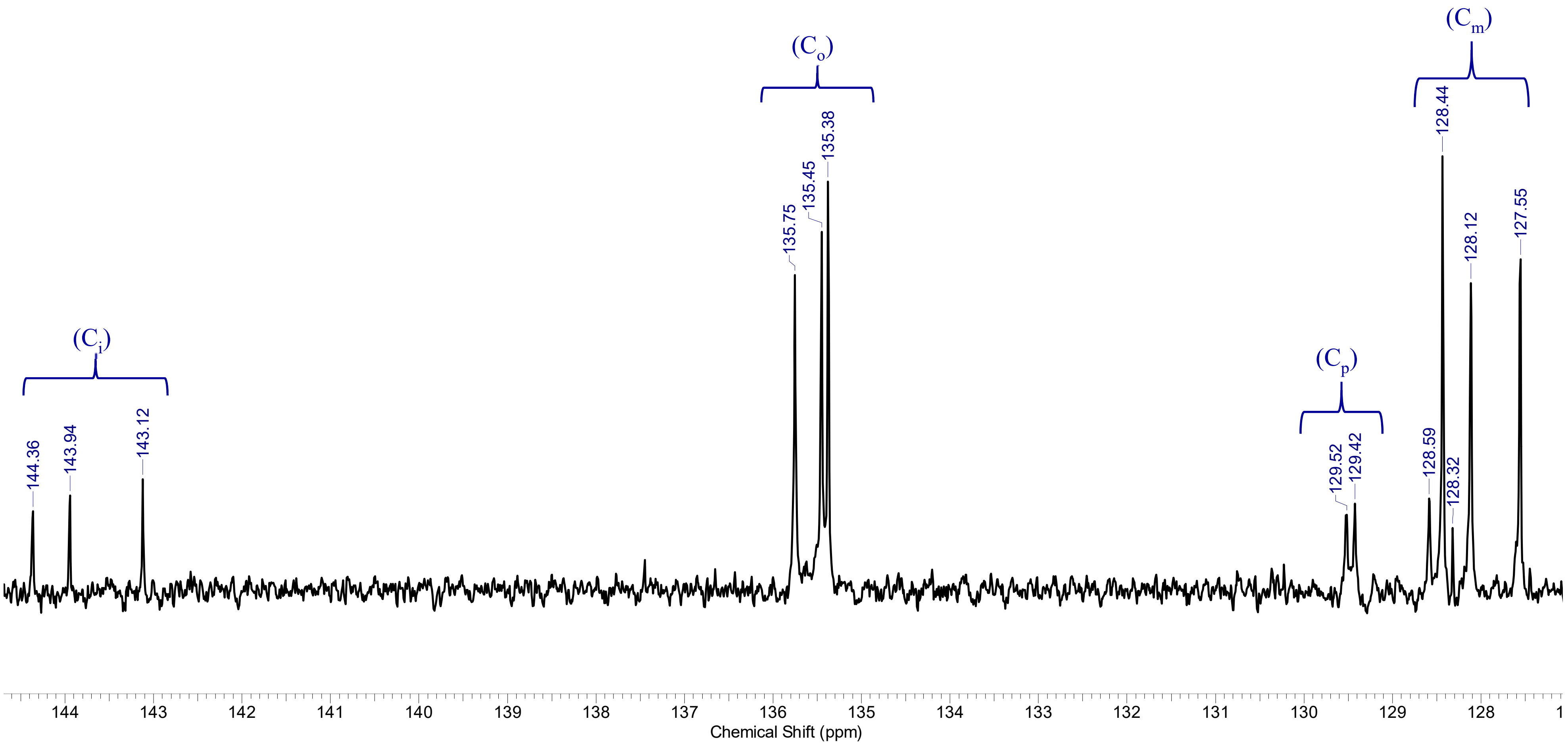

Figure S58. ^{13}C NMR spectrum (125.75 MHz, C_2DCl_2) of compound **7** **(**aliphatic part).

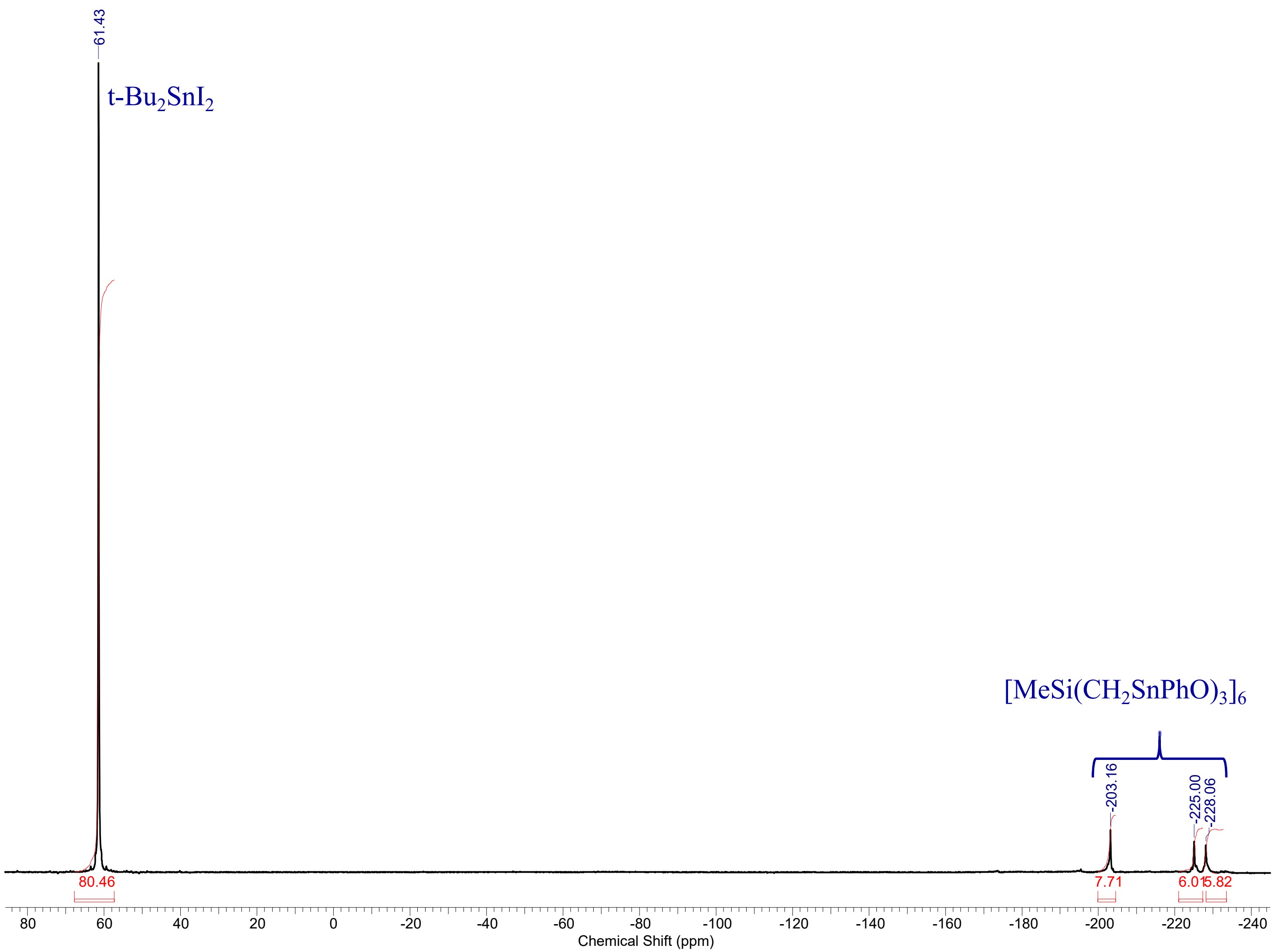

Figure S59. ^{119}Sn NMR spectrum (223.85 MHz, C_6D_6) of the crude reaction mixture obtained from the reaction between $MeSi(CH_2SnPhI_2)_3$ and (t-$Bu_2SnO)_3$ giving compound **7** and t-Bu_2SnI_2.

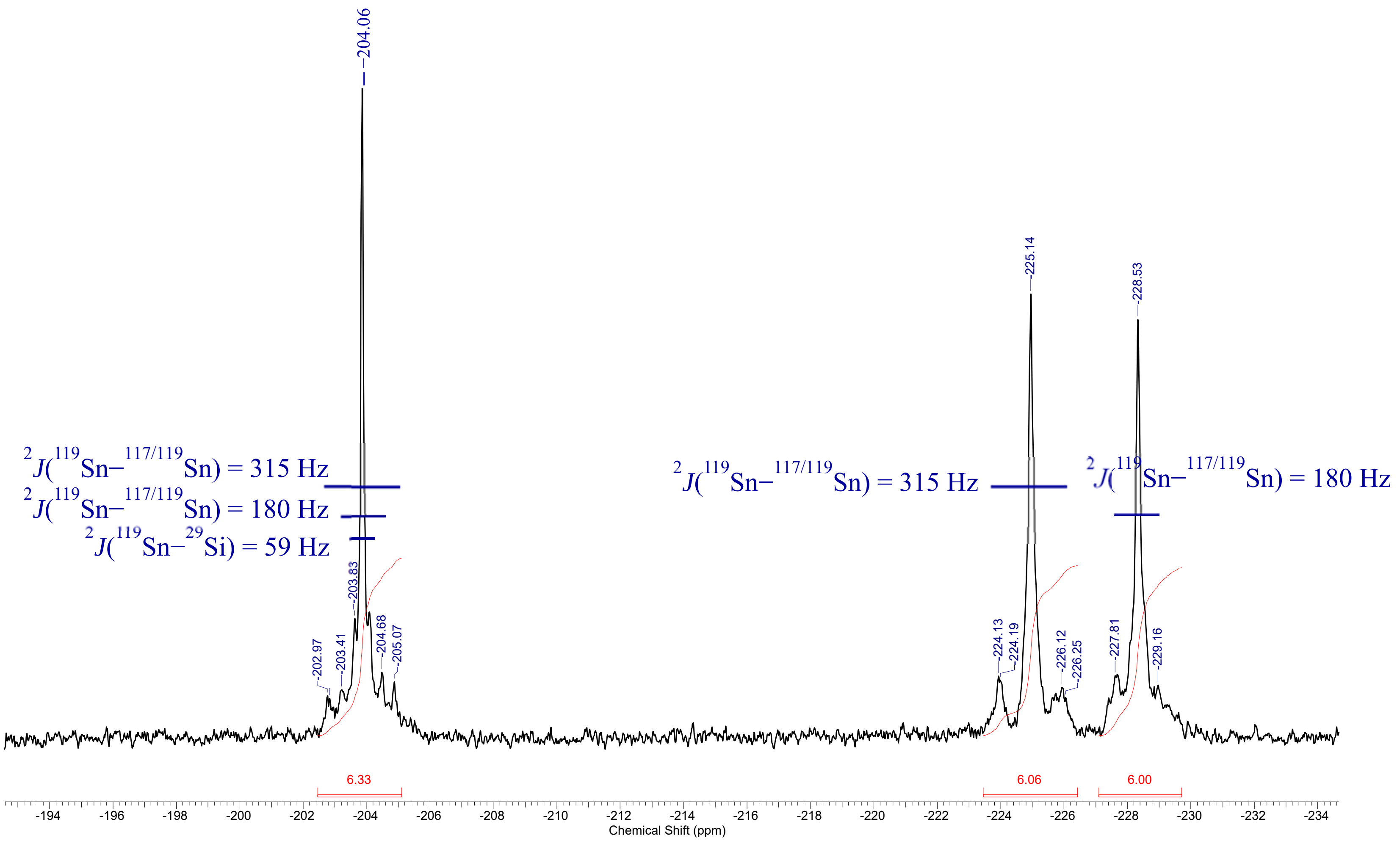

Figure S60. ^{119}Sn NMR spectrum (149.26 MHz) of a solution of compound **7** in $CDCl_3$.

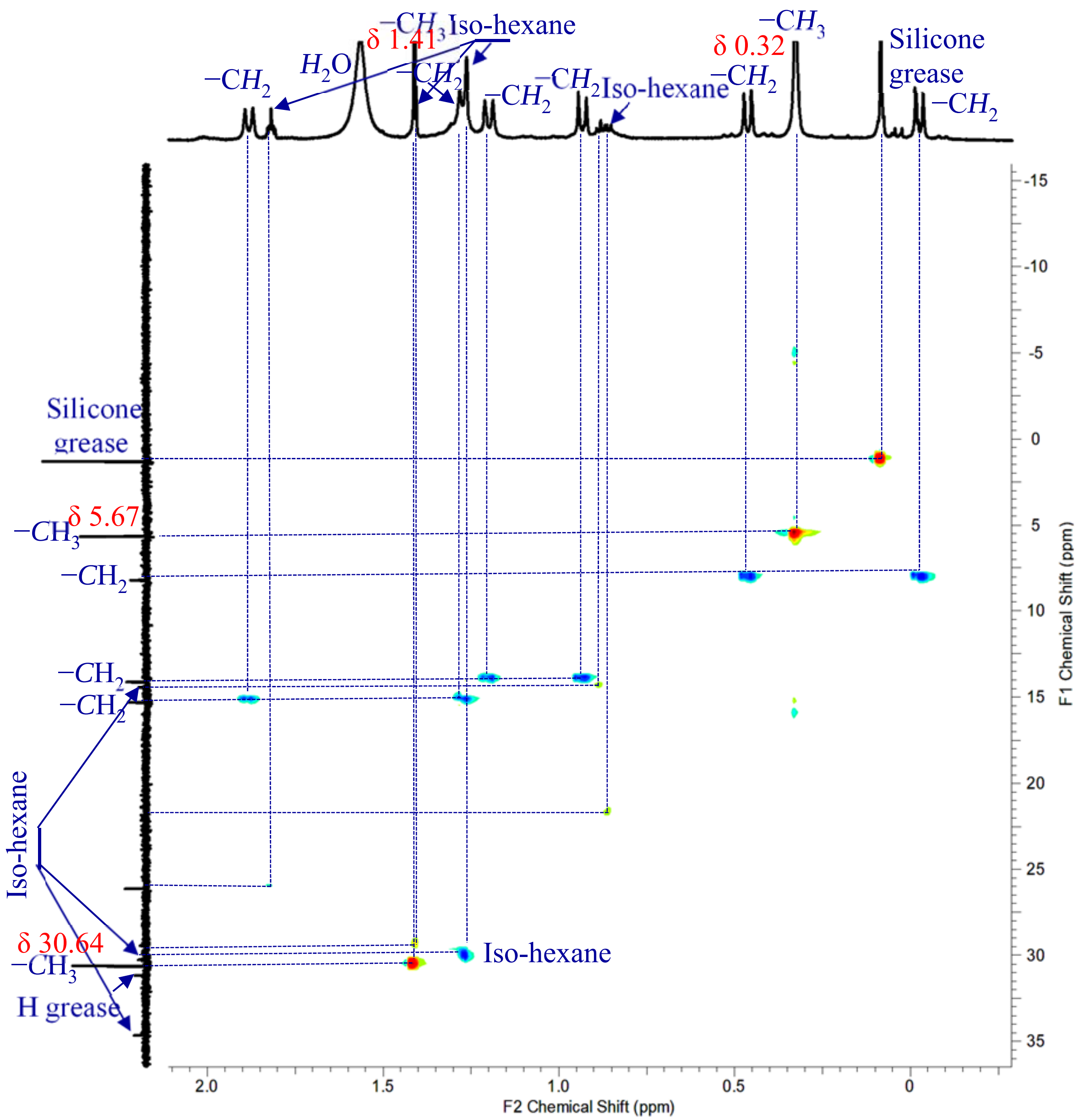

FigureS61. HSQC 2D NMR spectrum (aliphatic part) of **7**.

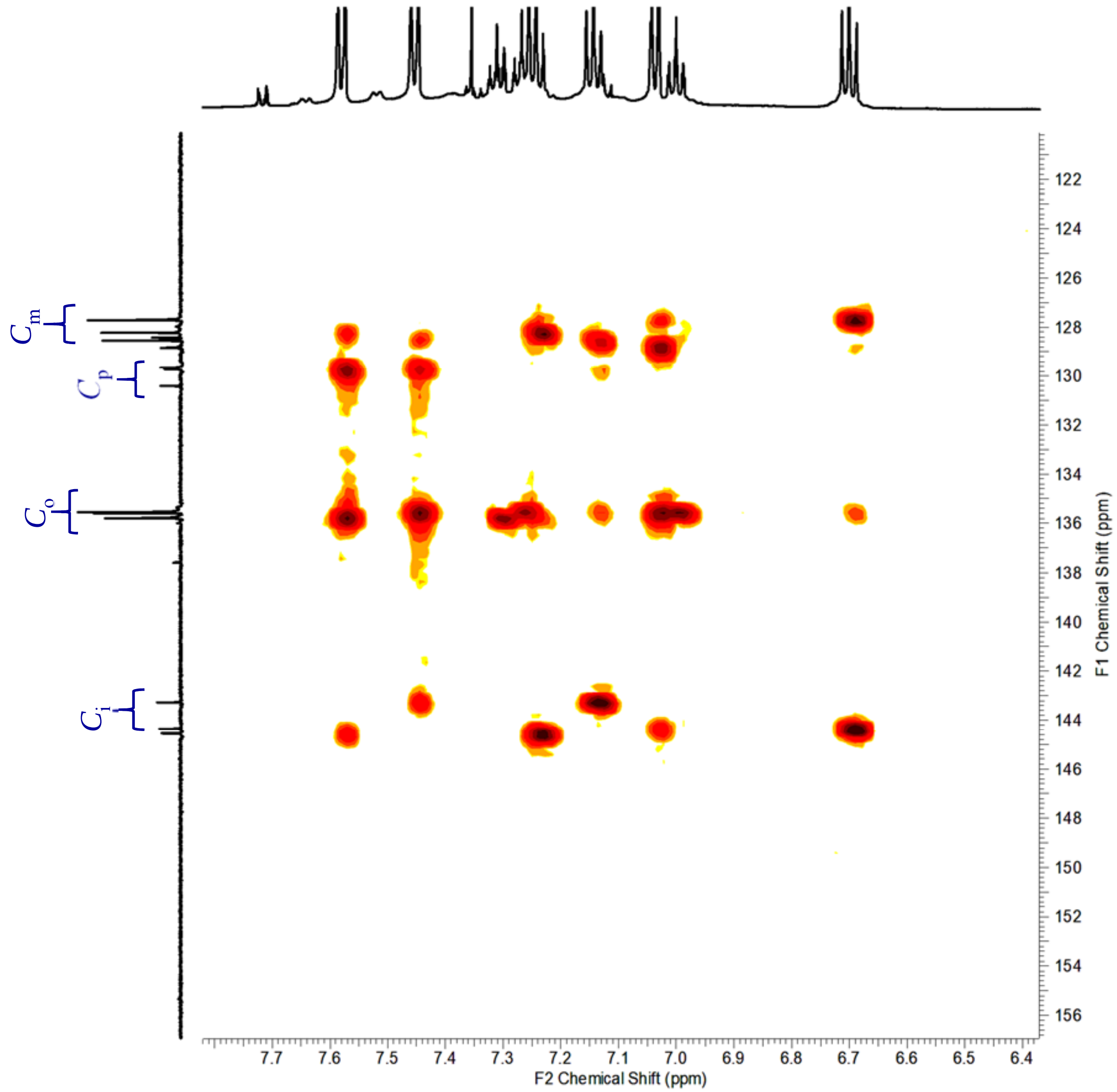

Figure S62. HSQC 2D NMR spectrum (aromatic part) of compound **7.**

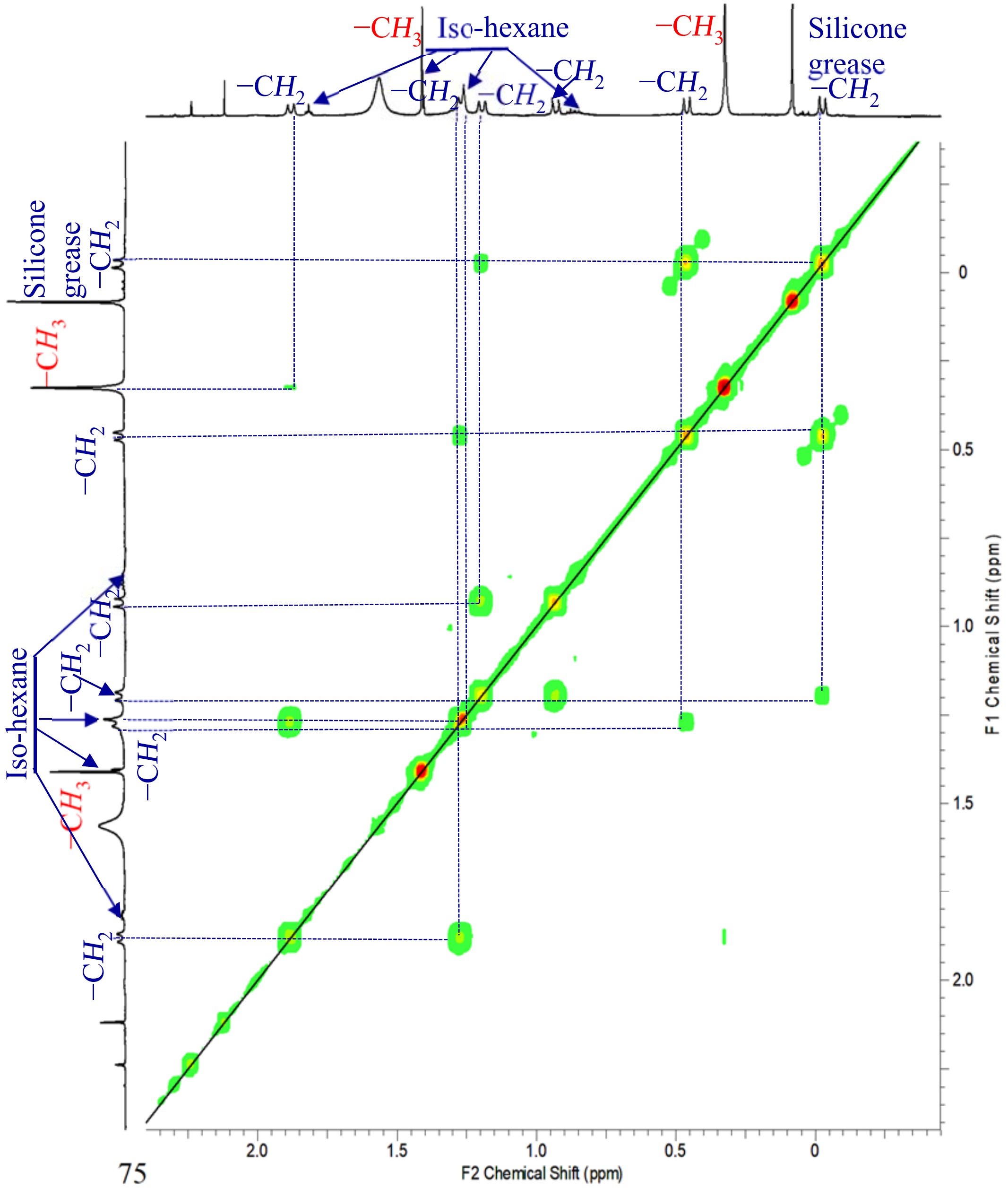

Figure S63. COSY 2D NMR spectrum (aliphatic part) of **7**.

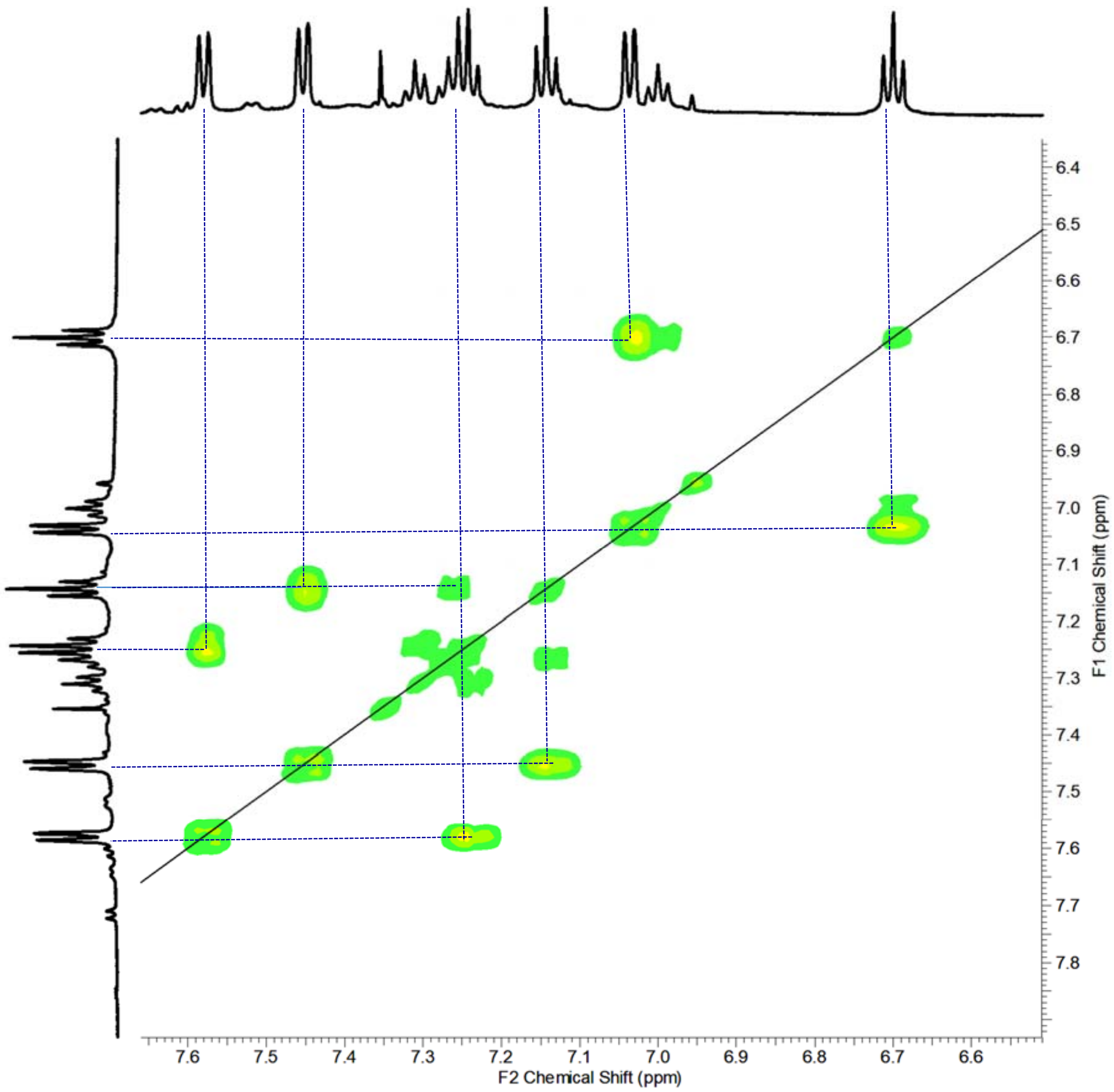

Figure S64. COSY 2D NMR spectrum (aromatic part) of compound **7.**

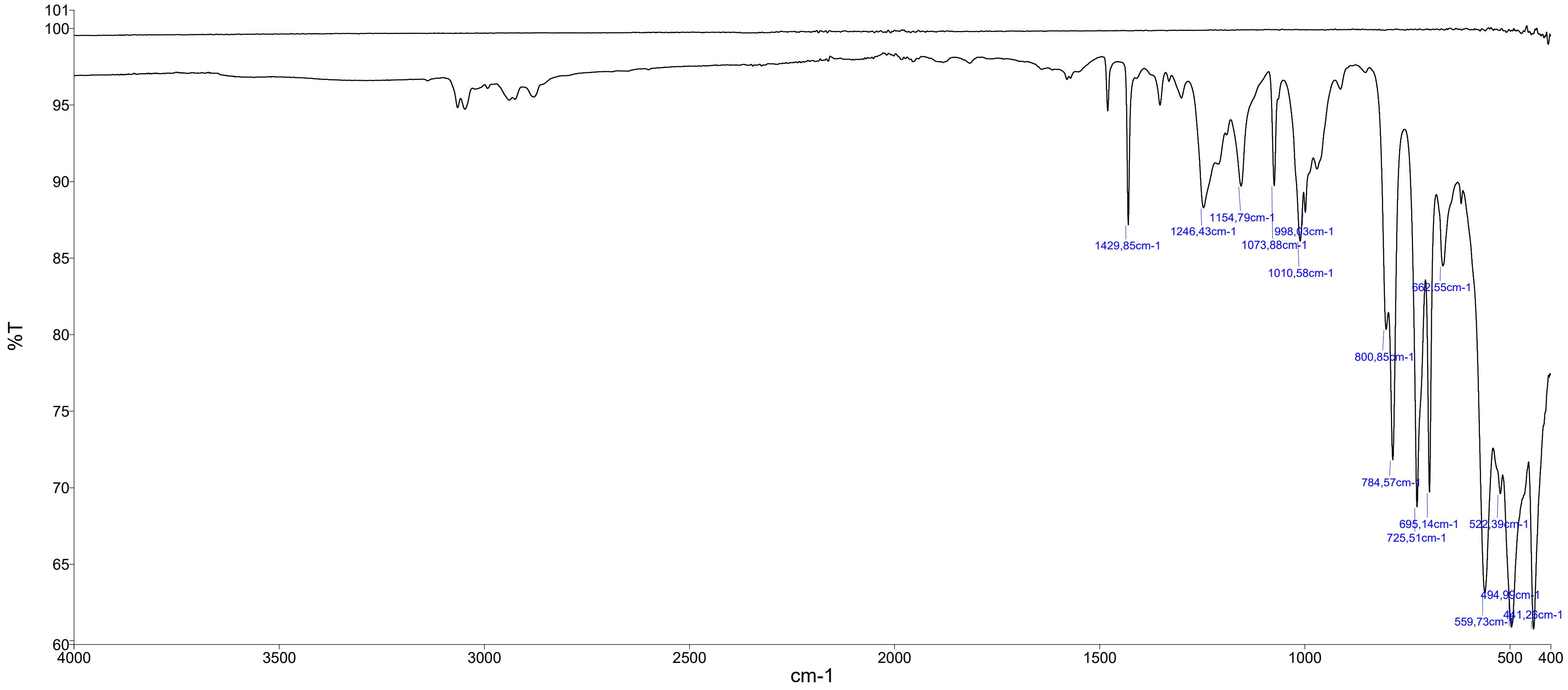

Figure S65. IR spectrum (ATR) of $[MeSi(CH_2SnPhO)_3]_6$, **7**, revealing the absence of SnOH.

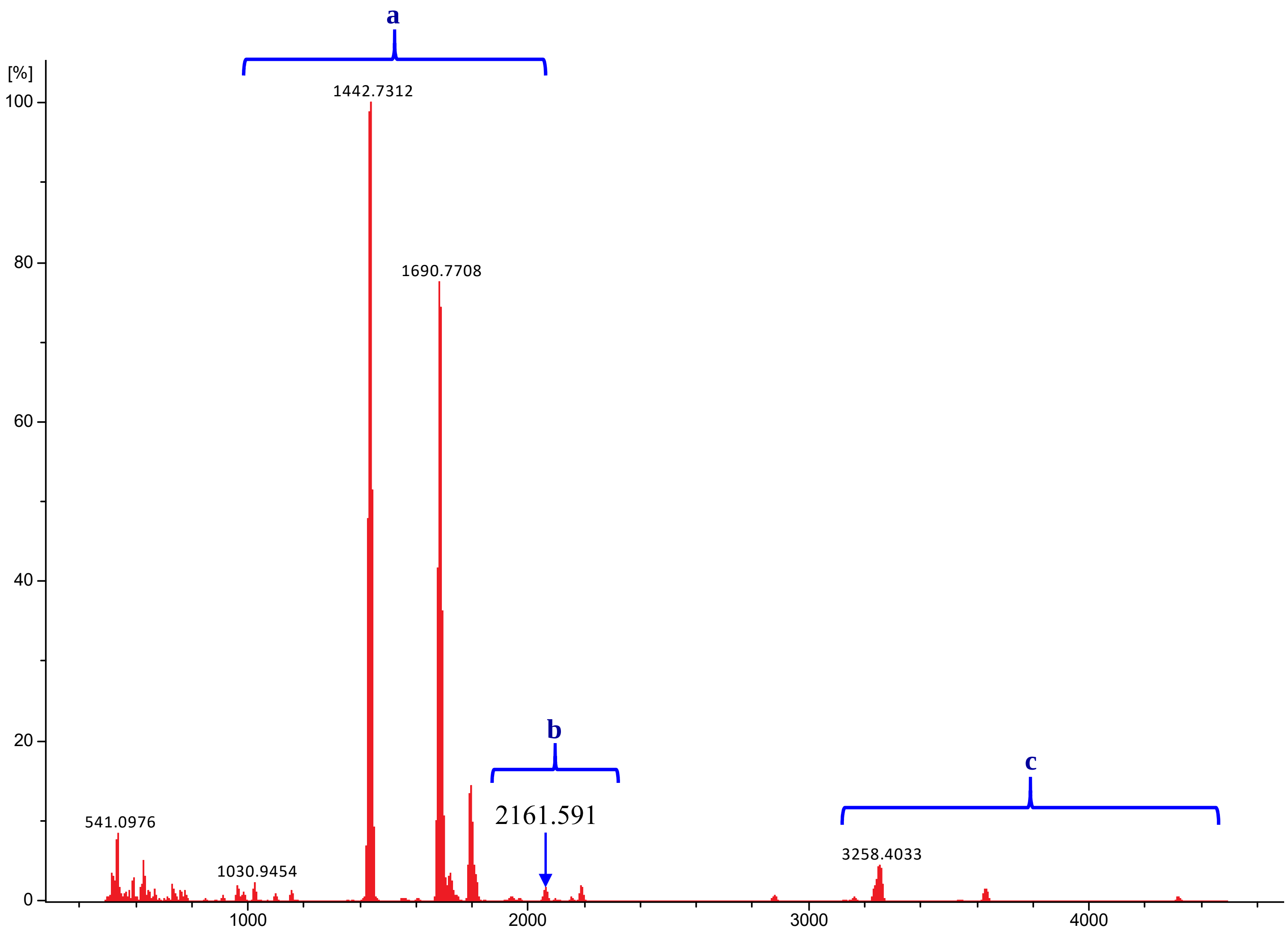

Figure S66. ESI MS spectrum (positive mode) of **7**. The horizontal axis shows the m/z values. The letter a, b, c, and the arrow refer to the subsequent figures showing these areas in detail.

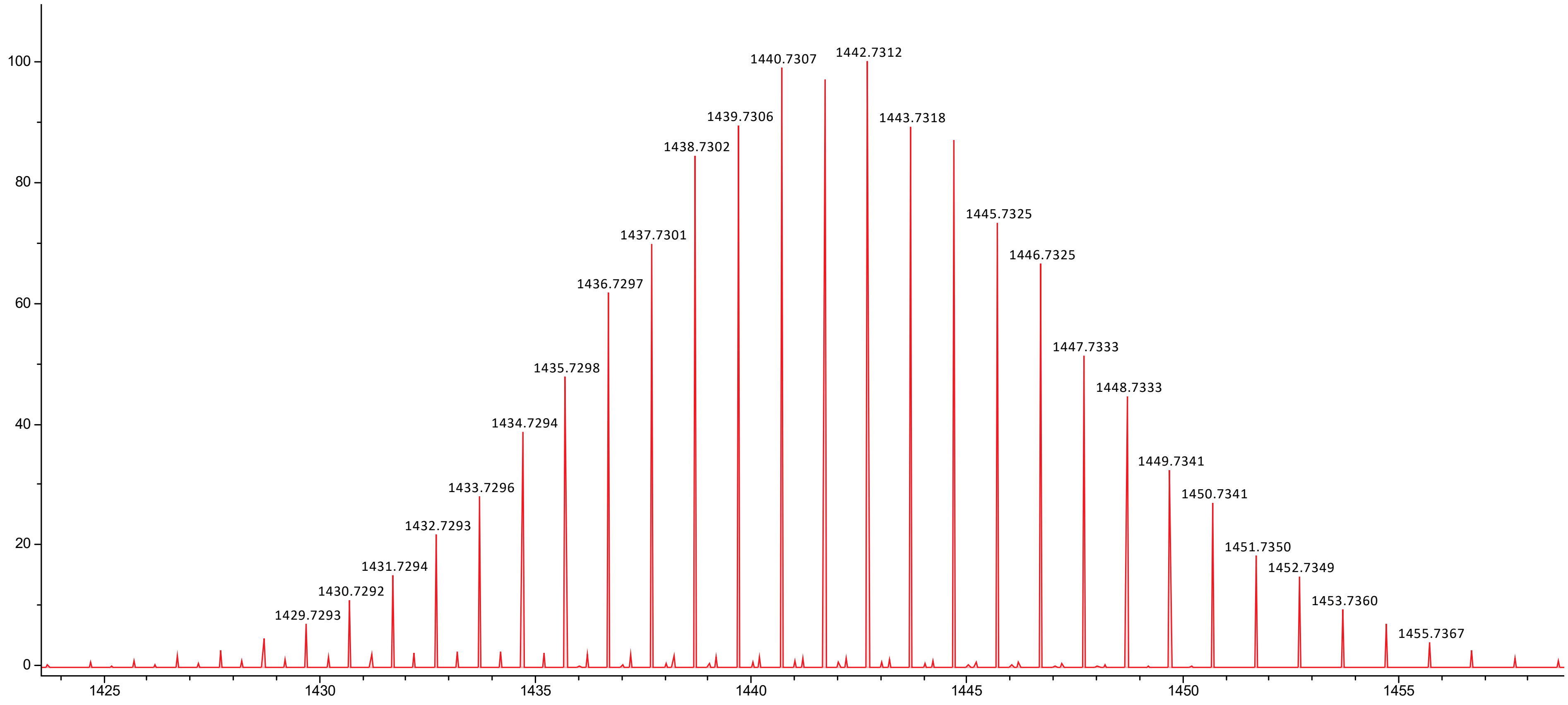

Figure S67. A fraction (a, from Figure S66) of the ESI MS spectrum (positive mode) of **7**: mass cluster at m/z 1442.7312. The horizontal axis shows the m/z values.

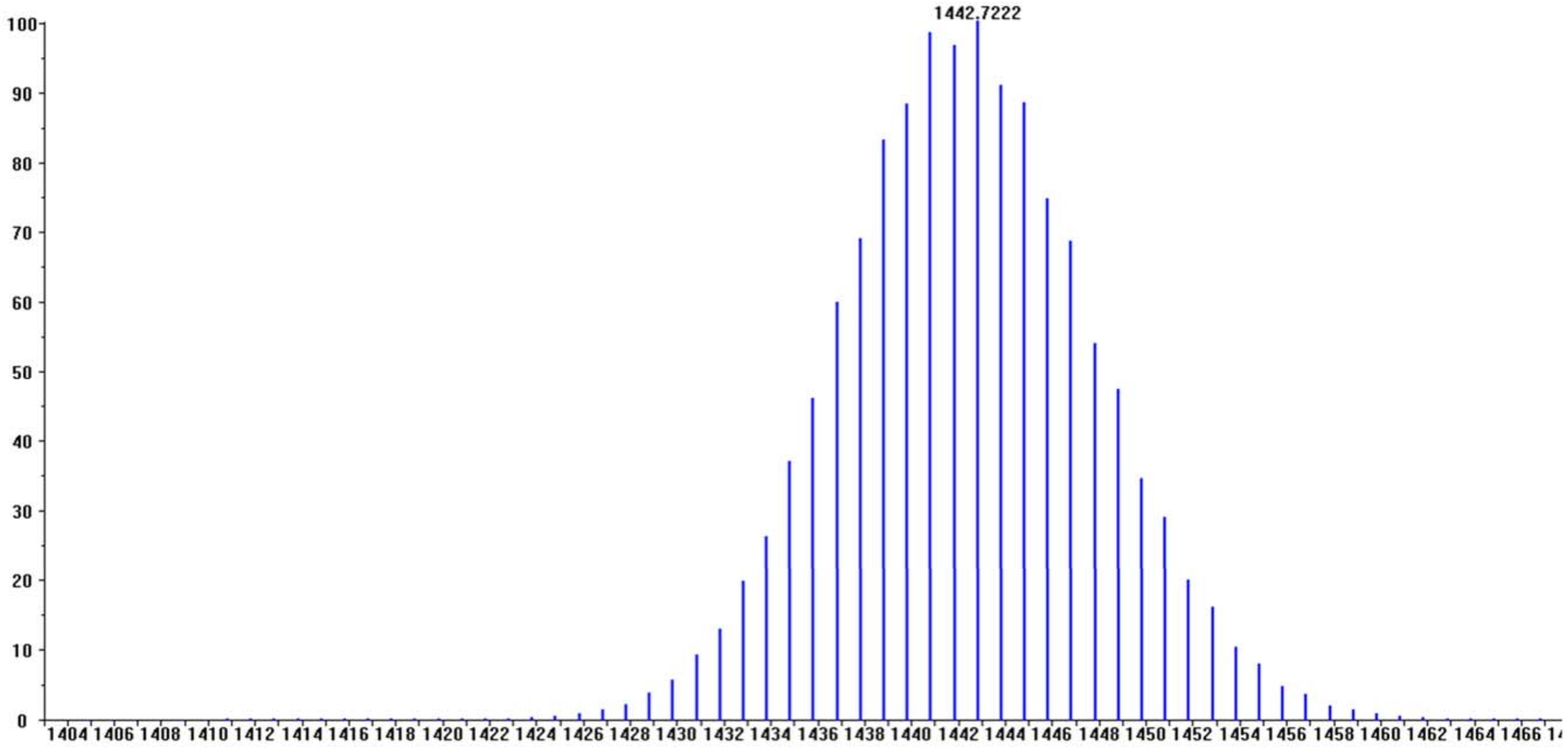

Figure S68. Simulated mass cluster for $C_{44}H_{49}O_6Si_2Sn_6^+$: {[MeSi(CH$_2$SnPhO)$_3$]$_2$ + H$^+$}$^+$. The horizontal axis shows the m/z values.

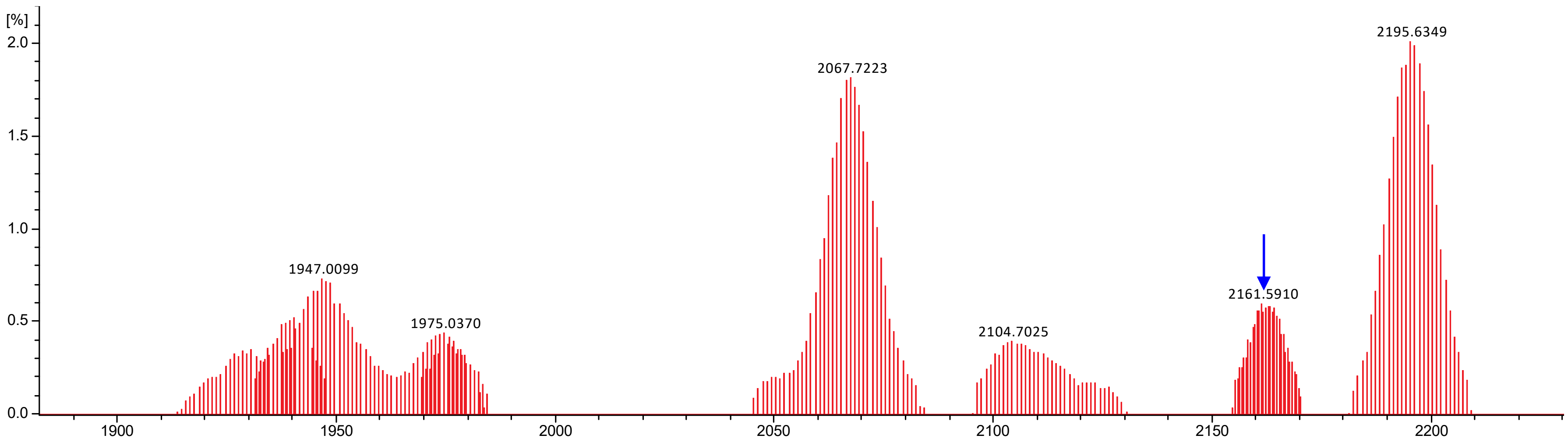

Figure S69. A fraction (b from Figure S66) of the ESI MS spectrum of **7**: mass cluster at m/z 2161.5910. The horizontal axis shows the m/z values. The blue arrow refers to the subsequent Figure S70.

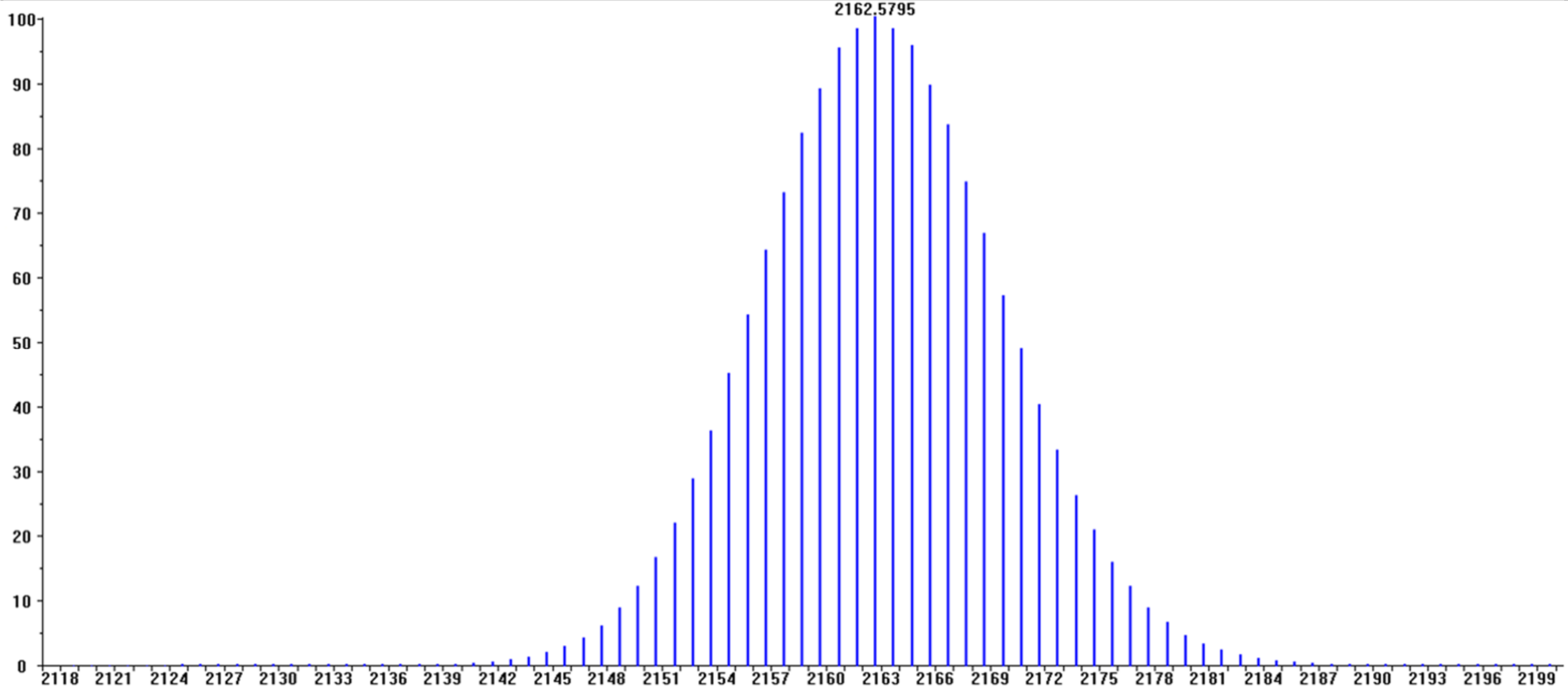

Figure S70. Simulated mass cluster for $C_{66}H_{73}O_9Si_3Sn_9^+$: {[MeSi(CH$_2$SnPhO)$_3$]$_3$ + H$^+$}$^+$. The horizontal axis shows the m/z values.

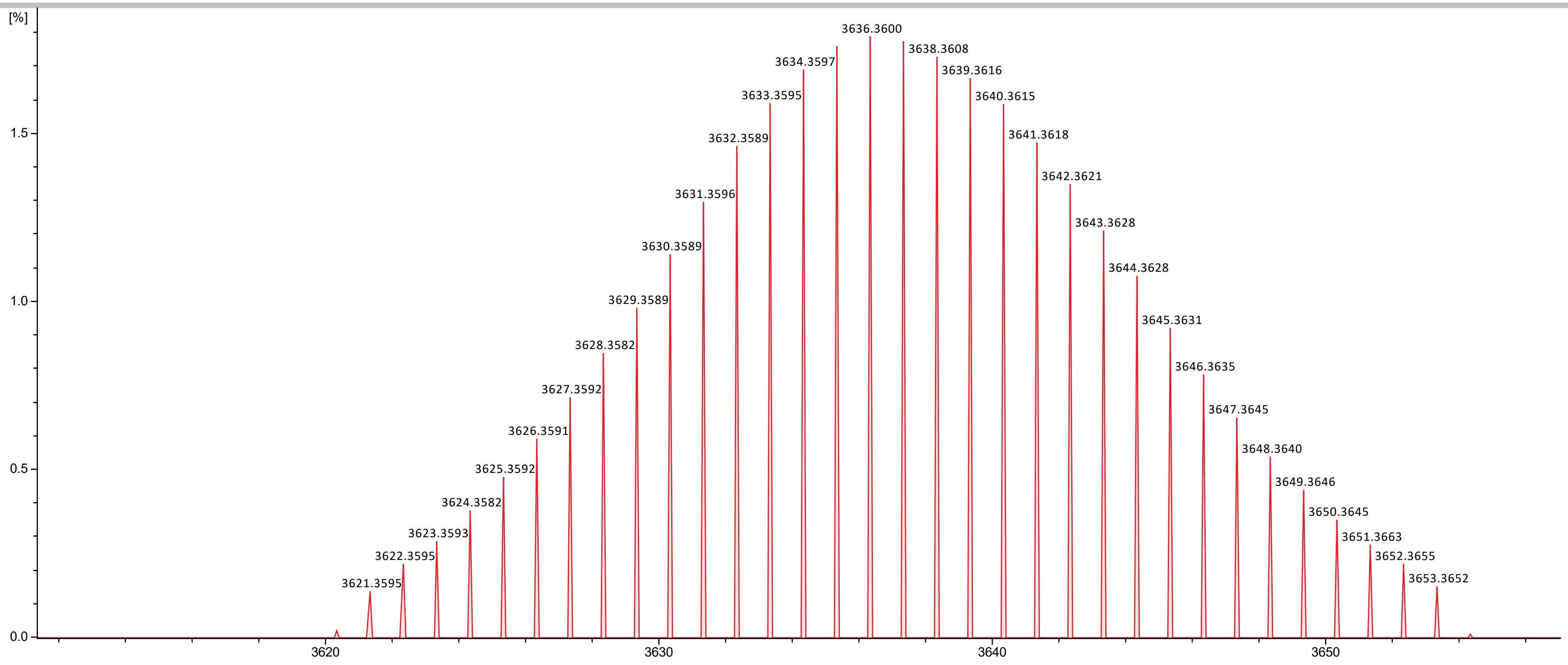

Figure S71. A fraction (c from Figure S66) of the ESI MS spectrum (positive mode) of **7**: mass cluster at m/z 3636.3600. The horizontal axis shows the m/z values.

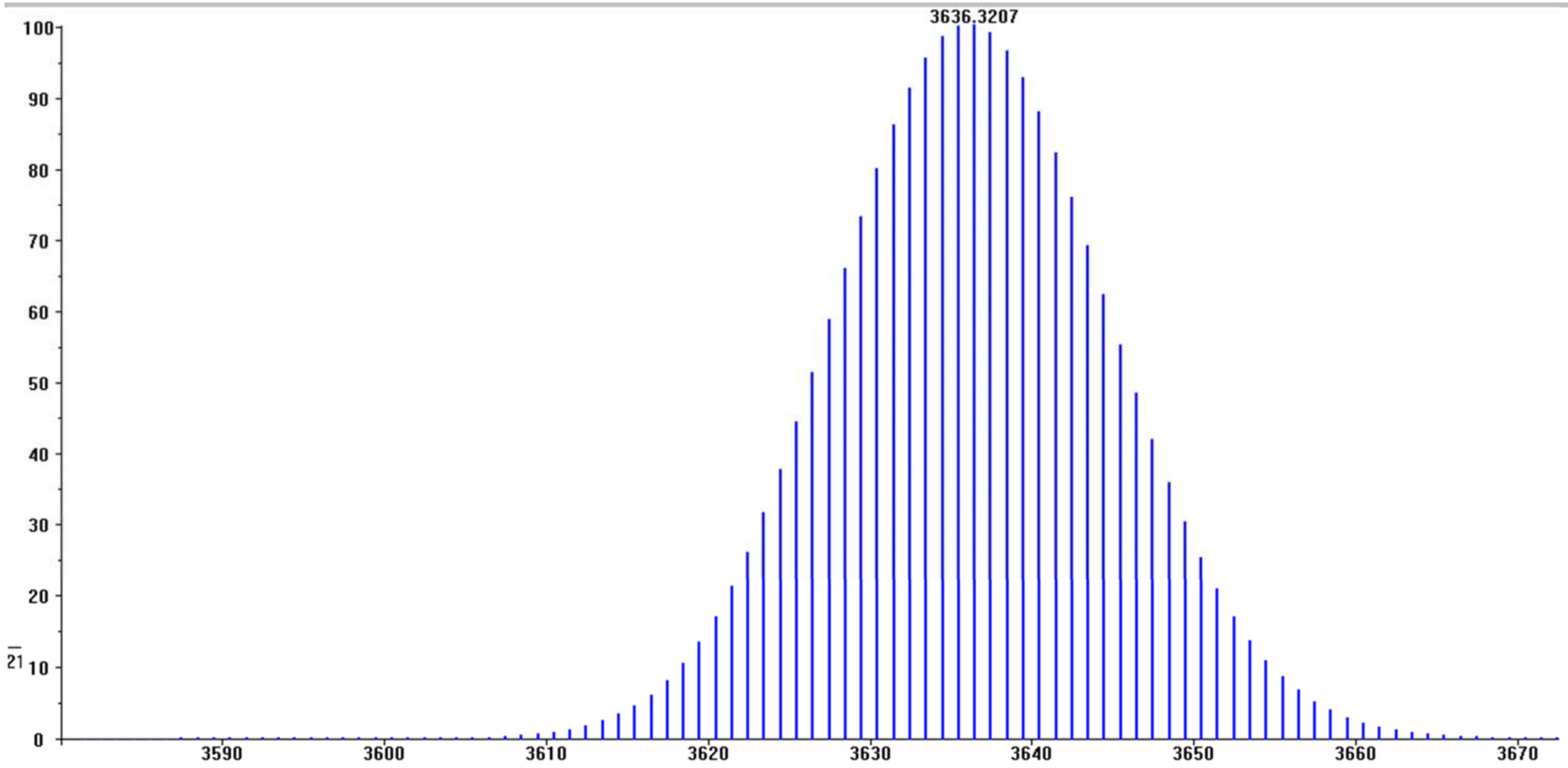

Figure S72. Simulated mass cluster of $C_{111}H_{125}O_{16}Si_5Sn_{15}^+$: $\{[MeSi(CH_2SnPhO)_3]_5 + MeOH + H^+\}^+$. The horizontal axis shows the m/z values.

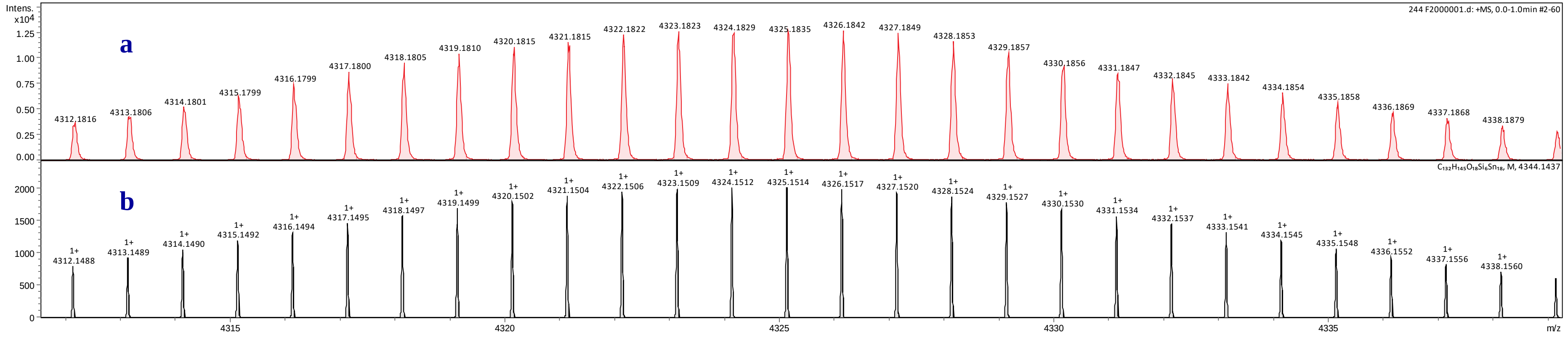

Figure S73. A fraction (c from Figure S66) of the ESI MS spectrum (positive mode) of **7**. a) experimental mass cluster at m/z 4324.1823). b) the simulated mass cluster of $C_{132}H_{145}O_{18}Si_6Sn_8^+$ {[MeSi(CH$_2$SnPhO)$_3$]$_6$ + H$^+$}$^+$.

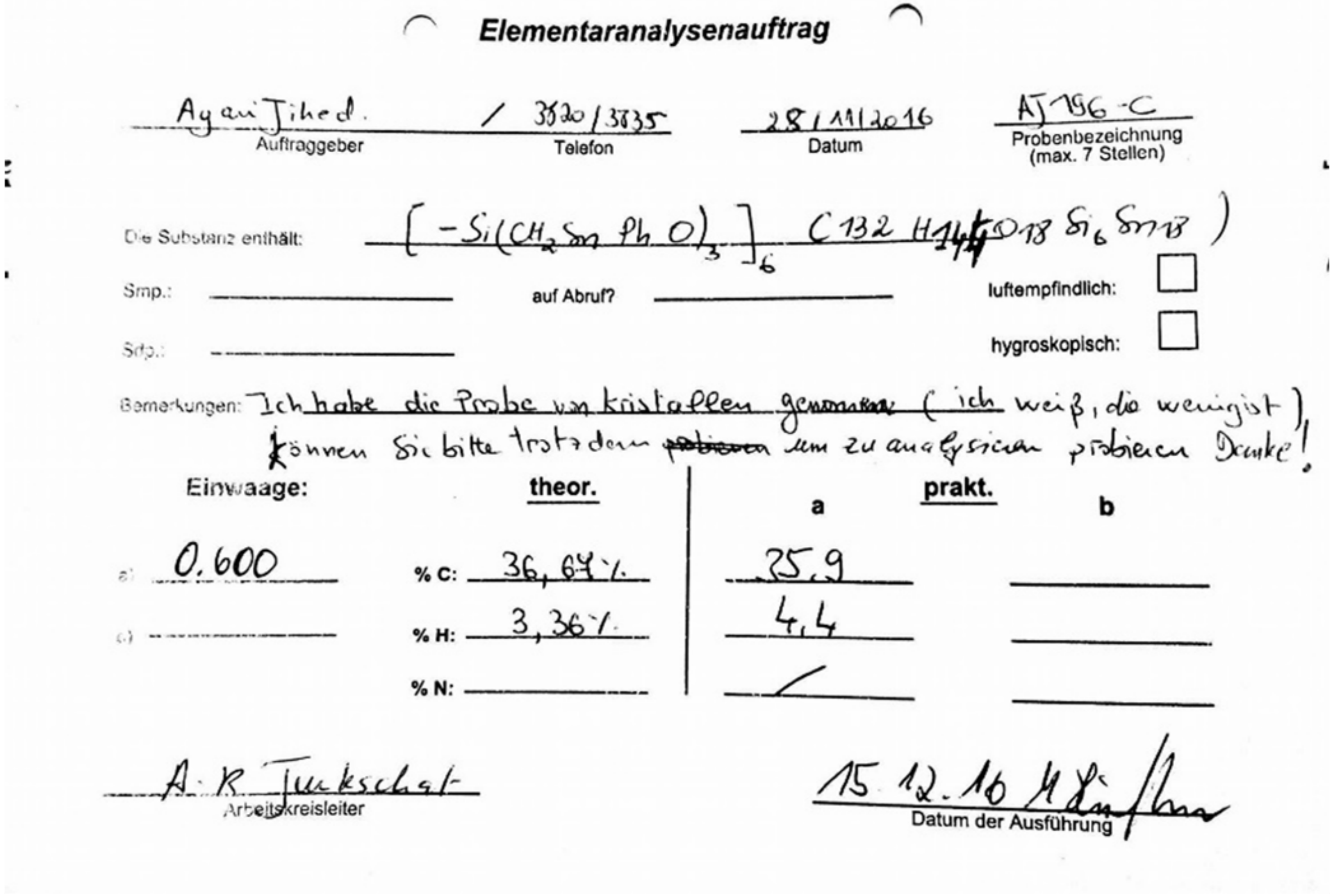

Elementaranalysenauftrag

Ayari Jihed. / 3820/3835 — 28/11/2016 — AJ 196-C

Auftraggeber — Telefon — Datum — Probenbezeichnung (max. 7 Stellen)

Die Substanz enthält: [-Si(CH₂SnPhO)₃]₆ C132 H144 O18 Si6 Sn18)

Smp.: — auf Abruf? — luftempfindlich: ☐

Sdp.: — hygroskopisch: ☐

Bemerkungen: Ich habe die Probe von kristallen genommen (ich weiß, die wenigist) können Sie bitte trotzdem um zu analysieren probieren Danke!

Einwaage:	theor.	prakt. a	prakt. b
a) 0.600	% C: 36,64 %	25.9	
c)	% H: 3,36 %	4,4	
	% N:	/	

A. R. Jurkschat — Arbeitskreisleiter

15.12.16 — Datum der Ausführung

Figure S74. Elemental analysis of **7**.

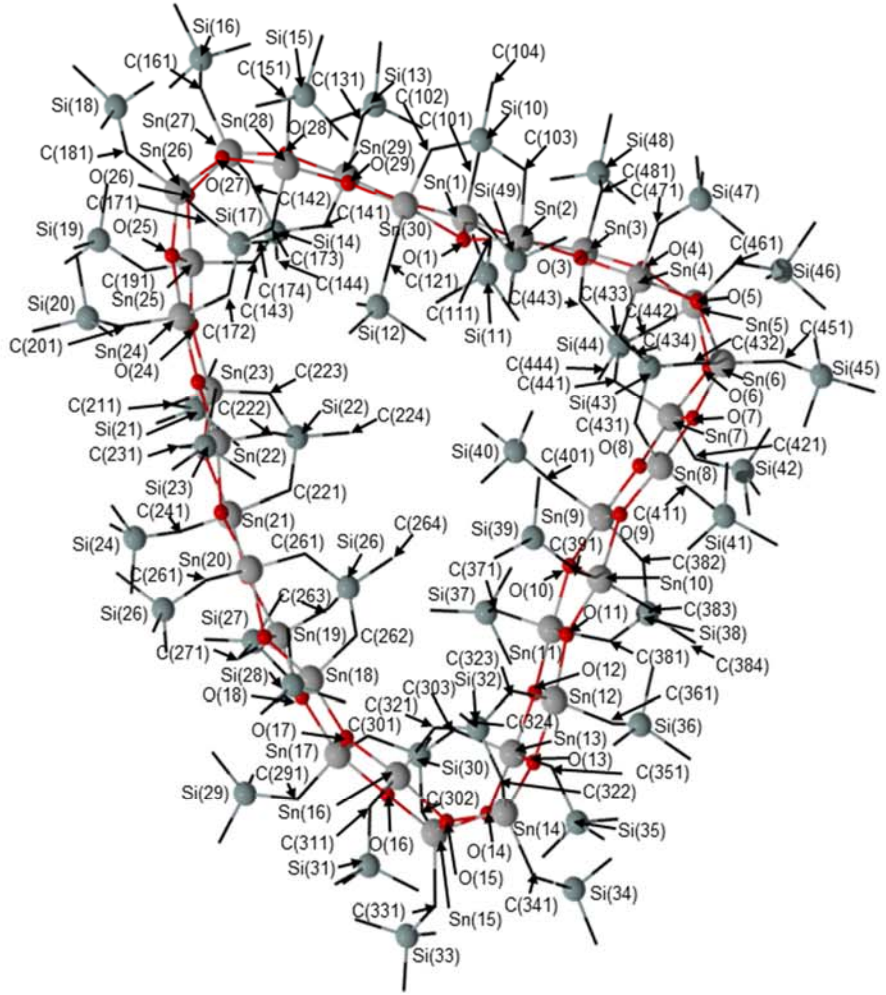

Figure S75. POV-Ray image of the structure of **8**. The subsequent Tables S3 and S4 contain interatomic distances and angles, respectiveloy, involving the tin atoms.

Table S3. Selected interatomic distances (Å) for **8**.

Sn1–O1	1.98(3)	Sn6–O5	2.07(2)	Sn11–O10	2.160(19)	Sn17–O16	2.142(18)
Sn1–O2	2.05(2)	Sn6–O6	2.19(2)	Sn11–O11	2.01(2)	Sn17–O17	2.060(19)
Sn1–O30	2.21(2)	Sn6–O7	2.03(2)	Sn11–O12	2.140(18)	Sn17–O18	2.134(18)
Sn1–C101	2.27(3)	Sn6–C432	2.23(3)	Sn11–C371	2.19(3)	Sn17–C291	2.16(3)
Sn1–C111	2.01(8)	Sn6–C451	2.07(4)	Sn11–C381	2.23(3)	Sn17–C301	2.24(3)
Sn2–O1	2.09(3)	Sn7–O6	2.11(2)	Sn12–O11	2.12(2)	Sn18–O17	2.149(18)
Sn2–O2	2.06(2)	Sn7–O7	2.01(2)	Sn12–O12	2.057(19)	Sn18–O18	2.043(19)
Sn2–O3	2.15(2)	Sn7–O8	2.184(19)	Sn12–O13	2.168(18)	Sn18–O19	2.146(18)
Sn2–C103	2.18(3)	Sn7–C421	2.19(3)	Sn12–C323	2.21(3)	Sn18–C262	2.14(3)
Sn2–C491	2.18(4)	Sn7–C441	2.13(3)	Sn12–C361	2.09(3)	Sn18–C281	2.16(3)
Sn3–O2	2.20(2)	Sn8–O7	2.23(2)	Sn13–O12	2.042(18)	Sn19–O18	2.150(19)
Sn3–O3	2.02(2)	Sn8–O8	2.022(19)	Sn14–O14	2.162(19)	Sn19–O19	1.970(18)
Sn3–O4	2.02(2)	Sn8–O9	2.099(18)	Sn14–O15	1.98(2)	Sn19–O20	2.165(18)
Sn3–C443	2.18(3)	Sn8–C411	2.12(3)	Sn14–C322	2.20(3)	Sn19–C263	2.05(3)
Sn3–C481	2.11(3)	Sn8–C431	2.16(2)	Sn14–C341	2.19(3)	Sn19–C271	2.05(3)
Sn4–O3	2.13(2)	Sn9–O8	2.063(19)	Sn15–O14	1.990(19)	Sn19–O18	2.150(19)
Sn4–O4	2.06(2)	Sn9–O9	2.067(18)	Sn15–O15	2.20(2)	Sn19–O19	1.970(18)
Sn4–O5	2.07(2)	Sn9–O10	2.117(19)	Sn15–O16	2.092(17)	Sn19–O20	2.165(18)
Sn4–C433	2.10(3)	Sn9–C382	2.25(3)	Sn15–C302	2.21(3)	Sn19–C263	2.05(3)
Sn4–C471	2.18(3)	Sn9–C401	2.17(3)	Sn15–C331	2.24(2)	Sn19–C271	2.05(3)
Sn5–O4	2.15(2)	Sn10–O9	2.207(18)	Sn16–O15	2.13(2)	Sn20–O19	2.176(18)
Sn5–O5	2.19(3)	Sn10–O10	2.01(2)	Sn16–O16	2.029(18)	Sn20–O20	1.964(19)
Sn5–O6	2.04(2)	Sn10–O11	2.18(2)	Sn16–O17	2.100(18)	Sn20–O21	2.12(2)

Sn5–C442	2.31(4)	Sn10–C383	2.19(3)	Sn16–C311	2.23(3)	Sn20–C251	2.17(3)
Sn5–C461	2.08(4)	Sn10–C391	2.23(3)	Sn16–C321	2.21(3)	Sn20–C261	2.08(3)
Sn21–O20	2.127(18)	Sn23–O22	2.14(2)	Sn26–O25	2.10(2)	Sn29–O28	2.20(2)
Sn21–O21	1.98(2)	Sn23–O23	2.03(2)	Sn26–O26	2.132(18)	Sn29–O29	2.04(2)
Sn21–O22	2.113(19)	Sn23–O24	2.14(2)	Sn26–O27	1.98(2)	Sn29–O30	2.06(2)
Sn21–C221	2.17(3)	Sn23–C211	2.17(3)	Sn26–C171	2.15(3)	Sn29–C131	2.12(3)
Sn21–C241	2.21(3)	Sn23–C223	2.13(3)	Sn26–C181	2.05(3)	Sn29–C141	2.22(3)
Sn22–O21	2.17(2)	Sn24–O23	2.10(2)	Sn27–O26	2.043(18)	Sn30–O1	2.22(2)
Sn22–O22	1.97(2)	Sn24–O24	1.994(19)	Sn27–O27	2.13(2)	Sn30–O29	2.04(2)
Sn22–O23	2.14(2)	Sn24–O25	2.19(2)	Sn27–O28	2.01(2)	Sn30–O30	1.99(2)
Sn22–C222	2.06(3)	Sn24–C172	2.19(4)	Sn27–C142	2.13(3)	Sn30–C102	2.05(4)
Sn22–C231	2.30(3)	Sn24–C201	2.24(4)	Sn27–C161	2.18(2)	Sn30–C121	2.19(3)
		Sn25–O24	2.12(2)	Sn28–O27	2.15(2)		
		Sn25–O25	1.95(2)	Sn28–O28	2.00(2)		
		Sn25–O26	2.124(18)	Sn28–O29	2.22(2)		
		Sn25–C143	2.18(3)	Sn28–C151	2.38(4)		
		Sn25–C191	2.08(4)	Sn28–C173	2.13(4)		

Table S4. Interatomic angles (°) for compound **8**.

O1–Sn1–O2	72.6(10)	O3–Sn3–O4	73.9(9)	O5–Sn5–C442	146.5(11)	O6–Sn7–C441	94.5(10)	O10–Sn9–C401	101.8(9)
O1–Sn1O–30	78.0(10)	O2–Sn3–C443	94.3(9)	O6–Sn5–C442	90.0(12)	O7–Sn7–C441	123.6(8)	C382–Sn9–C401	122.0(11)
O2–Sn1O–30	150.5(8)	O3–Sn3–C443	126.9(10)	O4–Sn5–C461	113.7(11)	O8–Sn7–C441	94.9(9)		
O1–Sn1–C101	99.7(11)	O4–Sn3–C443	96.3(10)	O5–Sn5–C461	99.3(13)	C421–Sn7–C441	115.9(12)	Sn8–O9–Sn9	103.1(8)
O2–Sn1–C101	95.7(11)	O2–Sn3–C481	100.1(11)	O6–Sn5–C461	117.4(12)			Sn8–O9–Sn10	152.0(9)
O30–Sn1–C101	90.0(11)	O3–Sn3–C481	111.6(11)	C442–Sn5–C461	113.9(14)	Sn6–O7–Sn7	109.2(9)	Sn9–O9–Sn10	95.9(7)
O1–Sn1–C111	76.8(18)	O4–Sn3–C481	97.8(12)			Sn6–O7–Sn8	139.7(10)		
O2–Sn1–C111	59(2)	C443–Sn3–C481	121.5(12)	Sn4–O5–Sn5	103.0(11)	Sn7–O7–Sn8	103.7(9)	O9–Sn10–O10	77.7(7)
O30–Sn1–C111	114(2)			Sn4–O5–Sn5	129.7(12)			O9–Sn10–O11	154.4(8)
C101–Sn1–C111	154(2)	Sn3–O3–Sn2	103.3(10)	Sn5–O5–Sn6	103.0(11)	O7–Sn8–O8	75.1(8)	O10–Sn10–O11	76.7(8)
		Sn2–O3–Sn4	150.5(11)			O7–Sn8–O9	150.0(7)	O9–Sn10–C383	93.5(10)
Sn1–O1–Sn2	104.8(11)	Sn3–O3–Sn4	106.1(9)	O5–Sn6–O6	76.9(9)	O8–Sn8–O9	75.2(7)	O10–Sn10–C383	98.5(10)
Sn1–O1–Sn30	98.9(11)			O5–Sn6–O7	123.8(9)	O7–Sn8–C411	94.3(9)	O11–Sn10–C383	90.1(11)
Sn2–O1–Sn30	133.4(12)	O3–Sn4–O4	71.1(8)	O6–Sn6–O7	72.9(9)	O8–Sn8–C411	121.5(9)	O9–Sn10–C391	102.4(10)
		O3–Sn4–O5	148.3(9)	O5–Sn6–C432	92.5(11)	O9–Sn8–C411	96.9(10)	O10–Sn10–C391	126.4(10)
O1–Sn2–O2	70.1(9)	O4–Sn4–O5	77.8(9)	O6–Sn6–C432	146.2(11)	O7–Sn8–C431	96.5(9)	O11–Sn10–C391	92.8(10)
O1–Sn2–O3	145.2(9)	O3–Sn4–C433	96.8(10)	O7–Sn6–C432	87.7(10)	O8–Sn8–C431	119.4(9)	C383–Sn10–C391	134.5(12)
O2 Sn2 O3	77.7(9)	O4–Sn4–C433	120.3(9)	O5–Sn6–C451	108.4(14)	O9–Sn8–C431	102.1(8)		
O1–Sn2–C103	98.8(11)	O5–Sn4–C433	93.6(11)	O6–Sn6–C451	91.4(14)	C411–Sn8–C431	119.0(11)	Sn9–O10–Sn10	100.5(8)
O2–Sn2–C103	100.2(11)	O3–Sn4–C471	101.1(10)	O7–Sn6–C451	118.5(14)			Sn9–O10–Sn11	137.3(9)
O3–Sn2–C103	99.6(10)	O4–Sn4–C471	118.7(10)	C432–Sn6–C451	122.3(15)	Sn7–O8–Sn8	104.8(8)	Sn10–O10–Sn11	100.5(8)
O1–Sn2–C491	96.3(13)	O5–Sn4–C471	98.9(11)			Sn7–O8–Sn9	147.9(10)		
O2–Sn2–C491	138.3(12)	C433–Sn4–C471	121.0(11)	Sn5–O6–Sn6	102.1(9)	Sn8–O8–Sn9	106.0(8)	O10–Sn11–O11	77.1(8)
O3–Sn2–C491	99.0(12)			Sn5–O6–Sn7	126.4(12)			O10–Sn11–O12	153.8(7)
C103–Sn2–C491	121.1(13)	Sn3–O4–Sn4	108.9(9)	Sn6–O6–Sn7	99.8(9)	O8–Sn9–O9	75.1(7)	O11–Sn11–O12	77.6(8)
		Sn3–O4–Sn5	142.8(11)			O8–Sn9–O10	153.4(8)	O10–Sn11–C371	94.6(10)
Sn1–O2–Sn2	103.3(9)	Sn4–O4–Sn5	104.8(9)	O6–Sn7–O7	75.1(9)	O9–Sn9–O10	78.7(7)	O11–Sn11–C371	128.1(10)
Sn1–O2–Sn3	151.3(11)			O6–Sn7–O8	150.5(8)	O8–Sn9–C382	94.7(10)	O12–Sn11–C371	96.0(10)
Sn2–O2–Sn3	100.5(9)	O4–Sn5–O5	73.4(9)	O7–Sn7–O8	76.4(8)	O9–Sn9–C382	106.9(10)	O10–Sn11–C381	92.4(9)
		O4–Sn5–O6	124.3(8)	O6–Sn7–C421	102.9(11)	O10–Sn9–C382	97.0(9)	O11–Sn11–C381	103.3(10)
O2–Sn3–O3	77.5(9)	O5–Sn5–O6	77.7(9)	O7–Sn7–C421	120.5(11)	O8–Sn9–C401	92.0(10)	O12–Sn11–C381	99.6(9)
O2–Sn3–O4	150.3(8)	O4–Sn5–C442	88.9(11)	O8–Sn7–C421	98.0(11)	O9–Sn9–C401	130.4(9)	C371–Sn11–C381	128.3(12)
Sn10–O11–Sn11	100.1(9)	Sn13–O13–Sn14	108.2(8)	O15–Sn16–O16	76.9(8)	O18–Sn18–O19	73.6(7)	O20–Sn20–C251	129.5(11)
Sn10–O11–Sn12	152.3(11)			O15–Sn16–O17	149.8(8)	O17–Sn18–C262	94.6(9)	O21–Sn20–C251	96.6(12)

Sn11–O11–Sn12	103.7(9)	O13–Sn14–O14	75.2(7)	O16–Sn16–O17	74.7(7)	O18–Sn18–C262	109.5(9)	O19–Sn20–C261	94.2(10)
		O13–Sn14–O15	123.9(8)	O15–Sn16–C311	101.0(9)	O19–Sn18–C262	97.9(9)	C261–Sn20–O20	104.3(10)
O11–Sn12–O12	77.1(8)	O14–Sn14–O15	75.2(8)	O16–Sn16–C311	118.2(9)	O17–Sn18–C281	97.8(10)	O21 Sn20C261	101.3(11)
O11–Sn12–O13	152.3(8)	O13–Sn14–C322	89.4(10)	O17–Sn16–C311	101.5(9)	O18–Sn18–C281	125.7(9)	C251–Sn20–C261	126.1(14)
O12–Sn12–O13	75.2(7)	O14–Sn14–C322	150.9(10)	O15–Sn16–C321	92.5(9)	O19–Sn18–C281	96.3(10)		
O11–Sn12–C323	98.3(10)	O15–Sn14–C322	94.3(11)	O16–Sn16–C321	122.8(9)	C262–Sn18–C281	124.8(11)	Sn19–O20–Sn20	101.4(8)
O12–Sn12–C323	120.3(10)	O13–Sn14–C341	120.9(9)	O17–Sn16–C321	93.9(9)			Sn19–O20–Sn21	147.3(9)
O13–Sn12–C323	95.8(9)	O14–Sn14–C341	94.6(9)	C311–Sn16–C321	119.0(10)	Sn17–O18–Sn18	102.8(8)	Sn20–O20–Sn21	104.4(8)
O11–Sn12–C361	99.2(10)	O15–Sn14–C341	108.1(10)			Sn17–O18–Sn19	152.5(10)		
O12–Sn12–C361	115.8(9)	C322–Sn14–C341	114.5(11)	Sn15O16Sn16	107.5(8)	Sn18–O18–Sn19	98.8(7)	O20–Sn21–O21	74.3(8)
O13–Sn12–C361	92.5(9)			Sn15O16Sn17	141.2(9)			O20–Sn21–O22	148.6(7)
C323–Sn12–C361	123.6(11)	Sn13–O14–Sn14	100.2(7)	Sn16O16Sn17	105.8(7)	O18–Sn19–O19	74.9(7)	O21–Sn21–O22	74.9(8)
		Sn13–O14–Sn15	127.4(9)			O18–Sn19–O20	150.8(7)	O20–Sn21–C221	102.4(9)
Sn11–O12–Sn12	101.4(8)	Sn14–O14–Sn15	104.7(8)	O16–Sn17–O17	73.2(7)	O19–Sn19–O20	76.0(7)	O21–Sn21–C221	105.9(10)
Sn11–O12–Sn13	153.3(10)			O16–Sn17–O18	150.1(7)	O18 Sn19C263	95.6(10)	O22–Sn21–C221	91.9(9)
Sn12–O12–Sn13	105.2(8)	O14–Sn15–O15	74.1(8)	O17–Sn17–O18	77.7(7)	O19–Sn19–C263	103.0(10)	O20–Sn21–C241	99.3(10)
		O14–Sn15–O16	124.1(7)	O16–Sn17–C291	93.9(9)	O20–Sn19–C263	93.3(10)	O21–Sn21–C241	126.0(10)
O12–Sn13–O13	76.2(7)	O15–Sn15–O16	74.3(7)	O17–Sn17–C291	110.3(9)	O18–Sn19–C271	101.2(9)	O22–Sn21–C241	93.6(10)
O12–Sn13–O14	149.0(7)	O14–Sn15–C302	96.8(9)	O18–Sn17–C291	102.7(9)	O19–Sn19–C271	128.0(10)	C221–Sn21–C241	127.3(12)
O13–Sn13–O14	74.6(7)	O15–Sn15–C302	150.4(9)	O16–Sn17–C301	97.8(8)	O20–Sn19–C271	94.7(9)		
O12–Sn13–C303	95.1(9)	O16–Sn15–C302	88.9(8)	O17–Sn17–C301	127.1(9)	C263–Sn19–C271	128.8(12)	Sn20–O21–Sn21	104.1(8)
O13–Sn13–C303	124.3(9)	O14–Sn15–C331	110.0(9)	O18–Sn17–C301	94.1(8)			Sn20–O21–Sn22	145.5(11)
O14–Sn13–C303	93.0(9)	O15–Sn15–C331	95.0(9)	C291–Sn17–C301	122.4(11)	Sn18–O19–Sn19	101.3(7)	Sn21–O21–Sn22	101.1(9)
O12–Sn13–C351	96.4(10)	O16–Sn15–C331	117.7(8)			Sn18–O19–Sn20	134.4(9)		
O13–Sn13–C351	115.3(10)	C302–Sn15–C331	114.6(9)	Sn16–O17–Sn17	106.2(8)	Sn19–O19–Sn20	100.8(8)	O21–Sn22–O22	73.9(8)
O14–Sn13–C351	105.1(9)			Sn16–O17–Sn18	151.6(10)			O21–Sn22–O23	149.3(8)
C303–Sn13–C351	120.3(11)	Sn14–O15–Sn15	103.9(9)	Sn17–O17–Sn18	101.7(8)	O19–Sn20–O20	75.8(7)	O22–Sn22–O23	75.7(8)
		Sn14–O15–Sn16	131.5(10)			O19–Sn20–O21	149.3(7)	O21–Sn22–C222	96.8(10)
Sn12–O13–Sn13	103.3(8)	Sn15–O15–Sn16	100.3(9)	O17–Sn18–O18	77.7(7)	O20–Sn20–O21	74.8(8)	O22–Sn22–C222	100.3(9)
Sn12–O13–Sn14	141.6(9)			O17–Sn18–O19	151.1(7)	O19–Sn20–C251	95.2(11)	O23–Sn22–C222	92.7(10)
O21–Sn22–C231	93.6(9)	O25–Sn24–C201	95.7(13)			Sn27O28Sn29	140.9(11)		
O22–Sn22–C231	130.0(9)	C172–Sn24–C201	131.6(15)	Sn25–O26–Sn26	102.7(8)	Sn28O28Sn29	104.0(10)	Si10–C101–Sn1	110.6(17)
O23–Sn22–C231	102.7(9)			Sn25–O26–Sn27	127.6(8)			Si11–C111–Sn1	100(3)
C222–Sn22–C231	129.5(10)	Sn23–O24–Sn24	104.0(9)	Sn26–O26–Sn27	104.7(8)	O28–Sn29–O29	76.5(9)	Si12–C121–Sn30	120.6(16)
		Sn23–O24–Sn25	148.1(10)			O28–Sn29–O30	148.1(9)	Si13–C131–Sn29	120.9(17)

Sn21–O22–Sn22	103.4(9)	Sn24–O24–Sn25	107.3(8)	O26–Sn27–O27	73.0(8)	O29–Sn29–O30	72.3(9)	Si14–C141–Sn29	122.0(17)
Sn21–O22–Sn23	136.6(9)			O26–Sn27–O28	125.0(8)	O28–Sn29–C131	92.7(11)	Si15–C151–Sn28	116(2)
Sn22–O22–Sn23	102.0(8)	O24–Sn25–O25	73.9(8)	O27–Sn27–O28	74.7(8)	O29–Sn29–C131	111.0(11)	Si16–C161–Sn27	122.5(14)
		O24–Sn25–O26	146.5(7)	O26–Sn27–C142	95.6(11)	O30–Sn29–C131	104.4(12)	Si17–C171–Sn26	114.1(14)
O22–Sn23–O23	74.4(8)	O25–Sn25–O26	74.0(8)	O27–Sn27–C142	150.0(11)	O28–Sn29–C141	94.4(11)	Si17–C172–Sn24	121(2)
O22–Sn23–O24	146.2(7)	O24–Sn25–C143	93.9(10)	O28–Sn27–C142	90.8(12)	O29–Sn29–C141	125.1(11)	Si17–C173–Sn28	125.7(18)
O23–Sn23–O24	75.0(8)	O25–Sn25–C143	122.8(11)	O26–Sn27–C161	109.6(9)	O30–Sn29–C141	98.1(11)	Si18–C181–Sn26	123.5(17)
O22–Sn23–C211	94.1(11)	O26–Sn25–C143	95.1(10)	O27–Sn27–C161	94.3(8)	C131–Sn29–C141	123.6(13)	Si19–C191–Sn25	129.0(18)
O23–Sn23–C211	135.5(10)	O24–Sn25–C191	102.2(11)	O28–Sn27–C161	116.2(10)			Si20–C201–Sn24	128(3)
O24–Sn23–C211	97.7(11)	O25–Sn25–C191	114.7(11)	C142–Sn27–C161	115.7(12)	Sn28–O29–Sn29	102.2(10)	Si21–C211–Sn23	119.2(17)
O22–Sn23–C223	95.5(10)	O26–Sn25–C191	100.0(11)			Sn28–O29–Sn30	150.9(12)	Si22–C221–Sn21	114.8(14)
O23–Sn23–C223	103.7(11)	C143–Sn25–C191	122.5(13)	Sn26–O27–Sn27	107.0(9)	Sn29–O29–Sn30	106.1(9)	Si22–C222–Sn22	113.5(14)
O24–Sn23–C223	105.4(10)			Sn26–O27–Sn28	130.6(10)			Si22–C223Sn23	113.5(15)
C211–Sn23–C223	120.3(12)	Sn24–O25–Sn25	106.3(9)	Sn27–O27–Sn28	100.5(8)	O1–Sn30–O29	150.4(9)	Si23–C231–Sn22	113.4(14)
		Sn24–O25–Sn26	134.1(11)			O1–Sn30–O30	77.6(9)	Si24–C241–Sn21	122.9(18)
Sn22–O23–Sn23	100.2(8)	Sn25–O25–Sn26	110.4(10)	O27–Sn28–O28	74.3(9)	O29–Sn30–O30	73.7(9)	Si25–C251–Sn20	124.8(19)
Sn22–O23–Sn24	155.3(11)			O27–Sn28–O29	149.7(8)	O1–Sn30–C102	97.5(13)	Si26–C261–Sn20	117.3(16)
Sn23–O23–Sn24	104.3(9)	O25–Sn26–O26	70.9(8)	O28–Sn28–O29	76.9(9)	O29–Sn30–C102	96.5(14)	Si26–C262–Sn18	112.8(14)
		O25–Sn26–O27	124.6(8)	O27–Sn28–C151	95.4(13)	O30–Sn30–C102	104.5(14)	Si26–C263–Sn19	113.7(14)
O23–Sn24–O24	76.5(8)	O26–Sn26–O27	74.0(8)	O28–Sn28–C151	103.7(13)	O1–Sn30–C121	91.5(11)	Si27–C271–Sn19	122.2(16)
O23–Sn24–O25	148.0(8)	O25–Sn26–C171	93.3(10)	O29–Sn28–C151	100.3(12)	O29–Sn30–C121	101.0(11)	Si28–C281–Sn18	117.0(15)
O24–Sn24–O25	71.5(8)	O26–Sn26–C171	149.1(9)	O27–Sn28–C173	90.9(12)	O30–Sn30–C121	129.2(10)	Si29–C291–Sn17	121.7(15)
O23–Sn24–C172	98.4(12)	O27–Sn26–C171	95.6(11)	O28–Sn28–C173	116.2(12)	C102–Sn30–C121	126.1(14)	Si30–C301–Sn17	116.4(14)
O24–Sn24–C172	124.8(12)	O25–Sn26–C181	116.0(11)	O29–Sn28–C173	93.5(11)			Si30–C302–Sn15	113.1(13)
O25–Sn24–C172	98.5(12)	O26–Sn26–C181	95.1(10)	C151–Sn28–C173	139.8(15)	Sn1–O30–Sn29	149.5(11)	Si30–C303–Sn13	124.3(14)
O23–Sn24–C201	93.1(13)	O27–Sn26–C181	108.5(11)			Sn1–O30–Sn30	99.0(10)	Si31–C311–Sn16	116.4(13)
O24–Sn24–C201	103.6(13)	C171–Sn26–C181	115.7(12)	Sn27–O28–Sn28	110.1(10)	Sn29–O30–Sn30	107.6(10)	Si32–C321–Sn16	122.1(14)
Si32–C322–Sn14	112.6(17)	Si36–C361–Sn12	123.3(14)	Si39–C391–Sn10	113.9(16)	Si43–C432–Sn6	119.9(18)	Si48–C481–Sn3	122.4(18)
Si32–C323–Sn12	120.1(15)	Si37–C371–Sn11	118.2(16)	Si40–C401–Sn9	124.0(15)	Si43–C433–Sn4	125.1(15)	Si49–C491–Sn2	119(2)
Si33–C331–Sn15	120.1(14)	Si38–C381–Sn11	114.7(15)	Si41–C411–Sn8	121.4(14)	Si45–C451–Sn6	123(2)		
Si34–C341–Sn14	120.3(14)	Si38–C382–Sn9	108.0(14)	Si42–C421–Sn7	122(2)	Si46–C461–Sn5	132(2)		
Si35–C351–Sn13	119.5(16)	Si38–C383–Sn10	112.5(17)	Si43–C431–Sn8	121.4(13)	Si47–C471–Sn4	125.0(16)		

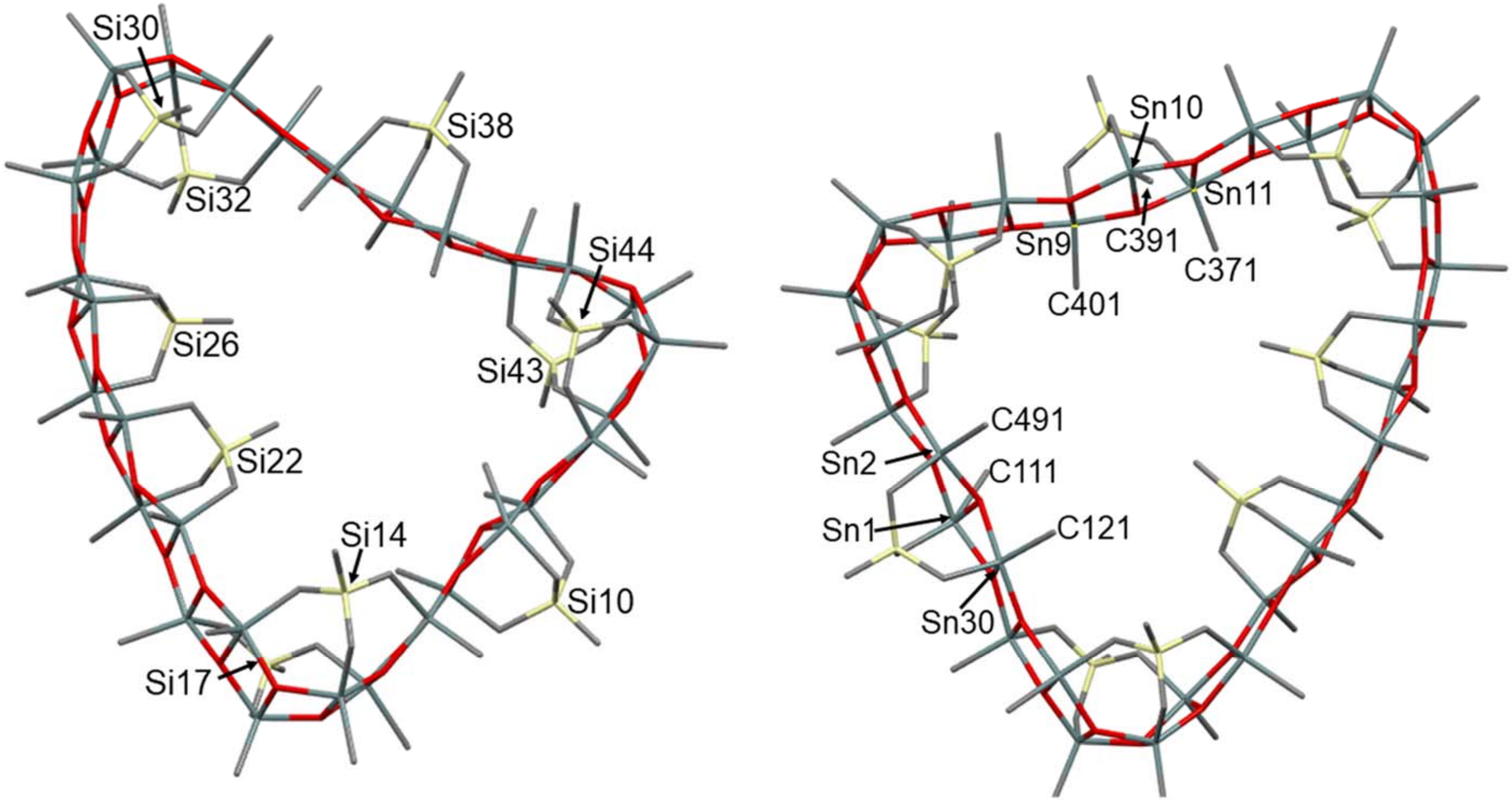

Figure S76. Simplified molecular structure of **8** illustrating the positions of the $SiCH_3$ moieties and the substituents at the tin atoms pointing inside the cavity.

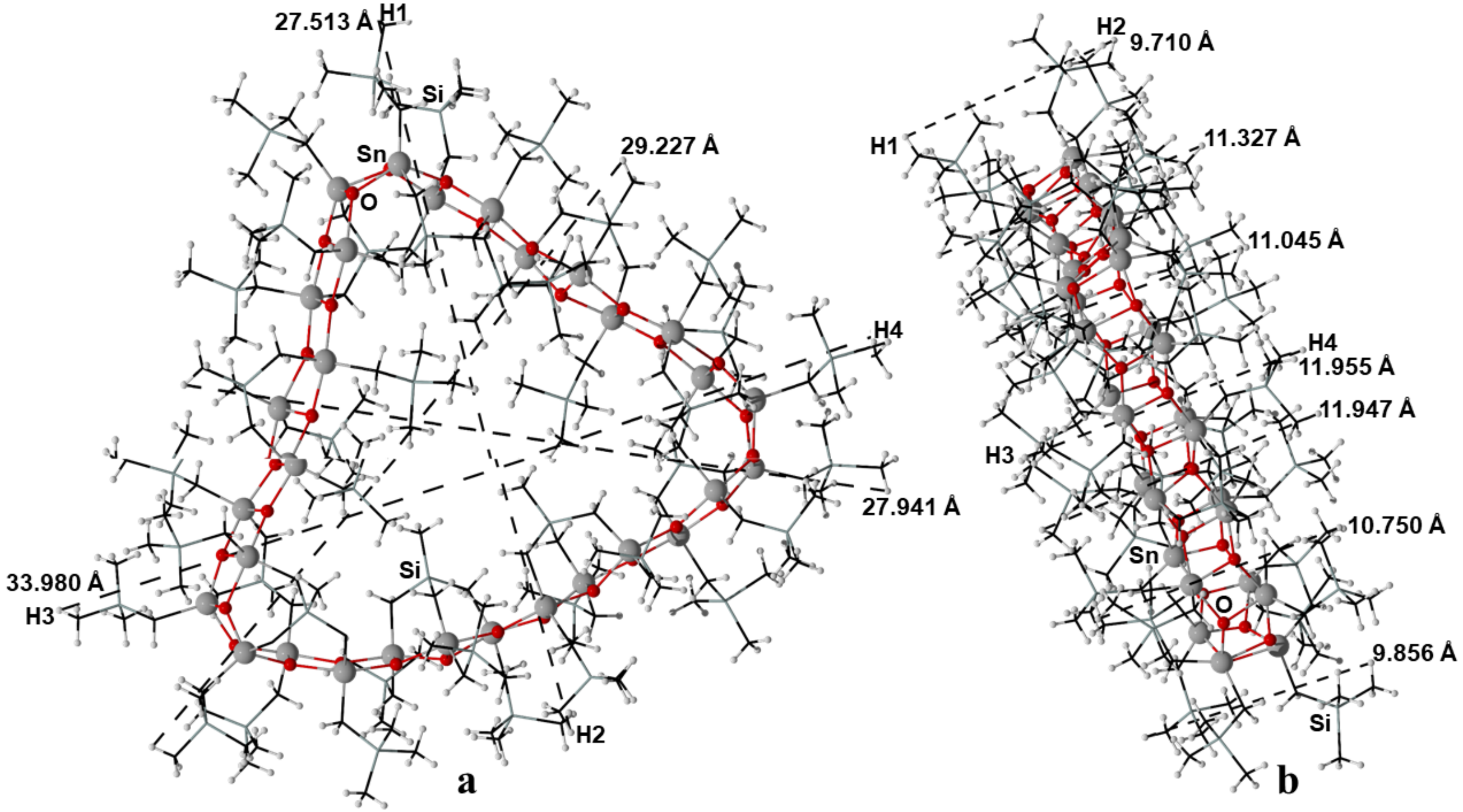

Figure S77. Front view a) and side view b) (POV-Ray) of **8** including the H24F···H46F (27.51(1) Å) and H16K···H34K (33.98(1) Å) distances and the distances indicative for the thickness (H34D···H35D 9.71(1) Å, H11F···H49C 11.96(1) Å).

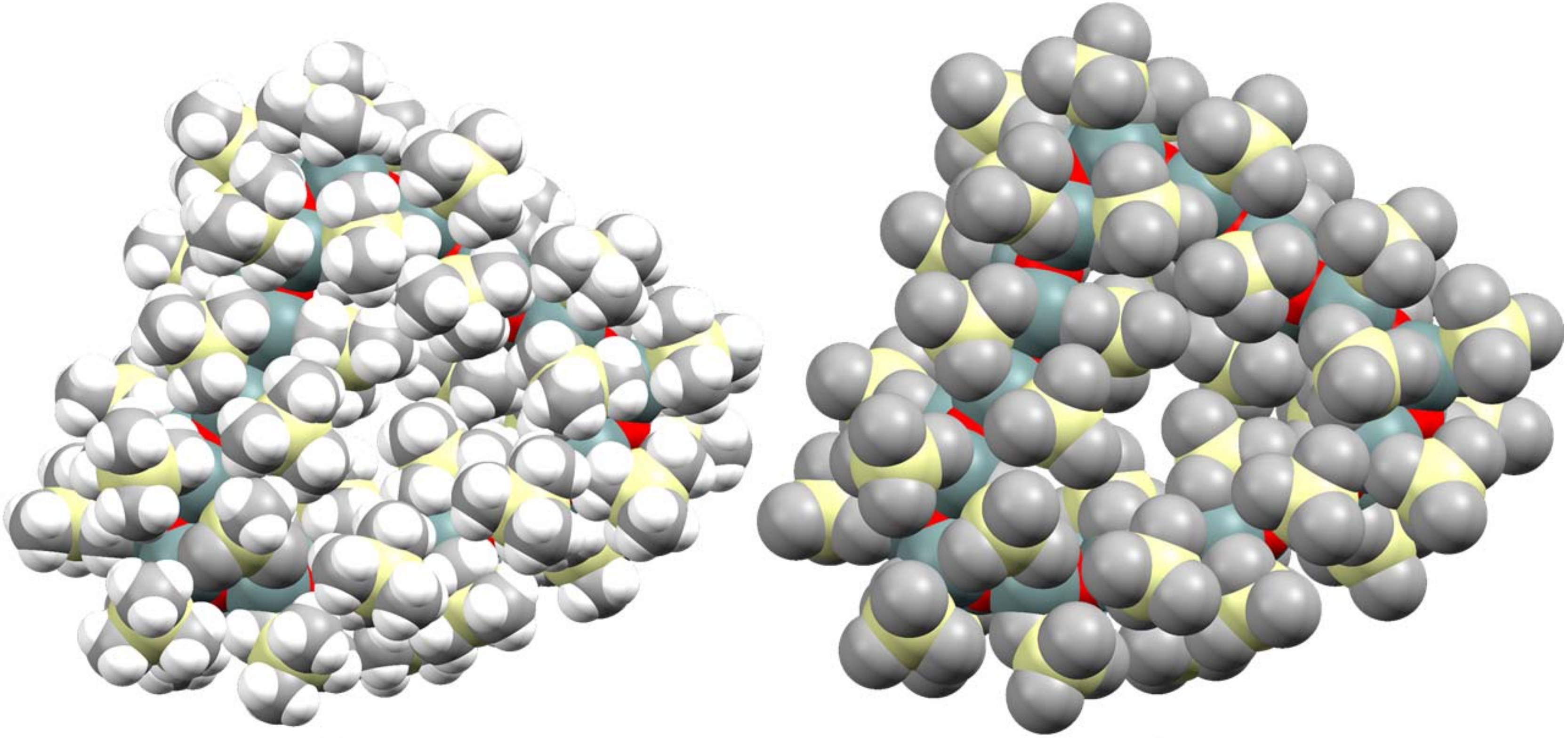

Figure S78. POV Ray images of the centred cavity of macrocycle **8** in space fill mode (left site with hydrogen atoms, right site without hydrogen atoms).

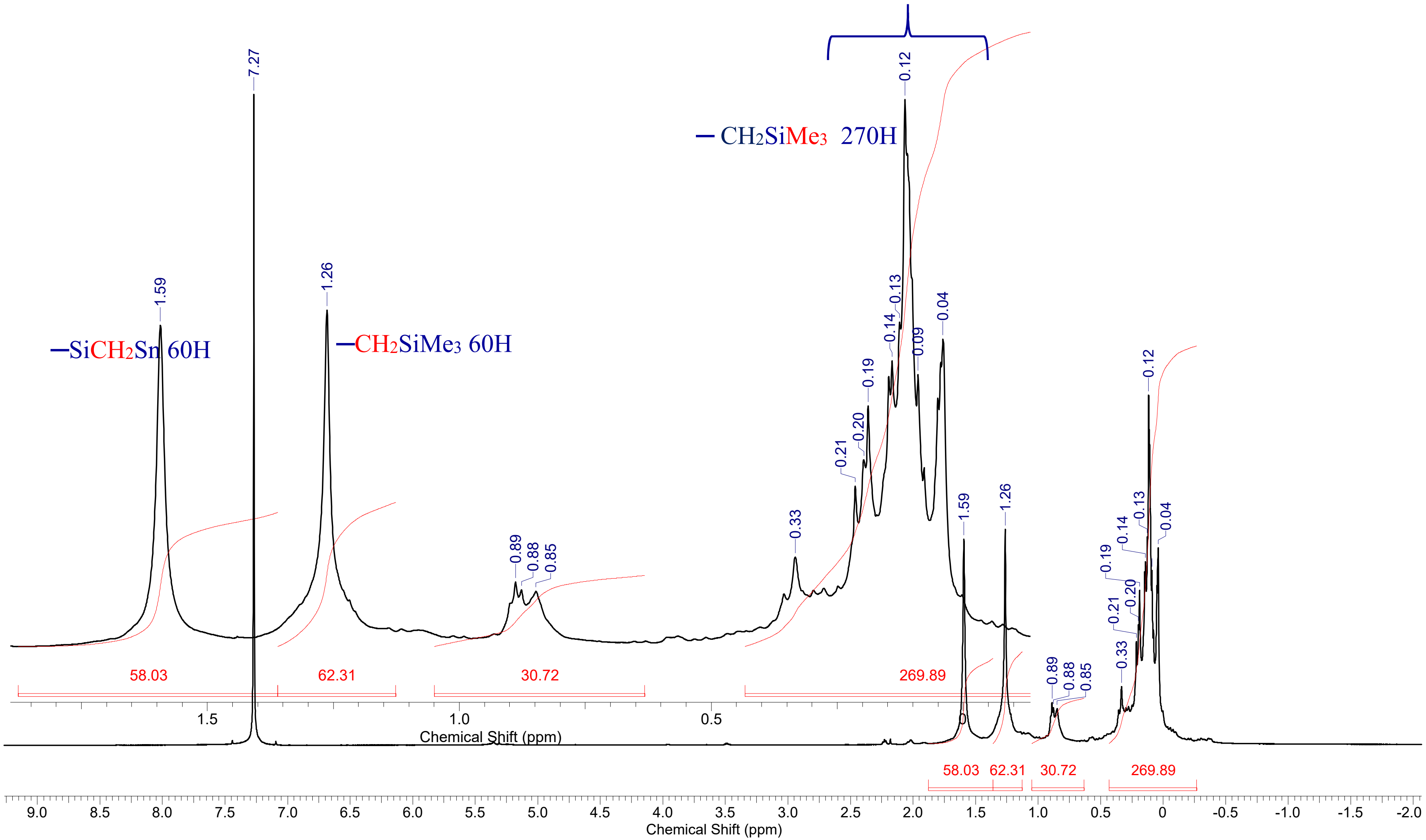

Figure S79. ^{1}H NMR spectrum (600.29 MHz, $CDCl_3$) of a solution of the bulk crystalline material (from which a single crystal of **8** was taken) obtained from the reaction between the diorganotin diiodide derivative **6** and sodium hydroxide, NaOH.

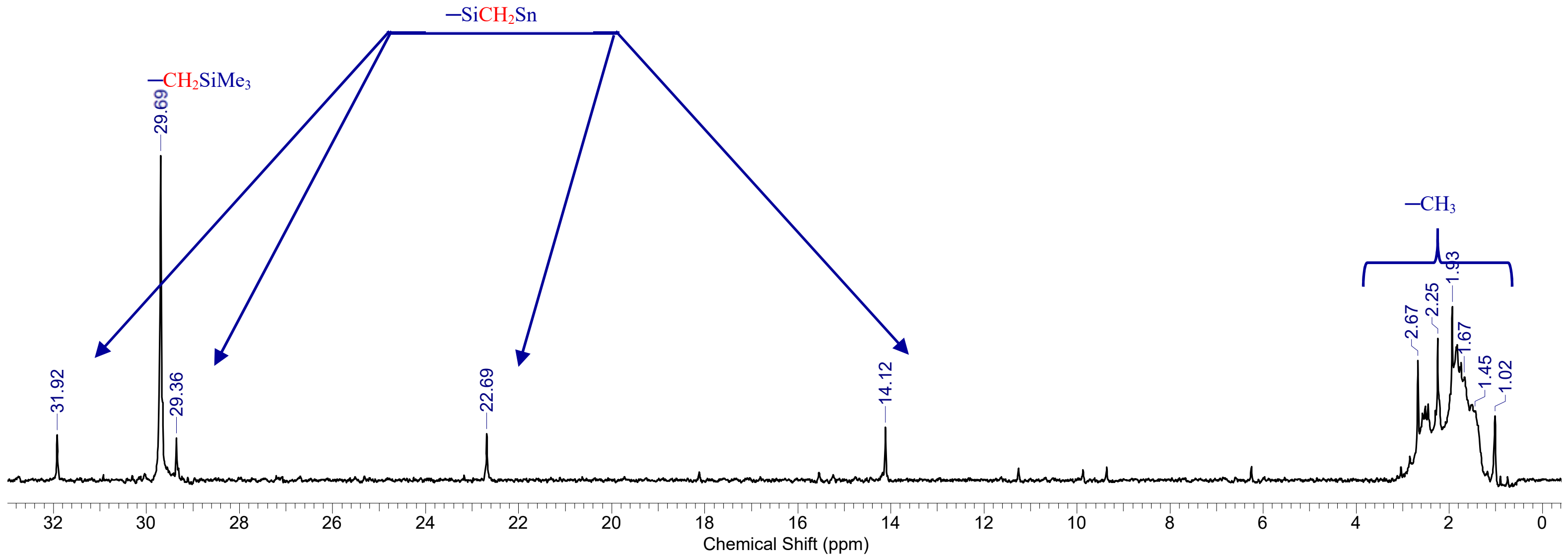

Figure S80. ^{13}C NMR spectrum (150.94 MHz, $CDCl_3$) of a solution of the bulk crystalline material (from which a single crystal of **8** was taken) obtained from the reaction between the diorganotin diiodide derivative **6** and sodium hydroxide, NaOH.

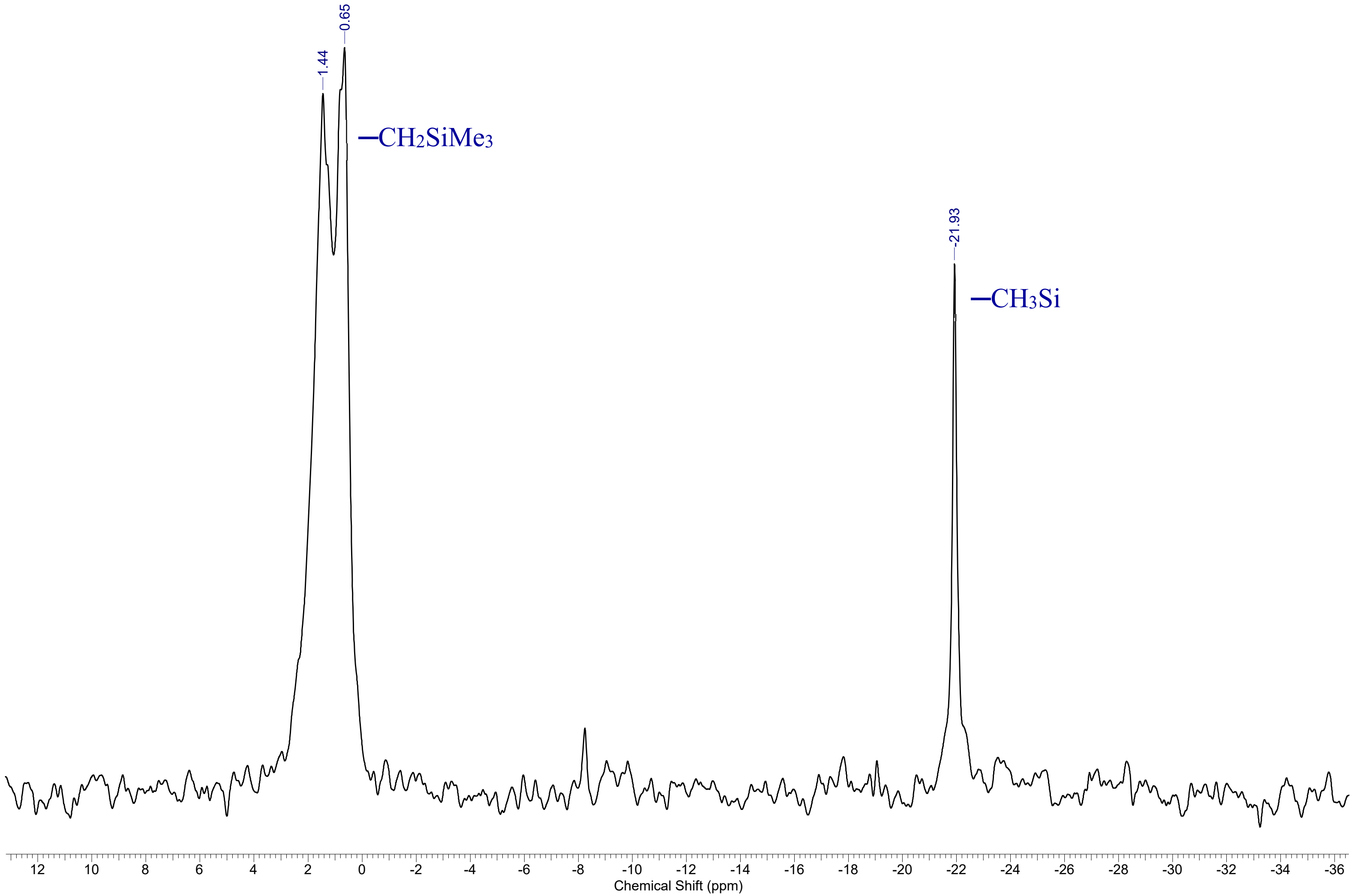

Figure S81. ^{29}Si NMR spectrum (119.26 MHz, $CDCl_3$) of a solution of the bulk crystalline material (from which a single crystal of **8** was taken) obtained from the reaction between the diorganotin diiodide derivative **6** and sodium hydroxide, NaOH.

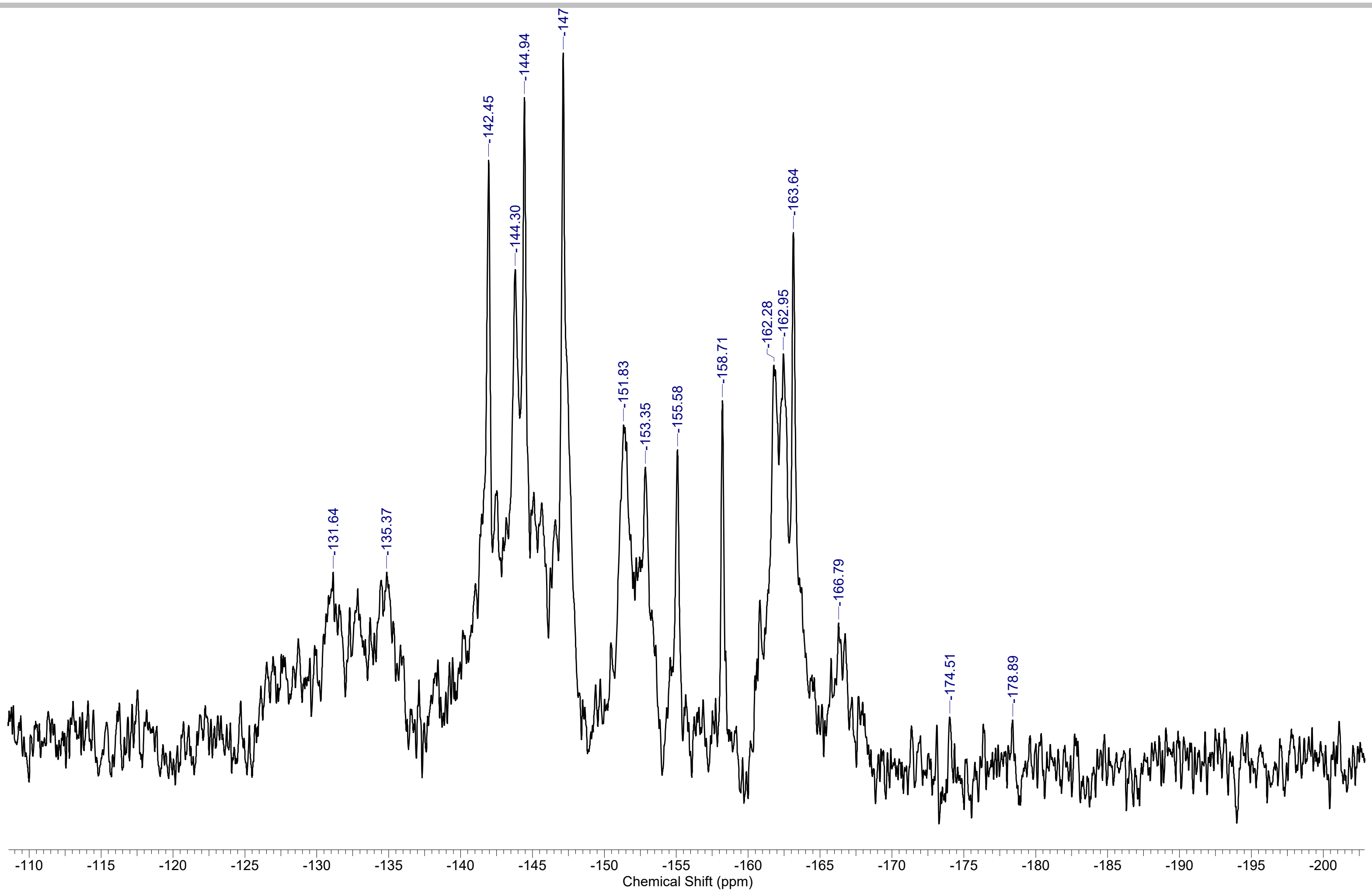

Figure S82. ^{119}Sn NMR (223.85 MHz, $CDCl_3$) of crude reaction mixture obtained from the reaction between the diorganotin diiodide derivative **6** and sodium hydroxide NaOH.

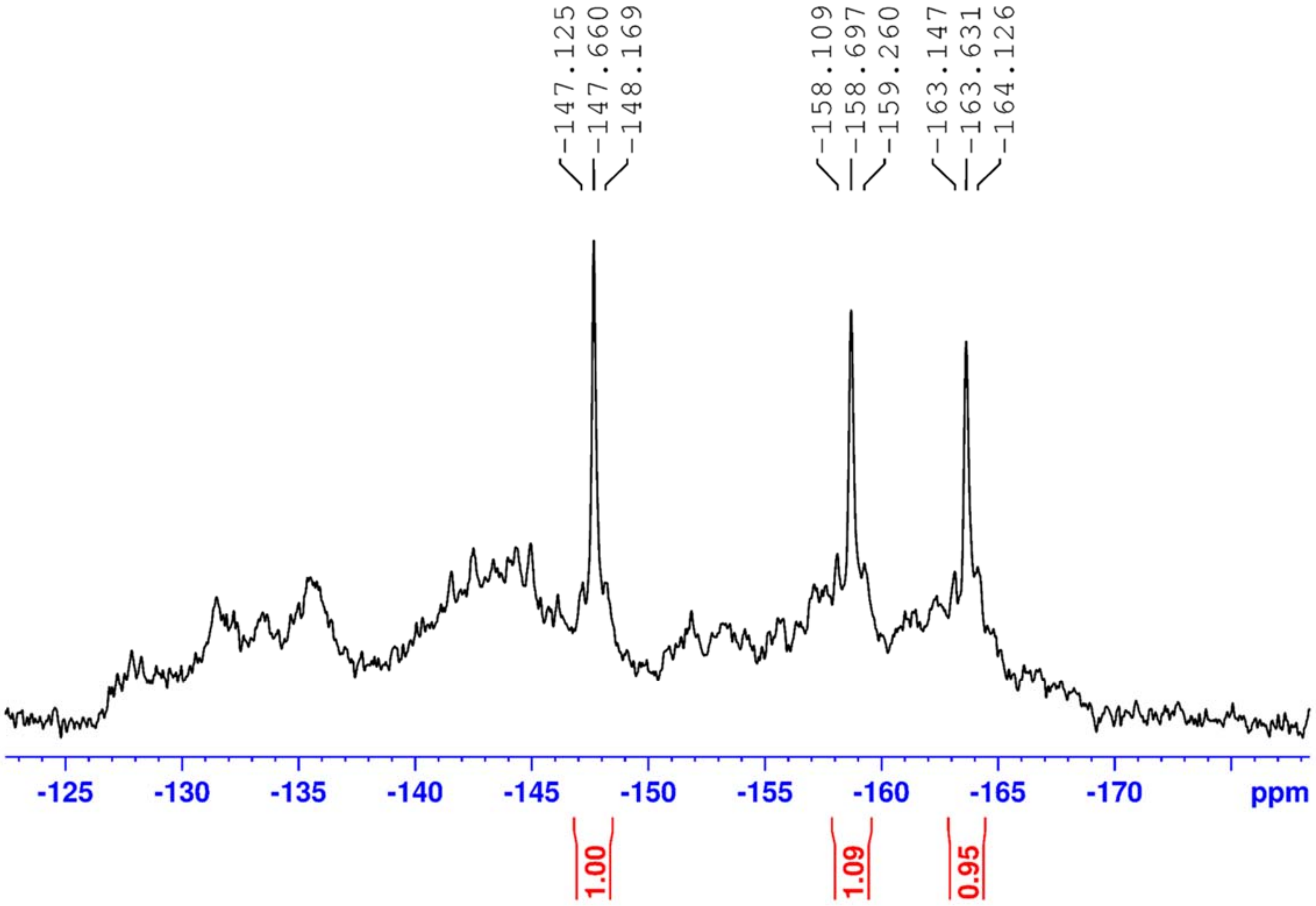

Figure S83. ^{119}Sn NMR spectrum (223.85 MHz, $CDCl_3$) of a solution of the bulk crystalline material (from which a single crystal of **8** was taken) obtained from the reaction between the diorganotin diiodide derivative **6** and sodium hydroxide, NaOH.

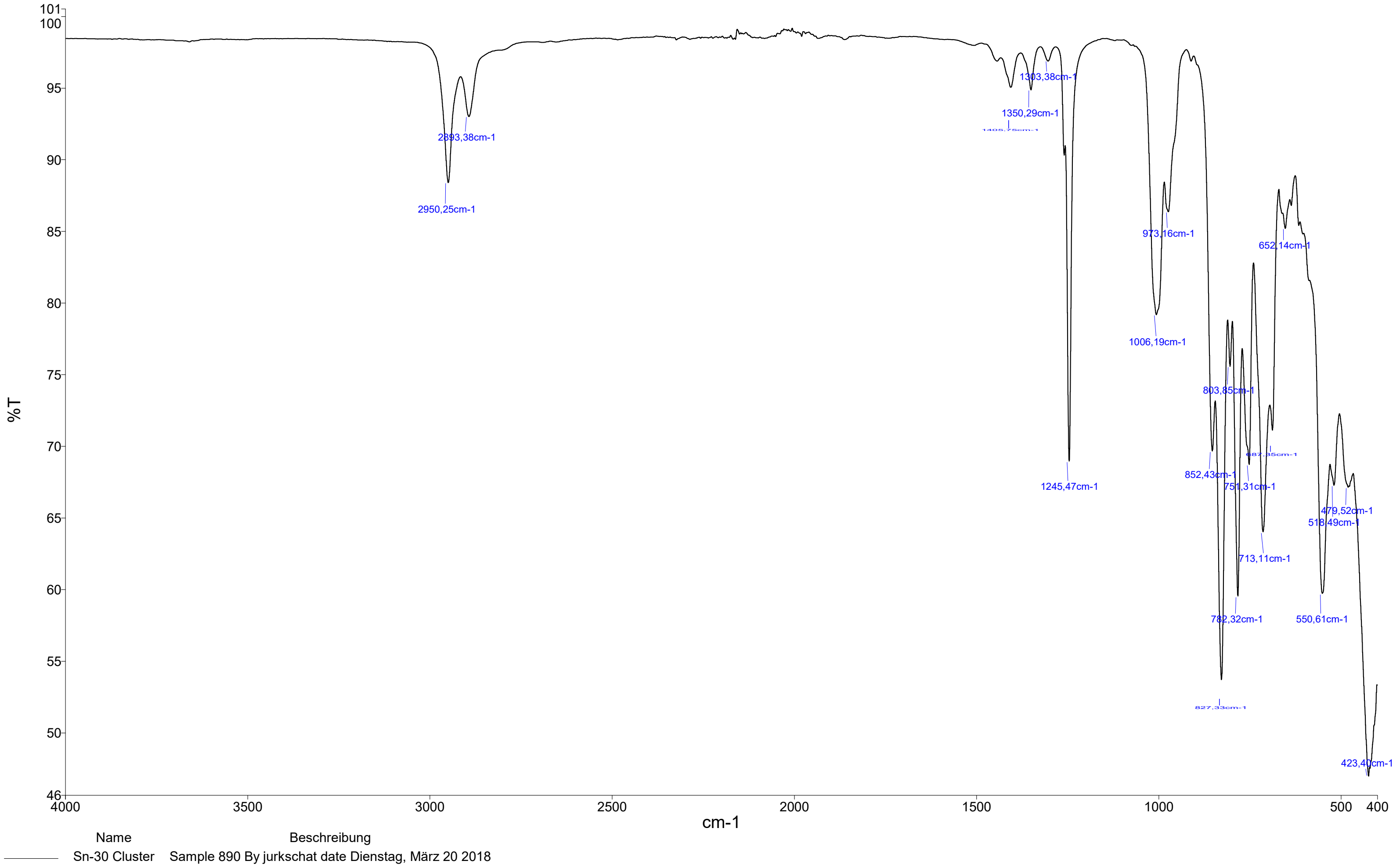

Figure S84. IR spectrum (ATR) of the bulk crystalline material (from which a single crystal of **8** was taken) obtained from the reaction between the diorganotin diiodide derivative **6** and sodium hydroxide, NaOH. It proofs the absence of SnOH in this material.

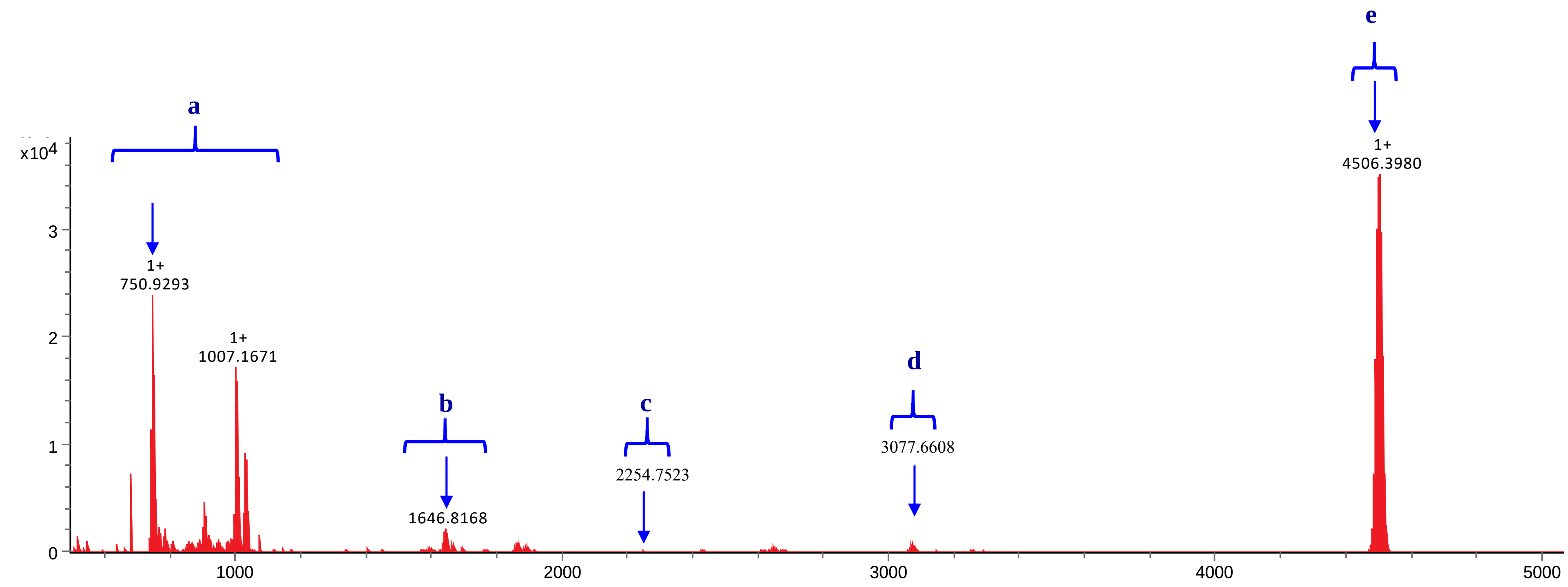

Figure S85. ESI MS spectrum (positive mode) of **8**. The blue arrows and letters refer to cut-outs of this spectrum shown in the subsequent figures. The horizontal axis shows the m/z values.

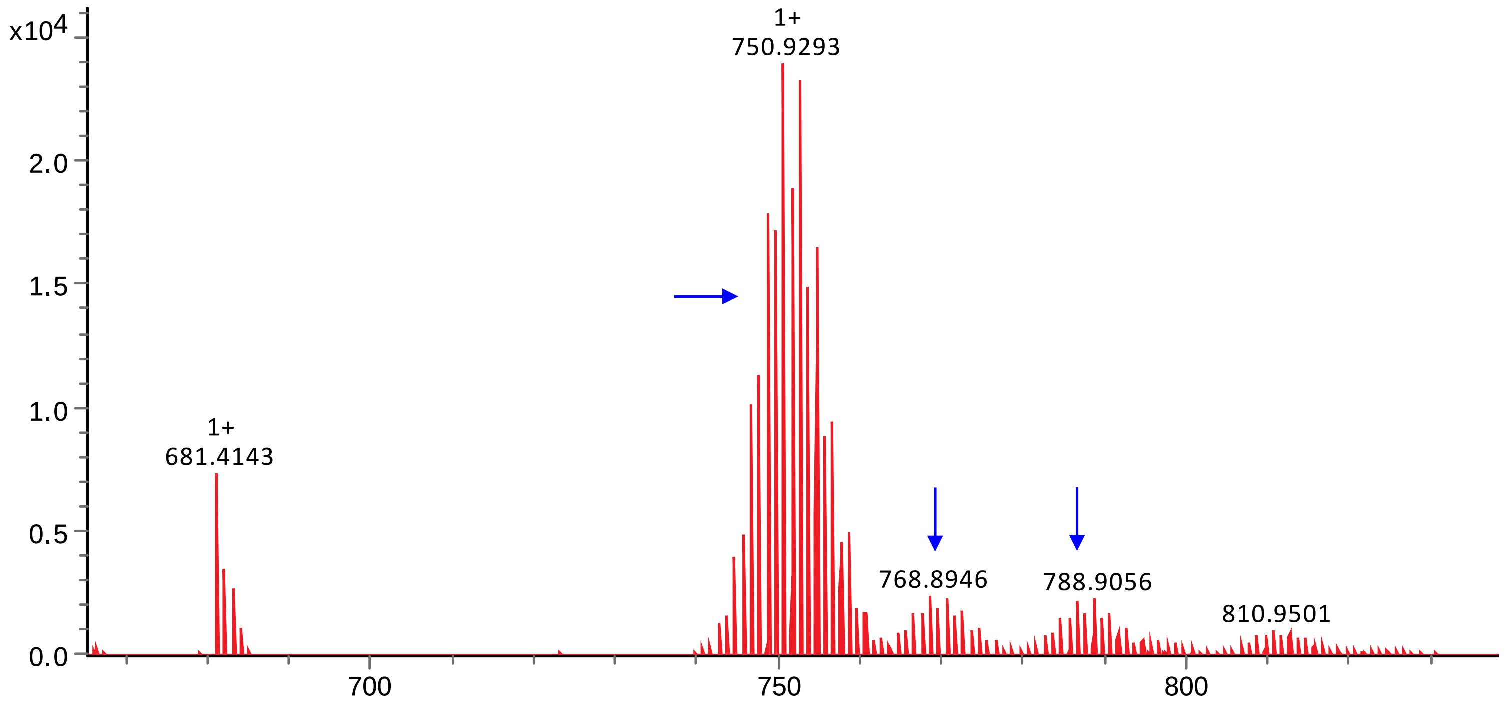

Figure S86. A fraction (a from Figure S85) of the ESI MS spectrum (positive mode) of **8**: mass cluster centred at m/z 750.9293. The blue arrow refers to the subsequent Figure S87. The horizontal axis shows the m/z values.

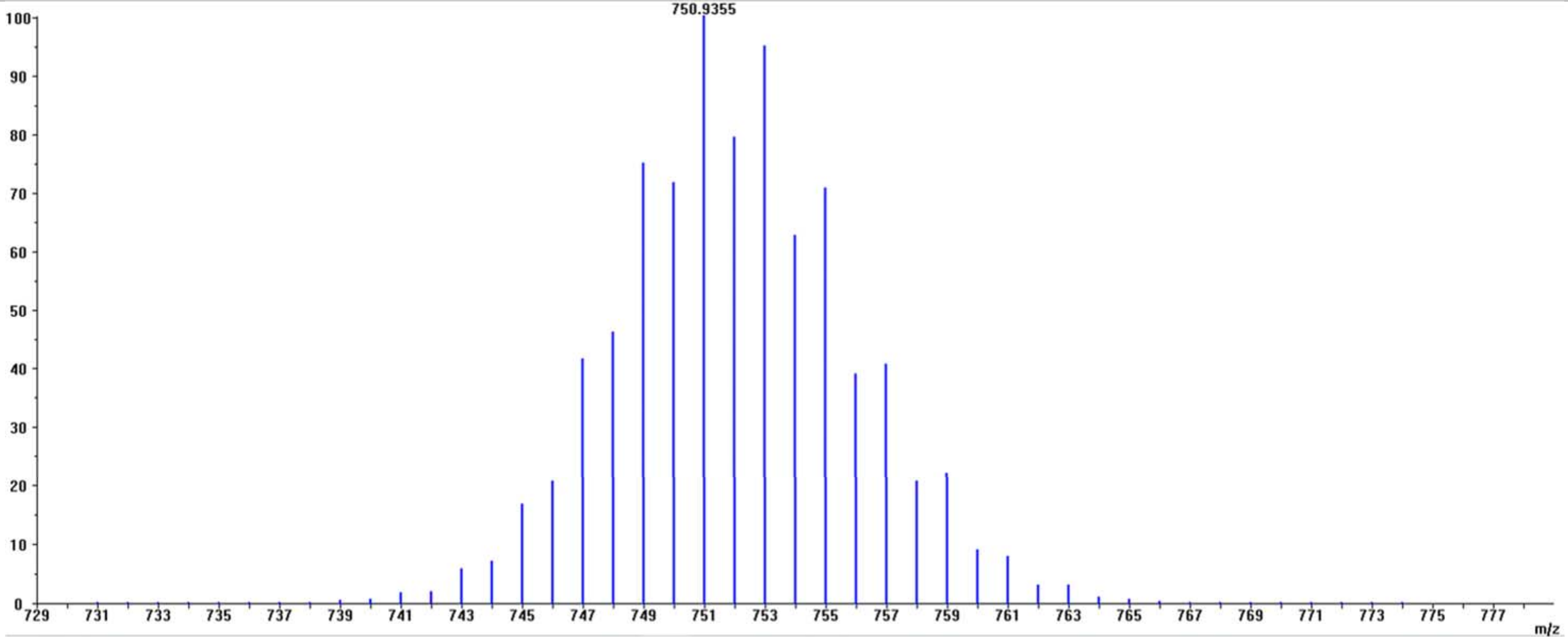

Figure S87a. Simulated mass cluster of $C_{16}H_{43}O_3Si_4Sn_3^+$: $[MeSi(CH_2SnCH_2SiMe_3O)_3 + H^+]^+$. The horizontal axis shows the m/z values.

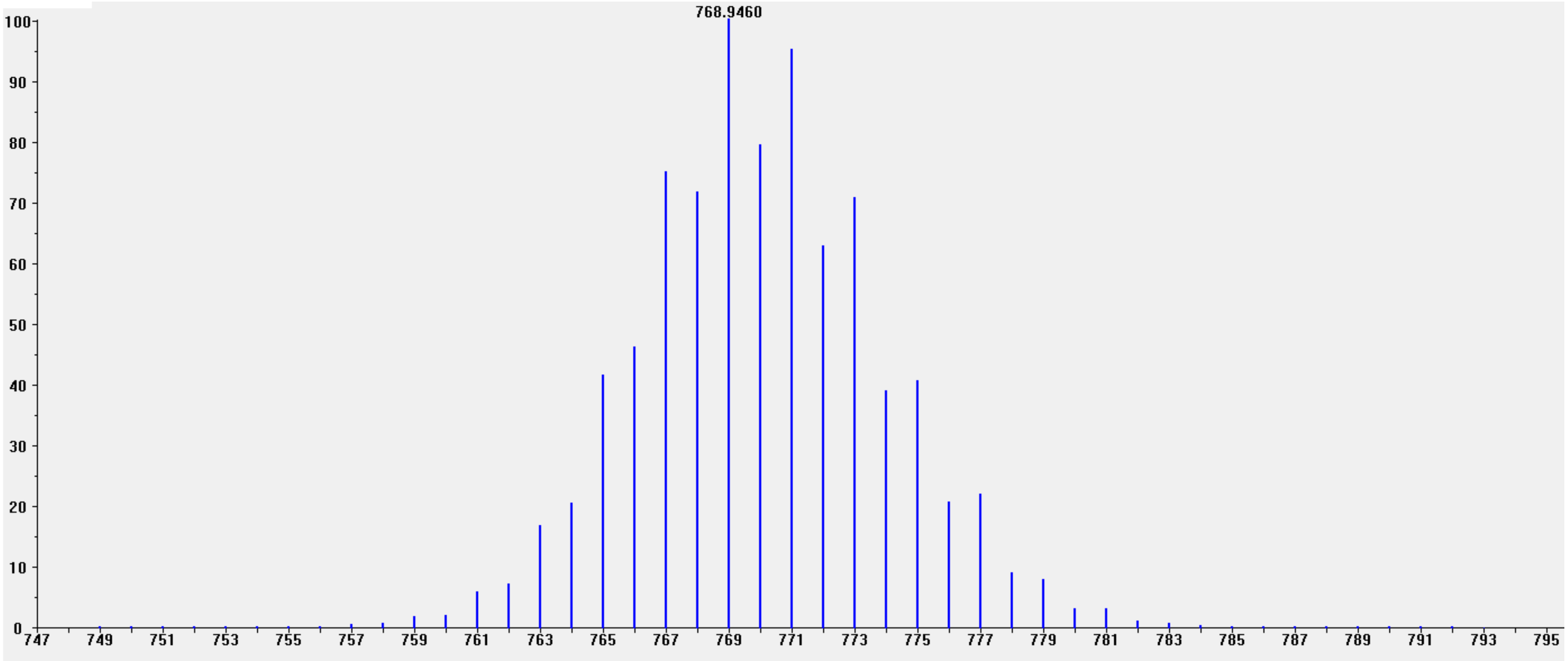

Figure S87b. Simulated mass cluster of $C_{16}H_{45}O_4Si_4Sn_3^+$: $[MeSi(CH_2SnCH_2SiMe_3O)_3 + H_2O + H^+]^+$. The horizontal axis shows the m/z values.

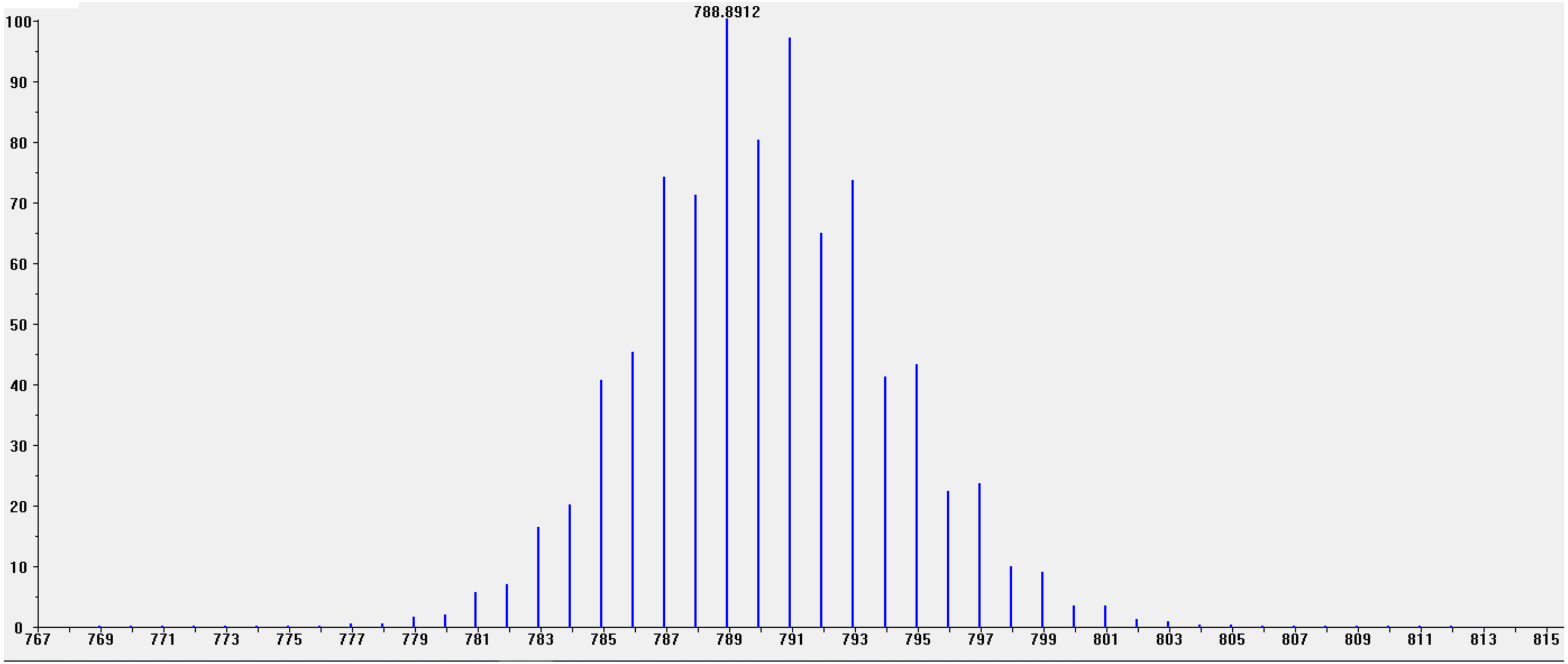

Figure S87c. Simulated mass cluster of $C_{16}H_{42}O_3Si_4KSn_3^+$: [MeSi($CH_2SnCH_2SiMe_3O$)$_3$ + K^+]$^+$. The horizontal axis shows the m/z values.

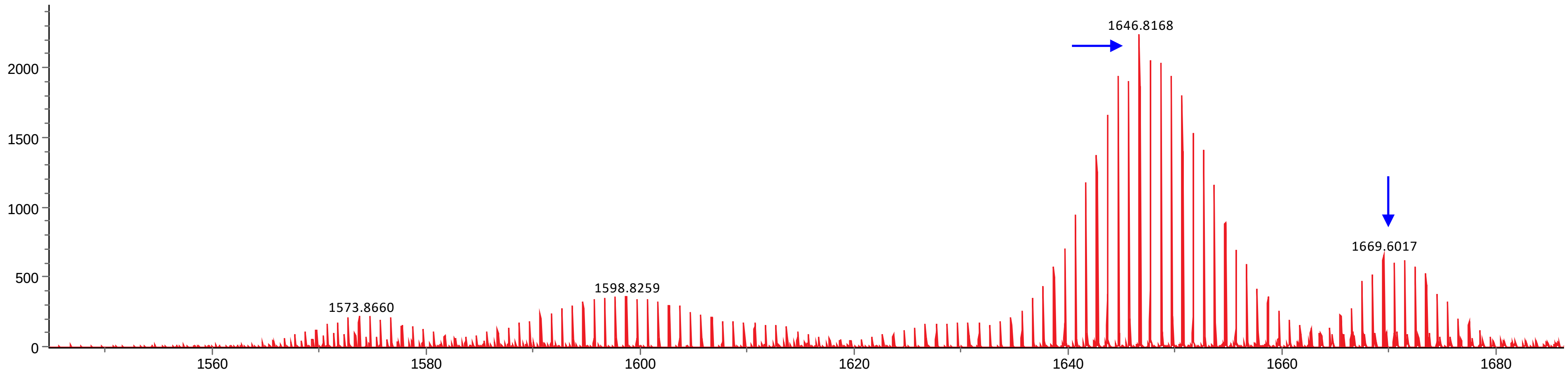

Figure S88. A fraction (b from Figure S85) of the ESI MS spectrum of **8**: mass cluster centred at m/z 1646.8168. The blue arrow refers to the subsequent Figure S89 .The horizontal axis shows the m/z values.

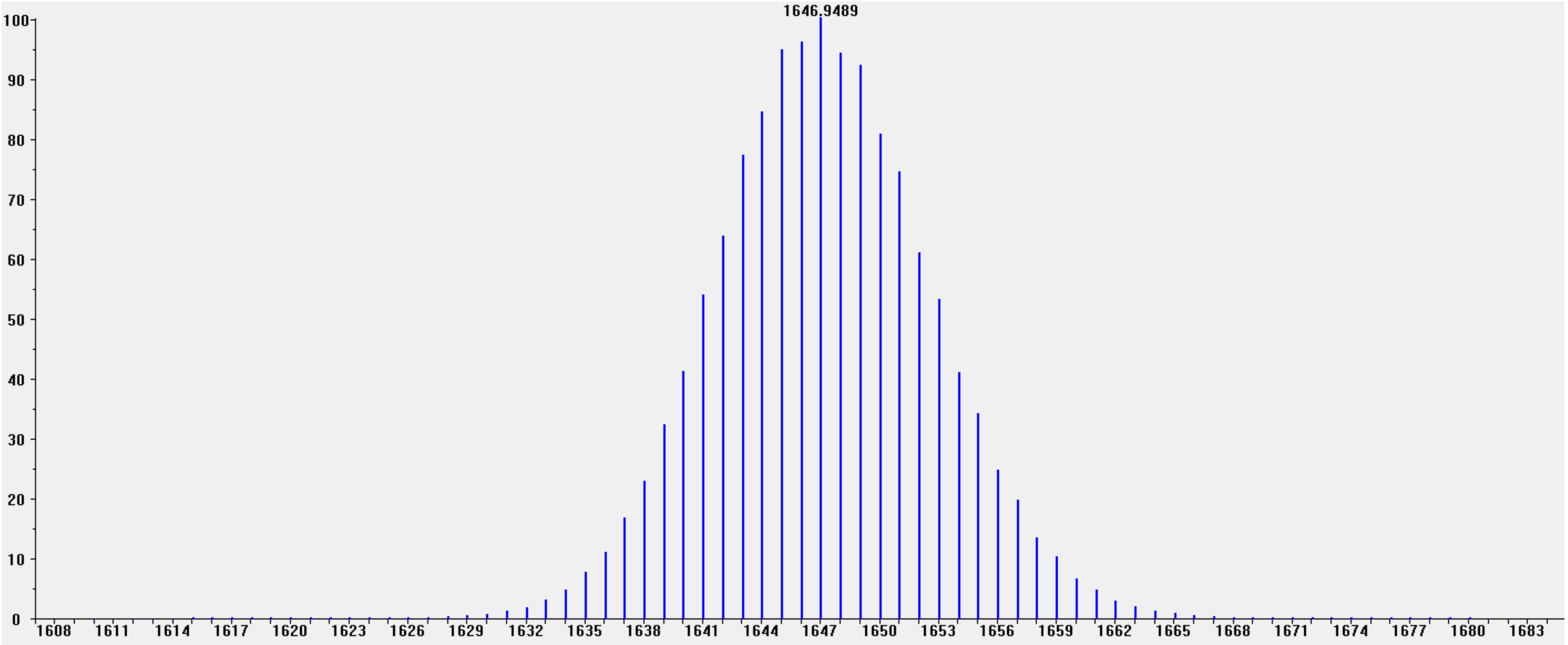

Figure S89a. Simulated mass cluster for $C_{32}H_{101}O_{14}Si_8Sn_6^+$: $\{[MeSi(CH_2Sn(OH)_2CH_2SiMe_3)_3]_2 + 2\ H_2O + H^+\}^+$. The horizontal axis shows the m/z values.

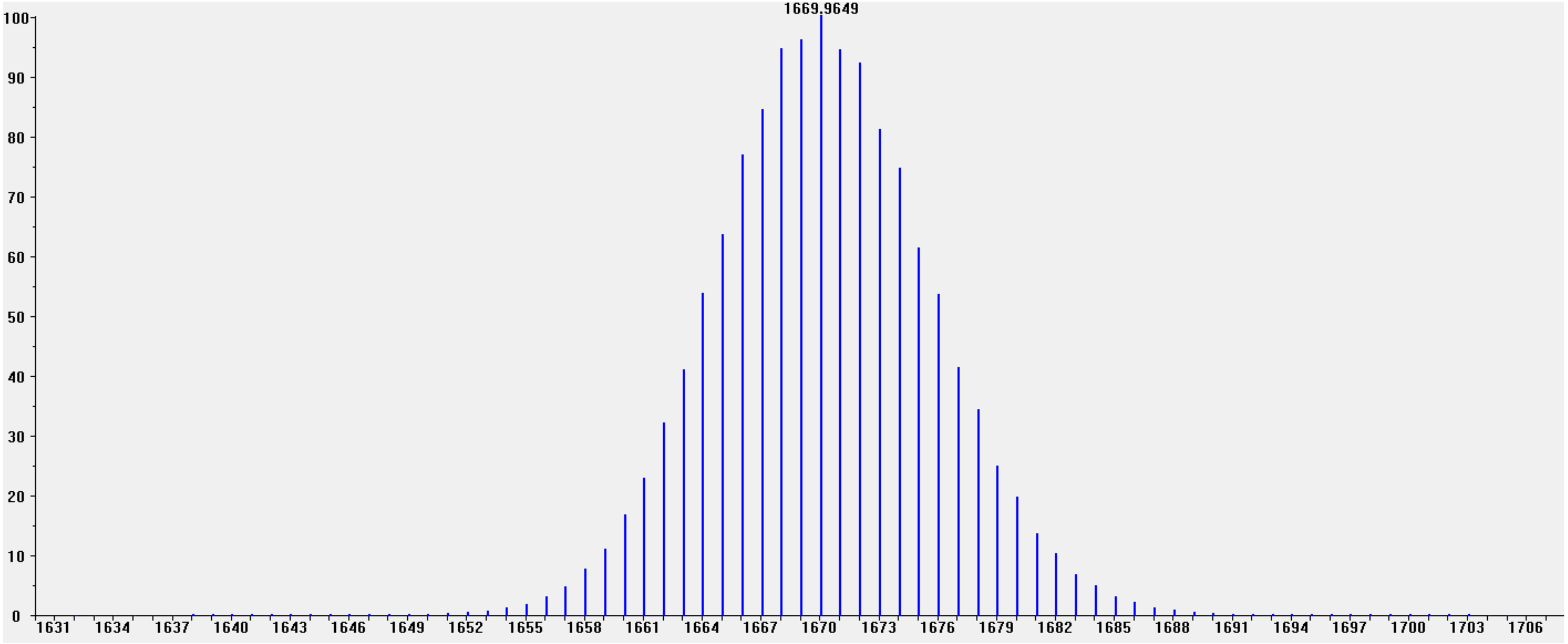

Figure S89b. Simulated mass cluster for $C_{34}H_{102}NO_{13}Si_8Sn_6^+$: {[MeSi(CH$_2$Sn(OH)$_2CH_2$SiMe$_3$)$_3$]$_2$ + CH_3CN + H_2O + H^+}$^+$. The horizontal axis shows the m/z values.

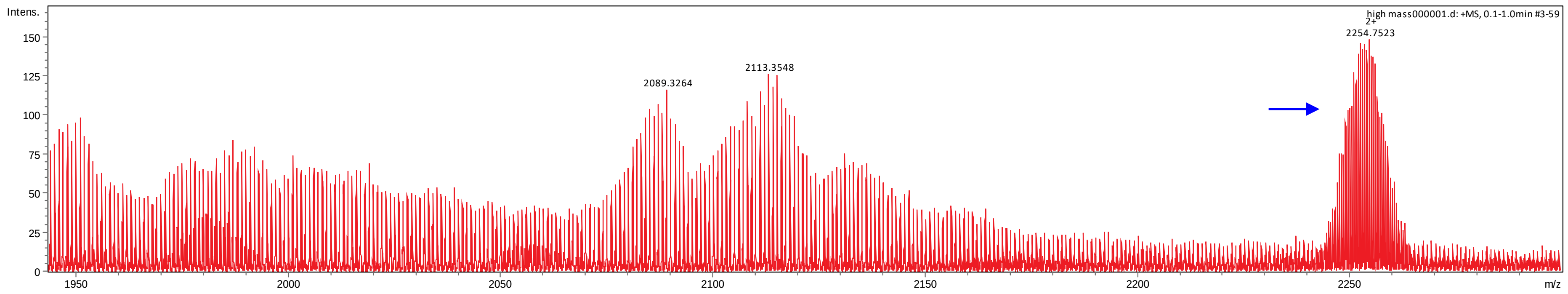

Figure S90. A fraction (c from Figure S85) of the ESI MS spectrum (positive mode) of **8**: mass cluster centred at m/z 2254.7523. The blue arrow refers to the subsequent Figure S91. The horizontal axis shows the m/z values.

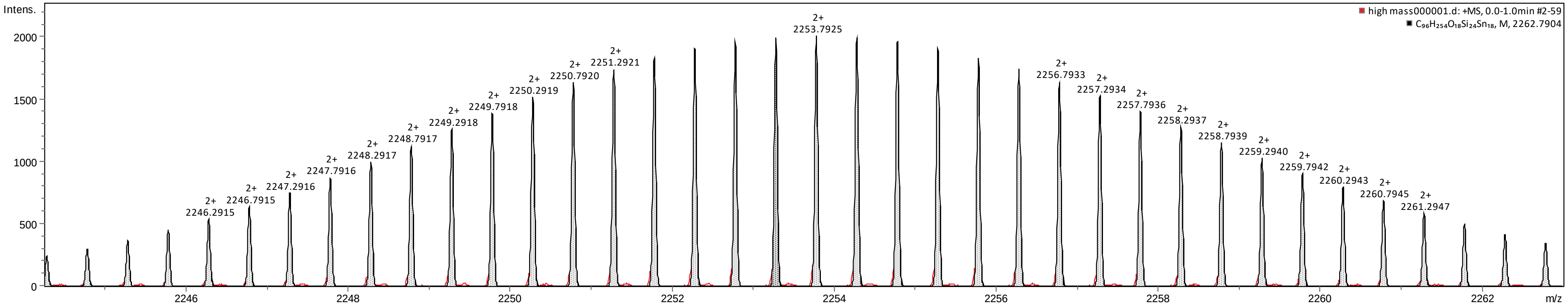

Figure S91. Simulated mass cluster for $C_{96}H_{254}O_{18}Si_{24}Sn_{18}^{2+}$: $\{[MeSi(CH_2SnCH_2SiMe_3O)_3]_6 + 2H^+\}^{2+}$. The horizontal axis shows the m/z values.

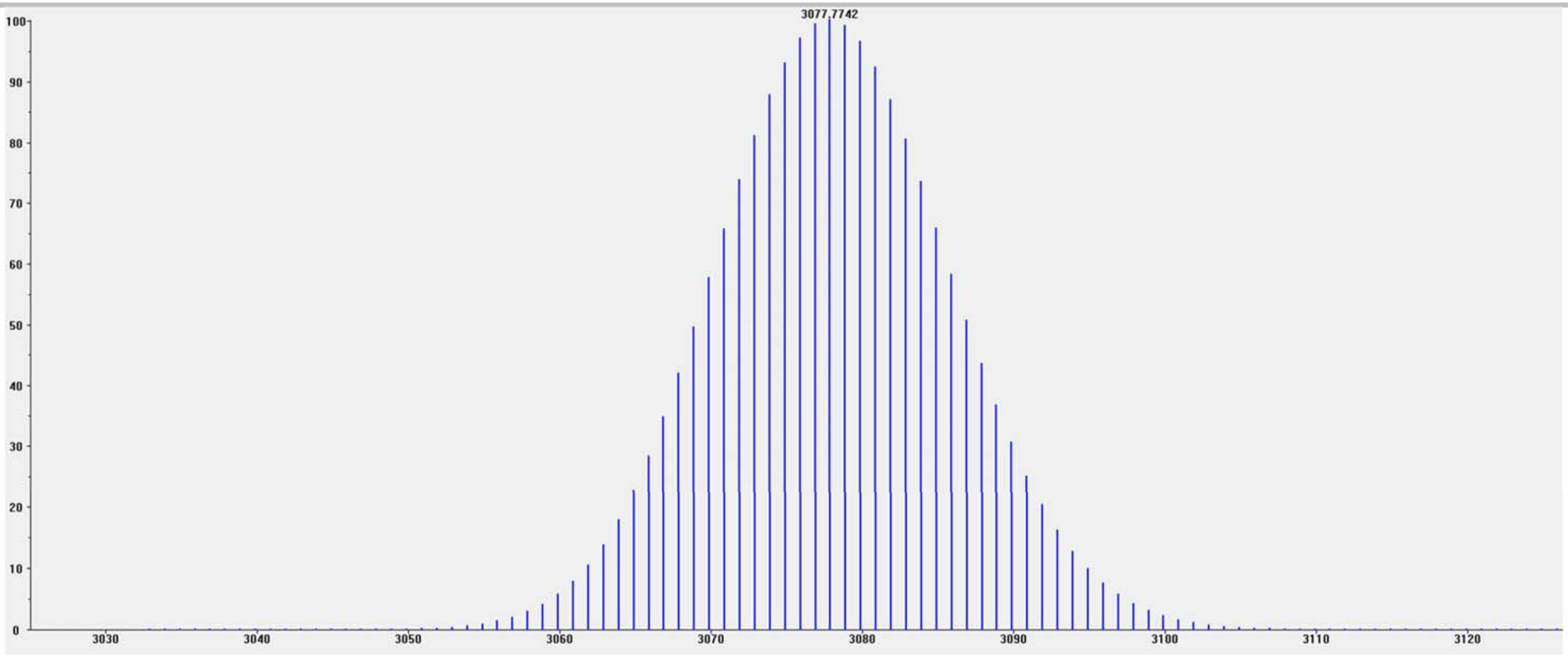

Figure S92. Simulated mass cluster of $C_{67}H_{176}NO_{13}Si_{16}Sn_{12}$ (3077.7742): $\{[MeSi(CH_2SnCH_2SiMe_3O)_3]_4 + H^+\}^+$. The horizontal axis shows the m/z values.

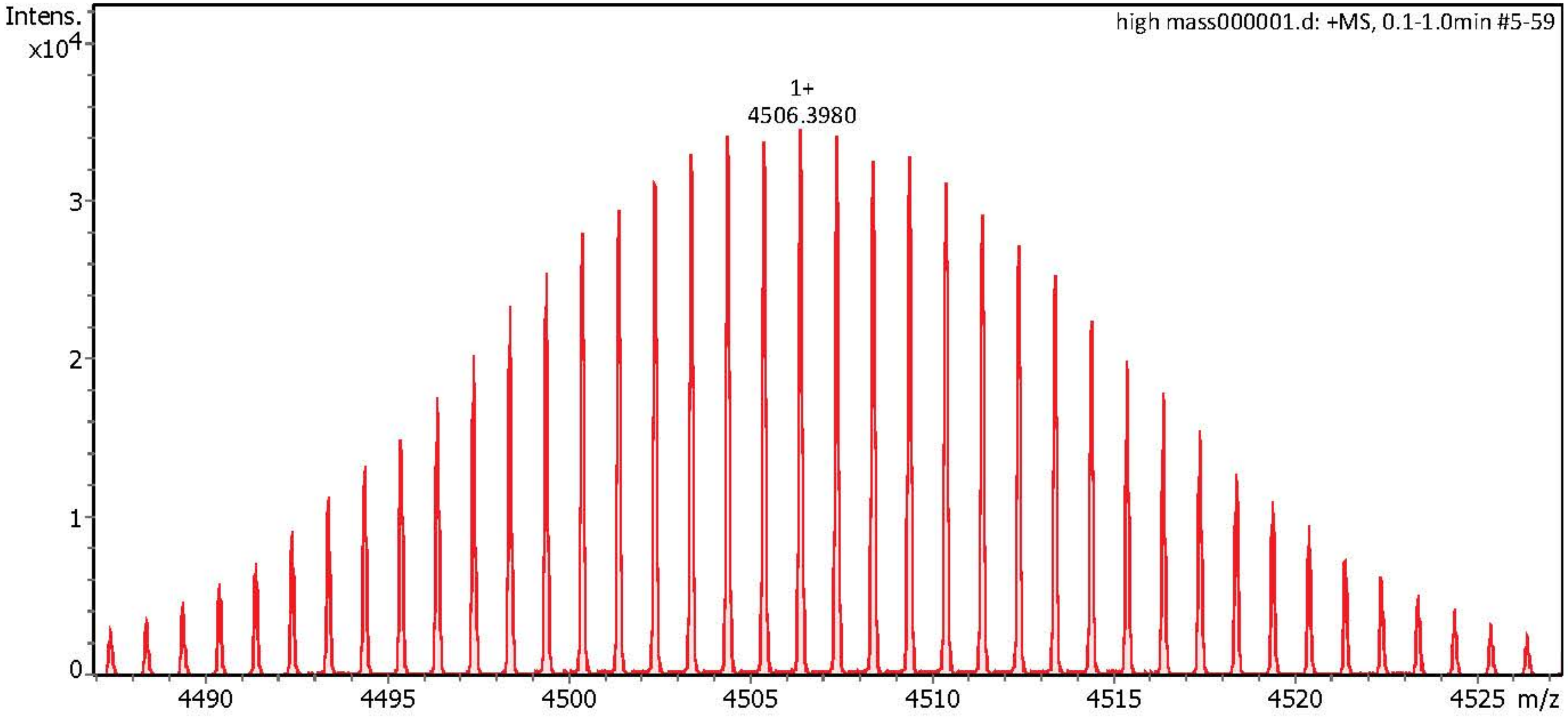

Figure S93. A fraction (d from Figure S85) of the ESI MS spectrum (positive mode) of **8**: mass cluster centred at m/z 4506.3980.

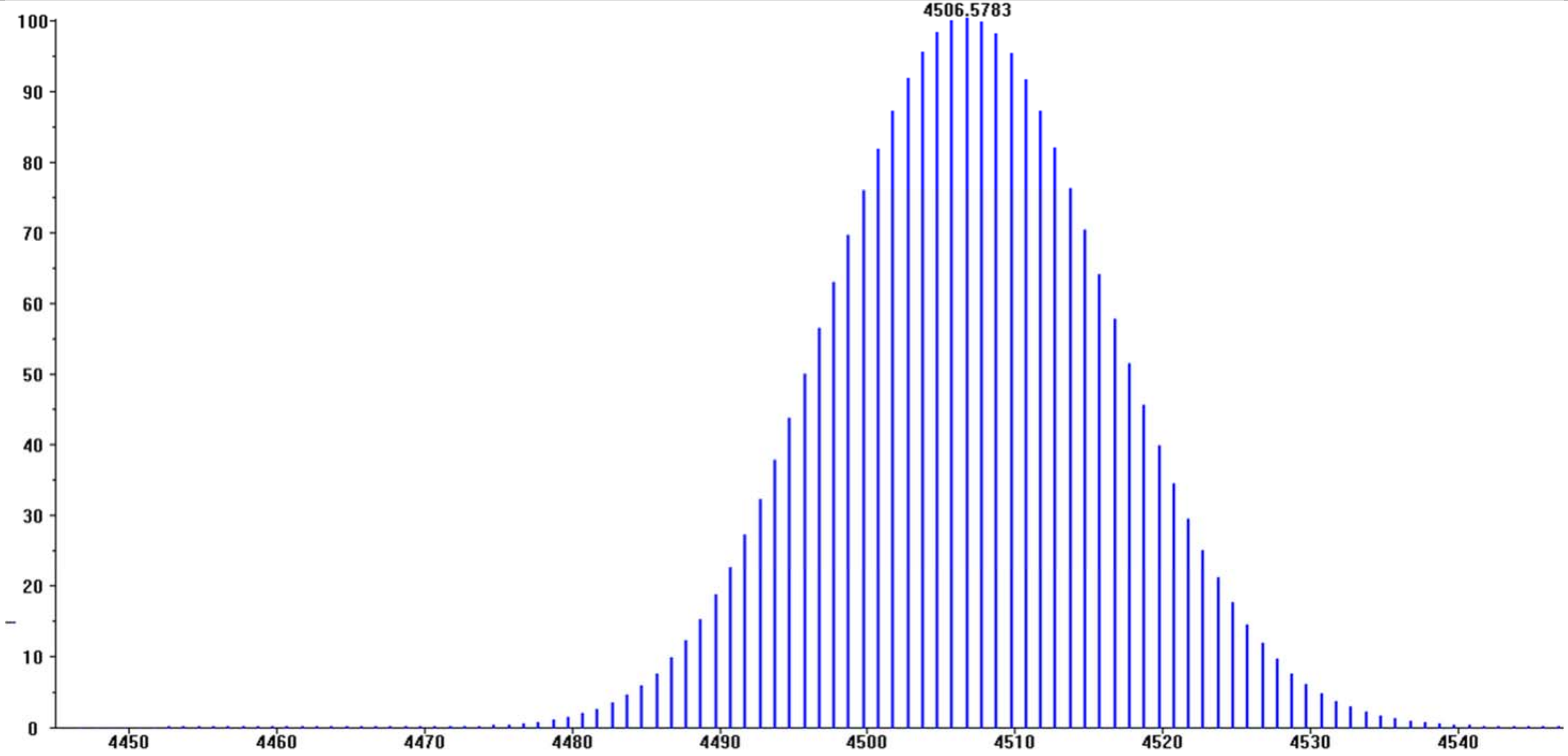

Figure S94. Simulated mass cluster for $C_{96}H_{253}O_{18}Si_{24}Sn_{18}^{+}$: {[MeSi(CH$_2$SnCH$_2$SiMe$_3$O)$_3$]$_6$ + H$^+$}$^+$. The horizontal axis shows the m/z values.

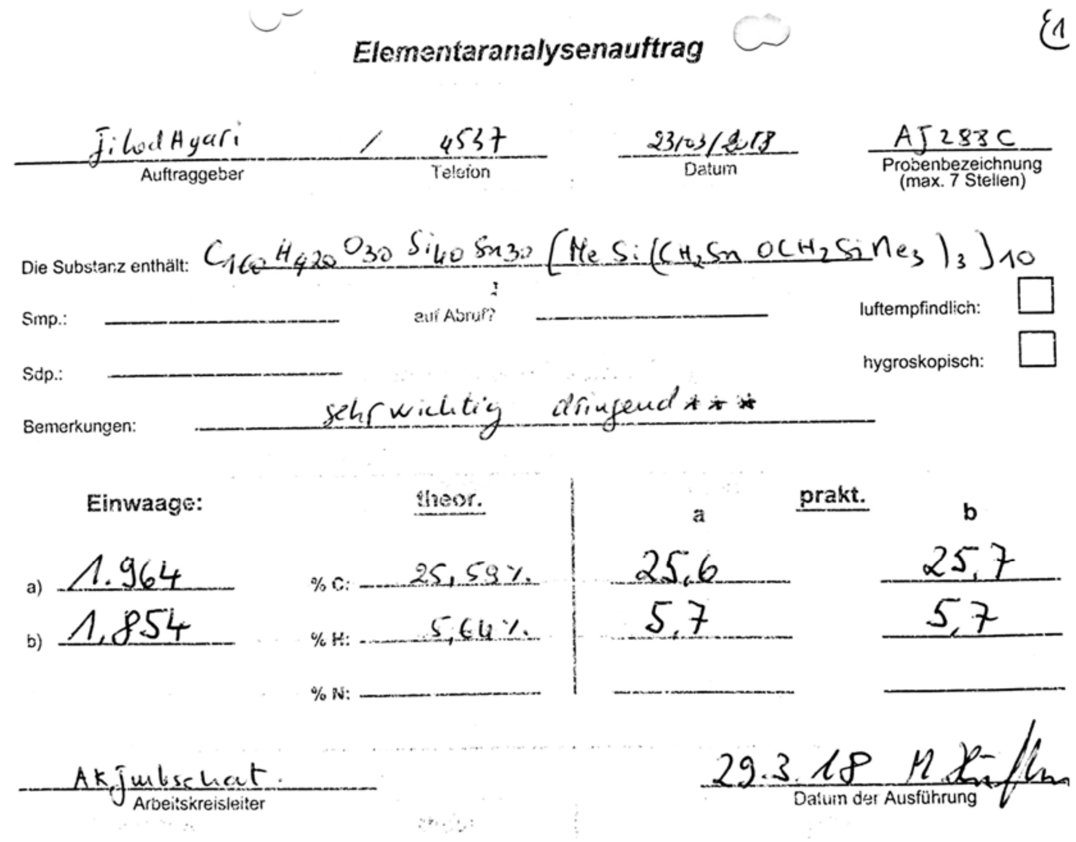

Elementaranalysenauftrag

E1

Jilod Hyari / 4537 | 23/03/2018 | AJ283C
Auftraggeber | Telefon | Datum | Probenbezeichnung (max. 7 Stellen)

Die Substanz enthält: $C_{160}H_{420}O_{30}Si_{40}Sn_{30}$ $[MeSi(CH_2SnOCH_2SiMe_3)_3]_{10}$

Smp.: ______ auf Abruf? ______ luftempfindlich: ☐

Sdp.: ______ hygroskopisch: ☐

Bemerkungen: sehr wichtig dringend***

Einwaage:		theor.	prakt. a	prakt. b
a) 1.964	% C:	25,59%	25,6	25,7
b) 1,854	% H:	5,64%	5,7	5,7
	% N:			

AK Jurkschat — Arbeitskreisleiter

29.3.18 [illegible] — Datum der Ausführung

Figure S95. Elemental analysis of the bulk microcrystalline material a single crystal of 8 was taken from to perform single crystal X-ray diffraction analysis.

Crystallographic data of compounds 2, 7, and 8.

Table S5. Crystallographic data.

	**2**	**7**	**8**
CCDC number	1995881	1995233	1953399
Chemical formula	$C_{58}H_{54}SiSn_3$	$C_{132}H_{144}O_{18}Si_6Sn_{18}\cdot 6\ CH_2Cl_2$	$C_{160}H_{414}O_{30}Si_{40}Sn_{30}\cdot 8\ CH_2Cl_2$
M_r (g·mol^{-1})	1135.17	4323.42	7503.19
Crystal system	Monoclinic	Monoclinic	Triclinic
Space group	$P2_1/c$	$P2_1/n$	*P-1*
Temperature (K)	173	173	100
a, *b*, *c* (Å)	18.2220(5), 10.8715(3), 26.1335(7)	14.0911 (5), 32.306 (2), 37.0724 (12)	16.822 (3), 23.161 (5), 41.207 (8)
α, β, γ (°)	90, 98.067(3), 90	90, 97.086 (4), 90	88.47 (3), 86.28 (3), 74.59 (3)
V (Å^3)	5125.8 (2)	16747.4 (13)	15444 (6)
Z	4	4	2
Radiation type	Mo *Ka*	Mo *Ka*	Cu *Ka*
μ (mm^{-1})	1.51	2.72	20.73
Crystal size (mm)	0.21 × 0.19 × 0.19	0.19 × 0.13 × 0.04	0.14 × 0.08 × 0.02
R_{int}	0.037	0.077	0.090
θ_{max} (°)	30.6	23.3	60.1
$(\sin \theta/\lambda)_{max}$ (Å^{-1})	0.725	0.556	0.562
$R[F^2 > 2s(F^2)]$, $wR(F^2)$, S	0.032, 0.073, 1.030	0.065, 0.131, 0.920	0.128, 0.350, 0.840

No. of reflections	54829	52875	57472
No. of reflections independent	14937	24054	33542
No. of reflections observed	11515	13695	11350
No. of parameters	536	1511	1183
No. of restraints	72	417	31
ρ_{max}, ρ_{min} (e Å^{-3})	0.63, -0.51	1.03, -0.80	1.26, -1.42

References

[1] U.-P. Apfel, D. Troegel, Y. Halpin, S. Tschierlei, U. Uhlemann, H. Gorls, M. Schmitt, J. Popp, P. Dunne, M. Venkatesan et al., *Inorganic chemistry* **2010**, *49*, 10117.

[2] M. Dub, *Organometallic compounds / Volume II Organometallic compounds of Germanium, Tin and Lead*, Springer-Verlag, Berlin, **1961**.

[3] M. Gielen, A. G. Davies, K. H. Pannell, E. R. T. Tiekink, *Tin chemistry. Fundamentals, frontiers, and applications / edited by Marcel Gielen … [et al.]*, Wiley, Chichester, **2008**.

[4] a) A. Jerschow and N. Mueller, J. Magn. Reson. A 1996, 123, 222−225. b) A. Jerschow and N. Mueller, J. Magn. Reson. A 1997, 125, 372−375.

Author Contributions

As part of her doctoral thesis, Mrs Jihed Ayari performed the synthesis of the compounds, characterized them and contributed in part to the design of the figures. Dr. Christian Göb and Dr. Michael Lutter performed the X-ray diffraction measurements and solved the structures, guided by Prof. Dr. Iris Oppel. Prof. Wolf Hiller performed the 1H-DOSY NMR spectra of compound **7**. Prof. Dr. Klaus Jurkschat administrated the project, guided Mrs Jihed Ayari during the work on her doctoral thesis and wrote the manuscript.
